# Straightforward Synthesis of Bis[(trifluoromethyl)sulfonyl]ethylated Isocoumarins from 2-Ethynylbenzoates

**DOI:** 10.1021/acs.joc.3c00611

**Published:** 2023-05-03

**Authors:** A. Sonia Petcu, Carlos Lázaro-Milla, F. Javier Rodríguez, Isabel Iriepa, Óscar M. Bautista-Aguilera, Cristina Aragoncillo, José M. Alonso, Pedro Almendros

**Affiliations:** † Instituto de Química Orgánica General, IQOG, CSIC, Juan de la Cierva 3, 28006 Madrid, Spain; ‡ Grupo de Lactamas y Heterociclos Bioactivos, Departamento de Química Orgánica, Unidad Asociada al CSIC, Facultad de Química, Universidad Complutense de Madrid, 28040 Madrid, Spain; § Universidad de Alcalá, Departamento de Química Orgánica y Química Inorgánica, 28805 Alcalá de Henares, Madrid, Spain; ∥ Instituto de Investigación Química Andrés M. del Río (IQAR), Universidad de Alcalá, 28805 Alcalá de Henares, Madrid, Spain

## Abstract

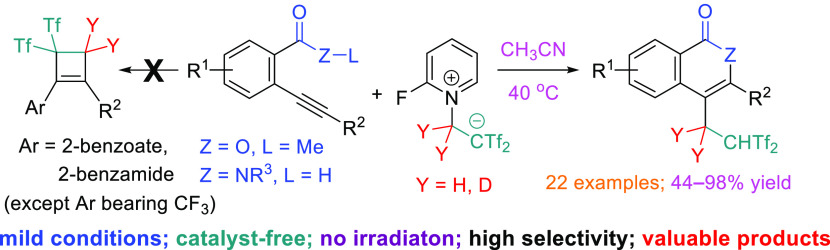

Herein, we report a facile isocoumarin and isoquinolone preparation by taking advantage of an initial bis(triflyl)ethylation [triflyl = (trifluoromethyl)sulfonyl] reaction, followed by heterocyclization, which contrasts with our previous results on cyclobutene formation. The efficiency of the catalyst- and irradiation-free heterocyclization/bis(triflyl)ethylation sequence showed exquisite dependence on the electronic nature of the substituents at the 2-ethynylbenzoate(benzamide) precursors. Molecular docking of model bis(triflyl)ethylated isocoumarins on human acetylcholinesterase (hAChE) revealed promising biological activities through selective coordination on both the catalytic active site and peripheral active site.

## Introduction

**Scheme 1:**
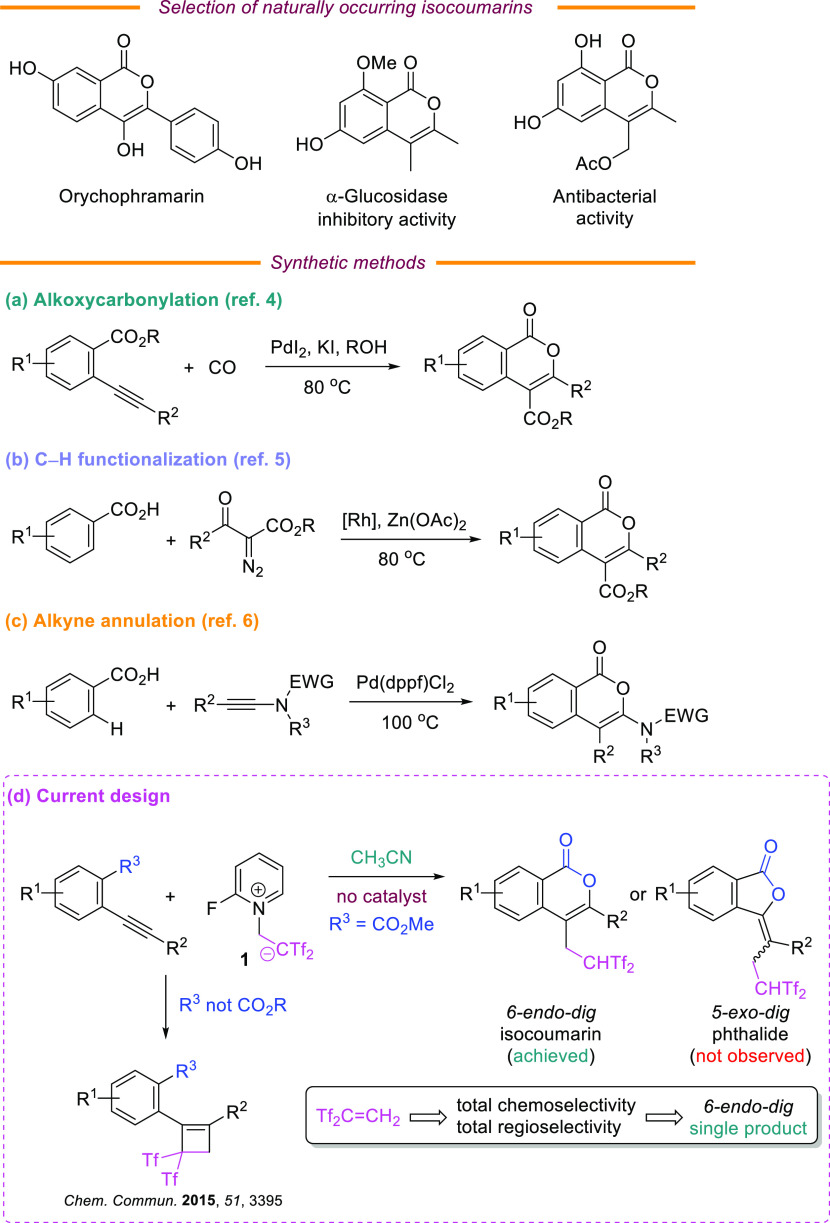
3,4-Disubstituted Isocoumarins: Natural Products, Previous Synthesis, and Concept

The 3,4-disubstituted isocoumarin framework displays interesting biological properties and it is widely spread in natural products and drugs (Scheme 1, top).^1,2^ Consequently, the preparation of this privileged core has been extensively documented.^3^ Notable synthesis of isocoumarins include the palladium-catalyzed oxidative alkoxycarbonylation of 2-alkynylbenzoic acids (Scheme 1a),^4^ the rhodium-catalyzed coupling of benzoic acids with α-diazocarbonyls (Scheme 1b),^5^ and the palladium-catalyzed cyclization of 2-iodobenzoic acids with ynamides (Scheme 1c).^6^ Despite the merits of previously reported procedures, most of them require the use of transition metals, hazardous reagents, or harsh reaction conditions. On the other hand, the SO_2_CF_3_ (Tf, triflyl) group is an appealing moiety because it combines the presence of two prominent functionalities, namely, the sulfone motif and the organofluorinated structure. The appearance of the strongly electron-withdrawing Tf moiety in drugs provides an improvement in the metabolic stability and lipophilicity.^7^ In addition, the enhancement of both water solubility in bioactive molecules and dyes and catalytic activity in organocatalysts by the presence of the Tf_2_CH substituent has been documented.^8^ Although the incorporation of the bis(triflyl)alkyl group is challenging, Yanai et al. pioneered the use of shelf-stable and readily available betaine **1** as source of Tf_2_C=CH_2_.^9^ Our group reported the reaction of reagent **1** with aryl-substituted alkynes for the preparation of cyclobutenes (Scheme 1d, left).^10^ Next, in order to explore the reactivity of aryl-substituted alkynes bearing an electron-withdrawing group at the *ortho* position, we decided to test the reaction of a 2-ethynylbenzoate. Notably, the intermolecular carbocyclization was totally suppressed, giving rise instead to the formation of a 3-aryl-4-bis[(trifluoromethyl)sulfonyl]ethylated isocoumarin. In addition, although the Tf_2_C=CH_2_ molecule released in the reaction medium is capable of reacting with any of the two carbons of the triple bond, the current process is totally regioselective leading exclusively to the 6-*endo*-*dig* oxycyclization product, while the 5-*exo*-*dig* oxycyclization product is not detected. Due to the interest in the product and the mildness of the protocol, we determine to study in more detail this intramolecular oxycyclization reaction with the concomitant transfer of the Tf_2_CH group (Scheme 1d, right).

## Results and Discussion

Annulation precursors, 2-ethynylbenzoates **2**, were easily prepared from 2-iodobenzoates by taking advantage of the Sonogashira reaction.^11^ Methyl 2-[(4-methoxyphenyl)ethynyl]benzoate **2a** was selected as a model substrate for studying the reaction with Yanai’s reagent **1**. After evaluating a series of reaction conditions, it was observed that the required isocoumarin **3a** was achieved in an excellent 95% yield by mixing equimolar amounts of compounds **1** and **2a** in acetonitrile at 40 °C. No conversion was observed when the reaction was tested at −40 °C, while a lot of unreacted starting material and isocoumarin **3a** were identified at rt. No improvements were detected by changing the solvent and amount of reagents or increasing the reaction temperature. It is important to mention the superacidic character of the Tf_2_CH group, which resulted in the obtention of sodium salt **3a** following column chromatography on silica gel.^12^

**Scheme 2:**
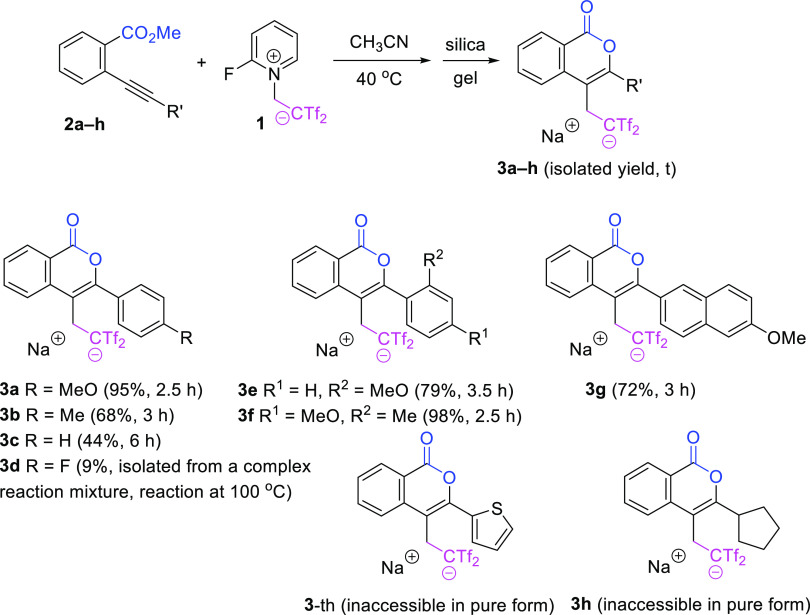
Controlled Preparation of 3-Aryl-4-bis(triflyl)ethylated Isocoumarins **3a**–**h**

Upon identification of satisfactory reaction conditions for the formation of heterocycle **3a**, we focused on screening the substrate scope (Scheme 2). Taking into account the high attainability of 2-ethynylbenzoates, the above sequence should permit access to isocoumarins displaying good structural variety. Electron-donating (Me, MeO) and neutral (H) substituents were accommodated in the arene ring at the alkyne functionality of the cyclization precursors **2** to form 3,4-difunctionalized isocoumarins **3** in good yields. To figure out the tolerance of heteroaryl substitution on the alkyne terminal, a 2-thienyl-capped alkyne was examined. The reaction takes place but it was a bit messy and isocoumarin **3**-th cannot be obtained in pure enough form for synthetic purposes. The steric influence was not very important because 2-methoxy substituted heterocycle **3e** was obtained in comparable figures to its 4-methoxy isomer **3a**. In addition, a naphthyl nucleus was situated in adduct **3g**. However, the placement of an electron-poor substituent was detrimental to the transformation, e.g., high temperature (100 °C) was necessary for the completion of the reaction of **2d** bearing a 4-fluorophenyl group, which resulted in a complex reaction mixture. Indeed, the efficiency of the oxycyclization/bis(triflyl)ethylation sequence showed exquisite dependence on the electronic nature of the alkyne substitution, and alkyl-terminated alkyne **2h** formed isocoumarin **3h** accompanied by side reactions, which make its isolation in the pure form impossible. The above observations indicate the relevance of electron-rich substituents at the alkyne end in this functionalization/heterocyclization sequence. In no case, the formation as minor products of bis(triflyl)cyclobutenes arising from a [2 + 2] cyclization reaction was detected in the crude reaction mixtures by ^1^H NMR. Neither the genesis of the phthalide (5-membered lactone) through competitive 5-*exo*-*dig* oxycyclization was observed, and the 6-*endo*-*dig* oxycyclization was the only operative path.

**Scheme 3:**
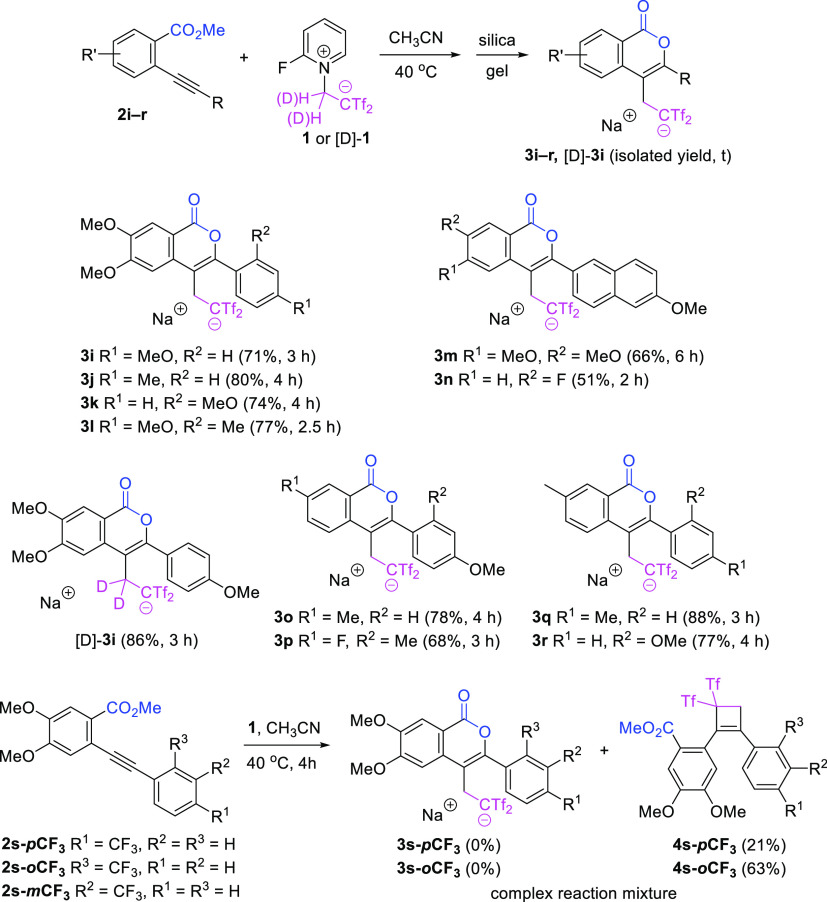
Controlled Preparation of 3-Aryl-4-bis(triflyl)ethylated Isocoumarins **3i**–**r**

Next, we decided to investigate the generality of the reaction in regard to the substitution on the benzoate moiety (Scheme 3). Again, the identity of the substituent had a significant impact on the efficiency of the cascade sequence. The presence of electron-releasing groups (Me, MeO) such as in cyclization precursors **2i**–**2m** and **2o**,**q**,**r** was found to contribute positively. Substrates **2j** and **2m** bear electron-donating substituents placed at the para-position on the two aryl moieties (benzoate as well as arene at the alkyne), which should assist the lactonization sequence. The regioselectivity trend is dictated by the substituent at the alkyne end which lacks an electron-withdrawing moiety (carboxylate) and may better stabilize the carbocation initially formed by the attack of the alkyne on Tf_2_C=CH_2_, suppressing the 5-*exo*-*dig* path and allowing the selective formation of isocoumarins through the *6*-*endo*-*dig* path. Likewise, deuterated 3-aryl-4-bis(triflyl)ethylated isocoumarin [D]-**3i** was conveniently obtained by way of the reaction of 2-ethynylbenzoate **2i** and deuterated betaine [D]-**1**. Although the installation of electron-withdrawing groups influenced negatively, the presence of a fluorine atom was tolerated and isocoumarins **3n** and **3p** were achieved in reasonable yields. On the contrary, 2-((4-(trifluoromethyl)phenyl)ethynyl)benzoate **2s**-***p*****CF**_**3**_ and 2-((2-(trifluoromethyl)phenyl)ethynyl)benzoate **2s**-***o*****CF**_**3**_ did not perform well in the oxycyclization reaction, and cyclobutenes **4s**-***p*****CF**_**3**_ and **4s**-***o*****CF**_**3**_ were isolated as sole products in 21% yield (40% conversion) and 63% yield, respectively. The reaction between 2-((3-(trifluoromethyl)phenyl)ethynyl)benzoate **2s**-***m*****CF**_**3**_ and reagent **1** resulted in a complex reaction mixture. From these experiments, the precise electronic effects that dictate the reactivity control may be inferred. Taking into account the results from Schemes 2 and 3, a tendency may be established. The presence of electron-rich substituents both at the benzoate and the alkyne favors the heterocyclization/functionalization cascade, but the electronic nature of the substituent at the alkyne end is a more influential controlling factor, also imposing the regioselectivity of the nucleophilic attack to Tf_2_C=CH_2_.

**Scheme 4:**
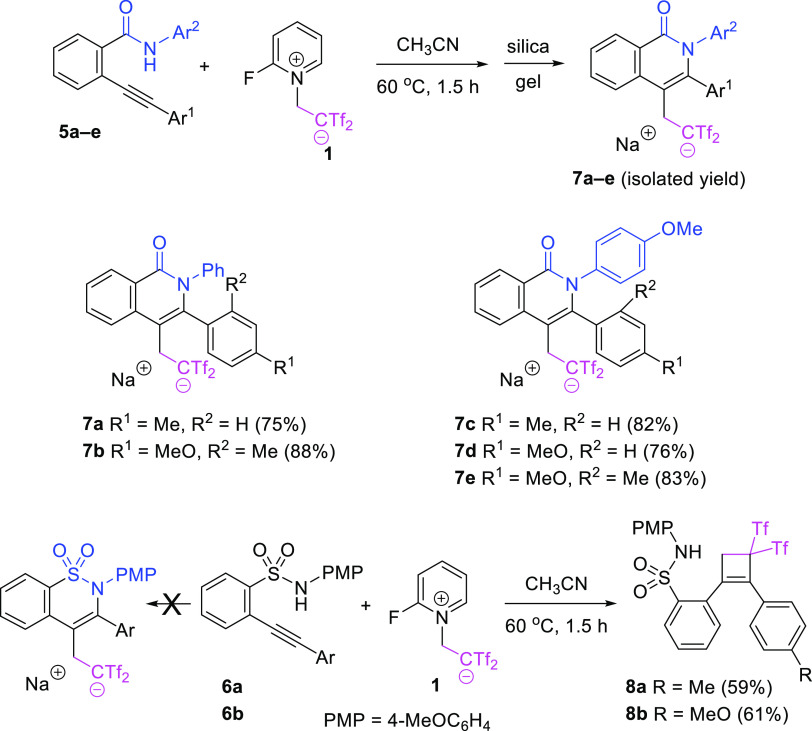
Controlled Preparation of 3-Aryl-4-bis(triflyl)ethylated Isoquinolinones **7a**–**e**

Taking into account the relevance of the nitrogen heterocycles isoquinolin-1(2*H*)-one and benzosultam as key components of natural products and pharmaceuticals, we decided to test 2-ethynylbenzamides **5** and 2-ethynylbenzenesulfonamides **6** as cyclization precursors, instead of 2-ethynylbenzoates **2**. While benzamide-derived arylethylenes **5** were prone to the 6-*endo*-*dig* azacyclization/functionalization cascade to form isoquinolin-1(2*H*)-ones **7**, their benzenesulfonamide counterparts **6** experienced the intermolecular [2 + 2] cyclization to provide (2-aryl-3,3-bis(triflyl)cyclobut-1-en-1-yl)benzenesulfonamides **8** (Scheme 4). No differences were encountered when the mixture of **6b** and **1** were heated in acetonitrile at 60 or 100 °C (sealed tube). The regiocontrol on the cyclobutene formation step is imparted by the electron-rich substituent at the alkyne moiety, which avoids the formation of regioisomeric 2-(2-aryl-4,4-bis(triflyl)cyclobut-1-en-1-yl)benzenesulfonamides. The benzosultam core was unachievable because the presence of the less nucleophilic benzenesulfonamide group imposed a different chemoselectivity. The above results point to the nontrivial predictability of the reactivity pattern when reagent **1** and a functionalized alkyne are put together, but at the same time, the exquisite chemo- and regioselectivities of the reactions between betaine **1** and the alkyne moiety are remarkable. Energy-dispersive X-ray analysis (EDX) in representative isocoumarins **3a**, **3g**, and **7b** pointed to the identification of sodium and calcium as major metal components (see the Supporting Information). Consequently, products are not obtained as pure sodium salts.

In order to clarify the possible role of adventitious water in the reaction mechanism, two control experiments were carried out using the reaction between 2-ethynylbenzoate **2i** and zwitterion **1** as a model (Supporting Scheme S1). The first experiment was performed under optimized conditions but in the presence of 1 equiv of water. In the event, the addition of water was slightly beneficial and formed a bicycle **3i** in 78% yield (71% yield under standard conditions,^13^Scheme 3). By contrast, the use of 3 Å molecular sieves (MS) as a water-trapping agent was detrimental and resulted in the formation of **3i** in a diminished 25% yield after a prolonged reaction time. In addition, with the aim to observe whether there is an increase or a decrease in the desired product formation when strictly dehydrated acetonitrile was used as solvent followed by the controlled addition of water, additional experiments were conducted (Supporting Table S1). When the reaction was run in rigorously anhydrous acetonitrile, almost absence of formation of **3i** was observed (entry 1, Table S1), while the addition of 1 equiv of water resulted in the formation of **3i** in a poor 32% yield (entry 2, Table S1). Isocoumarin **3i** was obtained in yields around 70% with the addition of 2 equiv or largest amounts of water (entries 3–5, Table S1).

**Scheme 5:**
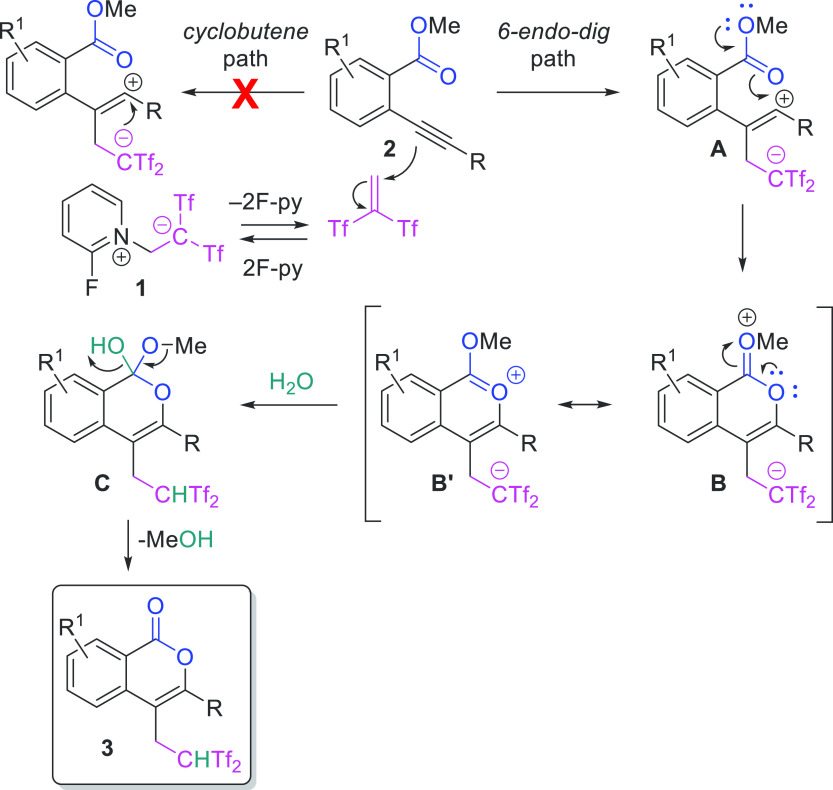
Plausible Pathway for the Functionalization–Oxycyclization Sequence

The reaction is proposed to proceed initially via a nucleophilic attack of the alkyne moiety of **2** to the highly polar olefin Tf_2_C=CH_2_, which is generated in situ from betaine **1** (Scheme 5). This interaction resulted in the formation of zwitterionic species **A**, which should suffer annulation across the carbonyl oxygen, triggered by the methoxy group conjugation, and the carbocation to generate intermediate **B**. This intermediate **B** is stabilized by resonance with species **B’**. Subsequent water addition toward the carbonylic carbon of intermediates **B**/**B’** should form species **C**, which should evolve to 3,4-disubstituted isocoumarins **3** through methanol release. The reaction between 2-ethynylbenzoate **2i** and zwitterion **1** in the presence of H_2_^18^O yielded ^18^O-unlabeled isocoumarin **3i**, which should support the above addition–elimination path. The regiochemical outcome (6-*endo*-*dig* path) should be a consequence of the preferential formation of intermediate **A** (having the carbocation closed to the nonelectron-deficient aromatic moiety), abolishing the *5*-*exo*-*dig* path.

The biological activity of the whole isochromane family has been widely explored in the context of neurological disorders.^14^ In this regard, 4-isochromanones decorated with fluoroaryl groups have been recently reported as good candidates against Alzheimer’s disease due to predicted coordination with human acetylcholinesterase (hAChE).^15^ We have therefore envisioned compounds **3** exhibiting more flexible fluoroalkyl substituents and limited steric hindrance as alternate inhibitors of hAChE, and consequently good candidates for docking study.

**Figure 1:**
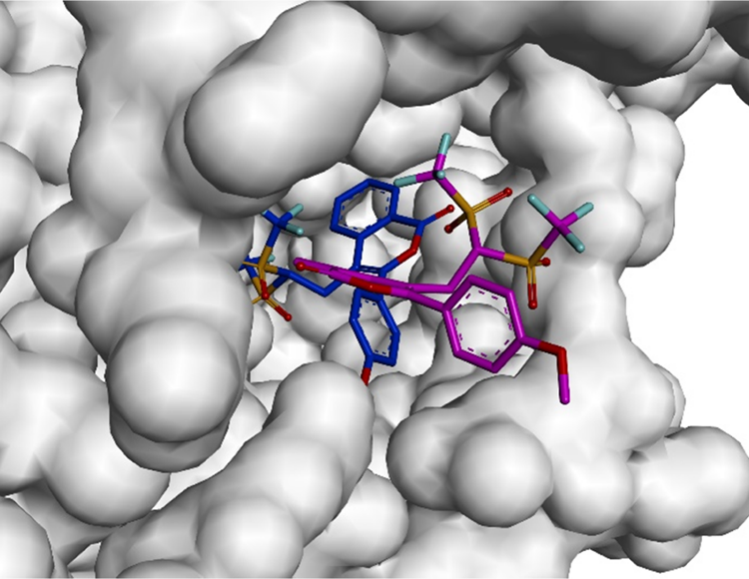
Surface representation of hAchE, **3a**, Mode I (blue) and **3a**, Mode II (pink) complexes.

The spatial conformation of compounds **3a** and **3l** was therefore explored by molecular docking in order to reveal the differences in their binding modes to the hAChE.^16^Figure 1 shows the docking orientation of **3a** in the active site of hAChE. Two binding modes (Modes I and II) can be proposed for this compound, resulting in its higher affinity with the catalytic active site (CAS) (binding energy: −9.8 kcal/mol) than with the peripheral anionic site (PAS) (binding energy: −8.2 kcal/mol).

**Figure 2:**
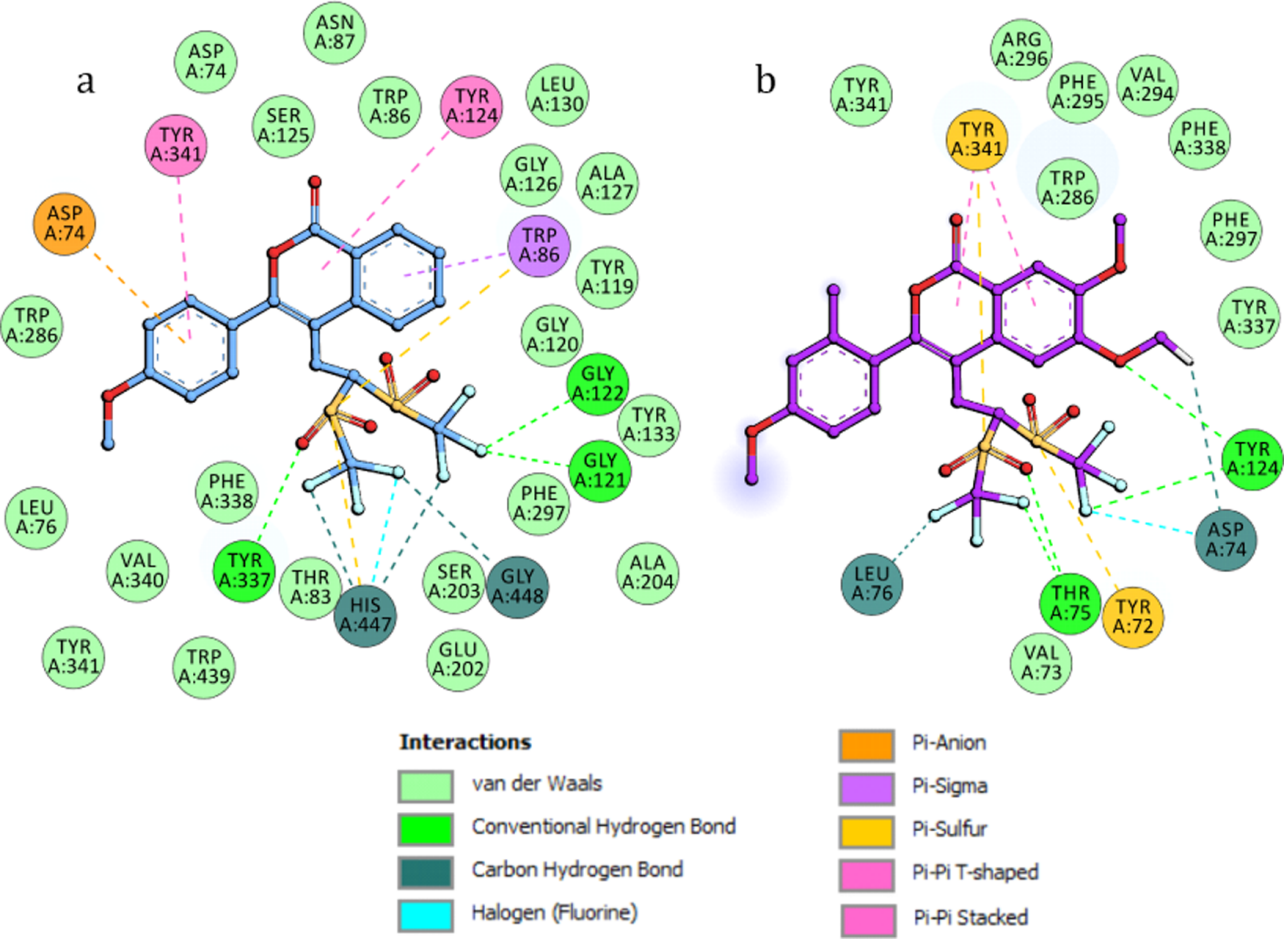
Amino acids in the binding site of hAChE interacting with ligand **3a**, Mode I (a) and ligand **3l, Mode II** (b).

At the CAS (Mode I), compound **3a** forms a stable network of interactions through triflyl groups (Figure 2a). The F atoms of the CF_3_ groups interacted with key amino acids to form strong halogen interactions. F atoms held Gly448, His447 (amino acid of the catalytic triad), Gly121, and Gly122 (oxyanion hole), resulting in the presence of O···F, N···F, C–H···F, and N–H···F interactions. The sulfonyl group was found to form a hydrogen bond with Tyr337 and π–sulfur interactions with His447 and Trp86. Near the bottom of the gorge, the benzene ring of the isocoumarin moiety established π–sigma interactions with Trp86, a residue known for attracting the quaternary amine of the acetylcholine. The methoxyphenyl and lactone moieties lay in the middle of the gorge between the CAS and PAS interacting with the amino acids Tyr341 and Tyr124 through π–π T-shaped interactions. Additionally, the phenyl ring forms π–anion interactions with Asp74 (PAS) (Figure 2a).

In Mode II, compound **3a** is located in the pocket-forming PAS where Trp286 interacts with the methoxyphenyl ring via the π–π stacking interaction, and with the lactone moiety forming two hydrogen bonds.^16^ In this situation, the halogen atoms interact with Val73, Asp74, and Leu76, which also highly contributed to the stabilization of the complex. On the other hand, sulfonyl groups form a hydrogen bond with Thr75 and π–sulfur interaction with Tyr72.

Interestingly, docking experiments showed that the more sterically demanding isocoumarin **3l** is selectively arranged in a position similar to that found for compound **3a** in Mode II. The most energetically favored binding mode places the ligand in the PAS with isocoumarin moiety stacking with Tyr341 residue and no binding of the compound was observed at the catalytic triad (Figure 2b).^16^ Based upon docking experiments, it can be proposed that the less substituted compound (**3a**) provides a better chance for the triflyl group to access the active site on the bottom of the gorge.

## Conclusions

In summary, we have developed a controlled synthesis of bis(triflyl)ethylated isocoumarin and isoquinolone cores by the interaction of 2-ethynylbenzoates or 2-ethynylbenzamides with Tf_2_C=CH_2_ without catalysts or light irradiation. This sequence is initiated by intermolecular nucleophilic attack of the triple bond to Tf_2_C=CH_2_, followed by concomitant heterocyclization. Precise electronic effects dictated the reactivity control, with electron-rich substituents both at the benzoate(benzamide) as well as on the alkyne favoring the heterocyclization/functionalization cascade. In addition, the presence of triflyl groups tethered to the isocoumarin core enhances hAChE inhibition according to molecular docking experiments, pointing to the promising biological importance of bis(triflyl)ethylated isocoumarins. Two selective binding conformations may be devised through coordination on both CAS and PAS regions, also dependent on the steric nature of the isocoumarin ligands.

## Data Availability

The data underlying this study are available in the published article and its Supporting Information

## Supporting Information Available

Experimental procedures, characterization data of new compounds, and copies of NMR spectra (PDF)
