# Supplementary material for: Straightforward Synthesis of Bis[(trifluoromethyl)sulfonyl]ethylated Isocoumarins from 2-Ethynylbenzoates
Source: J Org Chem. 2023 May 3;88(11):7373–80. doi: 10.1021/acs.joc.3c00611 (PMC10242750; doi:10.1021/acs.joc.3c00611)
Supplement: Supplementary file 1 — jo3c00611_si_001.pdf [file jo3c00611_si_001.pdf]

Supplementary Information for the paper

Straightforward Synthesis of Bis[(trifluoromethyl)sulfonyl]ethylated Isocoumarins from 2-Ethynylbenzoates

A. Sonia Petcu,[†] Carlos Lázaro-Milla,[‡] F. Javier Rodríguez,[‡] Isabel Iriepa,[§,⊥] Óscar M. Bautista-Aguilera, [§,⊥] Cristina Aragoncillo,[‡] José M. Alonso,[*,‡] and Pedro Almendros[*,†]

[†]Instituto de Química Orgánica General, IQOG-CSIC, Juan de la Cierva 3, 28006-Madrid, Spain

[‡]Grupo de Lactamas y Heterociclos Bioactivos, Departamento de Química Orgánica I, Unidad Asociada al CSIC, Facultad de Química, Universidad Complutense de Madrid, 28040-Madrid, Spain

[§]Universidad de Alcalá, Departamento de Química Orgánica y Química Inorgánica, 28805-Alcalá de Henares, Madrid, Spain

[⊥]Instituto de Investigación Química Andrés M. del Río (IQAR), Universidad de Alcalá, 28805-Alcalá de Henares, Madrid, Spain

E-mail: josalo08@ucm.es; palmendros@iqog.csic.es

Table of Contents

General Methods S2

Scheme S1 and Table S1. Effect of Water S2,S3

Experimental Section S3–S32

^{1}H NMR, ^{13}C NMR, ^{19}F NMR, and ^{23}Na NMR Spectra S33–S114

Molecular Docking Studies S115–S119

EDX Spectra S120–S125

General Methods: ^{1}H NMR and ^{13}C NMR spectra were recorded on a Bruker Avance AVIII-700 with cryoprobe, Bruker AMX-500, Bruker Avance-300, or Varian VRX-300S. NMR spectra were recorded in $CDCl_3$ solutions, except otherwise stated. Chemical shifts are given in ppm relative to TMS (^{1}H, 0.0 ppm), or $CDCl_3$ (^{1}H, 7.27 ppm; ^{13}C, 76.9 ppm), or acetone-d_6 (^{1}H, 2.05 ppm; ^{13}C, 206.3 ppm), or C_6D_6 (^{1}H, 7.16 ppm; ^{13}C, 128.0 ppm), or CD_3CN (^{1}H, 1.94 ppm; ^{13}C, 118.2 ppm), or DMSO-d_6 (^{1}H, 2.50 ppm; ^{13}C, 39.5 ppm). Chemical shifts in ^{19}F are given in ppm relative to (trifluoromethyl)benzene ($C_6H_5CF_3$) in $CDCl_3$ (^{19}F, –63.7 ppm). Chemical shifts in ^{23}Na are given in ppm relative to NaCl in D_2O (^{23}Na, 0.00 ppm). Structural assignments were made with additional information from gCOSY, gHSQC, and gHMBC experiments. Low and high resolution mass spectra were taken on an AGILENT 6520 Accurate-Mass QTOF LC/MS spectrometer using the electronic impact (EI) or electrospray modes (ES) unless otherwise stated. IR spectra were recorded on a Bruker Tensor 27 spectrometer. All commercially available compounds were used without further purification. Microwave irradiation was carried out in a Monowave 300 from Anton Paar GmbH. The reaction temperatures during microwave heating were measured with an internal infrared sensor. Column chromatography was carried out using silica gel 60, 0.04-0.06 mm, for flash chromatography (230-400 mesh ASTM) provided by Scharlau. For reactions that require heating, a heating-on block was used. All commercially available compounds were used without further purification.

Scheme S1. Effect of Water on the Functionalization-Oxycyclization Sequence

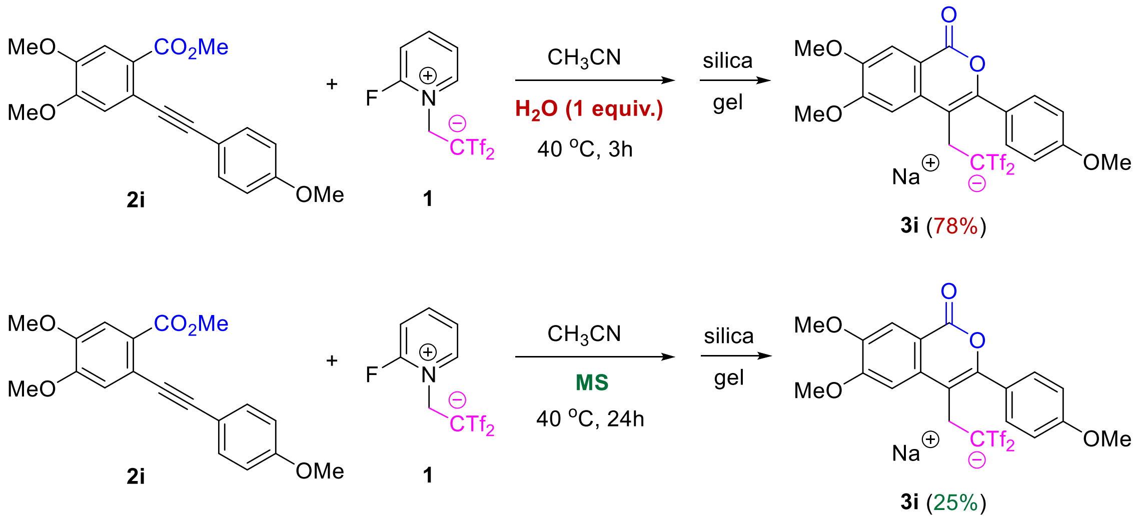

Table S1. Effect of Water Addition on the Reaction of Formation of Isocoumarin 3i

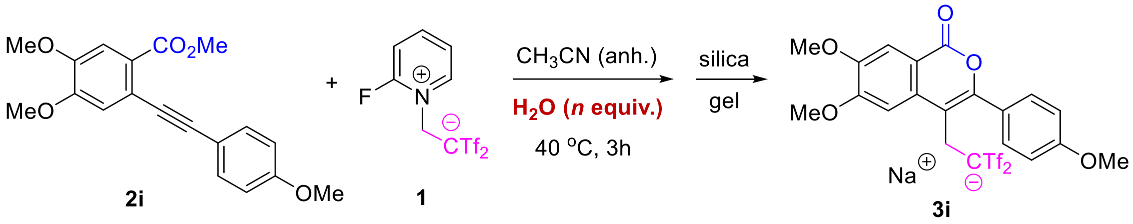

entry	H_2O (*n* equiv)	yield **3i** (%)[a]
1	*n* = 0	5
2	*n* = 1	32
3	*n* = 2	70
4	*n* = 3	73
5	*n* = 4	77

[a]Yield of pure, isolated product.

Zwitterion **1** was synthesized according to a literature procedure: Yanai, H.; Takahashi, Y.; Fukaya, H.; Dobashi, Y.; Matsumoto, T. *Chem. Commun*. **2013**, *49*, 10091.

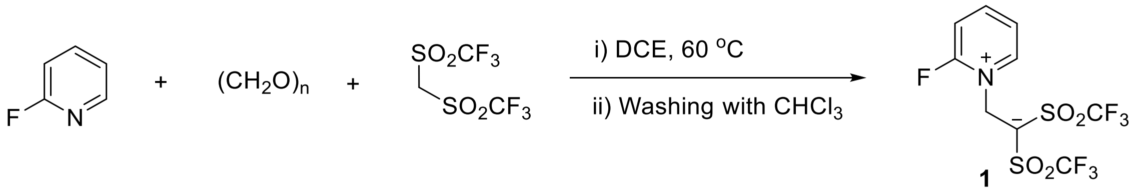

To a solution of Tf_2CH_2 (281 mg, 1.00 mmol) in 1,2-dichloroethane (6.0 mL), paraformaldehyde (90% purity, 73.0 mg, 2.19 mmol) and 2-fluoropyridine (172 μL, 2.00 mmol) were added at room temperature. After being stirred for 8 h at 60 °C, the reaction mixture was concentrated under reduced pressure. The resulting residue was washed with $CHCl_3$ (1.0 mL x 3) to give zwitterion **1** in 91% yield (356 mg, 0.915 mmol).

1. Synthesis of 2-ethynylbenzoates 2, 2-ethynylbenzamides 5 and 2-ethynylbenzenesulfonamides 6:

1.1. 2-Ethynylbenzoates **2**

2-Ethynylbenzoates **2** were prepared according to standard Sonogashira reaction conditions. Iodoesters **9** (1.1 equiv.), CuI (5 mol%) and $PdCl_2(PPh_3)_2$ (5 mol%) were placed in an oven-dried round bottom flask, and purged under Ar atmosphere. Then, TEA (3.8 mL/mmol) was added and

the mixture was stirred at room temperature for 5 minutes. After that time, the corresponding alkyne (1 equiv.) was added in one portion and the reaction was stirred at the same temperature until disappearance of the starting material (TLC). The crude reaction mixture was filtered through a celite pad, and the solvent was evaporated under reduced pressure. The mixture was purified on column chromatography using mixtures of hexanes and AcOEt.

Table S2

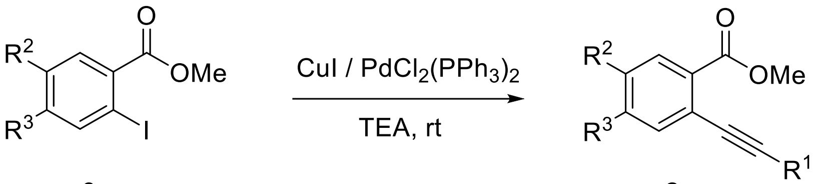

Entry	9	R^1	R^2	R^3	2 (%)
1	**9a**	4-OMe-C_6H_4	H	H	**2a** (98)
2	**9b**	4-Me-C_6H_4	H	H	**2b** (68)
3	**9c**	Ph	H	H	**2c** (98)
4	**9d**	4-F-C_6H_4	H	H	**2d** (98)
5	**9e**	2-OMe-C_6H_4	H	H	**2e** (98)
6	**9f**	2-Me-4-OMe-C_6H_3	H	H	**2f** (87)
7	**9g**	6-methoxynaphthyl	H	H	**2g** (85)
8	**9h**	Cyclopentyl	H	H	**2h** (15)
9	**9i**	4-OMe-C_6H_4	OMe	OMe	**2i** (81)
10	**9j**	4-Me-C_6H_4	OMe	OMe	**2j** (63)
11	**9k**	2-OMe-C_6H_4	OMe	OMe	**2k** (76)
12	**9l**	2-Me-4-OMe-C_6H_3	OMe	OMe	**2l** (86)
13	**9m**	6-methoxynaphthyl	OMe	OMe	**2m** (69)
14	**9n**	6-methoxynaphthyl	F	H	**2n** (88)
15	**9o**	4-OMe-C_6H_4	Me	H	**2o** (76)
16	**9p**	2-Me-4-OMe-C_6H_3	F	H	**2p** (75)
17	**9q**	4-Me-C_6H_4	Me	H	**2q** (85)
18	**9r**	2-OMe-C_6H_4	Me	H	**2r** (97)
19	**9s-*p*-CF_3**	4-CF_3-C_6H_4	OMe	OMe	**2s-*p*-CF_3** (59)
20	**9s-*o*-CF_3**	2-CF_3-C_6H_4	OMe	OMe	**2s-*o*-CF_3** (99)
21	**9t**	cyclopropyl	H	H	**2t** (47)
22	**9u**	2-thiophenyl	H	H	**2u** (77)

Compounds **2a, 2b, 2c, 2e, 2u,**[1] **2d, 2t**[2] **2o, 2q**[3] **2i**[4] and **2h**[5] were prepared according to the general procedure, exhibiting analytical data in accordance to previous reports.

Compound **2f**

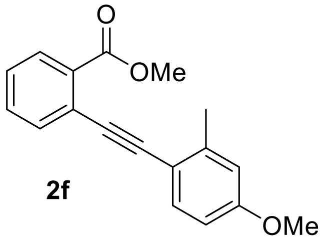

2f

Following the general procedure, starting from 127 mg of iodoester **9f**, 90 mg (87%) of compound **2f** were obtained as a yellow thick oil, after purification on column chromatography using hexanes/AcOEt (7:1) as eluent.

^{1}H NMR ($CDCl_3$, 300 MHz, 25 °C): δ = 7.98 (1H, ddd, *J* = 7.8, 1.5, 0.5 Hz, CH_{Ar}), 7.66 (1H, ddd, *J* = 7.8, 1.4, 0.5 Hz, CH_{Ar}), 7.50 (1H, td, *J* = 7.6, 1.5 Hz, CH_{Ar}), 7.50 (1H, d, *J* = 8.5 Hz, CH_{Ar}), 7.37 (1H, td, *J* = 7.6, 1.4 Hz, CH_{Ar}), 6.80 (1H, d, *J* = 2.6 Hz, CH_{Ar}), 6.75 (1H, dd, *J* = 8.5, 2.6 Hz, CH_{Ar}) 3.97 (3H, s, OCH_3), 3.84 (3H, s, $COOCH_3$), 2.57 (3H, s, CH_3); ^{13}C{1H} NMR ($CDCl_3$, 75 MHz, 25 °C): δ = 166.9 (C=O), 159.9 (C_{Ar}), 142.3 (C_{Ar}), 134.0 (CH_{Ar}), 133.7 (CH_{Ar}), 131.6 (CH_{Ar}), 131.4 (C_{Ar}), 130.4 (CH_{Ar}), 127.4 (CH_{Ar}), 124.3 (C_{Ar}), 115.4 (C_{Ar}), 115.1 (CH_{Ar}), 111.4 (CH_{Ar}), 93.7 (*C*≡C), 90.7 (C≡*C*), 55.3 (OCH_3), 52.2 (COO*C*H_3), 21.0 (CH_3); IR (CH_2Cl_2): ν = 2944, 1728, 1269 cm^{-1}; HRMS (ESI-TOF) m/z: $[M + H]^+$ Calcd for $C_{18}H_{17}O_3$ 281.1172; Found 281.1160.

Compound **2g**

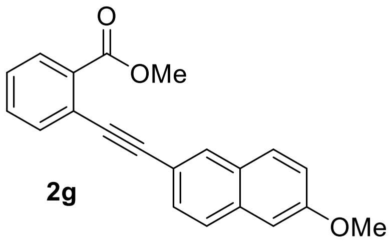

2g

Following the general procedure, starting from 88 mg of iodoester **9g**, 75 mg (85%) of compound **2g** were obtained as a yellow thick oil, after purification on column chromatography with gradient elution using hexanes/AcOEt (9:1 to 8:2) as solvent.

^{1}H NMR ($CDCl_3$, 300 MHz, 25 °C): δ = 8.05 (1H, s, CH_{Ar}), 8.01 (1H, dd, *J* = 7.9, 1.0 Hz, CH_{Ar}), 7.77 – 7.68 (3H, m, CH_{Ar}), 7.61 (1H, dd, *J* = 8.5, 1.6 Hz, CH_{Ar}), 7.52 (1H, td, *J* = 7.6, 1.4 Hz, CH_{Ar}), 7.40 (1H, td, *J* = 7.6, 1.4 Hz, CH_{Ar}), 7.18 (1H, dd, *J* = 8.9, 2.5 Hz, CH_{Ar}), 7.14 (1H, d, *J* = 2.5 Hz), 4.01 (3H, s, OCH_3), 3.94 (3H, s, $COOCH_3$); ^{13}C{1H} NMR ($CDCl_3$, 75 MHz, 25 °C): δ = 167.2 (C=O), 158.8 (C_{Ar}), 134.7 (C_{Ar}), 134.4 (CH_{Ar}), 132.2 (CH_{Ar}), 132.1 (C_{Ar}), 131.9 (CH_{Ar}), 130.9 (CH_{Ar}), 129.9 (CH_{Ar}), 129.5 (CH_{Ar}), 128.9 (C_{Ar}), 128.2 (CH_{Ar}), 127.3 (CH_{Ar}), 124.4 (C_{Ar}), 119.8 (CH_{Ar}), 118.6 (C_{Ar}), 106.2 (CH_{Ar}),

95.5 ($C{\equiv}C$), 88.4 ($C{\equiv}C$), 55.8 (OCH_3), 52.7 ($COOCH_3$); HRMS (ESI-TOF) m/z: $[M + H]^+$ Calcd for $C_{21}H_{17}O_3$ 317.1172; Found 317.1160.

Compound **2j**

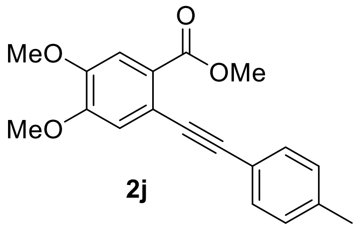

Following the general procedure, starting from 173 mg of iodoester **9j**, 88 mg (63%) of compound **2j** were obtained as a yellow thick oil, after purification on column chromatography using hexanes/Et_2O (3:1) as solvent.

^1H NMR ($CDCl_3$, 300 MHz, 25 °C): δ = 7.50 (1H, s, CH_{Ar}), 7.46 (2H, *AA'*XX', 2 x CH_{Ar}), 7.15 (2H, AA'*XX'*, 2 x CH_{Ar}), 7.06 (1H, s, CH_{Ar}), 3.94 (3H, s, OMe), 3.94 (3H, s, OMe), 3.93 (3H, s, CO_2Me), 2.36 (3H, s, Me); $^{13}C\{1H\}$ NMR ($CDCl_3$, 75 MHz, 25 °C): δ = 166.2 (C=O), 151.5 (C_{Ar}), 148.3 (C_{Ar}), 138.4 (C_{Ar}), 131.4 (2 x CH_{Ar}), 129.0 (2 x CH_{Ar}), 124.2 (C_{Ar}), 120.3 (C_{Ar}), 117.6 (C_{Ar}), 115.6 (CH_{Ar}), 112.8 (CH_{Ar}), 93.2 ($C{\equiv}C$), 87.9 ($C{\equiv}C$), 56.0 (OMe), 55.9 (OMe), 51.9 (CO_2Me), 21.4 (CH_3); HRMS (ESI-TOF) m/z: $[M + H]^+$ Calcd for $C_{19}H_{19}O_4$ 311.1278; Found 311.1274.

Compound **2k**

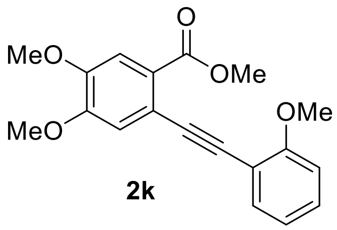

Following the general procedure, starting from 150 mg of iodoester **9k**, 95 mg (76%) of compound **2k** were obtained as a yellow thick oil, after purification on column chromatography using hexanes/AcOEt (3:1) as solvent.

^1H NMR ($CDCl_3$, 300 MHz, 25 °C): δ = 7.56 (1H, dd, *J* = 7.6, 1.8 Hz, CH_{Ar}), 7.52 (1H, s, CH_{Ar}), 7.32 (1H, ddd, *J* = 8.3, 7.6, 1.8 Hz, CH_{Ar}), 7.12 (1H, s, CH_{Ar}), 6.97 (1H, dd, *J* = 7.6, 1.1 Hz, CH_{Ar}), 6.92 (1H, d, *J* = 8.0 Hz, CH_{Ar}), 3.96 (3H, s, OCH_3), 3.96 (3H, s, OCH_3), 3.95 (3H, s, OCH_3), 3.94 (3H, s, $COOCH_3$);$^{13}C\{1H\}$ NMR ($CDCl_3$, 75 MHz, 25 °C): δ = 166.4 (C=O), 159.9 (C_{Ar}), 151.5 (C_{Ar}), 148.5 (C_{Ar}), 133.8 (CH_{Ar}), 129.8 (CH_{Ar}), 124.4 (C_{Ar}), 120.5 (CH_{Ar}), 117.8 (C_{Ar}), 115.9 (CH_{Ar}), 112.8 (CH_{Ar}), 112.7

(C_{Ar}), 110.7 (CH_{Ar}), 92.6 (*C*≡C), 89.5 (C≡*C*), 56.2 (OCH_3), 56.1 (OCH_3), 55.9 (OCH_3), 52.0 (COO*CH₃*); HRMS (ESI-TOF) m/z: $[M + H]^+$ Calcd for $C_{19}H_{19}O_5$ 327.1227; Found 327.1224.

Compound **2l**

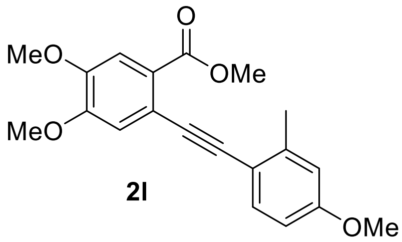

Following the general procedure, starting from 98 mg of iodoester **9l**, 75 mg (86%) of compound **2l** were obtained as a pale yellow thick oil, after purification on column chromatography using hexanes/AcOEt (3:1) as solvent.

^{1}H NMR ($CDCl_3$, 300 MHz, 25 °C): δ = 7.68 (1H, s, CH_{Ar}), 7.48 (1H, d, *J* = 8.5 Hz, CH_{Ar}), 7.06 (1H, s, CH_{Ar}), 6.79 (1H, d, *J* = 2.6 Hz, CH_{Ar}), 6.73 (1H, dd, *J* = 8.5, 2.6 Hz, CH_{Ar}), 3.97 (3H, s, OMe), 3.96 (3H, s, OMe), 3.94 (3H, s, OMe), 3.83 (3H, s, CO_2Me), 2.56 (3H, s, CH_3); ^{13}C{1H} NMR ($CDCl_3$, 75 MHz, 25 °C): δ = 165.4 (C=O), 159.6 (C_{Ar}), 151.5 (C_{Ar}), 148.3 (C_{Ar}), 142.1 (C_{Ar}), 133.5 (CH_{Ar}), 123.9 (C_{Ar}), 118.1 (C_{Ar}), 115.7 (CH_{Ar}), 115.6 (C_{Ar}), 115.0 (CH_{Ar}), 112.8 (CH_{Ar}), 111.3 (CH_{Ar}), 92.3 (*C*≡C), 90.9 (C≡*C*), 56.1 (OMe), 56.1 (OMe), 55.2 (OMe), 52.1 (CO_2Me), 21.0 (CH_3); HRMS (ESI-TOF) m/z: $[M+H]^+$ Calcd for $C_{20}H_{21}O_5$ 341.1384; Found 341.1380.

Compound **2m**

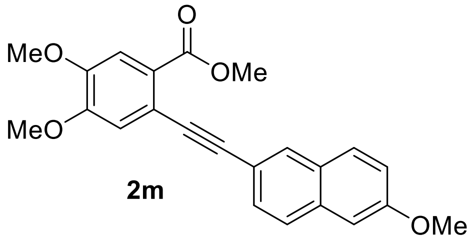

Following the general procedure, starting from 63 mg of iodoester **9m**, 42 mg (69%) of compound **2m** were obtained as a yellow thick oil, after purification on column chromatography using hexanes/Et_2O (3:1) as solvent.

^{1}H NMR ($CDCl_3$, 300 MHz, 25 °C): δ = 8.03 (1H, br s, CH_{Ar}) 7.73 (2H, dd, *J* = 8.8, 4.7 Hz, CH_{Ar}), 7.61 (1H, dd, *J* = 8.5, 1.6 Hz, CH_{Ar}), 7.54 (1H, s, CH_{Ar}), 7.19-7.13 (3H, m, CH_{Ar}), 3.99 (3H, s, OCH_3), 3.99 (3H, s, OCH_3), 3.97 (3H, s, OCH_3), 3.94 (3H, s, $COOCH_3$); ^{13}C{1H} NMR ($CDCl_3$, 75 MHz, 25 °C): δ = 166.3 (C=O), 158.4 (C_{Ar}), 151.6 (C_{Ar}), 148.5 (C_{Ar}), 134.2 (C_{Ar}), 131.3 (CH_{Ar}), 129.4 (CH_{Ar}), 129.0 (CH_{Ar}), 128.5 (C_{Ar}), 126.8 (CH_{Ar}), 124.4 (C_{Ar}), 119.4 (CH_{Ar}), 118.4 (C_{Ar}), 117.8 (C_{Ar}), 115.7 (CH_{Ar}),

113.0 (CH_{Ar}), 105.8 (CH_{Ar}), 93.7 (*C*≡C), 88.3 (C≡*C*), 56.2 (OCH_3), 56.1 (OCH_3), 55.4 (OCH_3), 52.1 ($COOCH_3$); HRMS (ESI-TOF) m/z: $[M+H]^+$ Calcd for $C_{23}H_{21}O_5$ 377.1384; Found 377.1384.

Compound **2n**

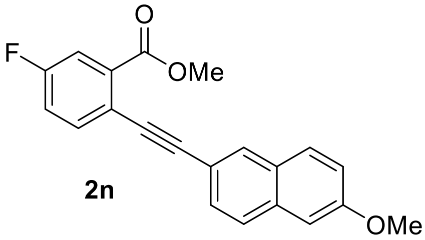

2n

Following the general procedure, starting from 94 mg of iodoester **9n**, 83 mg (88%) of compound **2n** were obtained as a colorless thick oil, after purification on column chromatography using hexanes/AcOEt (9:1) as solvent.

^{1}H NMR ($CDCl_3$, 300 MHz, 25 °C): δ = 8.02 (1H, br s, CH_{Ar}), 7.76-7.65 (4H, m, CH_{Ar}), 7.59 (1H, dd, *J* = 8.7, 2.5 Hz, CH_{Ar}), 7.27-7.23 (1H, m, CH_{Ar}), 7.18 (1H, dd, *J* = 8.5, 1.7 Hz, CH_{Ar}), 7.13 (1H, d, *J* = 2.6 Hz, CH_{Ar}), 4.01 (3H, s, OMe), 3.94 (3H, s, CO_2Me); ^{13}C{1H} NMR ($CDCl_3$, 75 MHz, 25 °C): δ = 165.6 (d, $^4J_{C\text{-}F}$ = 2.7 Hz, C=O), 161.6 (d, $^1J_{C\text{-}F}$ = 251.0 Hz, $C_{Ar}F$), 158.5 (C_{Ar}), 135.8 (d, $^3J_{C\text{-}F}$ = 7.8 Hz, CH_{Ar}), 134.4 (C_{Ar}), 133.6 (d, $^3J_{C\text{-}F}$ = 7.3 Hz, C_{Ar}), 131.5 (CH_{Ar}), 129.4 (CH_{Ar}), 129.0 (CH_{Ar}), 128.5 (C_{Ar}), 126.9 (CH_{Ar}), 120.3 (d, $^4J_{C\text{-}F}$ = 3.5 Hz, C_{Ar}), 119.5 (CH_{Ar}), 119.3 (d, $^2J_{C\text{-}F}$ = 22.1 Hz, CH_{Ar}), 118.0 (C_{Ar}), 117.6 (d, $^2J_{C\text{-}F}$ = 24.2 Hz, CH_{Ar}), 105.9 (CH_{Ar}), 94.8 (d, $^5J_{C\text{-}F}$ = 1.8 Hz, *C*≡C), 86.7 (C≡*C*), 55.4 (OMe), 52.5 (CO_2Me); ^{19}F NMR ($CDCl_3$, 282 MHz, 25 °C): δ = -110.5 (1F, s, F); HRMS (ESI-TOF) m/z: $[M+H]^+$ Calcd for $C_{21}H_{16}FO_3$ 335.1078; Found 335.1084.

Compound **2p**

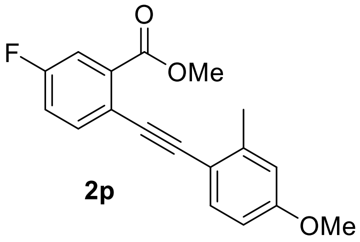

2p

Following the general procedure, starting from 62 mg of iodoester **9p**, 94 mg (75%) of compound **2p** were obtained as a yellow thick oil, after purification on column chromatography using hexanes/AcOEt (6:1) as solvent.

^{1}H NMR ($CDCl_3$, 300 MHz, 25 °C): δ = 7.73 – 7.58 (2H, m, CH_{Ar}), 7.48 (1H, d, *J* = 8.4 Hz, CH_{Ar}), 7.22 (1H, ddd, *J* = 8.6, 7.8, 2.8 Hz, CH_{Ar}), 6.80 (1H, d, *J* = 2.6 Hz, CH_{Ar}), 6.75 (1H, dd, *J* = 8.4, 2.6 Hz, CH_{Ar}), 3.98 (3H, s, OCH_3), 3.84 (3H, s, $COOCH_3$), 2.56 (3H, s, CH_3); ^{13}C{1H} NMR ($CDCl_3$, 75 MHz,

25 °C): δ = 165.7 (d, $^{4}J_{C\text{-}F}$ = 2.7 Hz, C=O), 161.3 (d, $^{1}J_{C\text{-}F}$ = 249.9 Hz, $C_{Ar}F$), 159.9 (C_{Ar}), 142.3 (C_{Ar}) 135.8 (d, $^{3}J_{C\text{-}F}$ = 7.8 Hz, CH_{Ar}), 133.6 (CH_{Ar}), 133.2 (d, $^{3}J_{C\text{-}F}$ = 7.8 Hz, C_{Ar}), 120.6 (d, $^{4}J_{C\text{-}F}$ = 3.6 Hz, C_{Ar}), 119.2 (d, $^{2}J_{C\text{-}F}$ = 22.1 Hz, CH_{Ar}), 117.4 (d, $^{2}J_{C\text{-}F}$ = 23.9 Hz, CH_{Ar}), 115.2 (C_{Ar}), 115.1 (CH_{Ar}), 111.4 (CH_{Ar}), 93.4 (d, $^{5}J_{C\text{-}F}$ = 1.6 Hz, , *C*≡C), 89.7 (C≡*C*), 55.3 (OMe), 52.5 (CO_2Me), 21.0 (Me); ^{19}F NMR ($CDCl_3$, 282 MHz, 25 °C): δ = -111.5 (1F, s, F); IR (CH_2Cl_2): ν = 2952, 1734, 1239 cm^{-1}; HRMS (ESI-TOF) m/z: $[M+H]^+$ Calcd for $C_{18}H_{16}FO_3$ 299.1078; Found 299.1070.

Compound **2r**

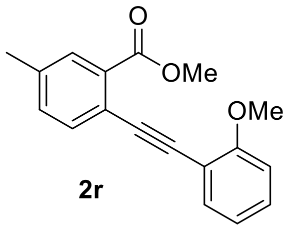

2r

Following the general procedure, starting from 130 mg of iodoester **9r**, 107 mg (97%) of compound **2r** were obtained as a pale yellow thick oil, after purification on column chromatography with gradient elution using hexanes/AcOEt (9:1 to 7:1) as solvent.

^{1}H NMR ($CDCl_3$, 300 MHz, 25 °C): δ = 7.79 (1H, dt, *J* = 2.0, 0.6 Hz, CH_{Ar}), 7.58 (1H, d, *J* = 7.9 Hz, CH_{Ar}), 7.55 (1H, ddd, *J* = 7.5, 1.7, 0.4 Hz, CH_{Ar}), 7.34 – 7.28 (2H, m, CH_{Ar}), 6.96 (1H, dd, *J* = 7.5, 1.1 Hz, CH_{Ar}), 6.93–6.89 (2H, m, CH_{Ar}), 3.97 (3H, s, OCH_3), 3.93 (3H, s, $COOCH_3$), 2.40 (3H, s, CH_3); ^{13}C{1H} NMR ($CDCl_3$, 75 MHz, 25 °C): δ = 167.2 (C=O), 160.1 (C_{Ar}), 138.1 (C_{Ar}), 134.1 (CH_{Ar}), 133.9 (CH_{Ar}), 132.5 (CH_{Ar}), 131.6 (C_{Ar}), 131.0 (CH_{Ar}), 129.9 (CH_{Ar}), 121.1 (C_{Ar}), 120.6 (CH_{Ar}), 112.6 (C_{Ar}), 110.8 (CH_{Ar}), 92.5 (*C*≡C), 90.0 (C≡*C*), 55.9 (OCH_3), 52.1 (COO*C*H_3), 21.3 (CH_3); HRMS (ESI-TOF) m/z: $[M + H]^+$ Calcd for $C_{18}H_{17}O_3$ 281.1172; Found 281.1164.

Compound **2s-*p*-CF₃**

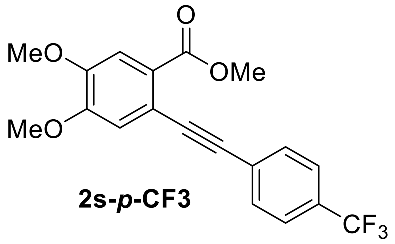

2s-*p*-CF3

Following the general procedure, starting from 114 mg of iodoester **9s-*p*-CF_3**, 63 mg (59%) of compound **2s-*p*-CF_3** were obtained as a yellow thick oil, after purification on column chromatography using hexanes/Et_2O (3:1) as solvent.

^{1}H NMR ($CDCl_3$, 300 MHz, 25 °C): δ = 7.68 (2H, AA'*XX'*, 2 x CH_{Ar}), 7.61 (2H, AA'*XX'*, 2 x CH_{Ar}), 7.53 (1H, s, CH_{Ar}), 7.09 (1H, s, CH_{Ar}), 3.97 (3H, s, OMe), 3.97 (3H, s, OMe), 3.96 (3H, s, CO_2Me); ^{13}C{1H} NMR ($CDCl_3$, 75 MHz, 25 °C): δ = 166.0 (C=O), 151.7 (C_{Ar}), 149.0 (C_{Ar}), 131.8 (2 x CH_{Ar}), 129.9 (q, J_{C-F} = 33.6 Hz, C_{Ar}-CF_3), 127.4 (C_{Ar}), 125.3 (q, J_{C-F} = 3.9 Hz, 2 x CH_{Ar}), 124.8 (C_{Ar}), 123.9 (q, J_{C-F} = 272.4 Hz, CF_3), 116.7 (C_{Ar}), 115.8 (CH_{Ar}), 113.0 (CH_{Ar}), 91.5 (*C*≡C), 91.0 (C≡*C*), 56.2 (OMe), 56.1 (OMe), 52.1 (CO_2Me); ^{19}F NMR ($CDCl_3$, 282 MHz, 25 °C): δ = -62.8 (3F, s, CF_3); HRMS (ESI-TOF) m/z: $[M+H]^+$ Calcd for $C_{19}H_{16}F_3O_4$ 365.0995; Found 365.0984.

Compound **2s-*o*-CF₃**

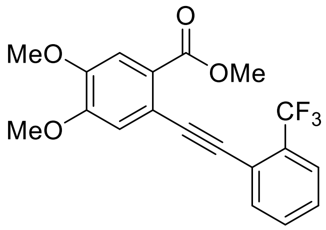

Following the general procedure, starting from 134 mg of iodoester **9s-*o*-CF₃**, 126 mg (99%) of compound **2s-*o*-CF₃** were obtained as a yellow oil, after purification on column chromatography using hexanes/Et_2O (3:1) as solvent.

^{1}H NMR ($CDCl_3$, 300 MHz, 25 °C): δ = 7.75 (1H, d, *J* = 7.7 Hz, H_{Ar}), 7.69 (1H, d, *J* = 7.8 Hz, H_{Ar}), 7.54 (1H, t, *J* = 7.5 Hz, H_{Ar}), 7.53 (1H, s, H_{Ar}), 7.43 (1H, t, *J* = 7.7 Hz, H_{Ar}), 7.09 (s, 1H), 3.97 (3H, s, OCH_3), 3.96 (3H, s, OCH_3), 3.96 (3H, s, CH_3); ^{13}C{1H} NMR ($CDCl_3$, 75 MHz, 25 °C): δ = 166.3 (C=O); 151.8 (C_{Ar}), 149.2 (C_{Ar}), 134.2 (CH_{Ar}), 131.6 (1C, d, $^4J_{CHAr-CF3}$ = 1.1 Hz, CH_{Ar}), 131.3 (1C, d, $^2J_{CHAr-CF3}$ = 30.4 Hz, C_{Ar}), 128.1 (CH_{Ar}), 126.0 (1C, q, $^3J_{CHAr-CF3}$ = 5.0 Hz, CH_{Ar}), 124.7 (C_{Ar}), 123.8 (1C, q, $^1J_{C-F}$ = 273.5 Hz, CF_3), 122.0 (1C, d, $^4J_{CHAr-CF3}$ = 2.5 Hz, C_{Ar}), 117.0 (C_{Ar}), 116.1 (CH_{Ar}), 113.0 (CH_{Ar}), 94.2 (*C*≡C), 89.0 (C≡*C*), 56.2 (2C, s, 2 OMe), 52.3 (OMe); ^{19}F NMR ($CDCl_3$, 282 MHz, 25 °C): δ = -62.1 (3F, s, CF_3); HRMS (ESI-TOF) m/z: $[M+H]^+$ Calcd for $C_{19}H_{16}F_3O_4$ 365.0995; Found 365.0981.

1.2. 2-Ethynylbenzamides **5**

2-Ethynylbenzamides **5** were prepared according to a modification of the standard Sonogashira reaction conditions. Iodoamides **10** (1.1 equiv.), CuI (5 mol%) and $PdCl_2(PPh_3)_2$ (5 mol%) were placed in an oven-dried round bottom flask, and purged under Ar atmosphere. Then, TEA (3.8 mL/mmol) and DMF (7-12 drops) were added and the mixture was stirred at room temperature for 5 minutes. After that time, the corresponding alkyne (1 equiv.) was added in one portion and the reaction was stirred at 85 °C under microwave irradiation until disappearance of the starting material (TLC). The crude reaction mixture was filtered through a celite pad, and the solvent was

evaporated under reduced pressure. The mixture was purified on column chromatography using mixtures of hexanes and AcOEt.

Table S3

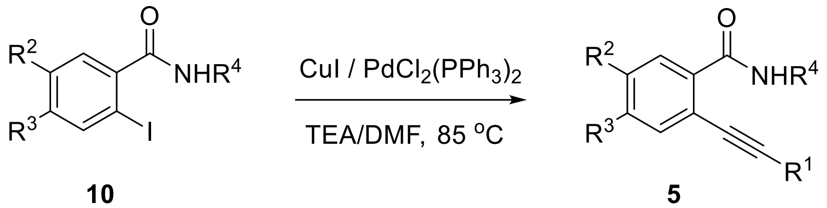

10 **5**

Entry	10	R^4	R^2	R^3	R^1	5 (%)
1	**10a**	Ph	H	H	4-MeC_6H_4	**5a** (68)
2	**10b**	Ph	H	H	2-Me-4-OMeC_6H_3	**5b** (66)
3	**10c**	4-OMe-C_6H_4	H	H	4-MeC_6H_4	**5c** (84)
4	**10d**	4-OMe-C_6H_4	H	H	4-OMeC_6H_4	**5d** (70)
5	**10e**	4-OMe-C_6H_4	H	H	2-Me-4-OMeC_6H_3	**5e** (79)

Compounds **5a**[6], **5c**, **5d**[7] were prepared according to the general procedure, exhibiting analytical data in accordance to previous reports.

Compound **5b**

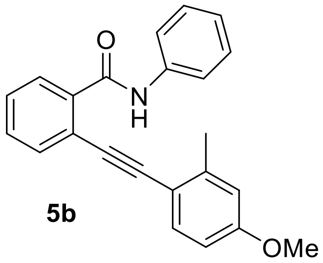

5b

Following the general procedure, starting from 80 mg of iodoester **10b**, 55 mg (66%) of compound **5b** were obtained as a yellow gum, after purification on column chromatography using hexanes/AcOEt (3:1) as solvent.

^{1}H NMR ($CDCl_3$, 300 MHz, 25 °C): δ = 9.27 (1H, br s, NH), 8.14 (1H, m, CH_{Ar}), 7.64 (3H, m, CH_{Ar}), 7.48 (2H, m, CH_{Ar}), 7.35 (2H, m, CH_{Ar}), 7.13 (1H, tt, *J* = 7.3, 1.2 Hz, CH_{Ar}), 6.77 (1H, d, *J* = 2.6 Hz, CHAr), 6.70 (1H, dd, *J* = 8.4, 2.6 Hz, CH_{Ar}), 3.82 (3H, s, OMe), 2.42 (3H, s, Me); ^{13}C{1H} NMR ($CDCl_3$,

75 MHz, 25 °C): δ = 164.5 (C=O), 160.4 (C_{Ar}), 142.4 (C_{Ar}), 138.0 (C_{Ar}), 135.4 (C_{Ar}), 133.5 (CH_{Ar}), 133.4 (CH_{Ar}), 130.9 (CH_{Ar}), 130.3 (CH_{Ar}), 129.1 (2 CH_{Ar}), 128.7 (CH_{Ar}), 124.4 (CH_{Ar}), 120.2 (C_{Ar}), 120.1 (2 CH_{Ar}), 115.4 (CH_{Ar}), 113.8 (C_{Ar}), 111.6 (CH_{Ar}), 96.0 (*C*≡C), 89.7 (C≡*C*), 55.3 (OMe), 20.9 (Me); HRMS (ESI-TOF) m/z: $[M]^+$ Calcd for $C_{23}H_{19}NO_2$ 341.1416; Found 341.1414.

Compound **5e**

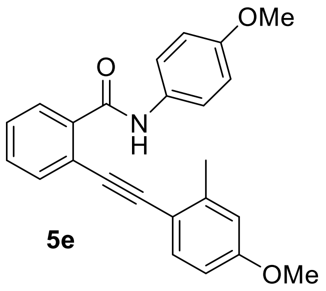

5e

Following the general procedure, starting from 80 mg of iodoester **10e**, 66 mg (79%) of compound **5e** were obtained as a yellow gum, after purification on column chromatography using hexanes/AcOEt (3:1) as solvent.

^1H NMR ($CDCl_3$, 300 MHz, 25 °C): δ = 9.18 (1H, br s, NH), 8.12 (1H, m, CH_{Ar}), 7.62 (1H, m, CH_{Ar}), 7.54 (2H, d, *J* = 9.0 Hz, CH_{Ar}), 7.45 (2H, m, CH_{Ar}), 7.37 (1H, d, *J* = 8.5 Hz, CH_{Ar}), 6.86 (2H, d, *J* = 9.0 Hz, CHAr), 6.77 (1H, d, *J* = 2.6 Hz, CH_{Ar}), 6.71 (1H, dd, *J* = 8.5, 2.6 Hz, CH_{Ar}), 3.81 (3H, s, OMe), 3.79 (2H, OMe), 2.41 (3H, s, Me); $^{13}C\{1H\}$ NMR ($CDCl_3$, 75 MHz, 25 °C): δ = 164.3 (C=O), 160.5 (C_{Ar}), 156.5 (CAr), 142.4 (C_{Ar}), 135.5 (C_{Ar}), 133.5 (CH_{Ar}), 133.4 (CH_{Ar}), 131.2 (C_{Ar}), 130.7 (CH_{Ar}), 130.2 (CH_{Ar}), 128.7 (CH_{Ar}), 121.8 (2 CH_{Ar}), 120.2 (C_{Ar}), 115.4 (CH_{Ar}), 114.2 (2 CH_{Ar}), 113.9 (C_{Ar}), 111.6 (CH_{Ar}), 95.8 (*C*≡C), 89.8 (C≡*C*), 55.5 (OMe), 55.3 (OMe), 21.0 (Me); IR (CH_2Cl_2): ν = 2972, 1700, 1238 cm^{-1}; HRMS (ESI-TOF) m/z: $[M]^+$ Calcd for $C_{24}H_{21}NO_3$ 371.1521; Found 371.1517.

1.3. 2-Ethynylbenzenesulfonamides **6**

2-Ethynylbenzenesulfonamides **6** were prepared according to a modification of the standard Sonogashira reaction conditions. Iodosulfonamides **11** (1.1 equiv.), CuI (5 mol%) and $PdCl_2(PPh_3)_2$ (5 mol%) were placed in an oven-dried round bottom flask, and purged under Ar atmosphere. Then, TEA (3.8 mL/mmol) and DMF (7-12 drops) were added and the mixture was stirred at room temperature for 5 minutes. After that time, the corresponding alkyne (1 equiv.) was added in one portion and the reaction was stirred at 100 °C under microwave irradiation until disappearance of the starting material (TLC). The crude reaction mixture was filtered through a celite pad, and the solvent was evaporated under reduced pressure. The mixture was purified on column chromatography using mixtures of hexanes and AcOEt.

Table S4

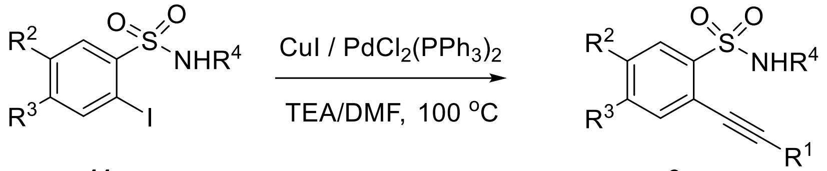

11 6

Entry	11	R^1	R^2	R^3	R^4	6 (%)
1	**11a**	4-Me-C_6H_4	H	H	4-OMeC_6H_4	**6a** (56)
2	**11b**	4-OMe-C_6H_4	H	H	4-OMeC_6H_4	**6b** (68)

Compounds **6a** and **6b** were prepared according to the general procedure, exhibiting analytical data in accordance to previous reports. [8]

2. 1,1-Bis(triflyl)ethene-assisted heterocyclization reactions

2.1. Oxycyclization reactions: Synthesis of isocoumarins **3** from 2-ethynylbenzoates **2**

To a solution of the appropriate 2-ethynylbenzoate **2** (1 equiv.) in MeCN (20 mL/mmol) was added zwitterion **1** (1 equiv.) in one portion. The reaction mixture was stirred at the indicated temperature (from 40 to 100 °C) until disappearance of starting material (TLC). After that time, the solvent was evaporated and the crude mixture was purified on column chromatography, yielding the indicated isocoumarins as sodium salts.

Table S5

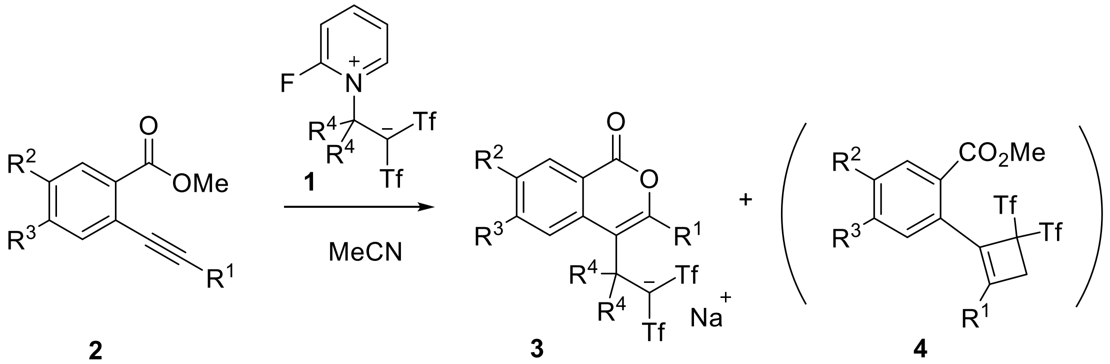

2 3 4

Entry	2	R^1	R^2	R^3	R^4	T (°C)	t (h)	3/4 (%)
1	**2a**	4-OMe-C_6H_4	H	H	H	40	2.5	**3a/4a** (95/0)
2	**2b**	4-Me-C_6H_4	H	H	H	40	3	**3b/4b** (68/0)
3	**2c**	Ph	H	H	H	40	6	**3c/4c** (22/0)

4	**2d**	4-F-C_6H_4	H	H	H	100	8	**3d/4d** (9/nd)[a]
5	**2e**	2-OMe-C_6H_4	H	H	H	40	3.5	**3e/4e** (79/0)
6	**2f**	2-Me-4-OMe-C_6H_3	H	H	H	40	2.5	**3f/4f** (98/0)
7	**2g**	6-MeO-naphthyl	H	H	H	40	3	**3g/4g** (72/0)
8	**2h**	Cyclopentyl	H	H	H	40	48	**3h/4h** (31/0)[b]
9	**2i**	4-OMe-C_6H_4	OMe	OMe	H	40	3	**3i/4i** (71/0)
10	**2j**	4-Me-C_6H_4	OMe	OMe	H	40	4	**3j/4j** (80/0)
11	**2k**	2-OMe-C_6H_4	OMe	OMe	H	40	4	**3k/4k** (74/0)
12	**2l**	2-Me-4-OMe-C_6H_3	OMe	OMe	H	40	2.5	**3l/4l** (77/0)
13	**2m**	6- MeO-naphthyl	OMe	OMe	H	40	6	**3m/4m** (63/0)
14	**2n**	6- MeO-naphthyl	F	H	H	40	2	**3n/4n** (51/0)
15	**2o**	4-OMe-C_6H_4	Me	H	H	40	4	**3o/4o** (78/0)
16	**2p**	2-Me-4-OMe-C_6H_3	F	H	H	40	3	**3p/4p** (68/0)
17	**2q**	4-Me-C_6H_4	Me	H	H	40	3	**3q/4q** (88/0)
18	**2r**	2-OMe-C_6H_4	Me	H	H	40	4	**3r/4r** (77/0)
19	**2s-*p*-CF_3**	4-CF_3-C_6H_4	OMe	OMe	H	40	4	**3s-*p*-CF_3/4s-*p*-CF_3** (0/21)
20	**2s-*o*-CF_3**	2-CF_3-C_6H_4	OMe	OMe	H	40	4	**3s-*o*-CF_3/4s-*o*-CF_3** (0/63)
21	**2i**	4-OMe-C_6H_4	OMe	OMe	D	40	3.5	**[D]-3i/4i** (86/0)

[a]Compound was isolated from a complex reaction mixture. Reaction was carried out at 100 °C.

[b]Compound was partially isolated from complex reaction mixtures, lacking of purity to consider full characterization. Yield estimated by NMR.

Compound **3a**

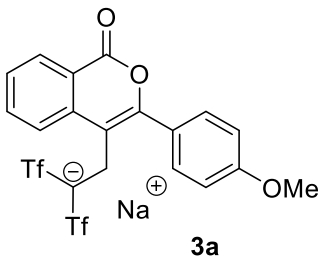

3a

Following the general procedure, starting from 20 mg of 2-ethynylbenzoate **2a**, 39 mg (95%) of compound **3a** were obtained as a colorless thick oil, after purification on column chromatography using hexanes/AcOEt (2:3) as solvent.

^{1}H NMR (d_6-acetone, 700 MHz, 25 °C): δ = 8.59 (d, 2H, *J* = 8.2 Hz, CH_{Ar}), 8.17 (dd, 1H, *J* = 7.9, 1.1 Hz, CH_{Ar}), 7.75 (m, 1H, CH_{Ar}), 7.49 (m, 2H, 2CH_{Ar}), 6.96 (m, 2H, 2CH_{Ar}), 3.89 (s, 2H, CH_2), 3.81 (s, 2H, OCH_3). ^{13}C{1H} NMR (d_6-acetone, 75 MHz, 25 °C): δ = 162.8 (C_{Ar}), 161.0 (C=O), 154.1 (*C*=C), 139.5 (C_{Ar}), 134.6 (CH_{Ar}), 132.4 (2CH_{Ar}), 129.3 (CH_{Ar}), 127.7 (CH_{Ar}), 137.3 (C_{Ar}), 126.3 (CH_{Ar}), 121.9

(2C, q, $J_{C\text{-}F}$ = 329.1 Hz, $2CF_3$), 121.6 (C_{Ar}), 113.8 ($2CH_{Ar}$), 111.9 (C=*C*), 64.2 (C^-Tf_2), 55.4 (OCH_3), 28.7 ($CH_2C^-Tf_2$); ^{19}F NMR (d_6-acetone, 282 MHz, 25 °C): δ = -79.7 (s, 6F, $2CF_3$); IR (CH_2Cl_2): ν = 2975, 1701, 1180 cm^{-1}; HRMS (ESI-TOF) m/z: $[M + H]^-$ Calcd for $[C_{20}H_{14}F_6O_7S_2]^-$ 544.0085; Found 544.0086.

Compound **3b**

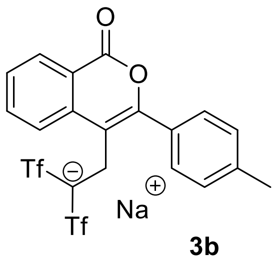

3b

Following the general procedure, starting from 32 mg of 2-ethynylbenzoate **2b**, 48 mg (68%) of compound **3b** were obtained as a colorless thick oil, after purification on column chromatography with gradient elution using hexanes/AcOEt (1:1 to 1:3) as solvent.

^{1}H NMR (d_6-acetone, 300 MHz, 25 °C): δ = 8.66 (1H, d, *J* = 8.2 Hz, CH_{Ar}), 8.23 (1H, dd, *J* = 7.9, 1.5 Hz, CH_{Ar}), 7.81 (1H, ddd, *J* = 8.4, 7.3, 1.5 Hz, CH_{Ar}), 7.55 (1H, ddd, *J* = 8.1, 7.3, 1.1 Hz, CH_{Ar}), 7.49 (2H, d, *J* = 8.2 Hz, CH_{Ar}), 7.27 (2H, d, *J* = 7.9 Hz, CH_{Ar}), 3.94 (2H, s, CH_2), 2.39 (3H, s, CH_3); ^{13}C{1H} NMR (d_6-acetone, 75 MHz, 25 °C): δ = 162.0 (C=O), 153.5 (*C*=C), 138.9 (C_{Ar}), 138.8 (C_{Ar}), 133.9 (CH_{Ar}), 131.5 (C_{Ar}), 130.2 ($2CH_{Ar}$), 128.6 (CH_{Ar}), 128.4 ($2CH_{Ar}$), 127.1 (CH_{Ar}), 125.7 (CH_{Ar}), 121,3 (2C, q, $J_{C\text{-}F}$ = 328.9 Hz, $2CF_3$), 120.9 (C_{Ar}), 111.5 (C=*C*), 63.6 (CH_{Ar}), 63.6-63.4 (m, $^-CTf_2$), 26.3 (CH_2), 20.5 (CH_3); ^{19}F NMR (d_6-acetone, 282 MHz, 25 °C): δ = -79.8 (s, 6F, $2CF_3$); HRMS (ESI-TOF) m/z: $[M]^-$ Calcd for $[C_{20}H_{13}F_6O_6S_2]^-$ 527.0063; Found 527.0067.

Compound **3c**

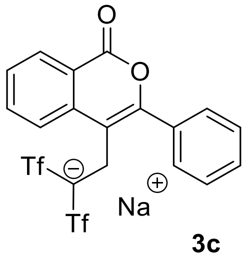

3c

Following the general procedure, starting from 30 mg of 2-ethynylbenzoate **2c**, 30 mg (44%) of compound **3c** were obtained as a colorless thick oil, after purification on column chromatography using hexanes/AcOEt (3:7) as solvent.

^{1}H NMR (d_6-acetone, 300 MHz, 25 °C): δ = 8.69 (1H, d, J = 8.0 Hz, CH_{Ar}), 8.24 (1H, dd, J = 7.9, 0.9 Hz, CH_{Ar}), 7.82 (1H, t, J = 7.3 Hz, CH_{Ar}), 7.59 (3H, m, CH_{Ar}), 7.46 (3H, m, CH_{Ar}), 3.96 (2H, s, CH_2); ^{13}C{1H} NMR (d_6-acetone, 75 MHz, 25 °C): δ = 161.9 (C=O), 153.3 (*C*=C), 138.7 (C_{Ar}), 134.4 (C_{Ar}), 133.9 (CH_{Ar}), 130.3 (2 CH_{Ar}), 128.9 (CH_{Ar}), 128.6 (CH_{Ar}), 127.7 (2 CH_{Ar}), 127.2 (CH_{Ar}), 125.7 (CH_{Ar}), 121.3 (q, J_{C-F} = 327.8 Hz, 2 x SO_2CF_3), 120.9 (C_{Ar}), 111.7 (C=*C*), 63.6 (C^-Tf_2), 26.3 ($CH_2C^-Tf_2$); ^{19}F NMR (d_6-acetone, 282 MHz, 25 °C): δ = -79.8 (s, 6F, $2CF_3$); HRMS (ESI-TOF) m/z: $[M]^-$ Calcd for $[C_{19}H_{11}F_6O_6S_2]^-$ 512.9901; Found 512.9909.

Compound **3d**

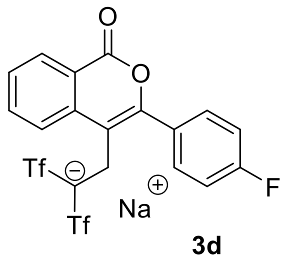

3d

Following a modification of the general procedure, starting from 25 mg of 2-ethynylbenzoate **2d**, and after heating the reaction mixture at 100 °C during 8h under mw irradiation, 5 mg (9%) of compound **3d** were obtained as a colorless thick oil, after purification on column chromatography using hexanes/AcOEt (3:7) as solvent.

^{1}H NMR (d_6-acetone, 700 MHz, 25 °C): δ = 8.69 (1H, m, CH_{Ar}), 8.24 (1H, ddd, J = 7.9, 1.4, 0.6 Hz, CH_{Ar}), 7.83 (1H, ddd, J = 8.3, 7.2, 1.5 Hz, CH_{Ar}), 7.67 (2H, dd, J = 8.8, 5.4 Hz, CH_{Ar}), 7.58 (1H, ddd, J = 8.1, 7.2, 1.1 Hz, CH_{Ar}), 7.25 (2H, t, J = 8.8 Hz, CH_{Ar}), 3.89 (2H, s, CH_2); ^{13}C{1H} NMR (d_6-acetone, 75 MHz, 25 °C): δ = 162.9 (1C, d, J_{C-F} = 246.5, C_{Ar}-F), 161.7 (C=O), 152.3 (*C*=C), 138.6 (C_{Ar}), 134.0 (CH_{Ar}), 132.5 (2C, d, J_{C-F} = 8.8 Hz, $2CH_{Ar}$), 130.8 (1C, d, J_{C-F} = 2.8 Hz, C_{Ar}), 128.6 (CH_{Ar}), 127.3 (CH_{Ar}), 125.8 (CH_{Ar}), 121.2 (2C, q, J_{C-F} = 327.3 Hz, 2 x SO_2CF_3), 121.0 (C_{Ar}), 114.6 (2C, d, J_{C-F} = 21.9 Hz, CH_{Ar}), 111.9 (C=*C*), 78.2 (C^-Tf_2), 26.3 ($CH_2C^-Tf_2$); HRMS (ESI-TOF) m/z: $[M]^-$ Calcd for $[C_{19}H_{10}F_7O_6S_2]^-$ 530.9807; Found 530.9809.

Compound **3e**

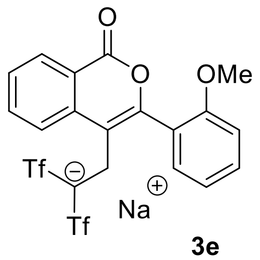

3e

Following the general procedure, starting from 42 mg of 2-ethynylbenzoate **2e**, 33 mg (79%) of compound **3e** were obtained as a colorless thick oil, after purification on column chromatography with gradient elution using hexanes/AcOEt (1:1 to 3:7) as solvent.

^{1}H NMR (d_6-acetone, 300 MHz, 25 °C): δ = 8.70 (1H, dt, *J* = 8.2, 0.9 Hz, CH_{Ar}), 8.23 (1H, dd, *J* = 8.0, 1.5 Hz, CH_{Ar}), 7.80 (1H, ddd, *J* = 8.4, 7.2, 1.5 Hz, CH_{Ar}), 7.61 – 7.49 (2H, m, CH_{Ar}), 7.43 (1H, ddd, *J* = 8.4, 7.4, 1.8 Hz, CH_{Ar}), 7.13 – 6.98 (2H, m, CH_{Ar}), 3.83 (1H, d, *J* = 15.5 Hz, CH_2), 3.80 (3H, s, OCH_3), 3.55 (1H, d, *J* = 15.5 Hz, CH_2); ^{13}C{1H} NMR (d_6-acetone, 75 MHz, 25 °C): δ = 163.3 (C=O), 158.3 (C_{Ar}), 150.3 (*C*=C), 139.5 (C_{Ar}), 134.7 (CH_{Ar}), 133.3 (CH_{Ar}), 131.7 (CH_{Ar}), 129.5 (CH_{Ar}), 128.0 (CH_{Ar}), 126.5 (CH_{Ar}), 123.9 (C_{Ar}), 122.2 (2C, q, J_{C-F} = 329.3 Hz, 2 x SO_2CF_3), 121.9 (C_{Ar}), 120.8 (CH_{Ar}), 114.2 (C=*C*), 111.8 (CH_{Ar}), 64.2-64.0 (m, $^-CTf_2$), 55.9 ($OfCH_3$), 27.6 (CH_2); ^{19}F NMR (d_6-acetone, 282 MHz, 25 °C): δ = -79.7 (s, 6F, $2CF_3$); ^{23}Na-RMN (d_6-acetone, 132 MHz, 25 °C): δ = -8.4 (s, 1Na, Na^+); IR (CH_2Cl_2): ν = 2974, 1699, 1178 cm^{-1}; HRMS (ESI-TOF) m/z: $[M]^-$ Calcd for $[C_{20}H_{13}F_6O_7S_2]^-$ 543.0012; Found 543.0014.

Compound **3f**

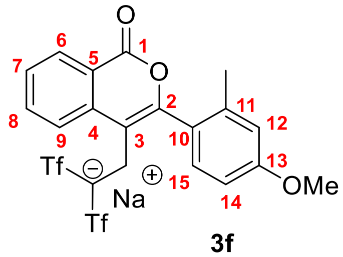

3f

Following the general procedure, starting from 21 mg of 2-ethynylbenzoate **3f**, 43 mg (98%) of compound **3f** were obtained as a colorless thick oil, after purification on column chromatography with gradient elution using hexanes/AcOEt (1:1 to 3:7) as solvent.

^{1}H NMR (d_6-acetone, 300 MHz, 25 °C): δ = 8.70 (1H, dt, *J* = 8.2, 0.9 Hz, CH_{Ar}, H-9), 8.22 (1H, ddd, *J* = 8.0, 1.5, 0.6 Hz, CH_{Ar}, H-6), 7.79 (1H, ddd, *J* = 8.2, 7.2, 1.5 Hz, CH_{Ar}, H-8), 7.54 (1H, ddd, *J* = 8.0, 7.2, 1.1 Hz, CH_{Ar}, H-7), 7.35 (1H, d, *J* = 8.4 Hz, CH_{Ar}, H-15), 6.86 (1H, d, *J* = 2.7 Hz, CH_{Ar}, H-12), 6.80 (1H, dd, *J* = 8.4, 2.7 Hz, CH_{Ar}, H-14), 3.83 (4H, s, OCH_3; CH*HC*$^-$Tf$_2$), 3.73 (1H, s, C*H*HC$^-$Tf$_2$), 2.32 (3H, s, CH_3). ^{13}C{1H} NMR (d_6-acetone, 75 MHz, 25 °C): δ = 163.0 (C=O), 161.2 (C_{Ar}, C-13), 153.9 (*C*=C, C-2), 141.0 (C_{Ar}, C-11), 139.7 (C_{Ar}, C-4), 134.7 (CH_{Ar}, C-8), 133.4 (CH_{Ar}, C-15), 129.4 (CH_{Ar}, C-6), 128.0 (CH_{Ar}, C-7), 126.7 (C_{Ar}, C-10), 126.6 (CH_{Ar}, C-9), 122.2 (2C, q, J_{C-F} = 329.4 Hz, $2CF_3$), 121.9 (C_{Ar}, C-5), 116.4 (CH_{Ar}, C-12), 113.4 (C=*C*, C-3), 111.1 (CH_{Ar}, C-14), 64.4-64.3 (m, $^-CTf_2$), 55.5 (OCH_3), 27.5 (CH_2), 20.2 (CH_3). ^{19}F NMR (d_6-acetone, 282 MHz, 25 °C): δ = -74.6 (s, 6F, $2CF_3$); HRMS (ESI-TOF) m/z: $[M]^-$ Calcd for $[C_{21}H_{15}F_6O_7S_2]^-$ 557.0169; Found 557.0172.

Compound **3g**

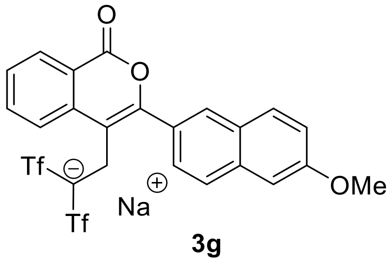

3g

Following the general procedure, starting from 23 mg of 2-ethynylbenzoate **2g**, 32 mg (72%) of compound **3g** were obtained as a colorless thick oil, after purification on column chromatography with gradient elution using hexanes/AcOEt (1:1 to 1:3) as solvent.

^{1}H NMR (d_6-acetone, 300 MHz, 25 °C): δ = 8.70 (1H, d, *J* = 8.6 Hz, CH_{Ar}), 8.25 (1H, dd, *J* = 7.9, 1.5 Hz, CH_{Ar}), 8.08 (1H, s, CH_{Ar}), 7.92 (1H, d, *J* = 8.9 Hz, CH_{Ar}), 7.88 (1H, d, *J* = 8.6 Hz, CH_{Ar}), 7.83 (1H, ddd, *J* = 8.5, 7.2, 1.5 Hz, CH_{Ar}), 7.68 (1H, dd, *J* = 8.5, 1.7 Hz, CH_{Ar}), 7.57 (1H, ddd, *J* = 8.1, 7.2, 1.1 Hz, CH_{Ar}), 7.37 (1H, d, *J* = 2.6 Hz, CH_{Ar}), 7.21 (1H, dd, *J* = 8.9, 2.6 Hz, CH_{Ar}), 4.03 (2H, s, CH_2), 3.96 (3H, s, OCH_3). ^{13}C{1H} NMR (d_6-acetone, 75 MHz, 25 °C): δ = 162.4 (C=O), 159.0 (C_{Ar}), 154.0 (*C*=C), 139.2 (C_{Ar}), 135.3 (C_{Ar}), 134.4 (CH_{Ar}), 130.3 (CH_{Ar}), 130.2 (CH_{Ar}), 129.9 (C_{Ar}), 129.0 (CH_{Ar}), 128.6 (CH_{Ar}), 128.5 (C_{Ar}), 127.6 (CH_{Ar}), 126.6 (CH_{Ar}), 126.1 (CH_{Ar}), 123.8 (C_{Ar}), 121.4 (C_{Ar}), 119.5 (CH_{Ar}), 112.2 (C=*C*), 106.1 (CH_{Ar}), 64.1-63.9 (m, $^{-}CTf_2$), 55.2 (OCH_3), 26.8 (CH_2). ^{19}F NMR (d_6-acetone, 282 MHz, 25 °C): δ = -79.7 (s, 6F, $2CF_3$); HRMS (ESI-TOF) m/z: $[M]^-$ Calcd for $[C_{24}H_{15}F_6O_7S_2]^-$ 593.0169; Found 593.0165.

Compound **3h**

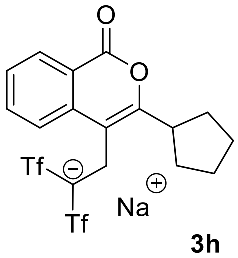

3h

Following the general procedure, starting from 12 mg of 2-ethynylbenzoate **2h** and after purification on column chromatography using hexanes/AcOEt/diethyl ether (1:3:1) as solvent, compound **3h** was partially isolated from complex reaction mixture, lacking purity to consider full characterization.

Compound **3i**

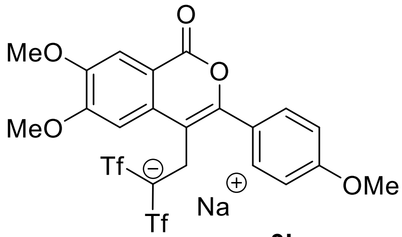

3i

Following the general procedure, starting from 21 mg of 2-ethynylbenzoate **2i**, 29 mg (71%) of compound **3i** were obtained as a colorless thick oil, after purification on column chromatography using hexanes/AcOEt (3:7) as solvent.

^{1}H NMR (d_6-acetone, 300 MHz, 25 °C): δ = 8.13 (1H, s, CH_{Ar}), 7.58 (1H, s, CH_{Ar}), 7.52 (2H, *AA'*XX', CH_{PMP}), 6.98 (2H, AA'*XX'*, CH_{PMP}), 3.99 (3H, s, OMe), 3.93 (3H, s, OMe), 3.91 (2H, s, $CH_2C^-Tf_2$), 3.86 (3H, s, OMe); ^{13}C{1H} NMR (d_6-acetone, 75 MHz, 25 °C): δ = 162.6 (C=O), 161.0 (C_{Ar}), 155.9 (C_{Ar}), 153.3 (*C*=C), 149.9 (C_{Ar}), 135.4 (C_{Ar}), 132.5 (2 x CH_{PMP}), 127.8 (C_{Ar}), 122.6 (q, J_{C-F} = 329.4 Hz, 2 x SO_2CF_3), 114.8 (C_{Ar}), 113.9 (2 x CH_{PMP}), 111.9 (C=*C*), 109.7 (CH_{Ar}), 108.4 (CH_{Ar}), 64.4 (C^-Tf_2), 57.5 (OMe), 56.1 (OMe), 55.6 (OMe), 27.5 ($CH_2C^-Tf_2$); ^{19}F NMR (d_6-acetone, 282 MHz, 25 °C): δ = -79.6 (6F, s, 2 x SO_2CF_3); HRMS (ESI-TOF) m/z: $[M]^-$ Calcd for $[C_{22}H_{17}F_6O_9S_2]^-$ 603.0224; Found 603.0241.

Compound **3j**

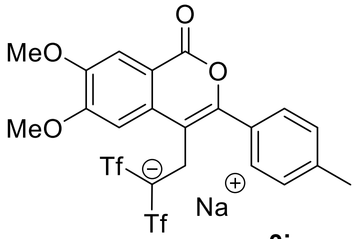

3j

Following the general procedure, starting from 23 mg of 2-ethynylbenzoate **2j**, 37 mg (80%) of compound **3j** were obtained as a colorless thick oil, after purification on column chromatography using hexanes/AcOEt (3:7) as solvent.

^{1}H NMR (d_6-acetone, 300 MHz, 25 °C): δ = 8.15 (1H, s, CH_{Ar}), 7.58 (1H, s, CH_{Ar}), 7.47 (2H, *AA'*XX', 2 x CH_{Ar}), 7.25 (2H, AA'*XX'*, 2 x CH_{Ar}), 3.99 (3H, s, OMe), 3.93 (3H, s, OMe), 3.91 (2H, s br, $CH_2C^-Tf_2$), 2.38 (3H, s, CH_3); ^{13}C{1H} NMR (d_6-acetone, 75 MHz, 25 °C): δ = 162.5 (C=O), 155.9 (C_{Ar}), 153.4 (*C*=C), 150.0 (C_{Ar}), 139.4 (C_{Ar}), 135.4 (C_{Ar}), 132.8 (C_{Ar}), 131.1 (2 x CH_{Ar}), 129.2 (2 x CH_{Ar}), 122.1 (q, J_{C-F} = 328.6 Hz, 2 x SO_2CF_3), 114.8 (C=*C*), 112.0 (C_{Ar}), 109.7 (CH_{Ar}), 108.5 (CH_{Ar}), 64.6 (C^-Tf_2), 57.5 (OMe), 56.1 (OMe), 27.5 ($CH_2C^-Tf_2$), 21.4 (CH_3); ^{19}F NMR (d_6-acetone, 282 MHz, 25 °C): δ = -79.8 (6F, s, 2 x SO_2CF_3); HRMS (ESI-TOF) m/z: $[M]^-$ Calcd for $[C_{22}H_{17}F_6O_8S_2]^-$ 587.0275; Found 587.0302.

Compound **3k**

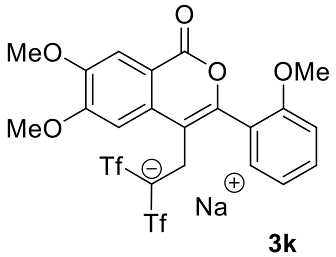

3k

Following the general procedure, starting from 23 mg of 2-ethynylbenzoate **2k**, 32 mg (74%) of compound **3k** were obtained as a colorless thick oil, after purification on column chromatography using hexanes/AcOEt (3:7) as solvent.

^{1}H NMR (d$_6$-acetone, 300 MHz, 25 °C): δ = 8.18 (1H, s, CH$_{Ar}$), 7.53 (1H, d, *J* = 7.3 Hz, CH$_{Ar}$), 7.49 (1H, s, CH$_{Ar}$), 7.44-7.38 (2H, m, CH$_{Ar}$), 7.06 (1H, d, *J* = 8.3 Hz, CH$_{Ar}$), 7.00 (1H, dd, *J* = 7.4, 1.0 Hz, CH$_{Ar}$), 3.99 (3H, s, OMe), 3.93 (3H, s, OMe), 3.79 (3H, s, OMe), 3.77-3.65 (1H, m, C*H*HC$^-$Tf$_2$), 3.59-3.46 (1H, m, CH*H*C$^-$Tf$_2$); ^{13}C{1H} NMR (d$_6$-acetone, 75 MHz, 25 °C): δ = 162.9 (C=O), 155.8 (C$_{Ar}$), 149.9 (C$_{Ar}$), 135.2 (C$_{Ar}$), 133.3 (CH$_{Ar}$), 132.7 (C$_{Ar}$), 131.4 (CH$_{Ar}$), 124.2 (C$_{Ar}$), 122.1 (q, J_{C-F} = 331.6 Hz, 2 x SO$_2$CF$_3$), 120.7 (CH$_{Ar}$), 114.9 (C=*C*), 113.8 (C$_{Ar}$), 111.7 (CH$_{Ar}$), 109.7 (CH$_{Ar}$), 108.4 (CH$_{Ar}$), 64.2 (*C*$^-$Tf$_2$), 57.5 (OMe), 56.1 (OMe), 55.9 (OMe), 27.9 (*CH$_2$*C$^-$Tf$_2$); ^{19}F NMR (d$_6$-acetone, 282 MHz, 25 °C): δ = -79.8 (6F, s, 2 x SO$_2$CF$_3$); HRMS (ESI-TOF) m/z: [M]$^-$ Calcd for [C$_{22}$H$_{17}$F$_6$O$_9$S$_2$]$^-$ 603.0224; Found 603.0224.

Compound **3l**

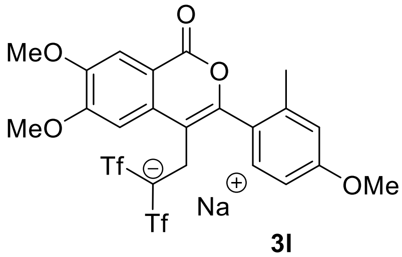

3l

Following the general procedure, starting from 21 mg of 2-ethynylbenzoate **2l**, 30 mg (77%) of compound **3l** were obtained as a colorless thick oil, after purification on column chromatography using hexanes/AcOEt (3:7) as solvent.

^{1}H NMR (d$_6$-acetone, 300 MHz, 25 °C): δ = 8.18 (1H, s, CH$_{Ar}$), 7.59 (1H, s, CH$_{Ar}$), 7.33 (1H, br s, CH$_{Ar}$), 6.84 (1H, d, *J* = 2.6 Hz, CH$_{Ar}$), 6.79 (1H, dd, *J* = 8.4, 2.7 Hz, CH$_{Ar}$), 3.99 (3H, s, OCH$_3$), 3.93 (3H, s, OCH$_3$), 3.83 (s, 4H, OCH$_3$; C*H*HC$^-$Tf$_2$), 3.69 (s, 1H, CH*H*C$^-$Tf$_2$), 2.30 (s, 3H, CH$_3$); ^{13}C{1H} NMR (d$_6$-acetone, 75 MHz, 25 °C): δ = 162.7 (C=O), 161.1 (C$_{Ar}$), 155.8 (C$_{Ar}$), 149.9 (C$_{Ar}$), 135.3 (C$_{Ar}$),

133.3 (CH_{Ar}), 127.0 (C_{Ar}), 122.1 (q, $J_{C\text{-}F}$ = 329.5 Hz, 2 x SO_2CF_3), 116.4 (C_{Ar}), 114.8 (C_{Ar}), 113.0 (*C*=C), 111.0 (CH_{Ar}), 109.7 (CH_{Ar}), 108.5 (CH_{Ar}), 90.8 (C_{Ar}), 64.3 ($C^{-}Tf_2$), 57.5 (OCH_3), 56.1 (OCH_3), 55.4 (OCH_3), 27.7 ($CH_2C^{-}Tf_2$), 20.2 (CH_3); ^{19}F NMR (d_6-acetone, 282 MHz, 25 °C): δ = -79.8 (6F, s, 2 x SO_2CF_3); HRMS (ESI-TOF) m/z: $[M]^-$ Calcd for $[C_{23}H_{19}F_6O_9S_2]^-$ 617.0380; Found 617.0382.

Compound **3m**

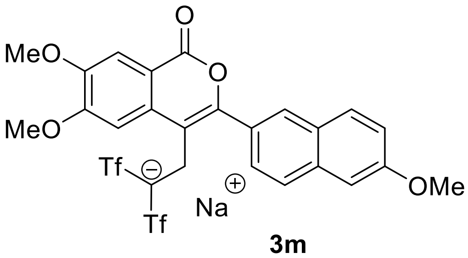

3m

Following the general procedure, starting from 12 mg of 2-ethynylbenzoate **2m**, 14 mg (66%) of compound **3m** were obtained as a colorless thick oil, after purification on column chromatography using hexanes/AcOEt (3:7) as solvent.

^{1}H NMR (d_6-acetone, 300 MHz, 25 °C): δ = 8.18 (1H, s, CH_{Ar}), 8.05 (1H, s br, CH_{Ar}), 7.90 (1H, d, *J* = 9.0 Hz, CH_{Ar}), 7.86 (1H, d, *J* = 8.5 Hz, CH_{Ar}), 7.66 (1H, dd, *J* = 8.5, 1.8 Hz, CH_{Ar}), 7.61 (1H, s, CH_{Ar}), 7.35 (1H, d, *J* = 8.6 Hz, CH_{Ar}), 7.19 (1H, dd, *J* = 8.9, 2.6 Hz, CH_{Ar}), 4.01 (3H, s, OMe), 4.00 (2H, s, $CH_2C^{-}Tf_2$), 3.95 (6H, s, 2 x OMe); ^{13}C{1H} NMR (d_6-acetone, 75 MHz, 25 °C): δ = 162.6 (C=O), 159.5 (C_{Ar}), 155.9 (C_{Ar}), 153.5 (*C*=C), 150.1 (C_{Ar}), 142.8 (C_{Ar}), 141.0 (C_{Ar}), 135.7 (C_{Ar}), 135.4 (CH_{Ar}), 130.8 (CH_{Ar}), 130.7 (C_{Ar}), 130.5 (CH_{Ar}), 129.1 (CH_{Ar}), 129.0 (C_{Ar}), 127.0 (CH_{Ar}), 120.3 (q, $J_{C\text{-}F}$ = 321.4 Hz, 2 x SO_2CF_3), 119.9 (C_{Ar}), 114.9 (C_{Ar}), 112.4 (*C*=C), 109.7 (CH_{Ar}), 108.5 (CH_{Ar}), 106.6 (CH_{Ar}), 68.8 ($C^{-}Tf_2$), 57.5 (OMe), 56.1 (OMe), 55.7 (OMe), 27.6 ($CH_2C^{-}Tf_2$); ^{19}F NMR (d_6-acetone, 282 MHz, 25 °C): δ = -79.8 (6F, s, 2 x SO_2CF_3); IR (CH_2Cl_2): ν = 2970, 1710, 1189 cm^{-1}; HRMS (ESI-TOF) m/z: $[M]^-$ Calcd for $[C_{26}H_{19}F_6O_9S_2]^-$ 653.0380; Found 653.0384.

Compound **3n**

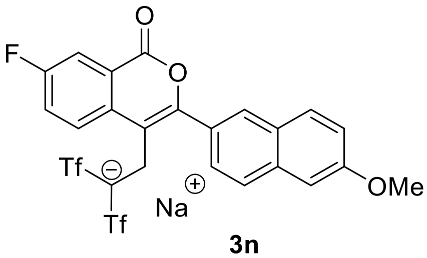

3n

Following the general procedure, starting from 24 mg of 2-ethynylbenzoate **2n**, 23 mg (51%) of compound **3n** were obtained as a colorless thick oil, after purification on column chromatography using hexanes/AcOEt (3:7) as solvent.

^{1}H NMR (d_6-acetone, 300 MHz, 25 °C): δ = 8.76 (1H, dd, *J* = 9.1, 5.1 Hz, CH_{Ar}), 8.07 (1H, br s, CH_{Ar}), 7.94-7.86 (3H, m, CH_{Ar}), 7.69-7.66 (1H, m, CH_{Ar}), 7.66-7.64 (1H, m, CH_{Ar}), 7.36 (1H, d, *J* = 2.6 Hz, CH_{Ar}), 7.20 (1H, dd, *J* = 8.9, 2.6 Hz, CH_{Ar}), 4.01 (s br, 2H, $CH_2C^{-}Tf_2$), 3.95 (s, 3H, OCH_3); ^{13}C{1H} NMR (d_6-acetone, 75 MHz, 25 °C): δ = 162.2 (d, J_{C-F} = 246.0 Hz, C_{Ar-F}), 162.0 (d, $^{4}J_{C-F}$ = 3.4 Hz, C=O), 159.6 (C_{Ar-OMe}), 154.0 (d, J_{C-F} = 2.7 Hz, C_{Ar}), 149.9 (*C*=C), 136.5 (d, J_{C-F} = 2.3 Hz, C_{Ar}), 135.9 (C_{Ar}), 130.8 (CH_{Ar}), 130.7 (CH_{Ar}), 130.1 (C_{Ar}), 129.7 (d, $^{3}J_{C-F}$ = 7.6 Hz, CH_{Ar}), 129.0 (CH_{Ar}), 127.1 (CH_{Ar}), 124.3 (C_{Ar}), 123.7 (d, $^{3}J_{C-F}$ = 8.3 Hz, C_{Ar}), 122.8 (d, $^{2}J_{C-F}$ = 22.5 Hz, CH_{Ar}), 120.1 (q, J_{C-F} = 324.7 Hz, 2 x SO_2CF_3), 120.0 (CH_{Ar}), 114.4 (d, $^{2}J_{C-F}$ = 23.1 Hz, CH_{Ar}), 112.3 (*C*=C), 106.63 (CH_{Ar}), 64.6 ($C^{-}Tf_2$), 55.7 (OMe), 27.4 ($CH_2C^{-}Tf_2$); ^{19}F NMR (d_6-acetone, 282 MHz, 25 °C): δ = -79.8 (6F, s, 2 x SO_2CF_3), -114.8 (1F, s, F); IR (CH_2Cl_2): ν = 2920, 1712, 1190 cm^{-1}; HRMS (ESI-TOF) m/z: $[M]^{-}$ Calcd for $[C_{24}H_{14}F_7O_7S_2]^{-}$ 611.0075; Found 611.0079.

Compound **3o**

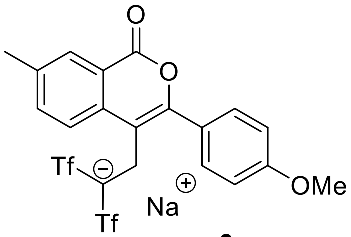

3o

Following the general procedure, starting from 23 mg of 2-ethynylbenzoate **2o**, 36 mg (78%) of compound **3o** were obtained as a colorless thick oil, after purification on column chromatography using hexanes/AcOEt (3:7) as solvent.

^{1}H NMR (d_6-acetone, 300 MHz, 25 °C): δ = 8.58 (1H, d, *J* = 8.4 Hz, CH_{Ar}), 8.03 (1H, br s, CH_{Ar}), 7.63 (1H, dd, *J* = 8.6, 1.9 Hz, CH_{Ar}), 7.53 (2H, *AA'*XX', CH_{PMP}), 7.00 (2H, AA'*XX'*, CH_{PMP}), 3.93 (s, 2H, $CH_2C^{-}Tf_2$), 3.87 (s, 3H, OCH_3), 2.49 (3H, s, CH_3); ^{13}C{1H} NMR (d_6-acetone, 75 MHz, 25 °C): δ = 163.0 (C=O), 161.1 (C_{Ar}), 151.0 (*C*=C), 137.8 (C_{Ar}), 137.4 (C_{Ar}), 135.9 (CH_{Ar}), 132.6 (2 x CH_{PMP}), 129.1 (CH_{Ar}), 127.8 (C_{Ar}), 126.6 (CH_{Ar}), 121.8 (C_{Ar}), 113.9 (2 x CH_{PMP}), 112.2 (C=*C*), 65.8 ($C^{-}Tf_2$), 55.6 (OMe), 27.3 ($CH_2C^{-}Tf_2$), 21.1 (CH_3); ^{19}F NMR (d_6-acetone, 282 MHz, 25 °C): δ = -79.7 (6F, s, 2 x SO_2CF_3); HRMS (ESI-TOF) m/z: $[M]^{-}$ Calcd for $[C_{21}H_{15}F_6O_7S_2]^{-}$ 557.0169; Found 557.0168.

Compound **3p**

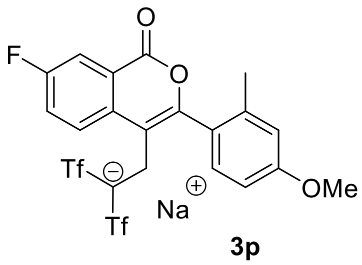

3p

Following the general procedure, starting from 22 mg of 2-ethynylbenzoate **2p**, 30 mg (68%) of compound **3p** were obtained as a colorless thick oil, after purification on column chromatography with gradient elution using hexanes/AcOEt (1:1 to 3:7) as solvent.

^{1}H NMR (d$_6$-acetone, 300 MHz, 25 °C): δ = 8.77 (1H, dd, *J* = 8.9, 5.2 Hz, CH$_{Ar}$), 7.86 (1H, dd, *J* = 8.9, 2.9 Hz, CH$_{Ar}$), 7.62 (1H, td, *J* = 8.7, 2.9 Hz, CH$_{Ar}$), 7.35 (1H, d, *J* = 8.4 Hz, CH$_{Ar}$), 6.86 (1H, d, *J* = 2.6 Hz, CH$_{Ar}$), 6.80 (1H, dd, *J* = 8.4, 2.6 Hz, CH$_{Ar}$), 3.83 (4H, s, OCH$_3$; CH*H*C$^-$Tf$_2$), 3.71 (1H, s, CH*H*C$^-$Tf$_2$), 2.32 (3H, s, CH$_3$). ^{13}C{1H} NMR (d$_6$-acetone, 75 MHz, 25 °C): δ = 161.4 (d, $^4J_{C-F}$ = 3.3 Hz, C=O), 161.3 (d, $^1J_{C-F}$ = 246.2 Hz, C$_{Ar-F}$), 160.5 (C$_{Ar-OMe}$), 152.5 (*C*=C), 140.3 (C$_{Ar}$), 135.6 (C$_{Ar}$), 132.6 (CH$_{Ar}$), 128.8 (d, $^3J_{C-F}$ = 7.6 Hz, CH$_{Ar}$), 125.5 (C$_{Ar}$), 122.8 (d, $^3J_{C-F}$ = 7.8 Hz, C$_{Ar}$), 121.8 (d, $^2J_{C-F}$ = 22.5 Hz, CH$_{Ar}$), 121.3 (q, J_{C-F} = 329.2 Hz, 2 x CF$_3$), 115.5 (CH$_{Ar}$), 113.5 (d, $^2J_{C-F}$ = 23.1 Hz, CH$_{Ar}$), 112.1 (C=*C*), 110.3 (CH$_{Ar}$), 63.4-63.3 (m, *C*$^-$Tf$_2$), 54.6 (OCH$_3$), 26.6 (CH$_2$), 19.3 (CH$_3$). ^{19}F NMR (d$_6$-acetone, 282 MHz, 25 °C): δ = -79.8 (6F, s, 2 x SO$_2$CF$_3$), -114.9 (1F, s, F); IR (CH$_2$Cl$_2$): ν = 2974, 1700, 1180 cm^{-1}; HRMS (ESI-TOF) m/z: [M]$^-$ Calcd for [C$_{21}$H$_{14}$F$_7$O$_7$S$_2$]$^-$ 575.0075; Found 575.0072.

Compound **3q**

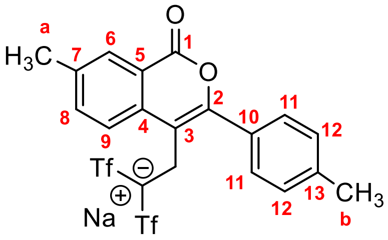

3q

Following the general procedure, starting from 22 mg of 2-ethynylbenzoate **2q**, 43 mg (88%) of compound **3q** were obtained as a colorless thick oil, after purification on column chromatography using hexanes/AcOEt (3:7) as solvent.

^{1}H NMR (d$_6$-acetone, 300 MHz, 25 °C): δ = 8.56 (1H, d, *J* = 8.3 Hz, CH$_{Ar}$, H-9), 8.02 (1H, s$_{br}$, CH$_{Ar}$, H-6), 7.62 (1H, dd, *J* = 8.3, 2.1 Hz, CH$_{Ar}$, H-8), 7.47 (2H, *AA'*XX', CH$_{Ar}$, H-11), 7.25 (2H, AA'*XX'*, CH$_{Ar}$, H-12), 3.92 (2H, s, *CH$_2$*C$^-$Tf$_2$), 2.48 (3H, s, CH$_3$), 2.38 (3H, s, CH$_3$); ^{13}C{1H} RMN (d$_6$-acetone, 75 MHz, 25 °C): δ = 162.9 (C=O), 153.6 (*C*=C, C-2), 139.6 (C$_{Ar}$, C-13), 137.9 (C$_{Ar}$, C-7), 137.3 (C$_{Ar}$, C-4),

136.0 (CH_{Ar}, C-8), 132.5 (C_{Ar}, C-10), 131.1 (2 x CH_{Ar}, C-11), 129.2 (2 x CH_{Ar}, C-12), 129.1 (CH_{Ar}, C-6), 126.6 (CH_{Ar}, C-9), 122.1 (2C, q, J_{C-F} = 331.4 Hz, 2 x SO_2CF_3), 121.8 (C_{Ar}, C-5), 112.3 (C=*C*, C-3), 64.5 (C^-Tf_2), 27.2 (CH_2), 21.4 (CH_3), 21.1 (CH_3); ^{19}F NMR (d_6-acetone, 282 MHz, 25 °C): δ = -79.8 (6F, s, 2 x SO_2CF_3). HRMS (ESI-TOF) m/z: $[M]^-$ Calcd for $[C_{21}H_{15}F_6O_6S_2]^-$ 541.0220; Found 541.0221.

Compound **3r**

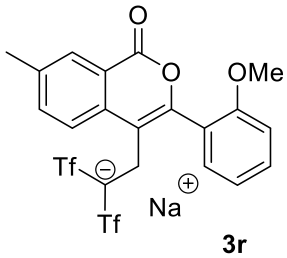

3r

Following the general procedure, starting from 20 mg of 2-ethynylbenzoate **2r**, 31 mg (77%) of compound **3r** were obtained as a colorless thick oil, after purification on column chromatography with gradient elution using hexanes/AcOEt (1:1 to 3:7) as solvent.

^1H NMR (d_6-acetone, 300 MHz, 25 °C): δ = 8.60 (1H, d, *J* = 8.3 Hz, CH_{Ar}), 8.06–7.98 (1H, m, CH_{Ar}), 7.62 (1H, ddd, *J* = 8.3, 2.0, 0.7 Hz, CH_{Ar}), 7.54 (1H, d, *J* = 6.9 Hz, CH_{Ar}), 7.06 (1H, dd, *J* = 8.5, 1.0 Hz, CH_{Ar}), 7.00 (1H, dd, *J* = 7.5, 1.0 Hz, CH_{Ar}), 3.78 (4H, s, OCH_3; CH*HC*$^-Tf_{2174}$), 3.55 (1H, s, CH_2), 2.48 (3H, s, CH_3). $^{13}C\{1H\}$ NMR (d_6-acetone, 75 MHz, 25 °C): δ = 163.3 (C_{Ar}), 158.3 (C=O), 137.9 (C_{Ar}), 137.2 (C_{Ar}), 135.9 (CH_{Ar}), 133.4 (CH_{Ar}), 131.5 (CH_{Ar}), 129.1 (CH_{Ar}), 126.5 (CH_{Ar}), 124.0 (C_{Ar}), 122.2 (q, J_{C-F} = 329.3 Hz, 2 x CF_3), 121.9 (C_{Ar}), 120.7 (CH_{Ar}), 114.2 (C=*C*), 111.7 (CH_{Ar}), 64.3 (C^-Tf_2), 55.9 (OCH_3), 27.7 (CH_2), 21.2 (CH_3). ^{19}F NMR (d_6-acetone, 282 MHz, 25 °C): δ = -79.7 (6F, s, 2 x SO_2CF_3). HRMS (ESI-TOF) m/z: $[M]^-$ Calcd for $[C_{21}H_{15}F_6O_7S_2]^-$ 557.0169; Found 557.0171.

Compound **4s-*p*-CF₃**

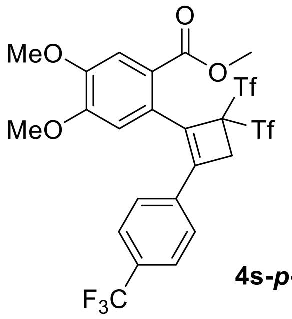

4s-*p*-CF3

Following the general procedure, starting from 23 mg of 2-ethynylbenzoate **2s-*p*-CF_3**, 9 mg (21%) of compound **4s-*p*-CF_3** were obtained as a colorless thick oil, after purification on column chromatography using hexanes/AcOEt (3:7) as solvent.

^{1}H NMR ($CDCl_3$, 300 MHz, 25 °C): δ = 7.62 (2H, *AA'*XX', CH_{Ar}), 7.48 (1H, s, CH_{Ar}), 7.42 (2H, AA'*XX'*, CH_{Ar}), 7.22 (1H, s, CH_{Ar}), 4.00 (3H, s, OMe), 3.93 (3H, s, OMe), 3.73 (2H, s, $CH_2C^-Tf_2$), 3.61 (3H, s, CO_2CH_3); ^{13}C{1H} NMR ($CDCl_3$, 125 MHz, 25 °C): δ = 166.3 (C=O), 151.6 (C_{Ar}), 149.6 (C_{Ar}), 134.0 (C=*C*), 132.8 (*C*=C), 131.5 (q, *J* = 32.9 Hz, C_{Ar}), 128.3 (q, $J_{C\text{-}F}$ = 257.7 Hz, CF_3), 127.7 (2 x CH_{Ar}), 125.7 (q, $J_{C\text{-}F}$ = 3.7 Hz, 2 x CH_{Ar}), 124.2 (C_{Ar}), 123.1 (C_{Ar}), 122.4 (C_{Ar}), 113.2 (CH_{Ar}), 112.4 (CH_{Ar}), 56.2 (OMe), 56.1 (OMe), 52.3 (CO_2CH_3), 33.4 (*CH_2*CTf_2); ^{19}F NMR ($CDCl_3$, 282 MHz, 25 °C): δ = -63.6 (6F, s, 2 x SO_2CF_3), -69.5 (3F, m, CF_3); HRMS (ESI-TOF) m/z: $[M + H]^+$ Calcd for $C_{23}H_{18}F_9O_8S_2$ 657.0294; Found 587.0273.

Compound **4s-*o*-CF₃**

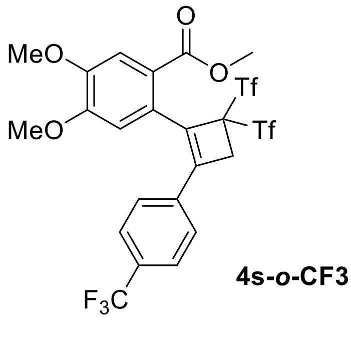

4s-*o*-CF3

Following the general procedure, starting from 27 mg of 2-ethynylbenzoate **2s-*o*-CF_3**, 31 mg (63%) of compound **4s-*o*-CF_3** were obtained as a colorless oil, after purification on column chromatography using hexanes/AcOEt (3:7) as solvent.

^{1}H NMR ($CDCl_3$, 300 MHz, 25 °C): δ = 7.75 (1H, *J* = 7.8 Hz, H_{Ar}), 7.52-7.40 (2H, m, H_{Ar}), 7.43 (1H, s, H_{Ar}), 7.35 (1H, s, H_{Ar}), 7.32 (1H, s, H_{Ar}), 3.96 (3H, s, OMe), 3.95 (3H, s, OMe), 3.85 (2H, br s, CH_2), 3.54 (3H, s, OMe); ^{13}C{1H} NMR ($CDCl_3$, 125 MHz, 25 °C): δ = 166.7 (C=O), 151.7 (C_{Ar}), 149.5 (C_{Ar}), 132.1 (CH_{Ar}), 131.9 (C_{Ar}), 130.9 (CH Ar), 130.6 (CH_{Ar}), 129.7 (C_{Ar}), 128.6 (1C, q, *J* = 142 Hz, CF_3), 126.6 (1C, q, *J* = 5.5 Hz, CH_{Ar}), 126.3 (2C, q, *J* = 135 Hz, CF_3), 124.2 (C_{Ar}), 122.3 (*C*=C), 122.0 (C=*C*), 117.6 (C_{Ar}), 113.6 (CH_{Ar}), 112.7 (CH_{Ar}), 88.8 (C), 56.3 (OMe), 56.1 (OMe), 52.4 (OMe), 37.05 (CH_2); ^{19}F NMR ($CDCl_3$, 282 MHz, 25 °C): δ = -57.8 (3F, CF_3), -69.2 (6F, 2 x SO_2CF_3); HRMS (ESI-TOF) m/z: $[M + H]^+$ Calcd for $C_{23}H_{18}F_9O_8S_2$ 657.0294; Found 657.0310.

Compound [D]-**3i**

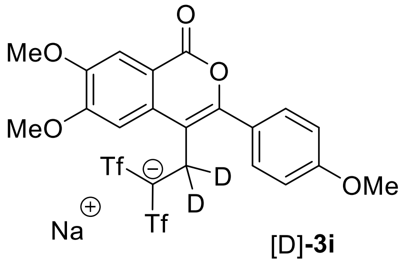

[D]-**3i**

Following the general procedure, starting from 19 mg of 2-ethynylbenzoate **2i** and deuterated zwitterion [D]-**1**, 28 mg (86%) of compound [D]-**3i** were obtained as a colorless thick oil, after purification on column chromatography using hexanes/AcOEt (3:7) as solvent.

^{1}H NMR (d$_6$-acetone, 300 MHz, 25 °C): δ = 8.13 (1H, s, CH$_{Ar}$), 7.58 (1H, s, CH$_{Ar}$), 7.51 (2H, *AA'*XX', CH$_{PMP}$), 6.99 (2H, AA'*XX'*, CH$_{PMP}$), 3.99 (3H, s, OMe), 3.93 (s, 3H, OMe), 3.86 (s, 3H, OMe); ^{13}C{1H} NMR (d$_6$-acetone, 75 MHz, 25 °C): δ = 162.6 (C=O), 161.0 (C$_{Ar}$), 155.9 (C$_{Ar}$), 153.3 (*C*=C), 149.9 (C$_{Ar}$), 135.4 (C$_{Ar}$), 132.5 (2 x CH$_{PMP}$), 127.9 (C$_{Ar}$), 122.1 (q, $J_{C\text{-}F}$ = 325.8 Hz, 2 x SO$_2$CF$_3$), 117.7 (C$_{Ar}$), 114.8 (C=*C*), 113.9 (2 x CH$_{PMP}$), 111.8 (C$_{Ar}$), 109.7 (CH$_{Ar}$), 108.4 (CH$_{Ar}$), 64.5 (C$^-$Tf$_2$), 57.5 (OCH$_3$), 56.1 (OCH$_3$), 55.6 (OCH$_3$); ^{19}F NMR (d$_6$-acetone, 282 MHz, 25 °C): δ = -79.8 (6F, s, 2 x SO$_2$CF$_3$); HRMS (ESI-TOF) m/z: [M]$^-$ Calcd for [$C_{22}H_{15}D_2F_6O_9S_2$]$^-$ 605.0349; Found 605.0351.

2.2. Azacyclization *vs* carbocyclization reactions: synthesis of isoquinolinones **7** and cyclobutenes **8**.

To a solution of 2-ethynylbenzamides **5** and 2-ethynylbenzenesulfonamides **6** (1 equiv.) in MeCN/DCE 1:1 (20 mL/mmol) was added zwitterion **1** (1 equiv.) in one portion. The reaction mixture was stirred at 60 °C under microwave irradiation for 1.5h. After that time, the solvent was evaporated and the crude mixture was purified on column chromatography, yielding the indicated isoquinolinones **7** as sodium salts, or cyclobutenes **8**.

Table S6

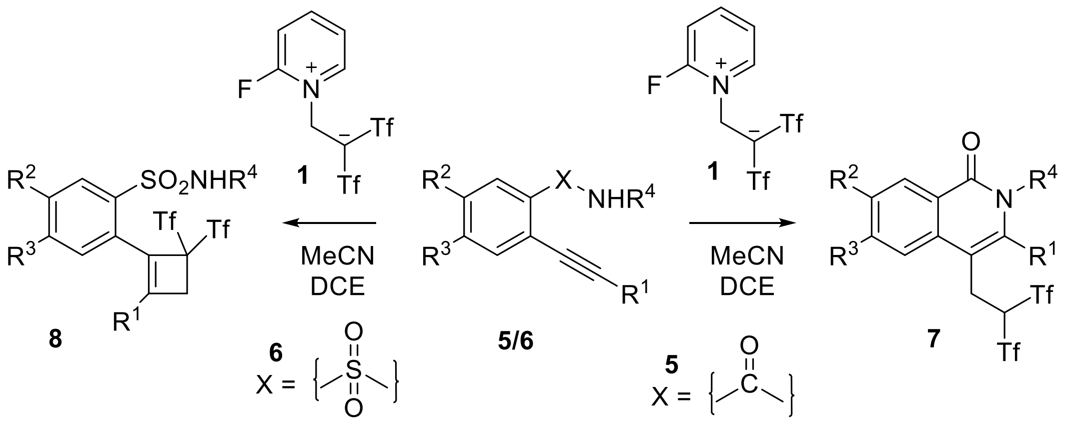

Entry	5/6	R^4	X	R^1	2 (%)	10 (%)
1	**5a**	Ph	C=O	4-Me-C$_6$H$_4$	**7a** (75)	–
2	**5b**	Ph	C=O	2-Me-4-OMe-C$_6$H$_3$	**7b** (88)	–
3	**5c**	4-OMe-C$_6$H$_4$	C=O	4-Me-C$_6$H$_4$	**7c** (82)	–
4	**5d**	4-OMe-C$_6$H$_4$	C=O	4-OMe-C$_6$H$_4$	**7d** (76)	–
5	**5e**	4-OMe-C$_6$H$_4$	C=O	2-Me-4-OMe-C$_6$H$_3$	**7e** (83)	–
6	**6a**	4-OMe-C$_6$H$_4$	SO$_2$	4-Me-C$_6$H$_4$	–	**8a**(59)
7	**6b**	4-OMe-C$_6$H$_4$	SO$_2$	4-OMe-C$_6$H$_4$	–	**8b**(61)

Compound **7a**

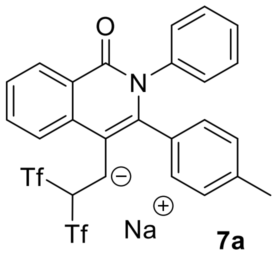

7a

Following the general procedure, starting from 20 mg of 2-ethynylbenzamide **5a**, 30 mg (75%) of compound **7a** were obtained as a yellow thick oil, after purification on column chromatography using hexanes/AcOEt (5:7) as solvent.

^{1}H NMR ($CDCl_3$, 300 MHz, 25 °C): δ = 8.29 (1H, d, *J* = 7.9 Hz, CH_{Ar}), 8.15 (1H, d, *J* = 8.0 Hz, CH_{Ar}), 7.54 (1H, t, *J* = 7.9 Hz, CH_{Ar}), 7.18 (10H, m, CH_{Ar}), 3.90 (2H, s, CH_2), 3.23 (3H, s, Me); ^{13}C{1H} NMR (d_6-acetone, 75 MHz, 25 °C): δ = 151.9 (C=O), 150.6 (C_{Ar}), 146.8 (C_{Ar}), 138.6 (C_{Ar}), 135.5 (C=C), 131.7 (CH_{Ar}), 131.5 (C_{Ar}), 130.1 ($2CH_{Ar}$), 128.4 ($2CH_{Ar}$), 128.2 ($2CH_{Ar}$), 126.9 (CH_{Ar}), 126.6 (CH_{Ar}), 125.2 (CH_{Ar}), 123.8 (C_{Ar}), 123.5 ($2CF_3$), 123.1 (CH_{Ar}), 122.9 ($2CH_{Ar}$), 110.6 (C=C), 63.5 (C^-Tf_2), 26.2 (CH_2), 20.4 (CH_3); ^{19}F NMR (d_6-acetone, 282 MHz, 25 °C): δ = -79.7 (6F, s, 2 x SO_2CF_3); IR (CH_2Cl_2): ν = 2974, 1700, 1174 cm^{-1}; HRMS (ESI-TOF) m/z: $[M]^-$ Calcd for $[C_{26}H_{18}F_6NO_5S_2]^-$ 602.0531; Found 602.0533.

Compound **7b**

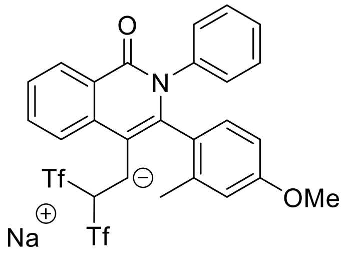

7b

Following the general procedure, starting from 18 mg of 2-ethynylbenzamide **5b**, 29 mg (88%) of compound **7b** were obtained as a yellow thick oil, after purification on column chromatography using hexanes/AcOEt (3:7) as solvent.

^{1}H NMR (d_6-acetone, 300 MHz, 25 °C): δ = 8.57 (1H, d, *J* = 7.8 Hz, CH_{Ar}), 8.37 (1H, dd, *J* = 7.9, 1.1 Hz, CH_{Ar}), 7.63 (1H, ddd, *J* = 8.1, 7.2, 1.5 Hz, CH_{Ar}), 7.46 (1H, ddd, *J* = 7.9, 7.2, 1.1 Hz, CH_{Ar}), 7.24 (3H, m, CH_{Ar}), 7.09 (2H, m, CH_{Ar}), 6.98 (1H, m, CH_{Ar}), 3.80 (4H, m, C*H*H + OMe), 3.65 (1H, m, CH*H*), 2.18 (3H, s, Me); ^{13}C{1H} NMR (d_6-acetone, 75 MHz, 25 °C): δ = 160.1 (C=O), 150.3 (C_{Ar}), 147.3 (C_{Ar}), 135.3 (C=C), 132.6 (CH_{Ar}), 131.6 (CH_{Ar}), 128.4 ($2CH_{Ar}$), 126.9 (CH_{Ar}), 126.8 (CH_{Ar}), 125.8 (C_{Ar}),

125.1 (CH_{Ar}), 124.1 (C_{Ar}), 122.9 (CH_{Ar}), 122.6 ($2CH_{Ar}$), 121.5 (2C, q, *J* = 325.5 Hz, $2CF_3$), 115.3 (CH_{Ar}), 111.4 (C=C), 110.0 (CH_{Ar}), 63.3 (C^-Tf_2), 54.5 (OMe), 26.2 (CH_2), 19.5 (CH_3); ^{19}F NMR (d_6-acetone, 282 MHz, 25 °C): δ = -79.6 (6F, s, 2 x SO_2CF_3); HRMS (ESI-TOF) m/z: $[M]^-$ Calcd for $[C_{27}H_{20}F_6NO_6S_2]^-$ 632.0636; Found 632.0639.

Compound **7c**

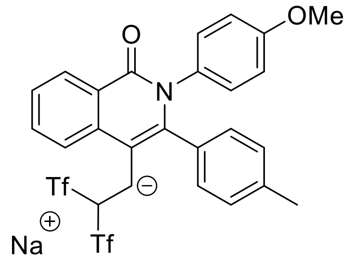

7c

Following the general procedure, starting from 18 mg of 2-ethynylbenzamide **5c**, 27 mg (82%) of compound **7c** were obtained as a yellow thick oil, after purification on column chromatography using hexanes/AcOEt (3:7) as solvent.

^1H NMR (d_6-acetone, 300 MHz, 25 °C): δ = 8.51 (1H, d, *J* = 8.1 Hz, CH_{Ar}), 8.37 (1H, dd, *J* = 7.9, 1.4 Hz, CH_{Ar}), 7.60 (1H, ddd, *J* = 8.1, 7.2, 1.5 Hz, CH_{Ar}), 7.45 (3H, m, CH_{Ar}), 7.24 (4H, m, CH_{Ar}), 6.84 (2H, m, CH_{Ar}), 3.89 (2H, s, C*HH*), 3.77 (3H, s, OMe), 2.37 (3H, s, Me); $^{13}C\{1H\}$ NMR (d_6-acetone, 75 MHz, 25 °C): δ = 156.2 (C=O), 151.9 (C_{Ar}), 149.6 (C_{Ar}), 139.4 (C_{Ar}), 138.9 (C_{Ar}), 138.6 (C_{Ar}), 135.5 (C=C), 131.7 (C_{Ar}), 131.4 (CH_{Ar}), 130.1 ($2CH_{Ar}$), 128.3 ($2CH_{Ar}$), 126.8 (CH_{Ar}), 126.5 (CH_{Ar}), 125.2 ($2CH_{Ar}$), 124.6 ($2CH_{Ar}$), 124.4 (C_{Ar}), 121.3 (2C, q, *J* = 327.8 Hz, $2CF_3$), 113.6 ($2CH_{Ar}$), 110.4 (C=C), 63.3 (C^-Tf_2), 54.6 (OMe), 26.3 (CH_2), 20.4 (CH_3); ^{19}F NMR (d_6-acetone, 282 MHz, 25 °C): δ = -79.6 (6F, s, 2 x SO_2CF_3); HRMS (ESI-TOF) m/z: $[M]^-$ Calcd for $[C_{27}H_{20}F_6NO_6S_2]^-$ 632.0636; Found 632.0640.

Compound **7d**

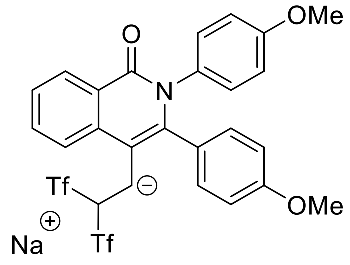

7d

Following the general procedure, starting from 18 mg of 2-ethynylbenzamide **5d**, 26 mg (76%) of compound **7d** were obtained as a yellow thick oil, after purification on column chromatography using hexanes/AcOEt (3:5) as solvent.

^{1}H NMR (d$_6$-acetone, 300 MHz, 25 °C): δ = 8.50 (1H, d, *J* = 8.1 Hz, CH$_{Ar}$), 8.37 (1H, dd, *J* = 7.9, 1.0 Hz, CH$_{Ar}$), 7.61 (1H, ddd, *J* = 8.1, 7.2, 1.5 Hz, CH$_{Ar}$), 7.51 (2H, m, CH$_{Ar}$), 7.43 (1H, m, CH$_{Ar}$), 7.26 (2H, m, CH$_{Ar}$), 6.97 (2H, m, CH$_{Ar}$), 6.86 (2H, m, CH$_{Ar}$), 3.90 (2H, s, C*HH*), 3.85 (3H, s, OMe), 3.77 (3H, s, OMe); ^{13}C{1H} NMR (d$_6$-acetone, 75 MHz, 25 °C): δ = 160.1 (C=O), 156.1 (C$_{Ar}$), 151.8 (C$_{Ar}$), 149.7 (C$_{Ar}$), 139.4 (C$_{Ar}$), 135.5 (C=C), 131.6 (2CH$_{Ar}$), 131.4 (CH$_{Ar}$), 126.8 (C$_{Ar}$), 126.7 (CH$_{Ar}$), 126.5 (CH$_{Ar}$), 125.1 (CH$_{Ar}$), 124.6 (2CH$_{Ar}$), 124.3 (C$_{Ar}$), 121.4 (2C, q, *J* = 327.0 Hz, 2CF$_3$), 113.6 (2CH$_{Ar}$), 113.0 (2CH$_{Ar}$), 110.2 (C=C), 63.5 (C$^-$Tf$_2$), 54.7 (OMe), 54.6 (OMe), 26.3 (CH$_2$); ^{19}F NMR (d$_6$-acetone, 282 MHz, 25 °C): δ = -79.6 (6F, s, 2 x SO$_2$CF$_3$); HRMS (ESI-TOF) m/z: [M]$^-$ Calcd for [C$_{27}$H$_{20}$F$_6$NO$_7$S$_2$]$^-$ 648.0585; Found 648.0585.

Compound **7e**

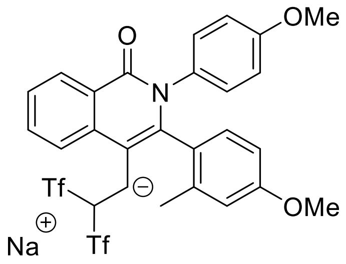

7e

Following the general procedure, starting from 19 mg of 2-ethynylbenzamide **5e**, 29 mg (83%) of compound **7e** were obtained as a yellow thick oil, after purification on column chromatography using hexanes/AcOEt (3:7) as solvent.

^{1}H NMR (d$_6$-acetone, 300 MHz, 25 °C): δ = 8.55 (1H, d, *J* = 7.8 Hz, CH$_{Ar}$), 8.37 (1H, dd, *J* = 7.9, 1.0 Hz, CH$_{Ar}$), 7.61 (1H, ddd, *J* = 8.1, 7.2, 1.5 Hz, CH$_{Ar}$), 7.44 (1H, ddd, *J* = 7.9, 7.2, 1.2 Hz, CH$_{Ar}$), 7.34 (1H, m, CH$_{Ar}$), 7.18 (2H, m, CH$_{Ar}$), 6.80 (4H, m, CH$_{Ar}$), 3.82 (4H, m, OMe + C*H*H), 3.75 (3H, s, OMe), 3.60 (1H, m, C*H*H), 2.22 (3H, s, Me); ^{13}C{1H} NMR (d$_6$-acetone, 75 MHz, 25 °C): δ = 160.1 (C=O), 156.0 (C$_{Ar}$), 149.6 (C$_{Ar}$), 139.5 (C$_{Ar}$), 135.5 (C=C), 132.6 (CH$_{Ar}$), 131.5 (CH$_{Ar}$), 126.9 (CH$_{Ar}$), 126.6 (CH$_{Ar}$), 125.9 (C$_{Ar}$), 125.1 (CH$_{Ar}$), 124.4 (2CH$_{Ar}$), 121.2 (2C, q, *J* = 327.8 Hz, 2CF$_3$), 115.4 (CH$_{Ar}$), 113.6 (2CH$_{Ar}$), 111.4 (C$_{Ar}$), 110.2 (C=C), 63.3 (C$^-$Tf$_2$), 54.7 (OMe), 54.5 (OMe), 26.6 (CH$_2$), 19.5 (Me); ^{19}F NMR (d$_6$-acetone, 282 MHz, 25 °C): δ = -79.7 (6F, s, 2 x SO$_2$CF$_3$); ^{23}Na NMR (d$_6$-acetone, 132 MHz, 25 °C): δ = -8.57 (Na$^+$); HRMS (ESI-TOF) m/z: [M]$^-$ Calcd for [C$_{28}$H$_{22}$F$_6$NO$_7$S$_2$]$^-$ 662.0742; Found 662.0742.

Compound **8a**

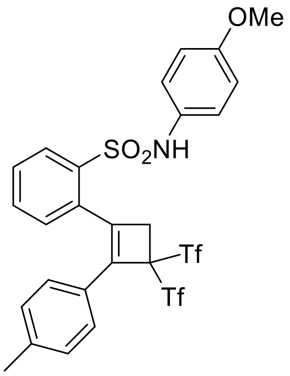

8a

Following the general procedure, starting from 20 mg of 2-ethynylbenzenesulfonamide **6a**, 19 mg (59%) of compound **8a** were obtained as a yellow thick oil, after purification on column chromatography using hexanes/AcOEt (6:1) as solvent.

^{1}H NMR ($CDCl_3$, 300 MHz, 25 °C): δ = 8.14 (1H, m, C_{Ar}), 7.53 (2H, m, C_{Ar}), 7.34 (1H, m, C_{Ar}), 7.13 (1H, s br, NH), 7.05 (2H, d, *J* = 8.3 Hz, $2CH_{Ar}$), 6.84 (2H, d, *J* = 8.3 Hz, $2CH_{Ar}$), 6.61 (2H, d, *J* = 9.1 Hz, $2CH_{Ar}$), 6.46 (2H, d, *J* = 9.1 Hz, $2CH_{Ar}$), 3.99 (2H, s br, CH_2), 3.61 (3H, s, OMe), 2.20 (3H, s, Me); ^{13}C{1H} NMR ($CDCl_3$, 75 MHz, 25 °C): δ = 156.6 (C_{Ar}), 147.5 (C_{Ar}), 140.9 (C_{Ar}), 138.5 (C_{Ar}), 139.1 (C_{Ar}), 133.5 ($2CH_{Ar}$), 131.4 (C=C), 131.2 (CH_{Ar}), 130.8 (CH_{Ar}), 130.4 (CH_{Ar}), 129.3 ($2CH_{Ar}$), 128.9 (C_{Ar}), 127.8 (CH_{Ar}), 125.3 (C=C), 121.1 ($2CH_{Ar}$), 119.8 (2C, q, *J* = 327.8 Hz, $2CF_3$), 114.4 ($2CH_{Ar}$), 86.4 (CTf_2), 55.4 (OMe), 40.0 (CH_2), 21.4 (Me); ^{19}F NMR ($CDCl_3$, 282 MHz, 25 °C): δ = -69.9 (6F, s, 2 x SO_2CF_3); HRMS (ESI-TOF) m/z: [M] Calcd for [$C_{26}H_{21}F_6NO_7S_3$] 669.0384; Found 669.0387.

Synthetic procedure for scale-up reaction of 3-aryl-4-bis(triflyl)ethylated isocoumarin 3a

To a solution of 2-ethynylbenzoate **2a** (320 mg, 1.2 mmol) in acetonitrile (24 mL) was added zwitterion **1** (467 mg, 1.2 mmol). The reaction mixture was stirred at 40 °C for 3 hours. The resulting mixture was concentrated in vacuo and the crude residue was purified using silica gel column flash chromatography eluting with hexanes/ethyl acetate (2:3) to get the target product as a colorless oil (607 mg, 93% yield; which is comparable to the experiment on a lesser scale in Scheme 2).

Compound **8b**

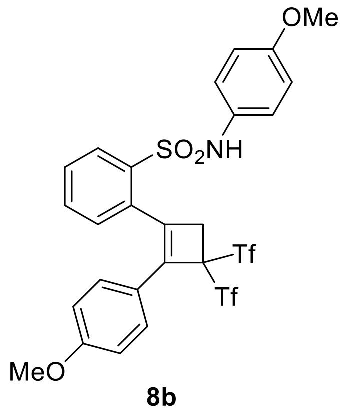

8b

Following the general procedure, starting from 20 mg of 2-ethynylbenzenesulfonamide **6b**, 20 mg (61%) of compound **8b** were obtained as a colorless thick oil, after purification on column chromatography using hexanes/AcOEt (3:1) as solvent.

^{1}H NMR ($CDCl_3$, 300 MHz, 25 °C): δ = 8.23 (1H, m, C_{Ar}), 7.61 (2H, m, C_{Ar}), 7.41 (1H, m, C_{Ar}), 7.25 (1H, s br, NH), 7.17 (2H, d, J = 8.9 Hz, $2CH_{Ar}$), 6.69 (2H, d, J = 9.0 Hz, $2CH_{Ar}$), 6.59 (2H, d, J = 8.9 Hz, $2CH_{Ar}$), 6.53 (2H, d, J = 9.0 Hz, $2CH_{Ar}$), 4.04 (2H, s br, CH_2), 3.74 (3H, s, OMe), 3.67 (3H, s, OMe); ^{13}C{1H} NMR ($CDCl_3$, 75 MHz, 25 °C): δ = 161.1 (C_{Ar}), 156.5 (C_{Ar}), 145.3 (C_{Ar}), 138.5 (C_{Ar}), 135.5 (C_{Ar}), 133.5 (CH_{Ar}), 131.5 (C=C), 131.3 (CH_{Ar}), 130.7 (CH_{Ar}), 130.4 (CH_{Ar}), 129.5 ($2CH_{Ar}$), 129.0 (C_{Ar}), 120.8 ($2CH_{Ar}$), 120.6 (C=C), 119.8 (2C, q, J = 329.3 Hz, $2CF_3$), 114.4 ($2CH_{Ar}$), 114.0 ($2CH_{Ar}$), 86.3 (CTf_2), 55.4 (OMe), 55.3 (OMe), 40.4 (CH_2); ^{19}F NMR ($CDCl_3$, 282 MHz, 25 °C): δ = -70.0 (6F, s, 2 x SO_2CF_3); IR (CH_2Cl_2): ν = 2974, 1700, 1206 cm^{-1}; HRMS (ESI-TOF) m/z: [M] Calcd for [$C_{26}H_{21}F_6NO_8S_3$] 685.0333; Found 685.0337.

References

[1] Yanai, H.; Kawazoe, T.; Ishii, N.; Witulski, B.; Takashi, M. Regioselective Synthesis of 4-Aryl-1,3-dihydroxy-2-naphthoates through 1,2-Aryl-Migrative Ring Rearrangement Reaction and their Photoluminiscence Properties. *Chem. Eur. J.* **2021**, *27*, 11442-11449.

[2] Norseeda, K.; Chaisan, N.; Thongsornkleeb, C.; Tummatorn, J.; Somsak, R. Metal-Free Synthesis of 4-Chloroisocoumarins by TMSCl-Catalyzed NCS-Induced Chlorinative Annulation of 2-Alkynylaryloate Esters. *J. Org. Chem.* **2019**, *84*, 16222-16236.

[3] Speranca, A.; Godoi, B.; Pinton, S.; Back, D.; Menezes, P.; Zeni, G. Regioselective synthesis of isochromenones by iron(III)/PhSeSePh-mediated cyclization of 2-alkynylaryl esters. *J. Org. Chem.* **2011**, *76*, 6789-6797.

[4] Roy, S.; Roy, S.; Neuenswander, B.; Hill, D.; Larock, R. C. Solution-Phase Synthesis of a Diverse Isocoumarin Library. *J. Comb. Chem.* **2009**, *11*, 1128-1135.

[5] Curti, F.; Tiecco, M.; Pirovano, V.; Germani, R.; Caselli, A.; Rossi, E.; Abbiati, G. *p*-TSA-Based DESs as "Active Green Solvents" for Microwave Enhanced Cyclization of 2-Alkynyl-(hetero)-arylcarboxylates: an Alternative Access to 6-Substituted 3,4-Fused 2-Pyranones. *Eur. J. Org. Chem*. **2019**, 1904-1914.

[6] Mehta, S.; Brahmchari, D. Phosphazene Superbase-Mediated Regio- and Stereoselective Iodoaminocyclization of 2-(1-Alkynyl)benzamides for the Synthesis of Isoindolin-1-ones. *J. Org. Chem*. **2019**, *84*, 5492-5503.

[7] Yao, B.; Jaccoud, C.; Wang, Q.; Zhu, J. Synergistic Effect of Palladium and Copper Catalysts: Catalytic Cyclizative Dimerization of ortho-(1-Alkynyl)benzamides Leading to Axially Chiral 1,3-Butadienes. *Chem. Eur. J*. **2012**, *18*, 5864-5868.

[8] Tian, M.; Bai, D.; Zheng, G.; Chang, J.; Li, X. Rh(III)-Catalyzed Asymmetric Synthesis of Axially Chiral Biindolyls by Merging C-H Activation and Nucleophilic Cyclization. *J. Am. Chem. Soc*. **2019**, *141*, 9527-9532.

^{1}H NMR compound **2f** ($CDCl_3$, 300 MHz, 25 °C)

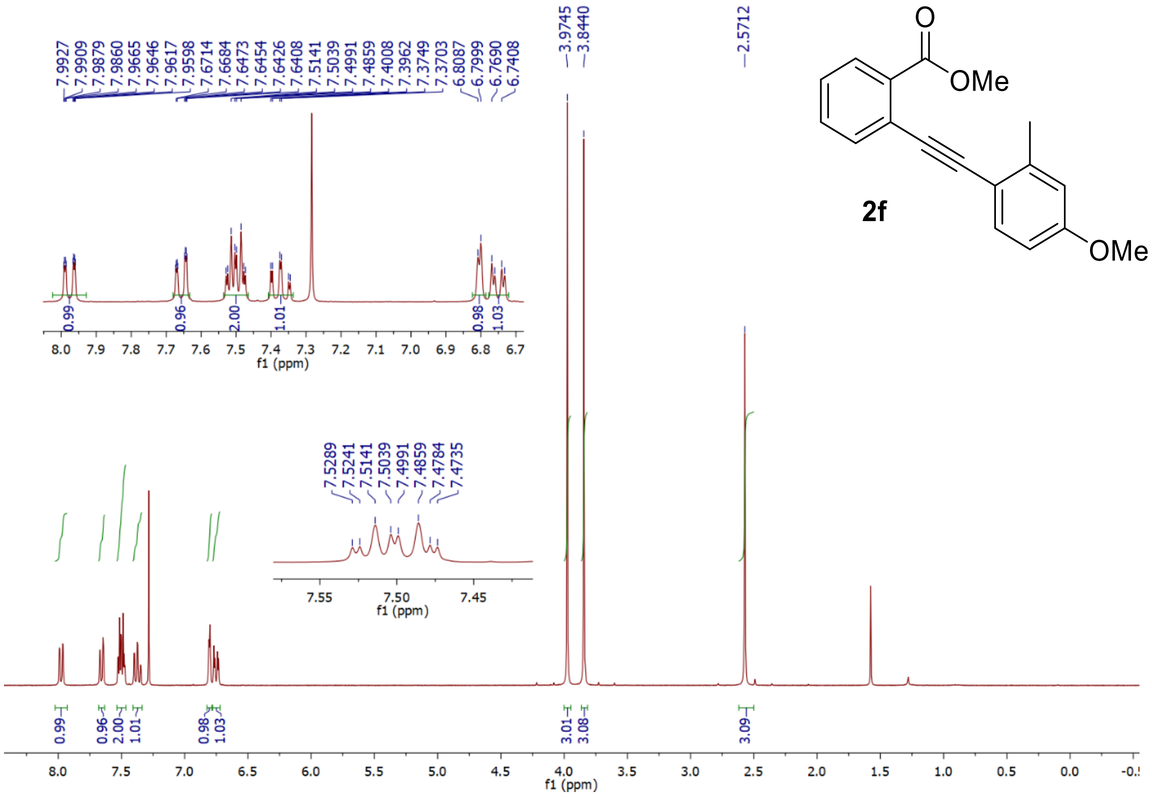

^{13}C NMR compound **2f** ($CDCl_3$, 75 MHz, 25 °C)

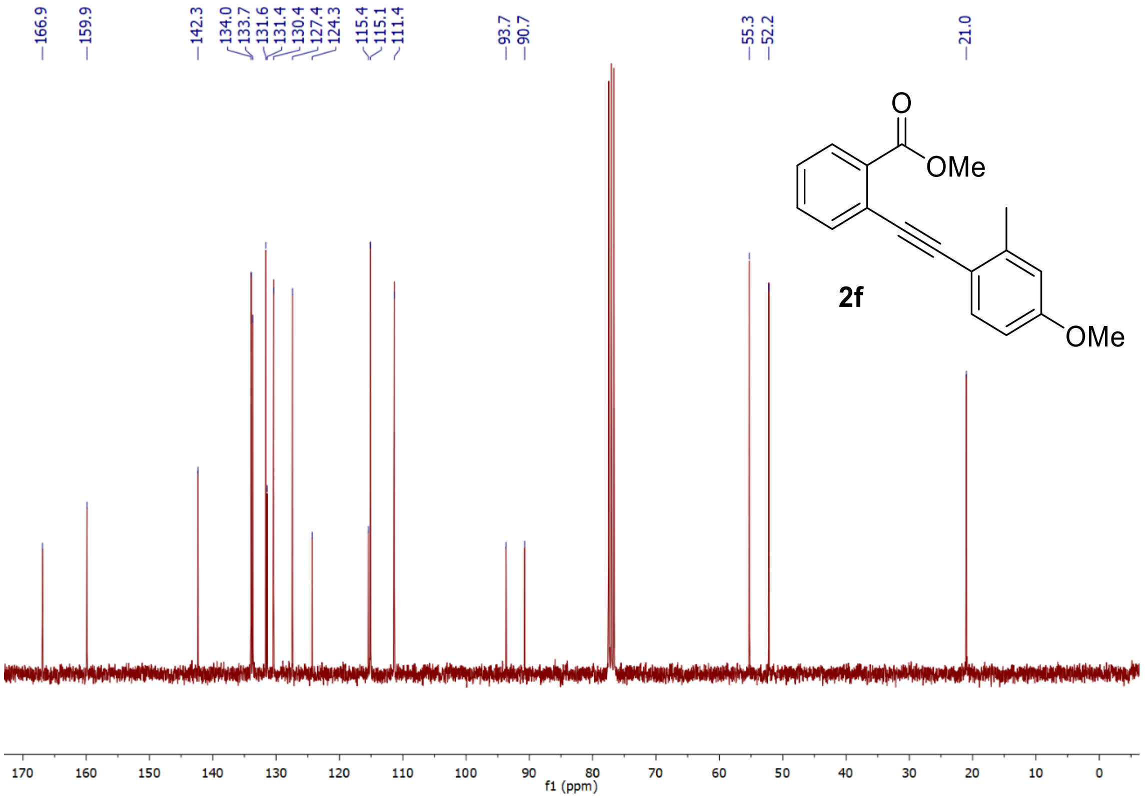

^{1}H NMR compound **2g** ($CDCl_3$, 300 MHz, 25 °C)

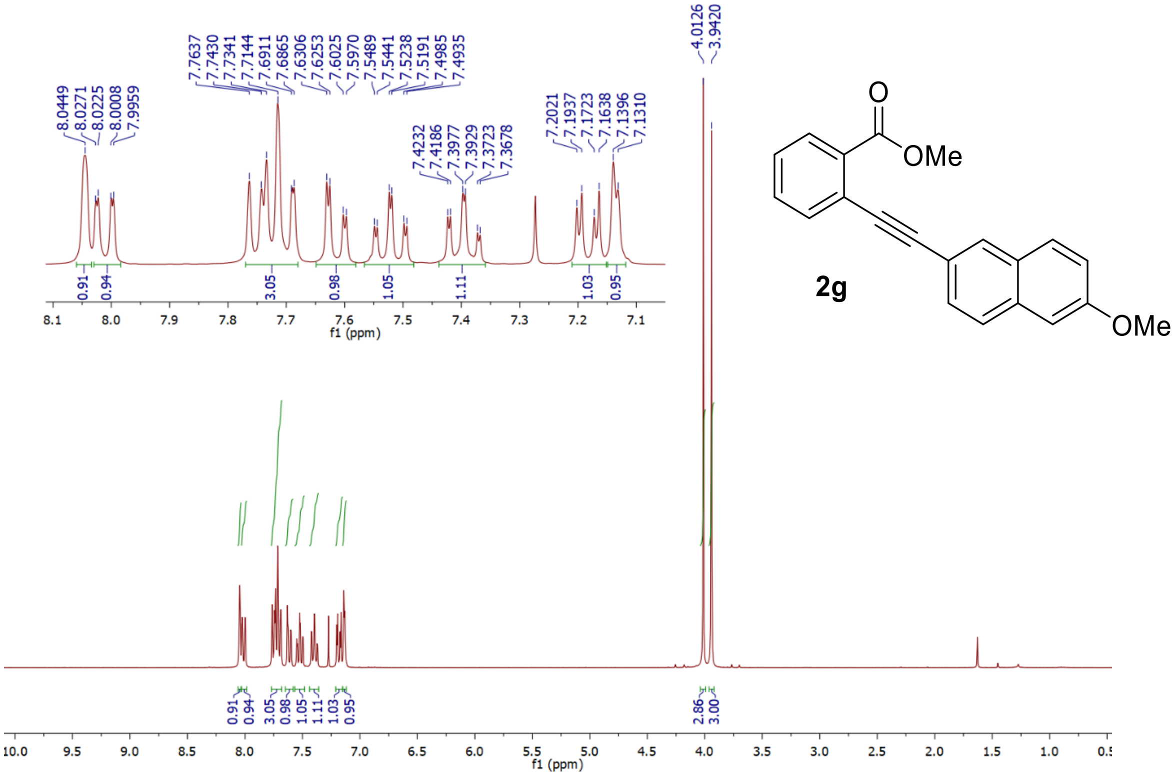

^{13}C NMR compound **2g** ($CDCl_3$, 75 MHz, 25 °C)

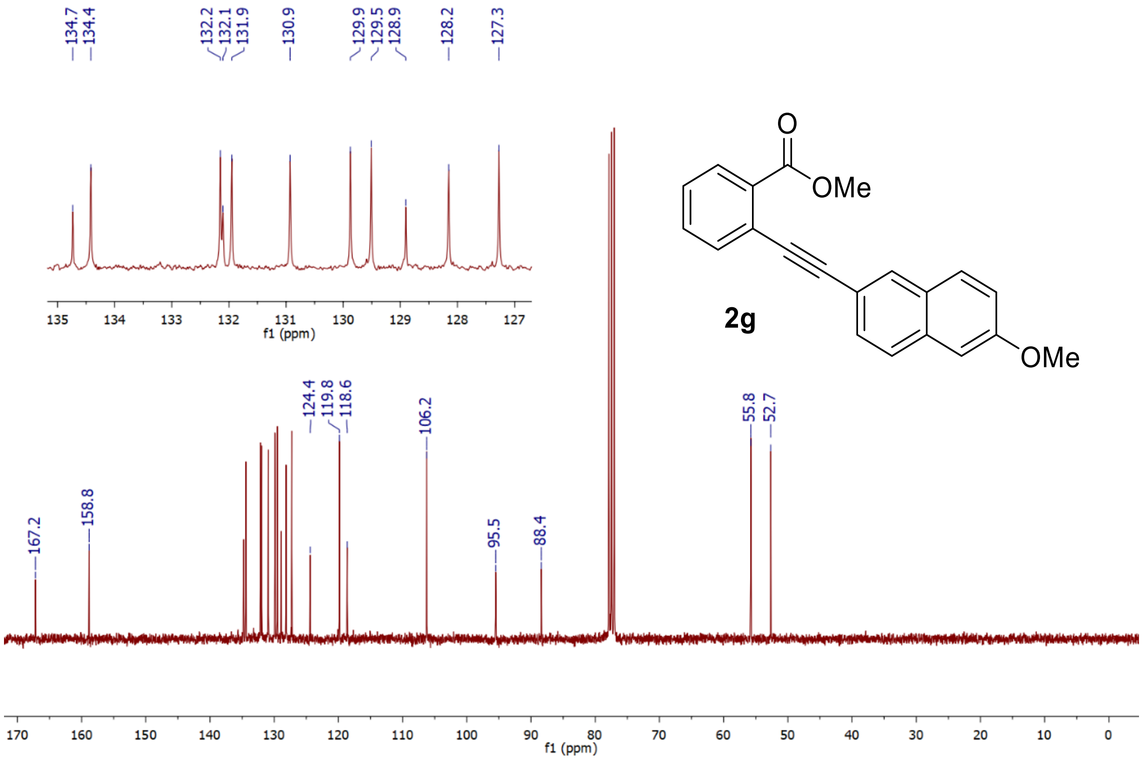

^{1}H NMR compound **2j** ($CDCl_3$, 300 MHz, 25 °C)

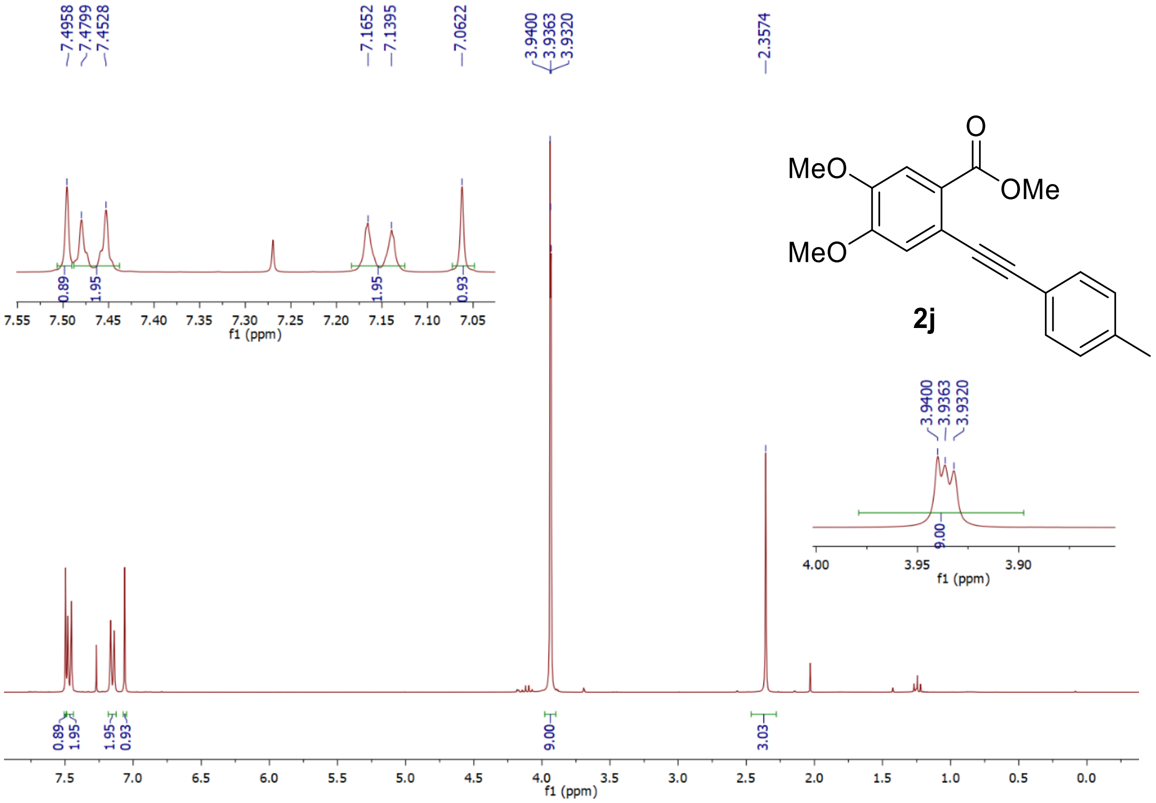

^{13}C NMR compound **2j** ($CDCl_3$, 75 MHz, 25 °C)

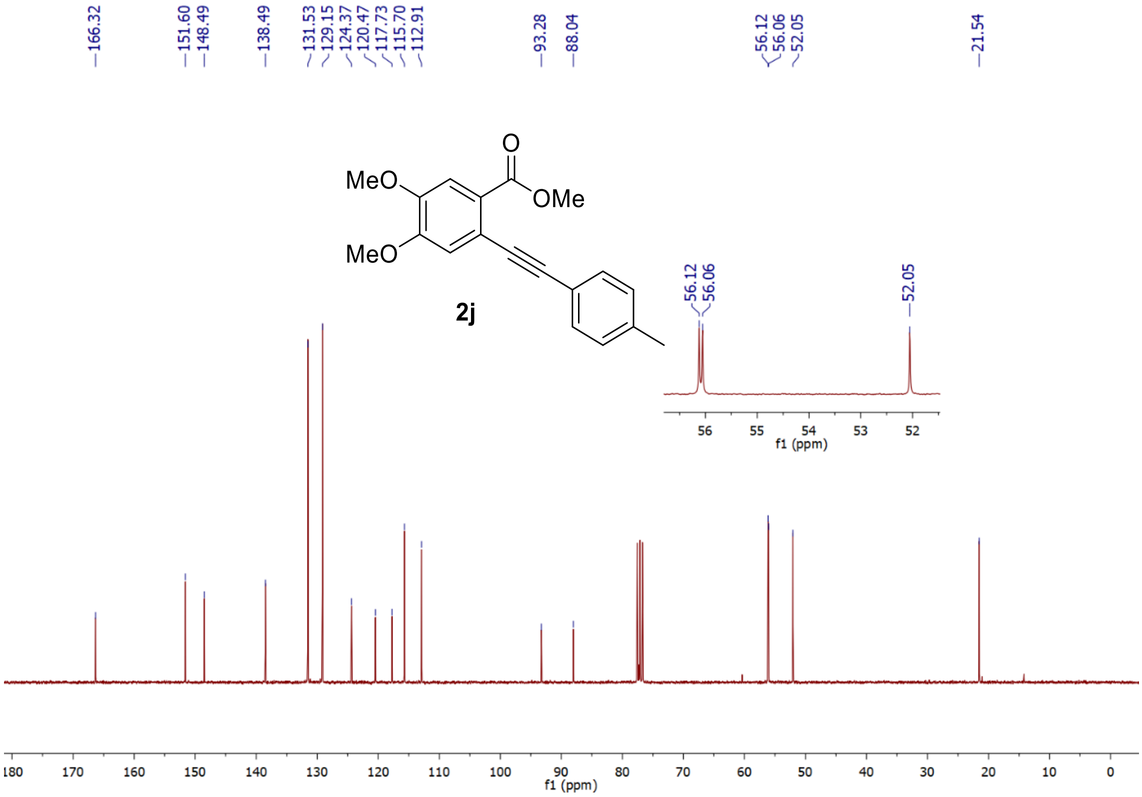

^{1}H NMR compound **2k** ($CDCl_3$, 300 MHz, 25 °C)

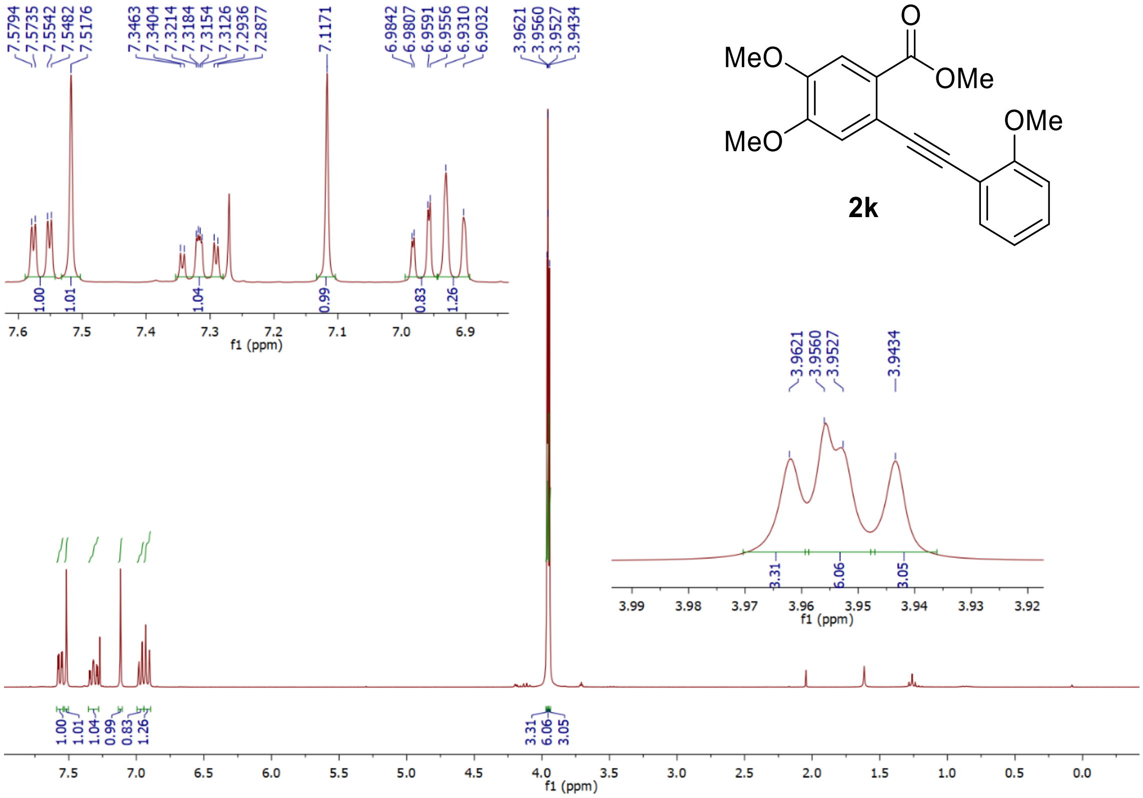

^{13}C NMR compound **2k** ($CDCl_3$, 75 MHz, 25 °C)

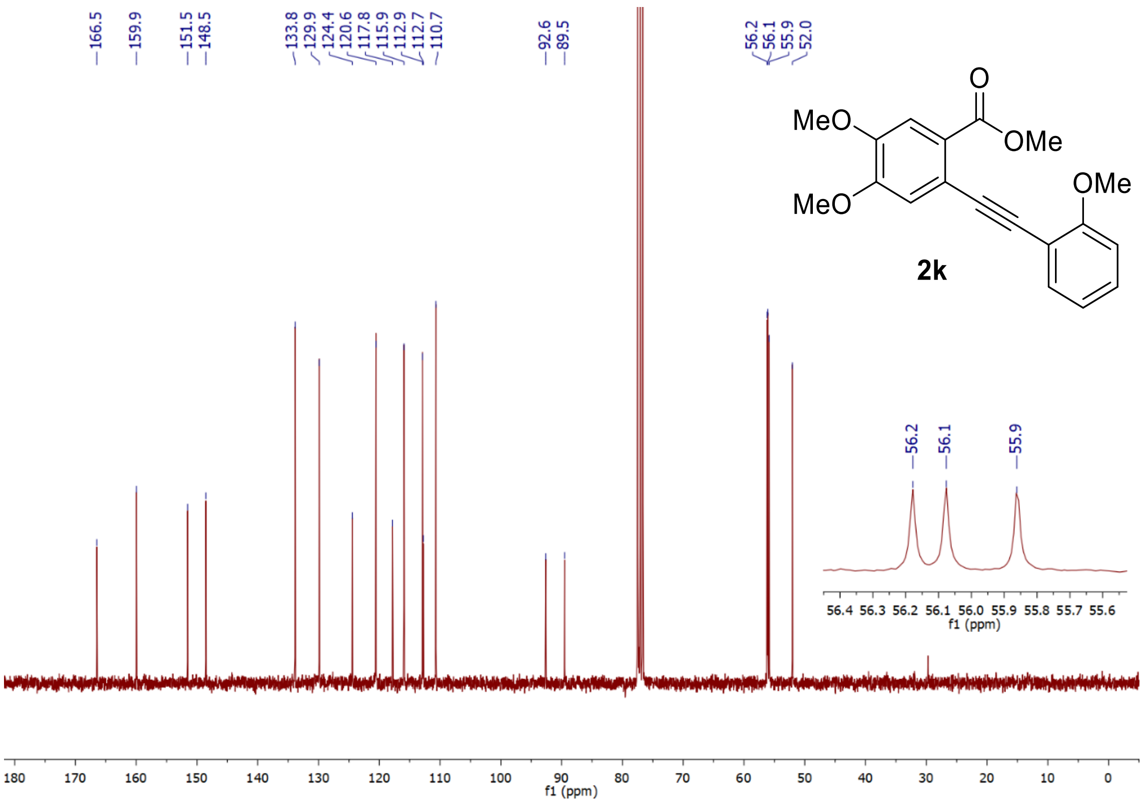

^{1}H NMR compound **2l** ($CDCl_3$, 300 MHz, 25 °C)

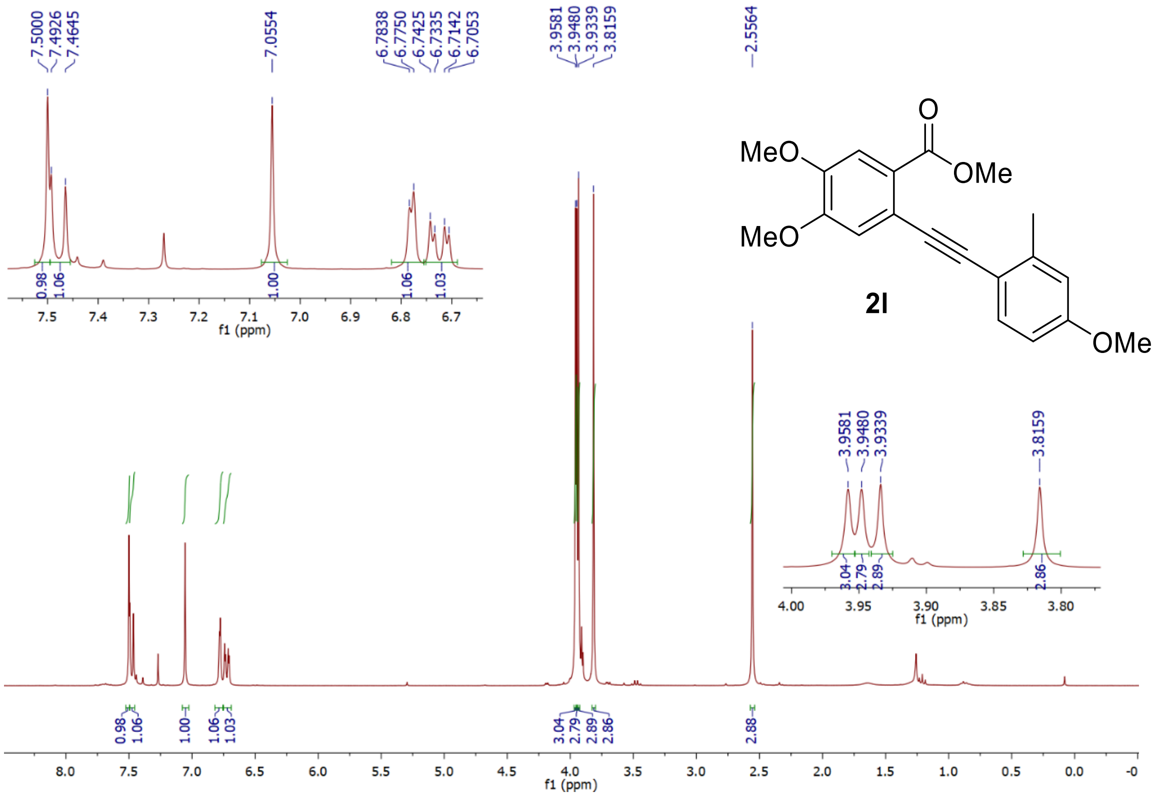

^{13}C NMR compound **2l** ($CDCl_3$, 75 MHz, 25 °C)

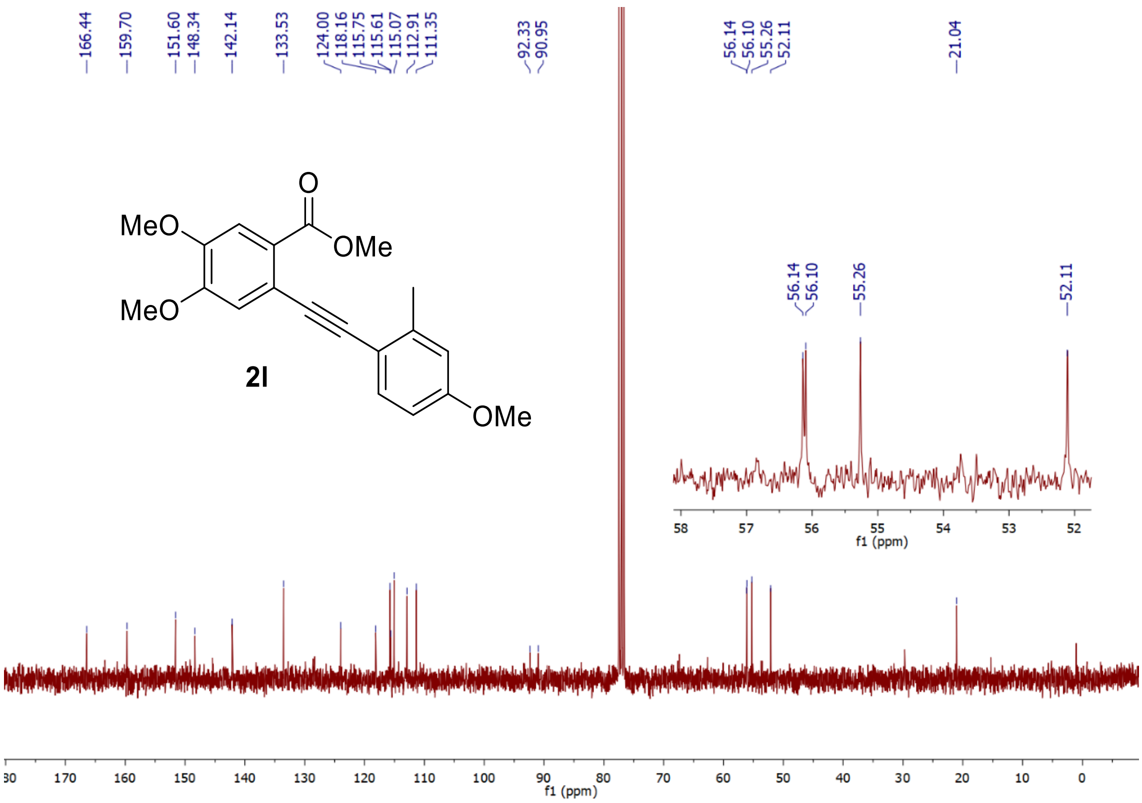

^{1}H NMR compound **2m** ($CDCl_3$, 300 MHz, 25 °C)

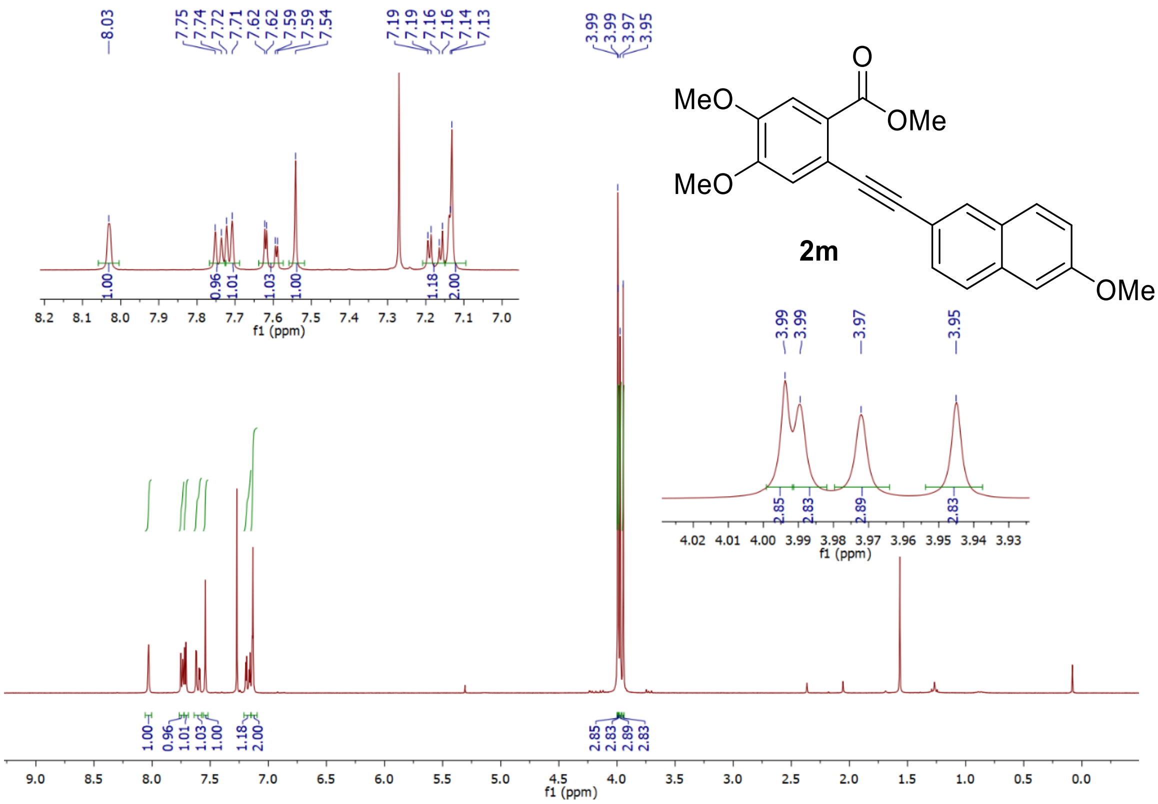

^{13}C NMR compound **2m** ($CDCl_3$, 75 MHz, 25 °C)

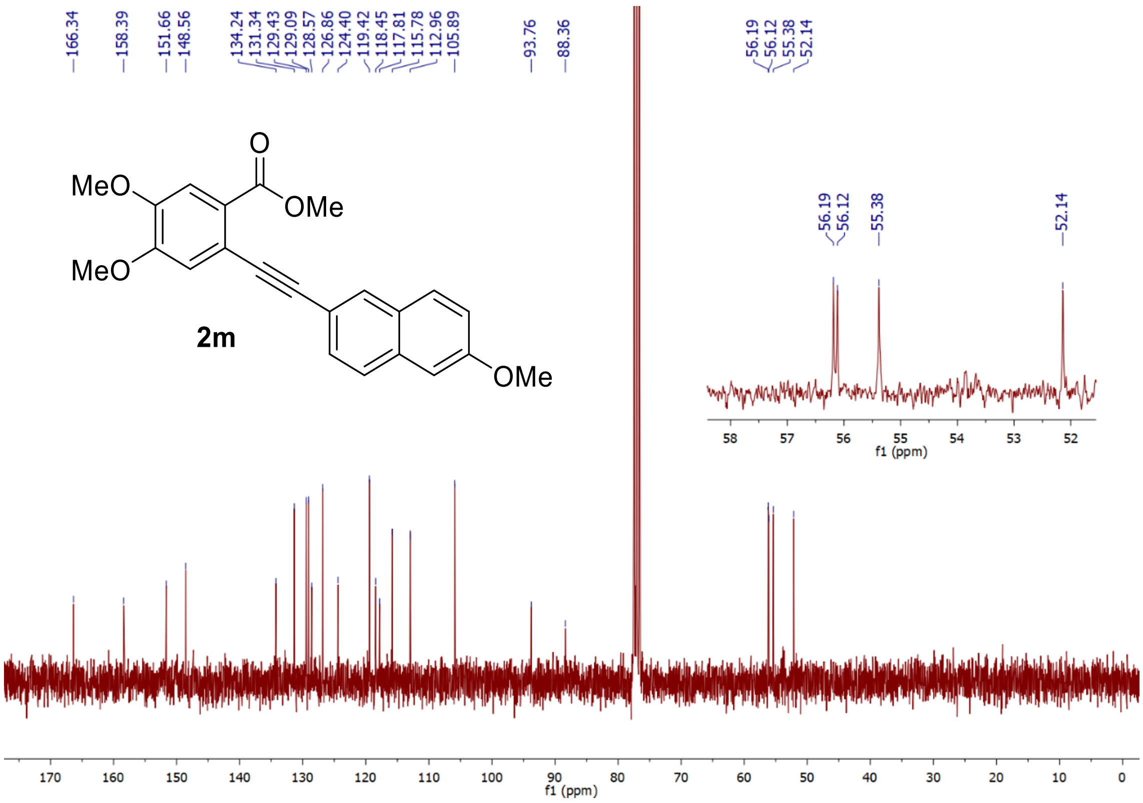

^{1}H NMR compound **2n** ($CDCl_3$, 300 MHz, 25 °C)

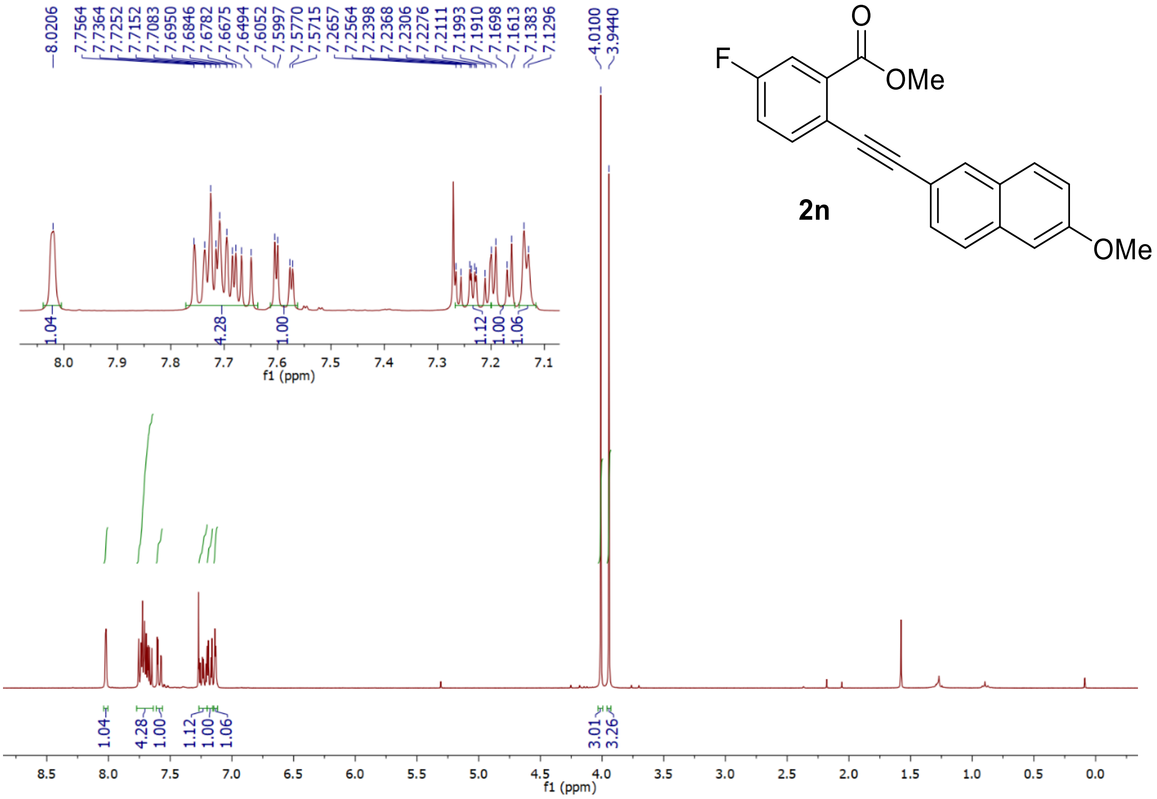

^{13}C NMR compound **2n** ($CDCl_3$, 75 MHz, 25 °C)

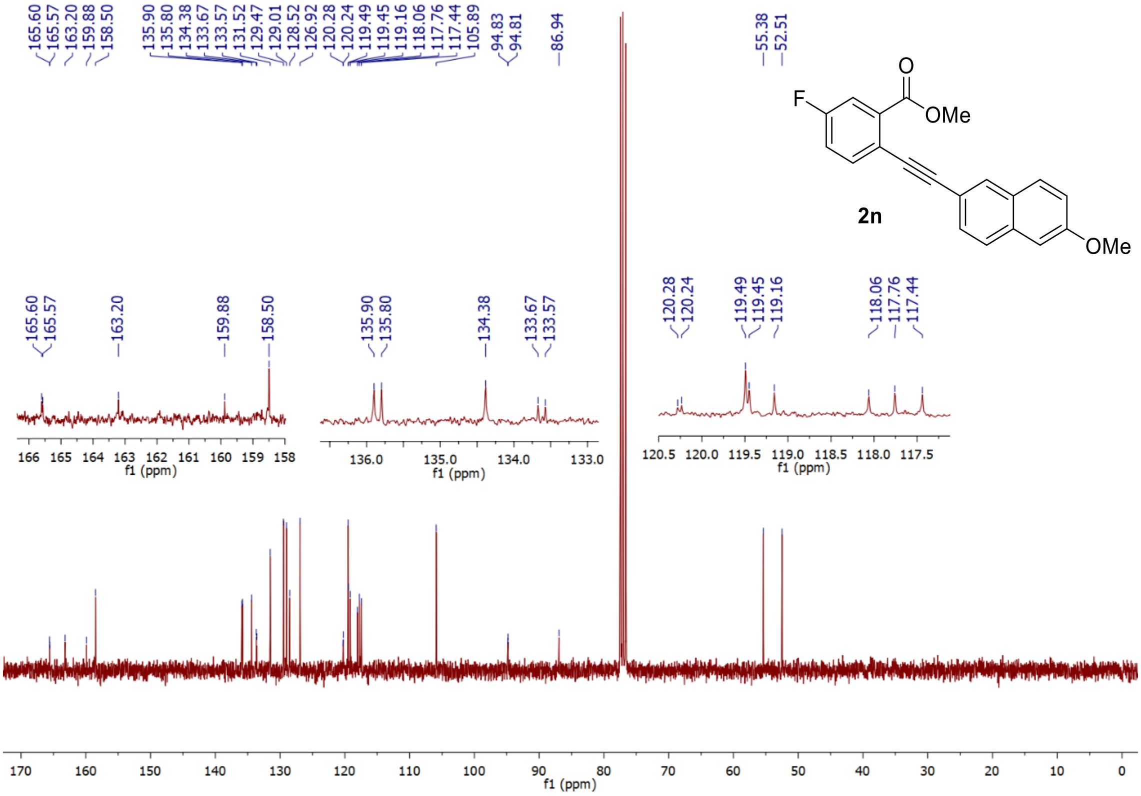

^{19}F NMR compound **2n** ($CDCl_3$, 282 MHz, 25 °C)

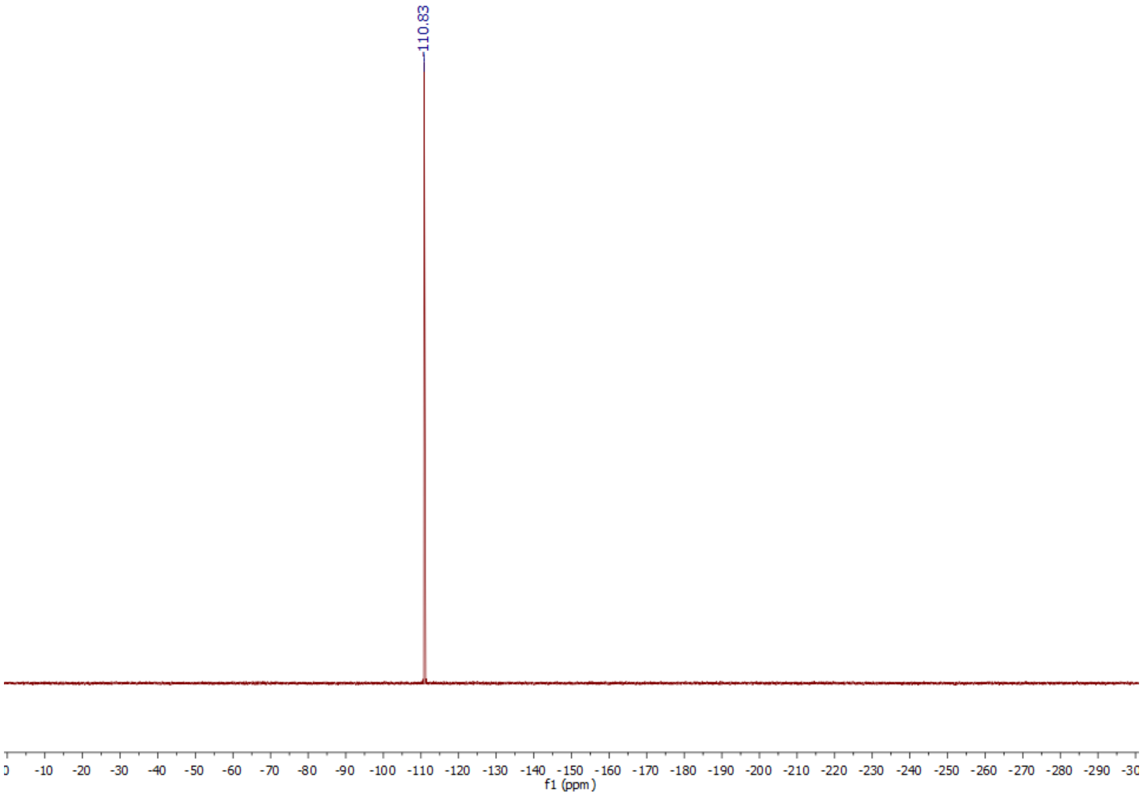

^1H NMR compound **2p** ($CDCl_3$, 300 MHz, 25 °C)

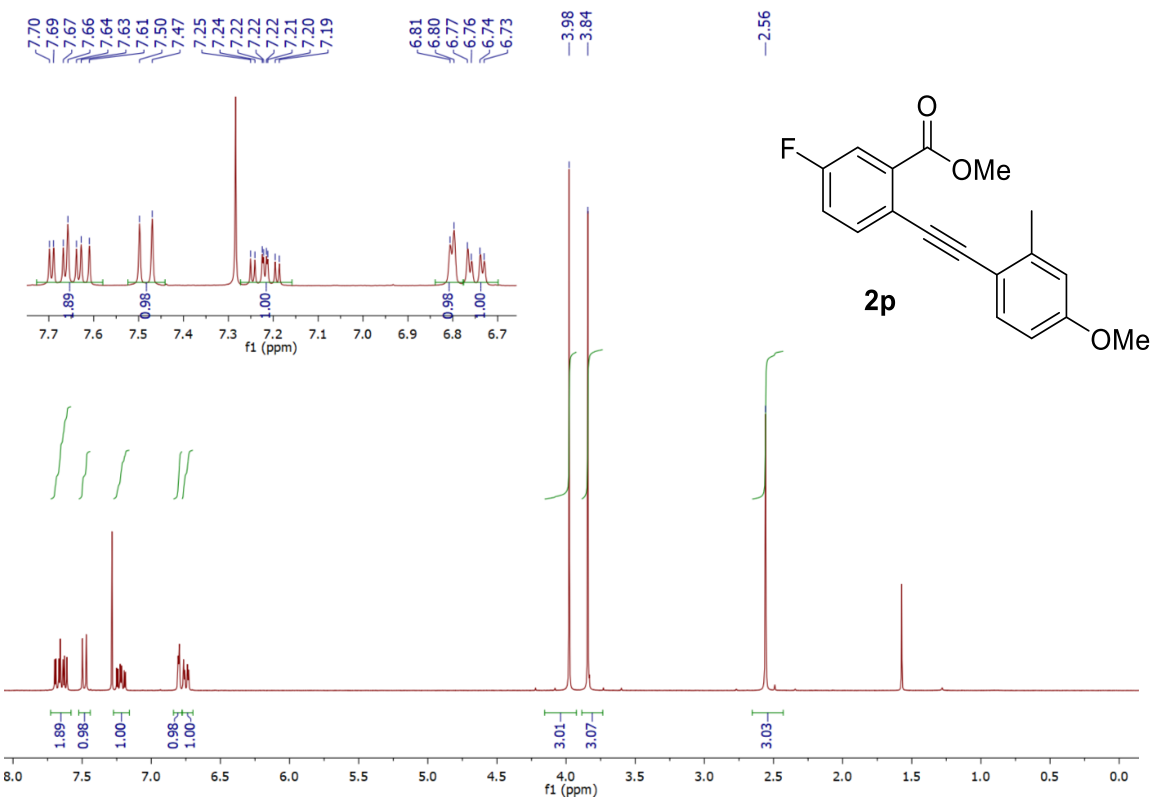

^{13}C NMR compound **2p** ($CDCl_3$, 75 MHz, 25 °C)

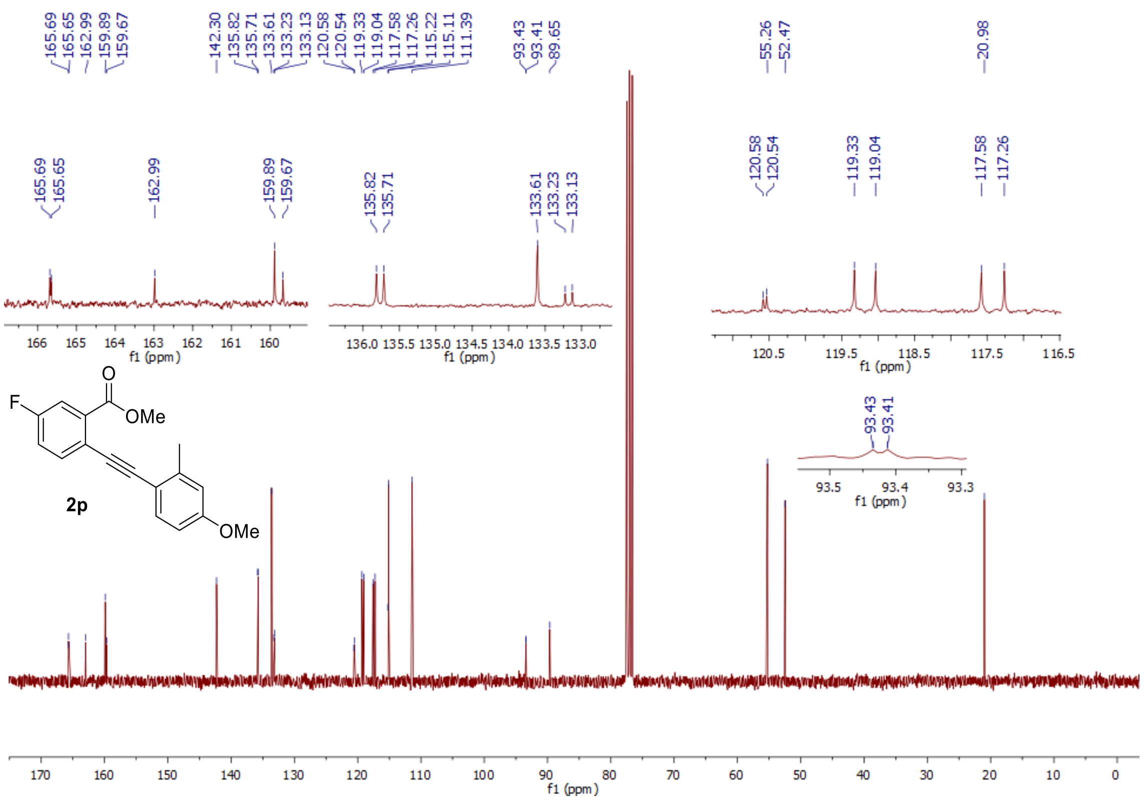

^{19}F NMR compound **2p** ($CDCl_3$, 282 MHz, 25 ^{o}C)

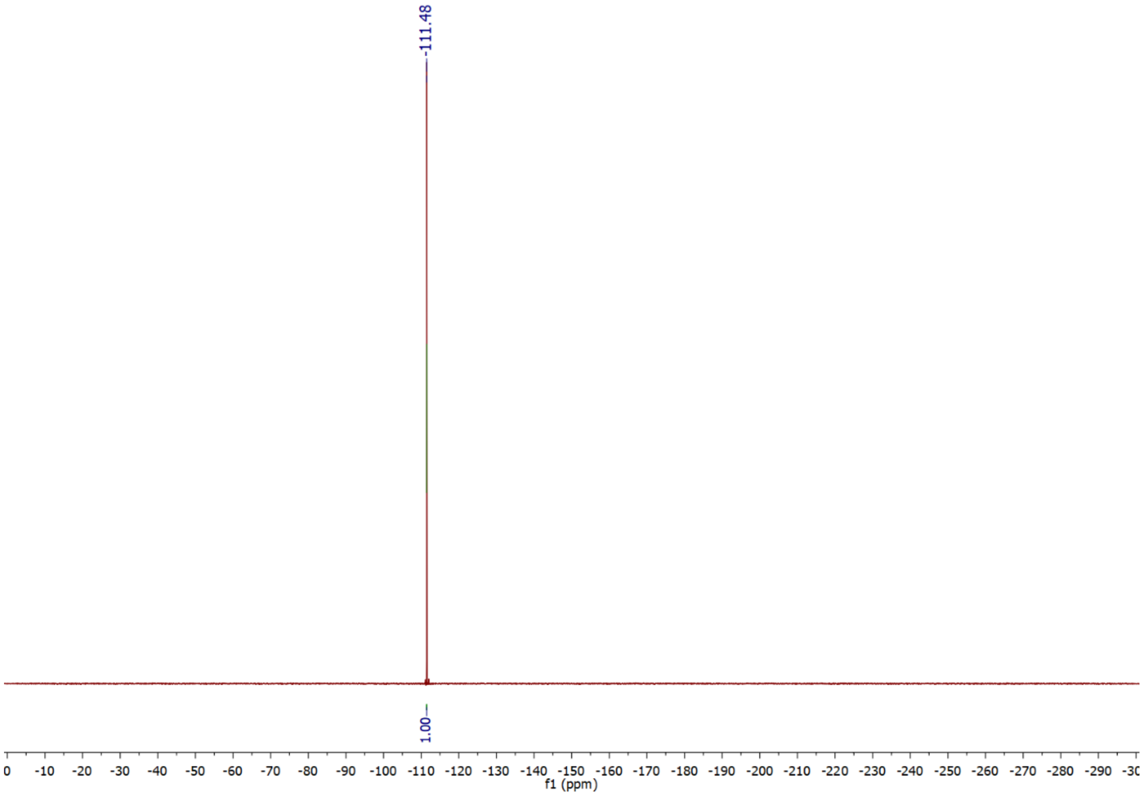

^{1}H NMR compound **2r** ($CDCl_3$, 300 MHz, 25 °C)

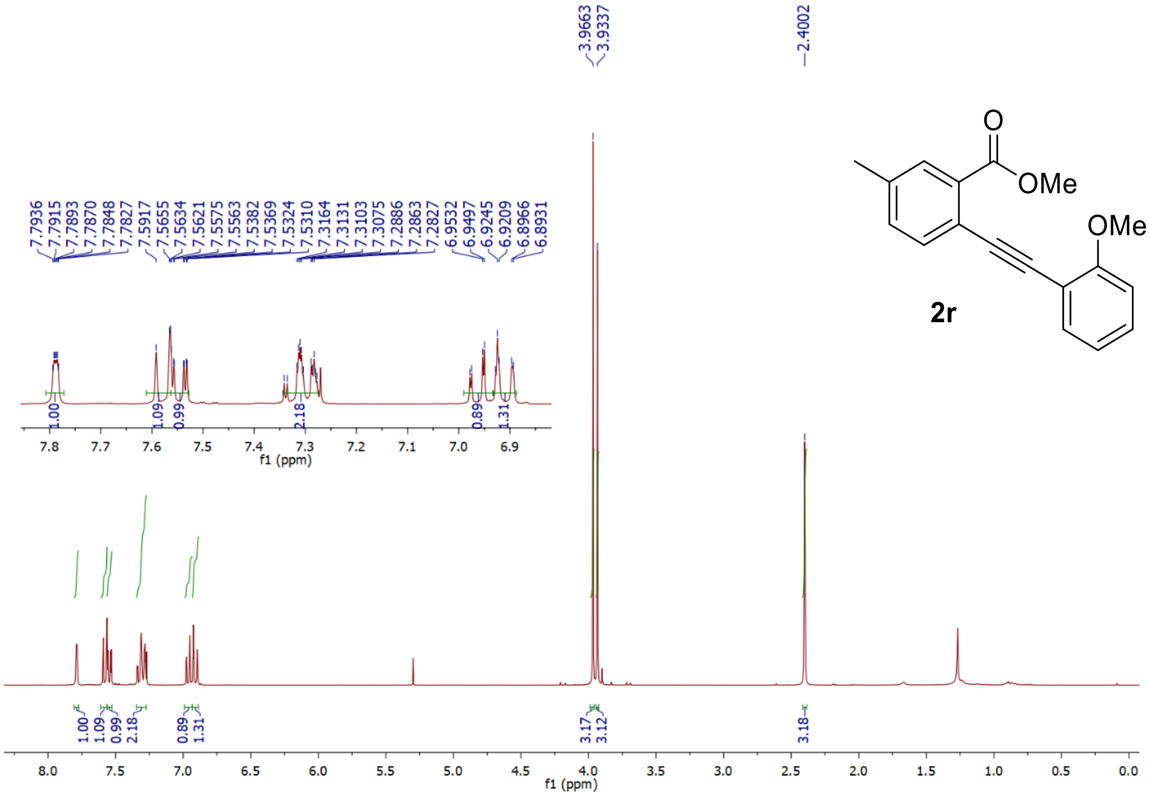

^{13}C NMR compound **2r** ($CDCl_3$, 75 MHz, 25 °C)

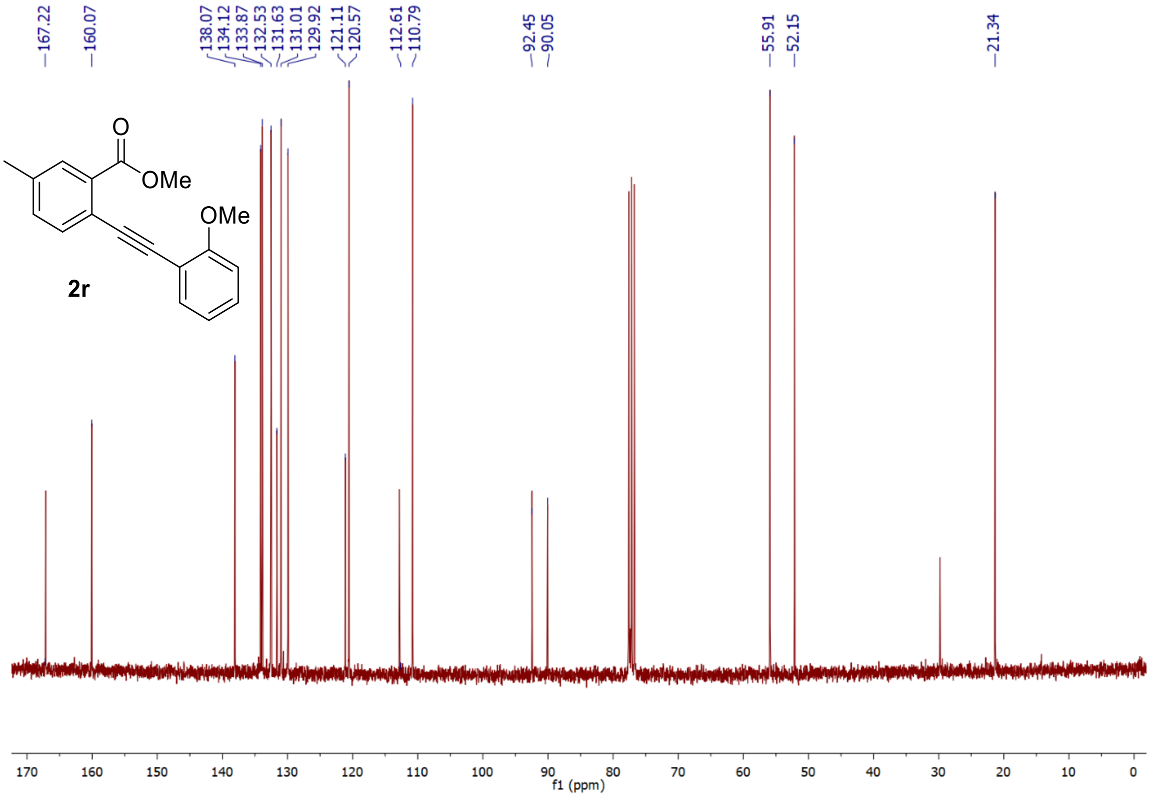

^{1}H NMR compound **2s-*p*-CF₃** (CDCl₃, 300 MHz, 25 °C)

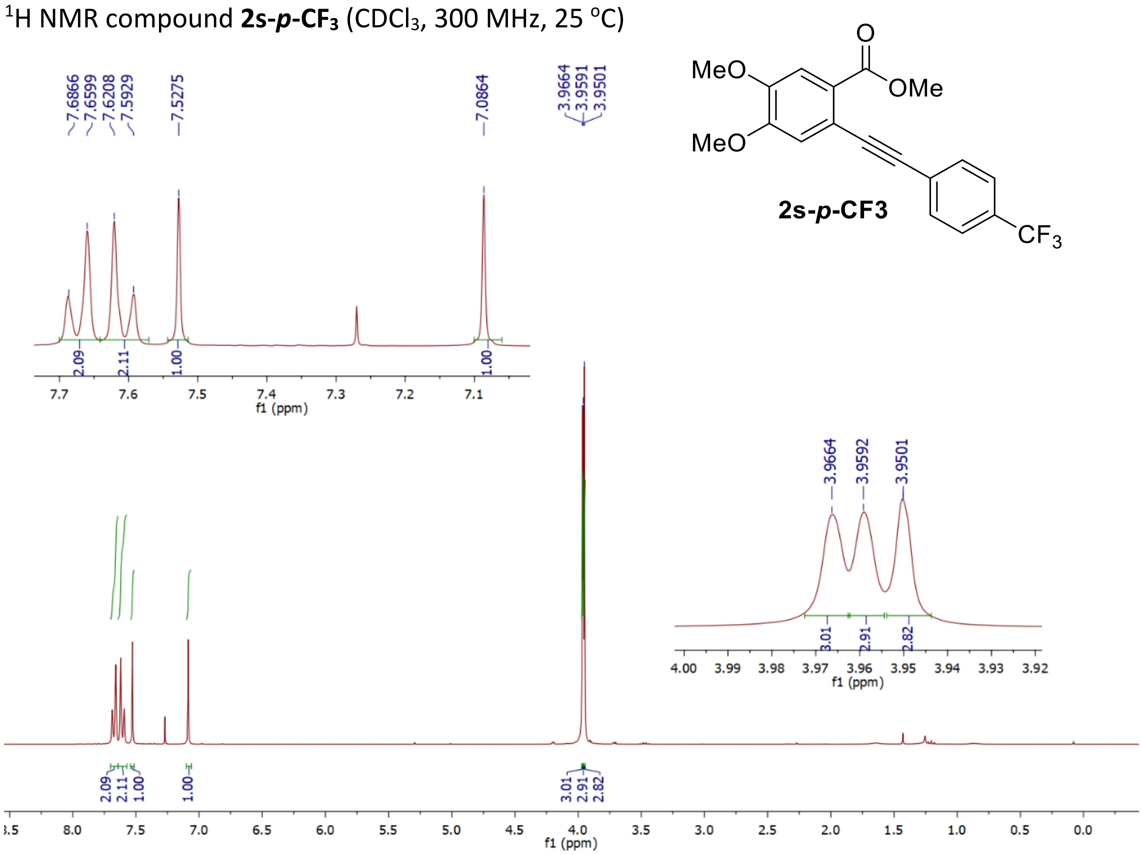

^{13}C NMR compound **2s-*p*-CF₃** (CDCl₃, 75 MHz, 25 °C)

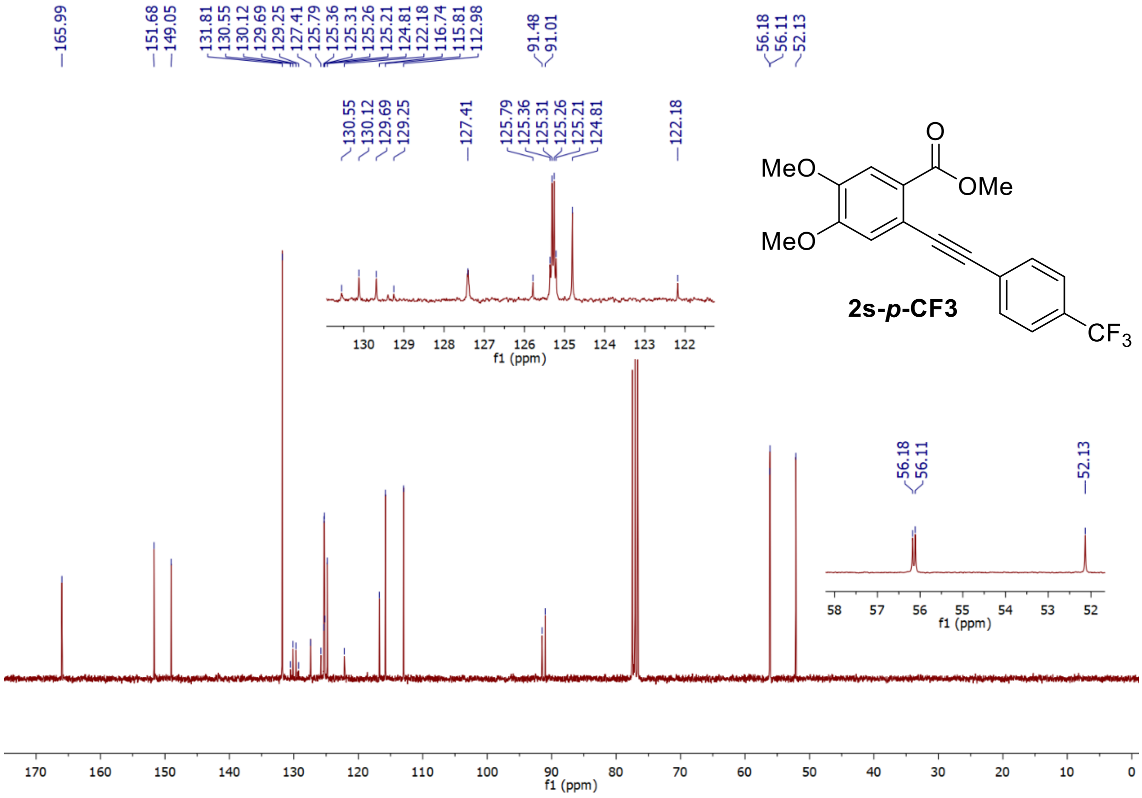

^{19}F NMR compound **2s-*p*-CF$_3$** ($CDCl_3$, 282 MHz, 25 °C)

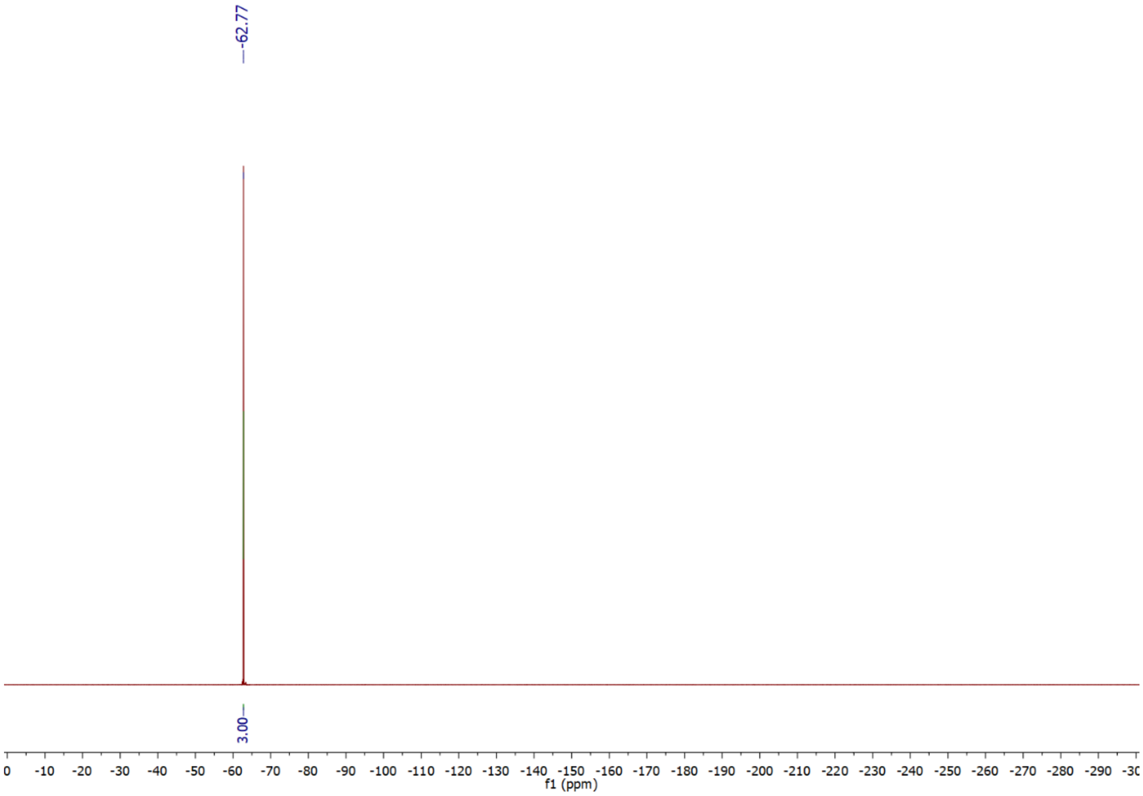

^{1}H NMR compound **2s-*o*-CF$_3$** ($CDCl_3$, 300 MHz, 25 °C)

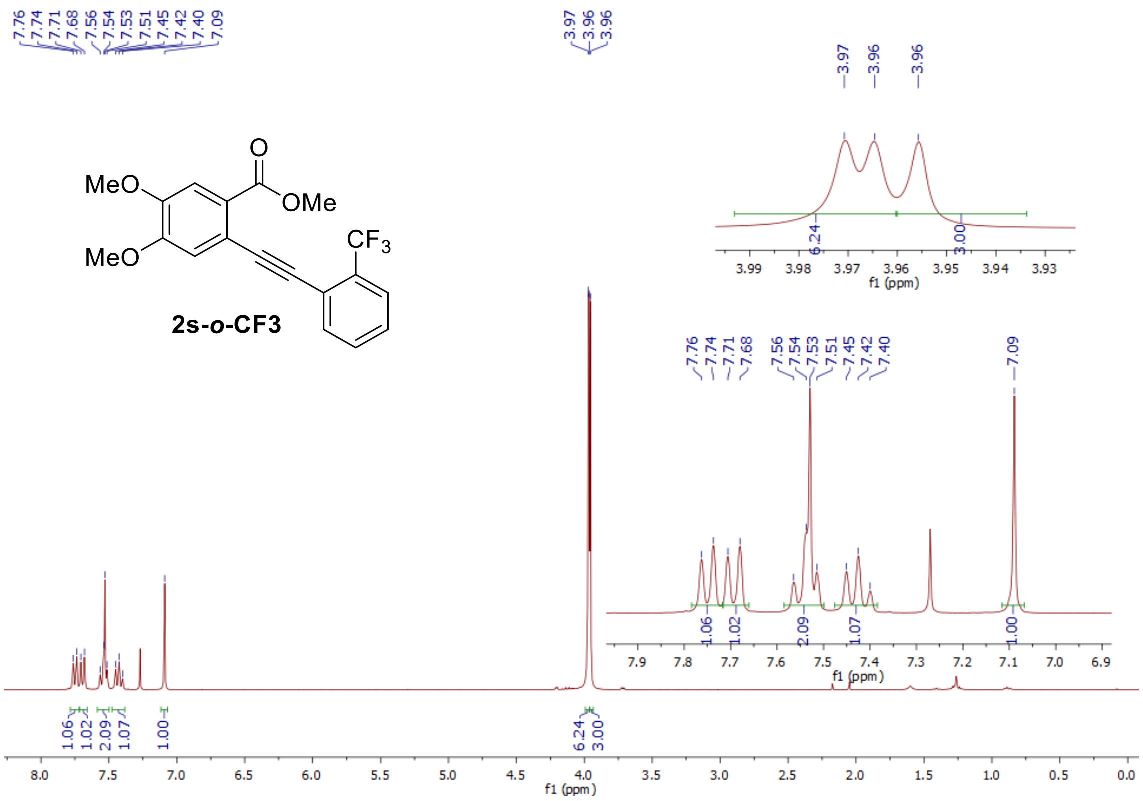

^{13}C NMR compound **2s-*o*-CF$_3$** ($CDCl_3$, 75 MHz, 25 °C)

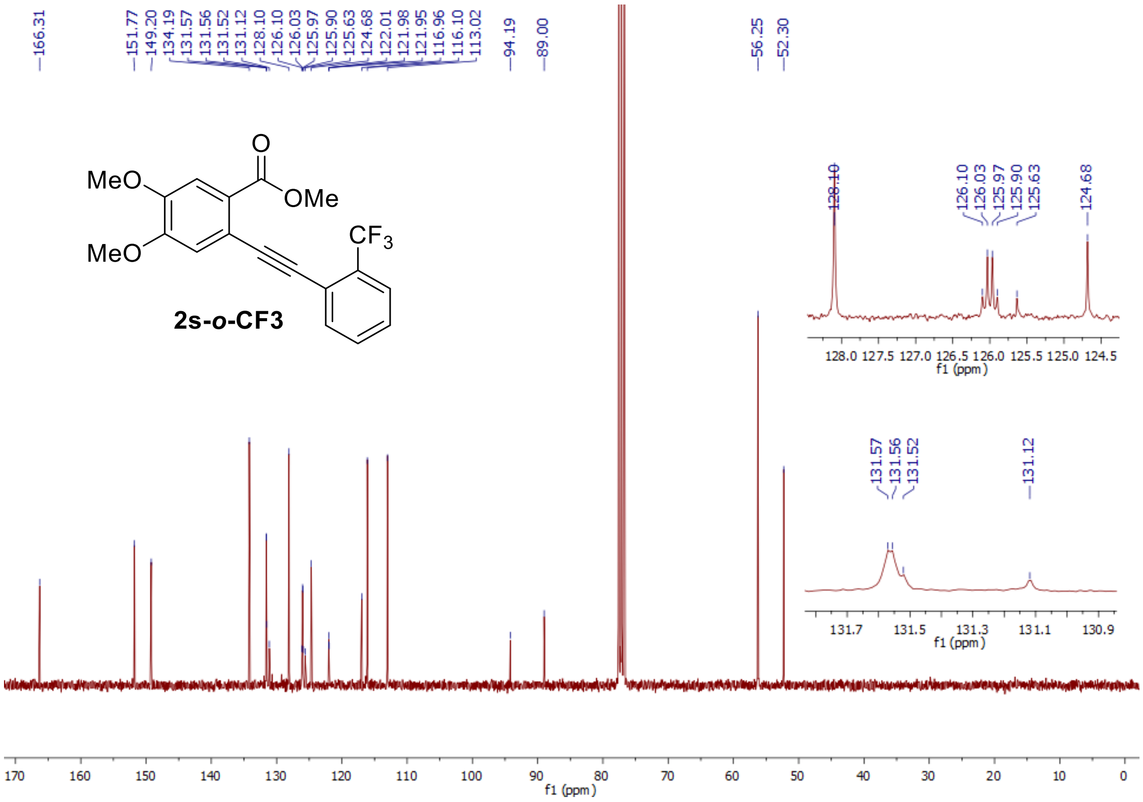

^{19}F NMR compound **2s-*o*-CF$_3$** ($CDCl_3$, 282 MHz, 25 °C)

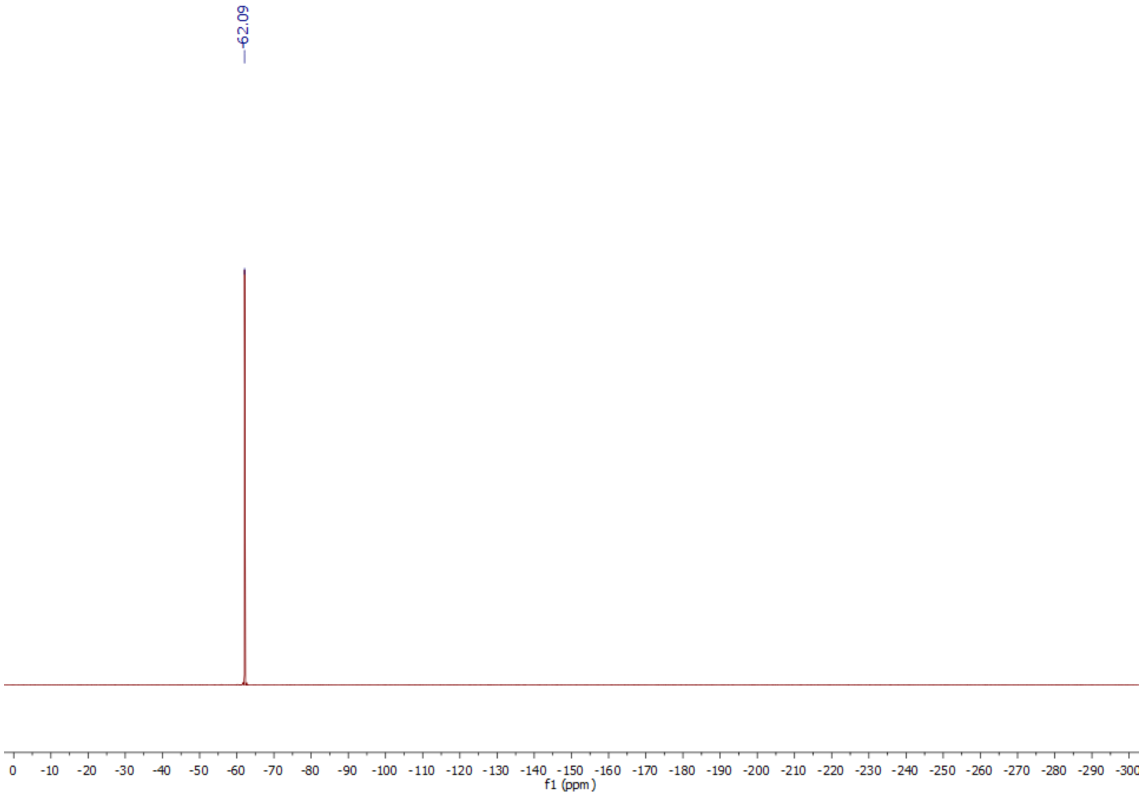

^{1}H NMR compound **5b** ($CDCl_3$, 300 MHz, 25 °C)

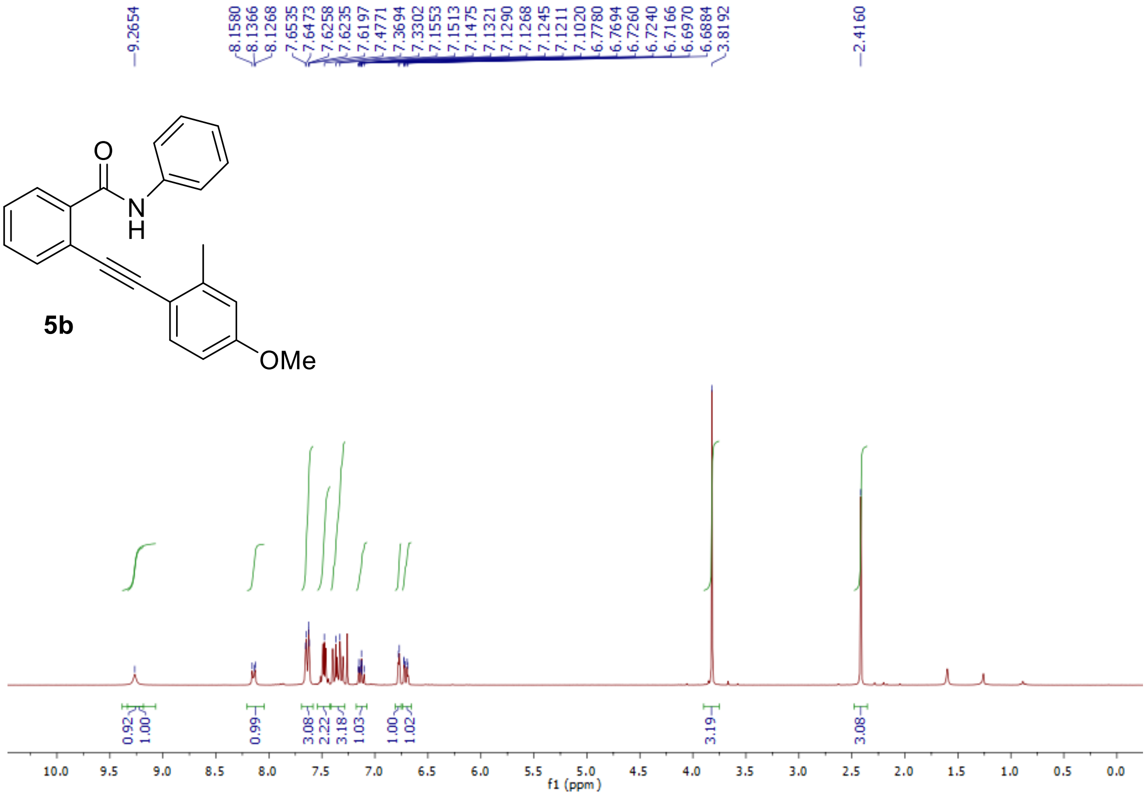

^{13}C NMR compound **5b** ($CDCl_3$, 75 MHz, 25 °C)

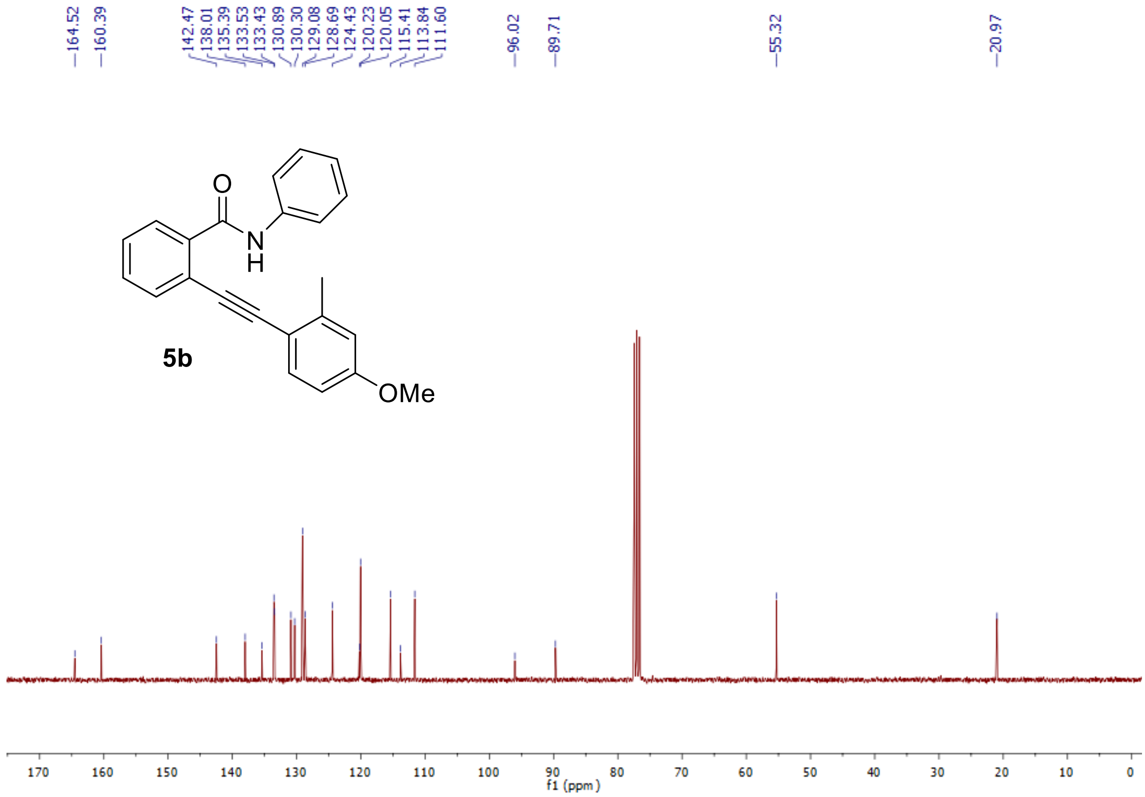

^{1}H NMR compound **5e** ($CDCl_3$, 300 MHz, 25 °C)

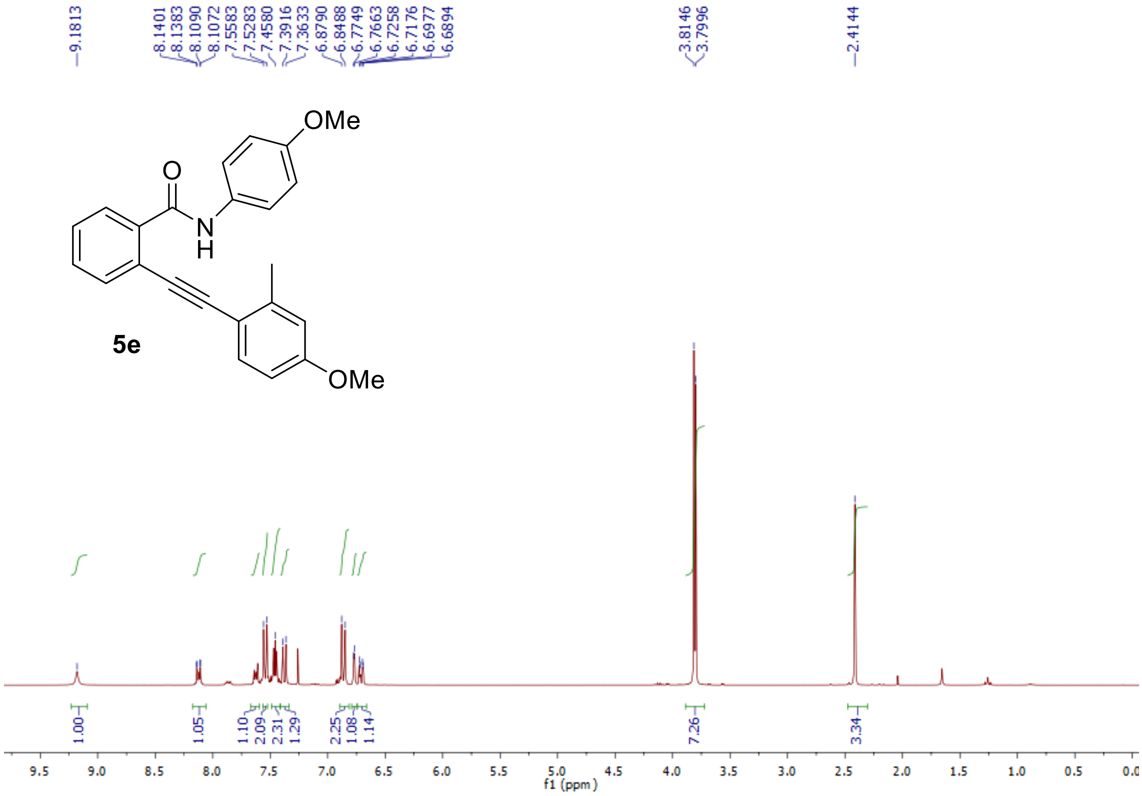

^{13}C NMR compound **5e** ($CDCl_3$, 75 MHz, 25 °C)

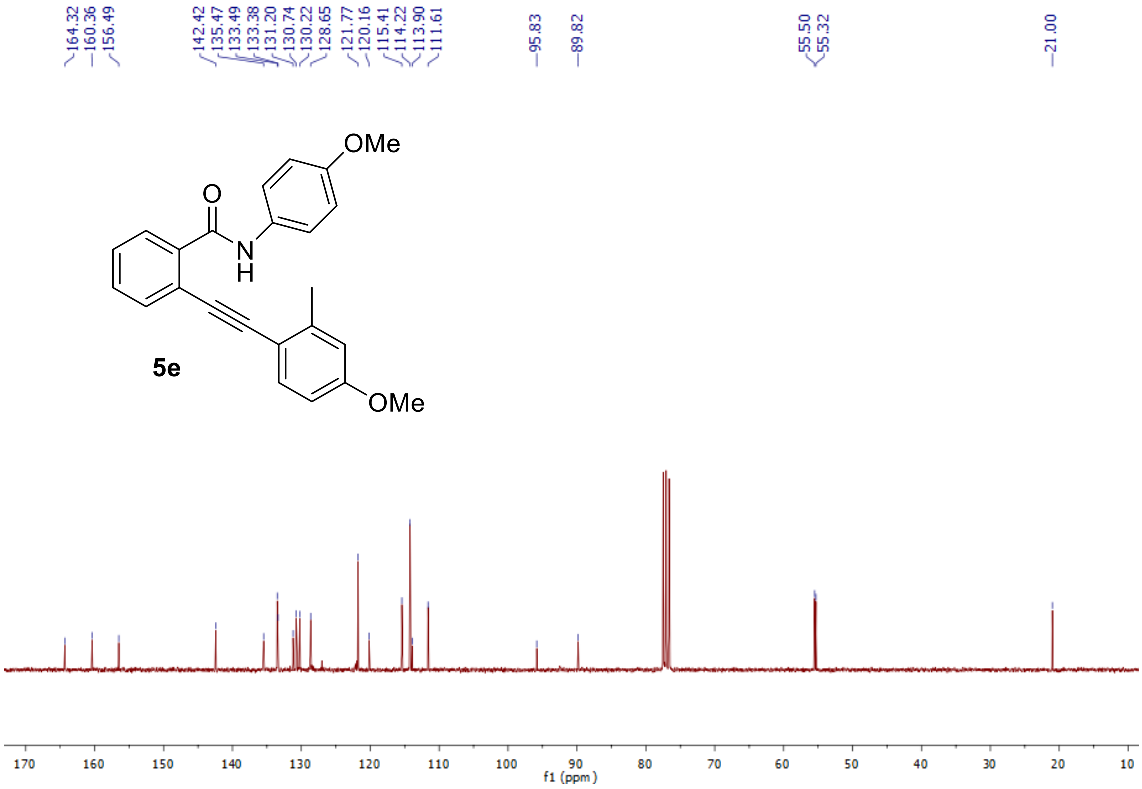

^{1}H NMR compound **3a** (d_6-acetone, 700 MHz, 25 °C)

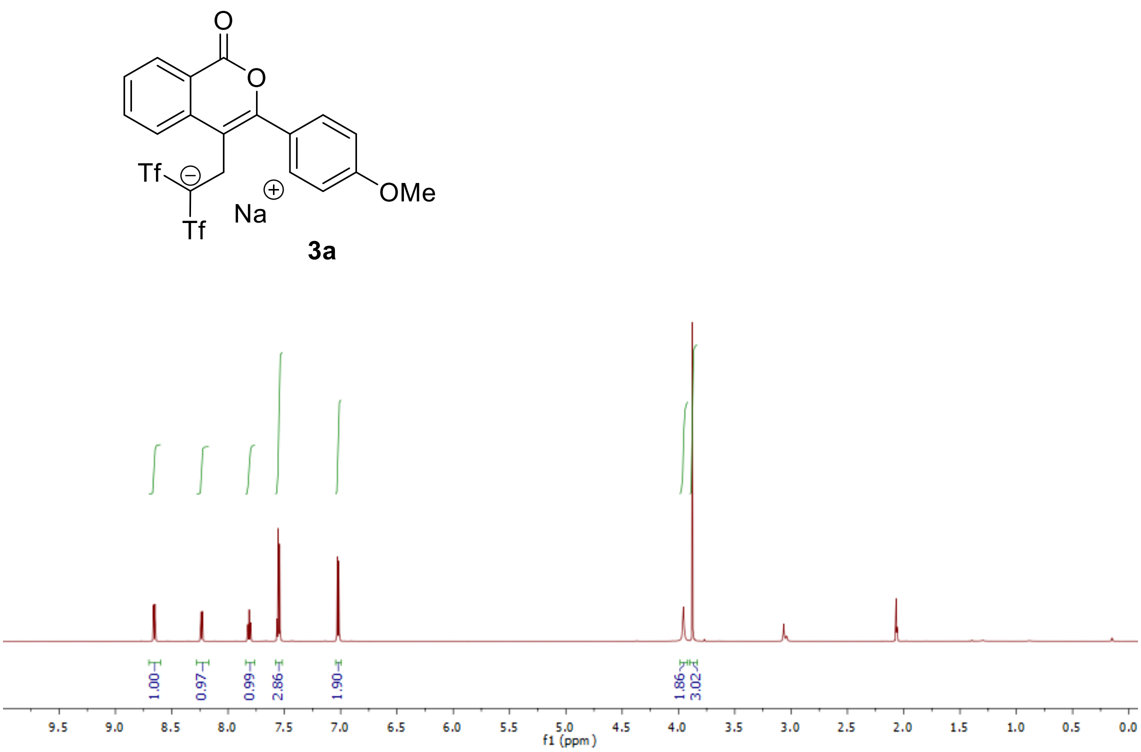

^{13}C NMR compound **3a** (d_6-acetone, 75 MHz, 25 °C)

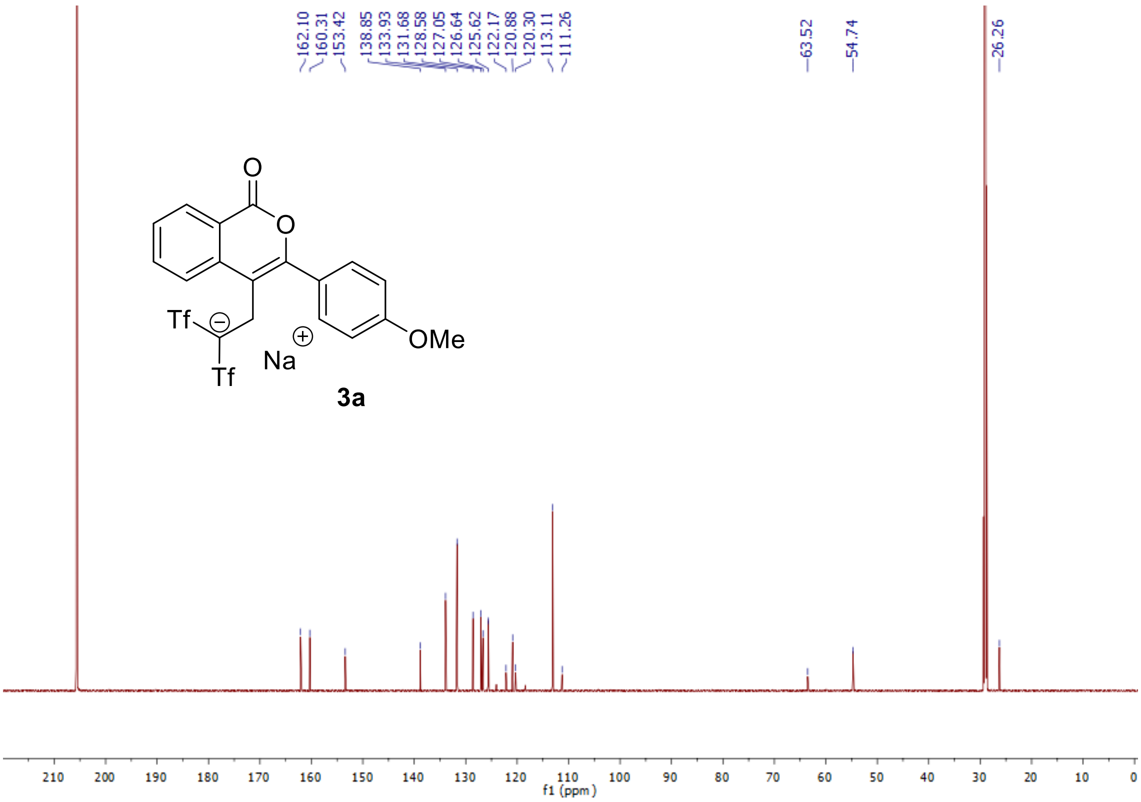

^{19}F NMR compound **3a** (d_6-acetone, 282 MHz, 25 ^oC)

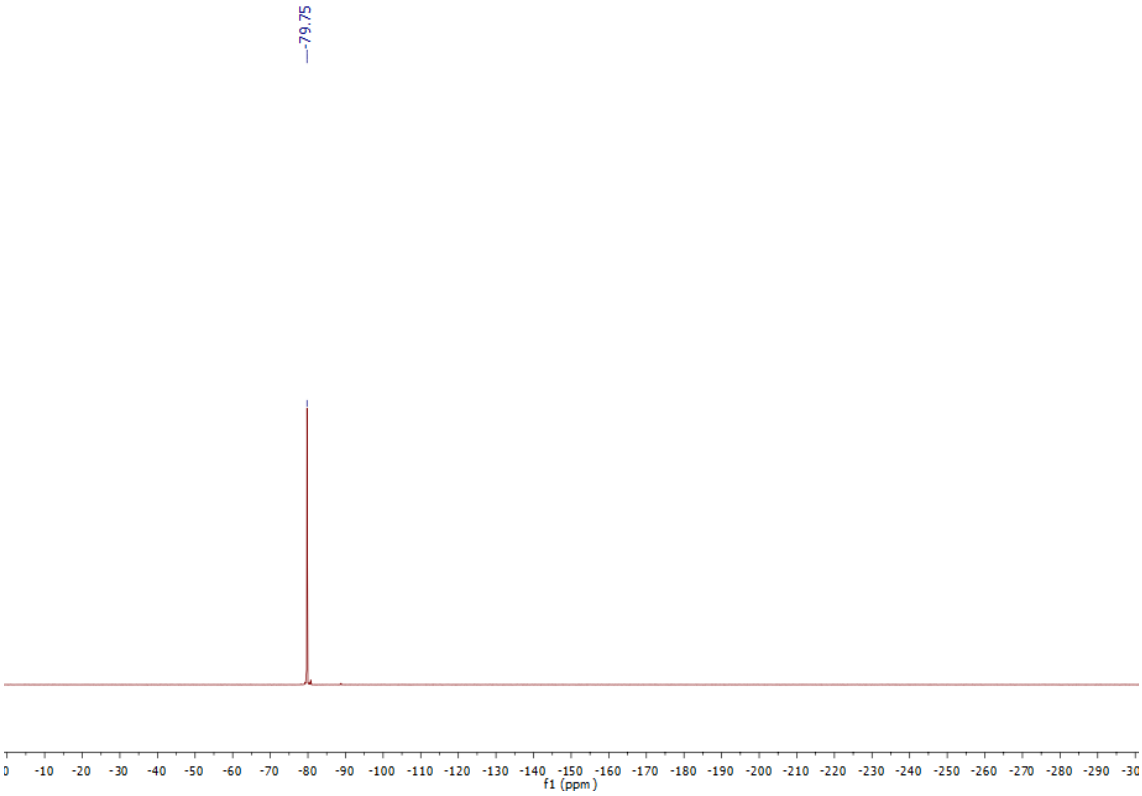

^{1}H NMR compound **3b** (d_6-acetone, 700 MHz, 25 °C)

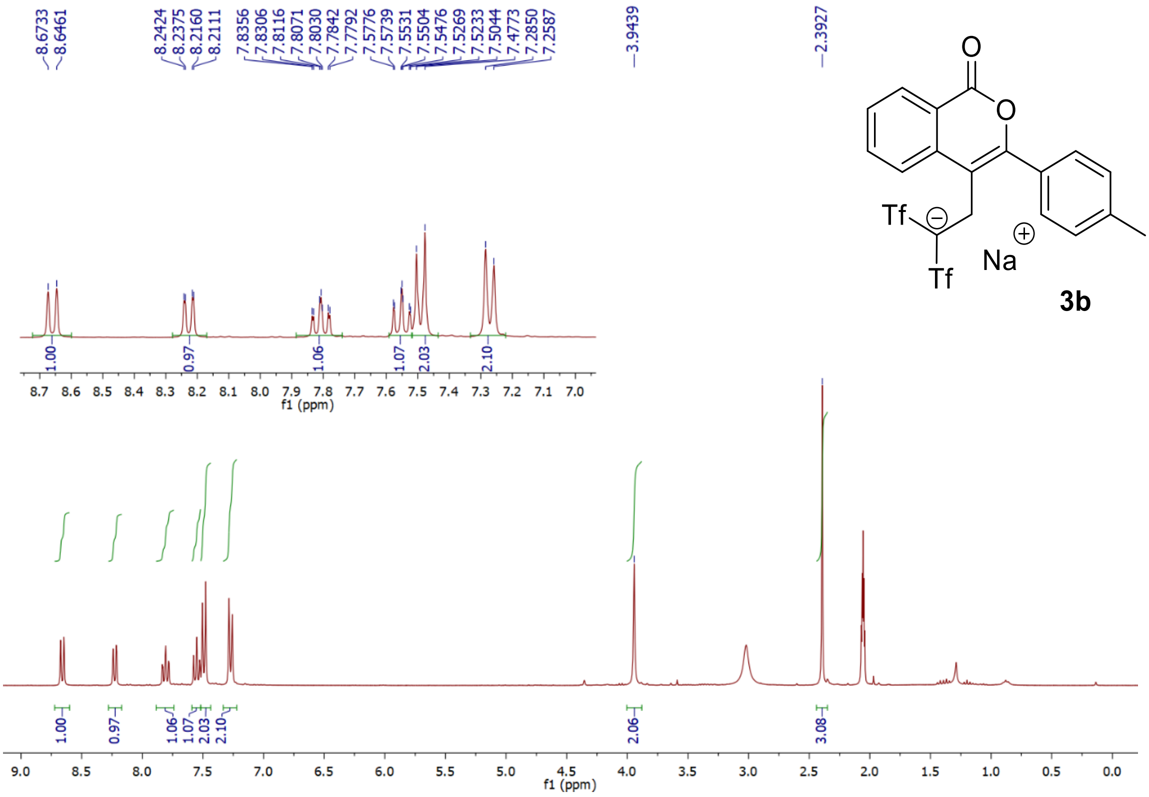

^{13}C NMR compound **3b** (d_6-acetone, 75 MHz, 25 °C)

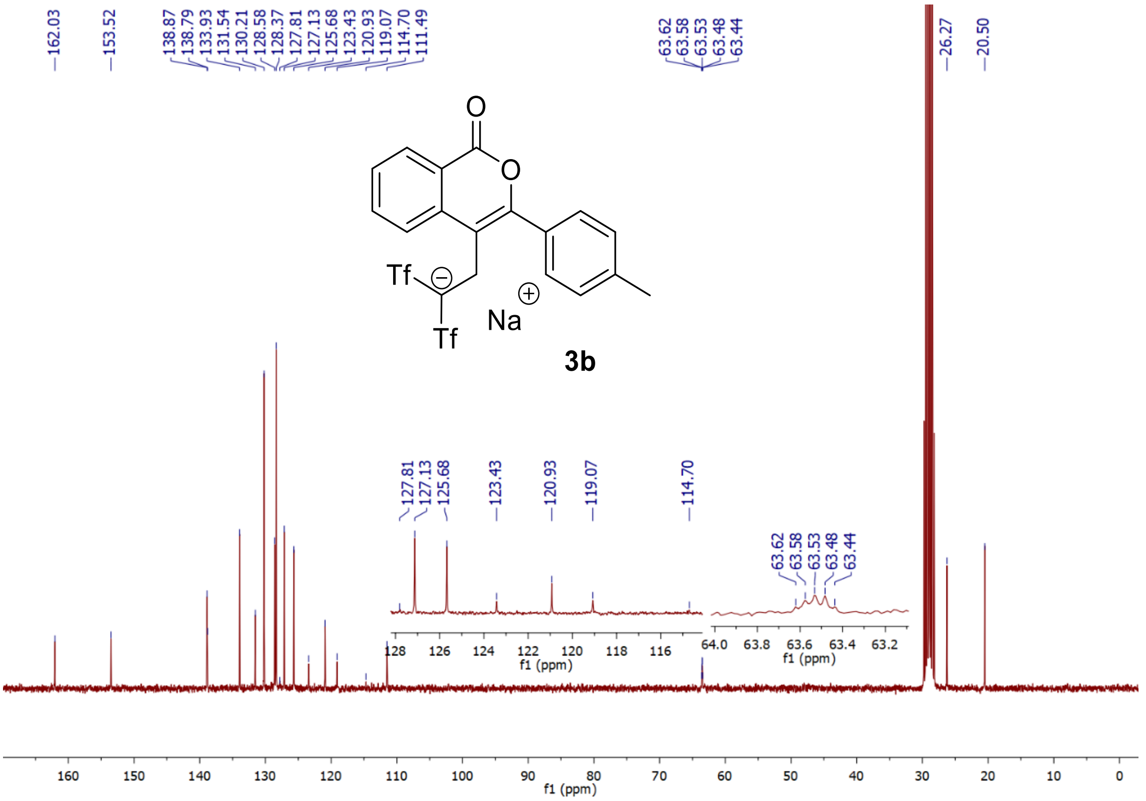

^{19}F NMR compound **3b** (d_6-acetone, 282 MHz, 25 ^{o}C)

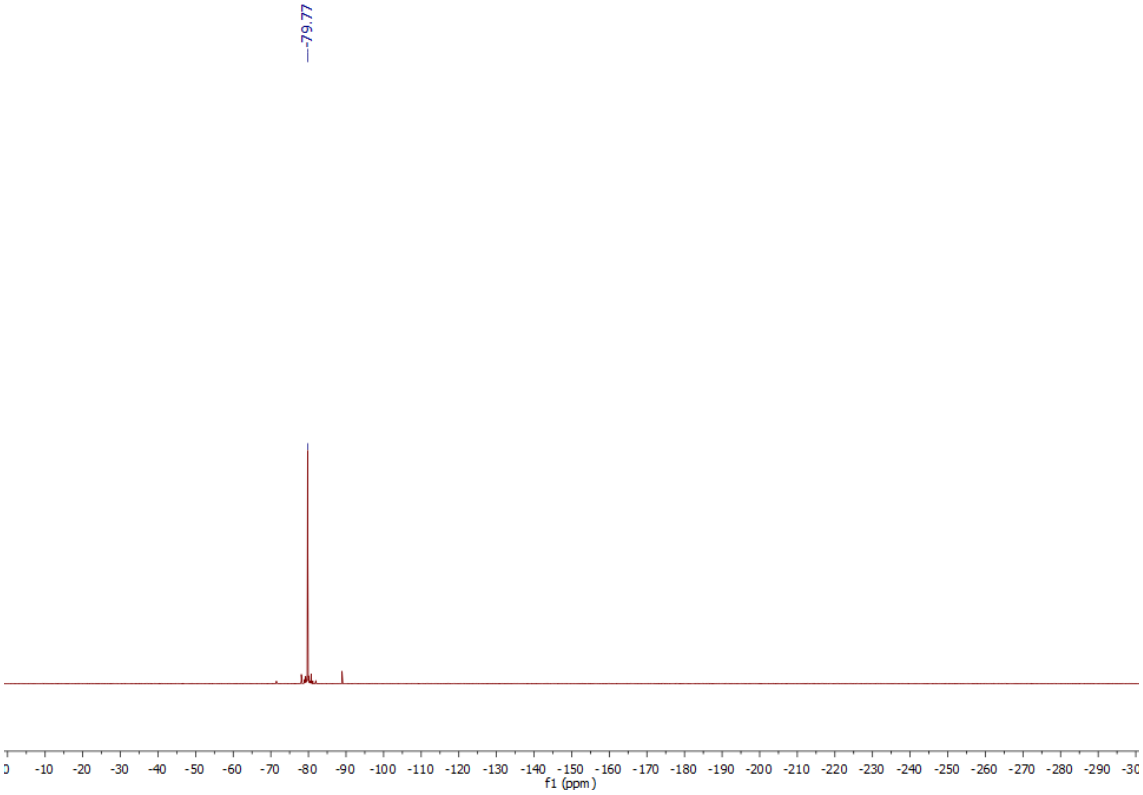

^{1}H NMR compound **3c** (d_6-acetone, 300 MHz, 25 °C)

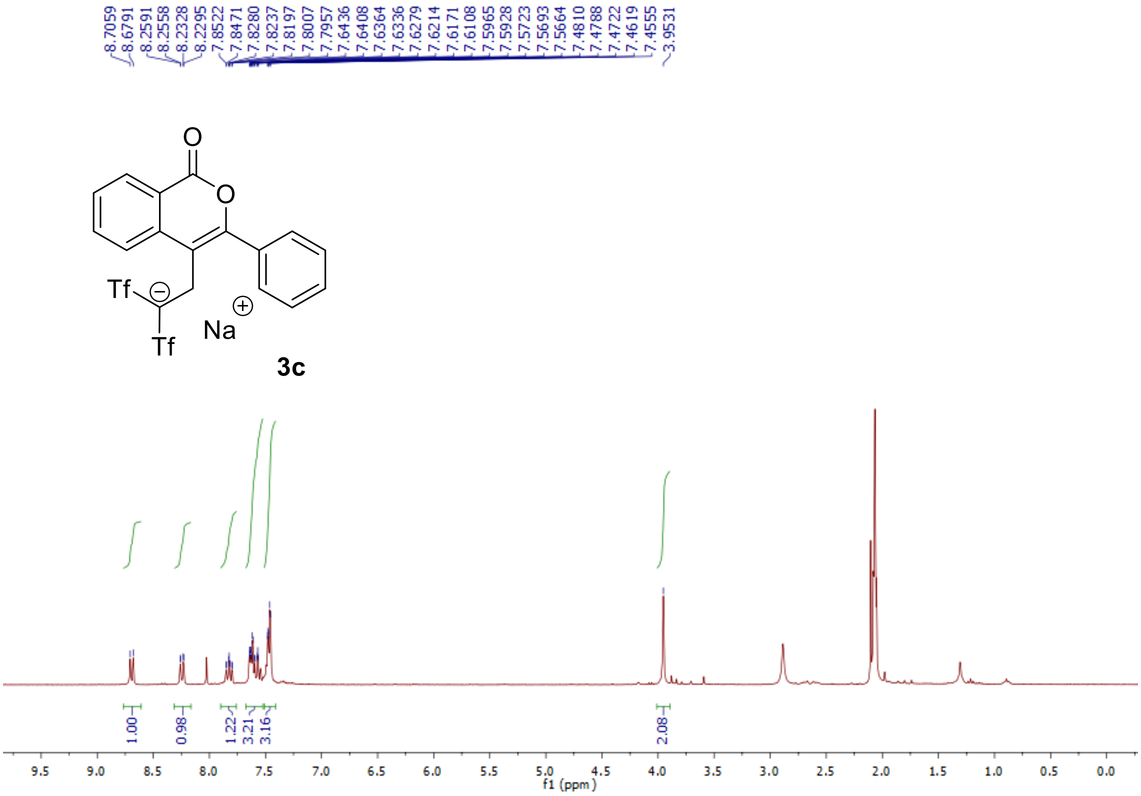

^{13}C NMR compound **3c** (d_6-acetone, 75 MHz, 25 °C)

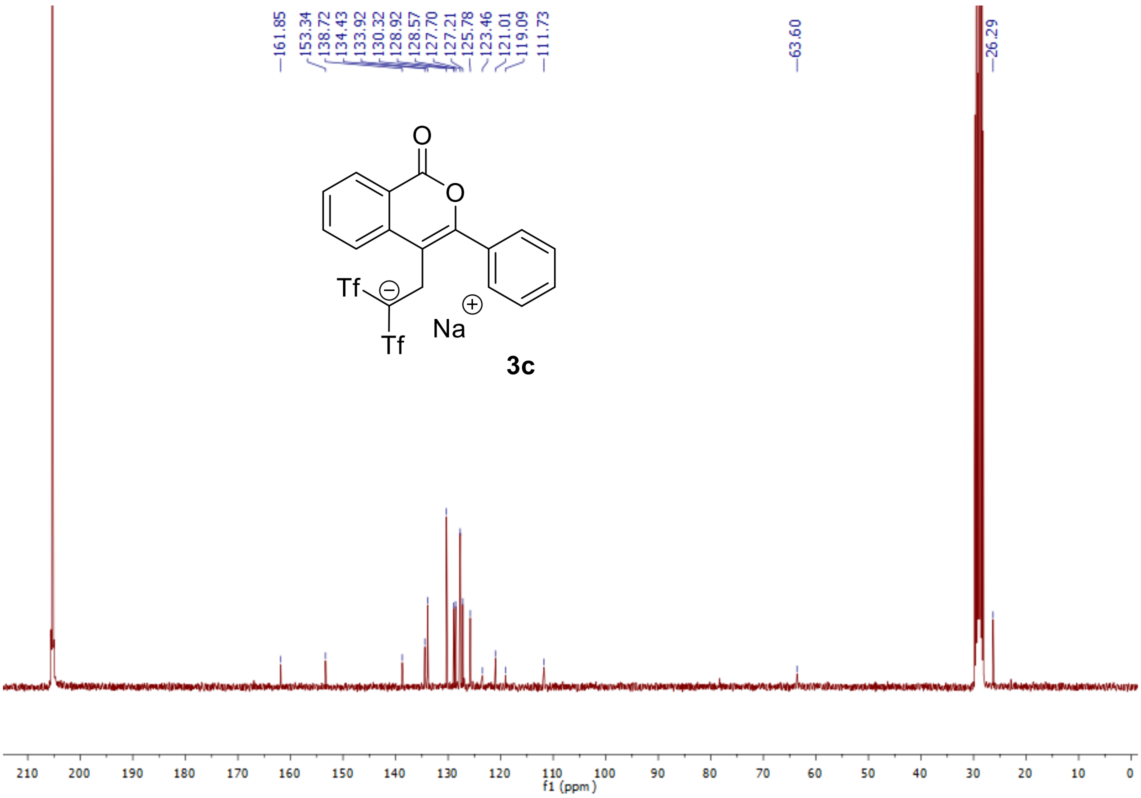

^{19}F NMR compound **3c** (d_6-acetone, 282 MHz, 25 ^{o}C)

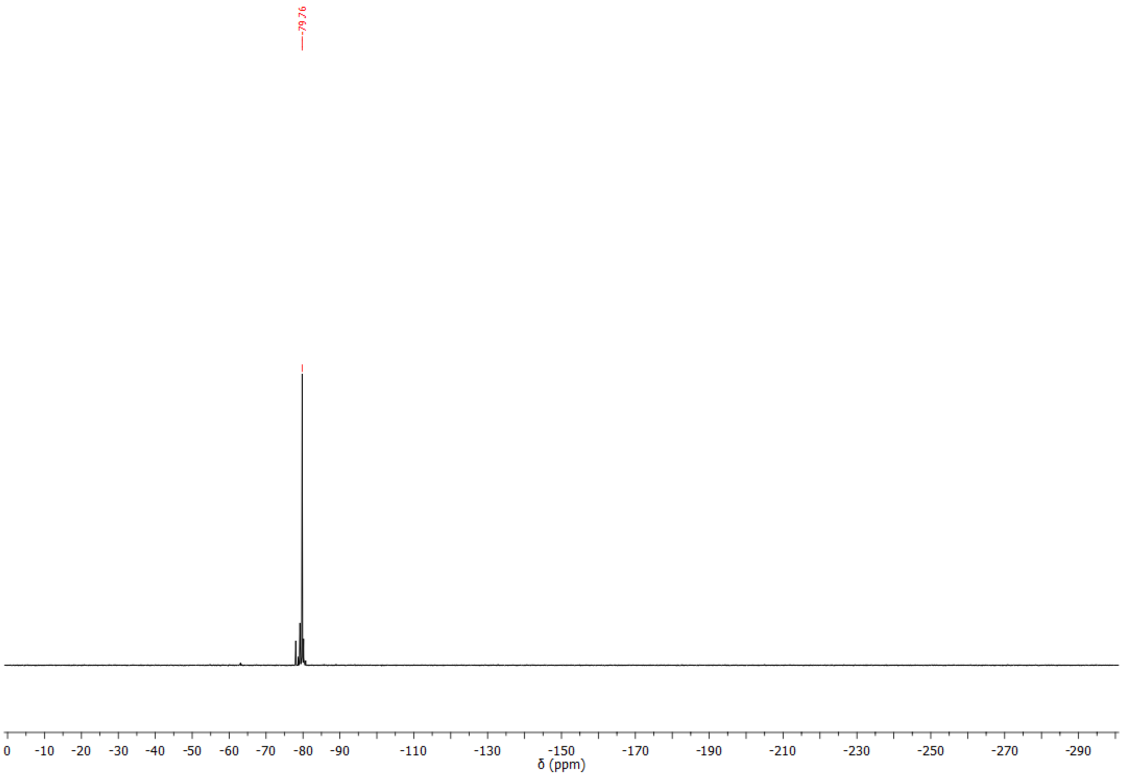

^{1}H NMR compound **3d** (d_6-acetone, 700 MHz, 25 °C)

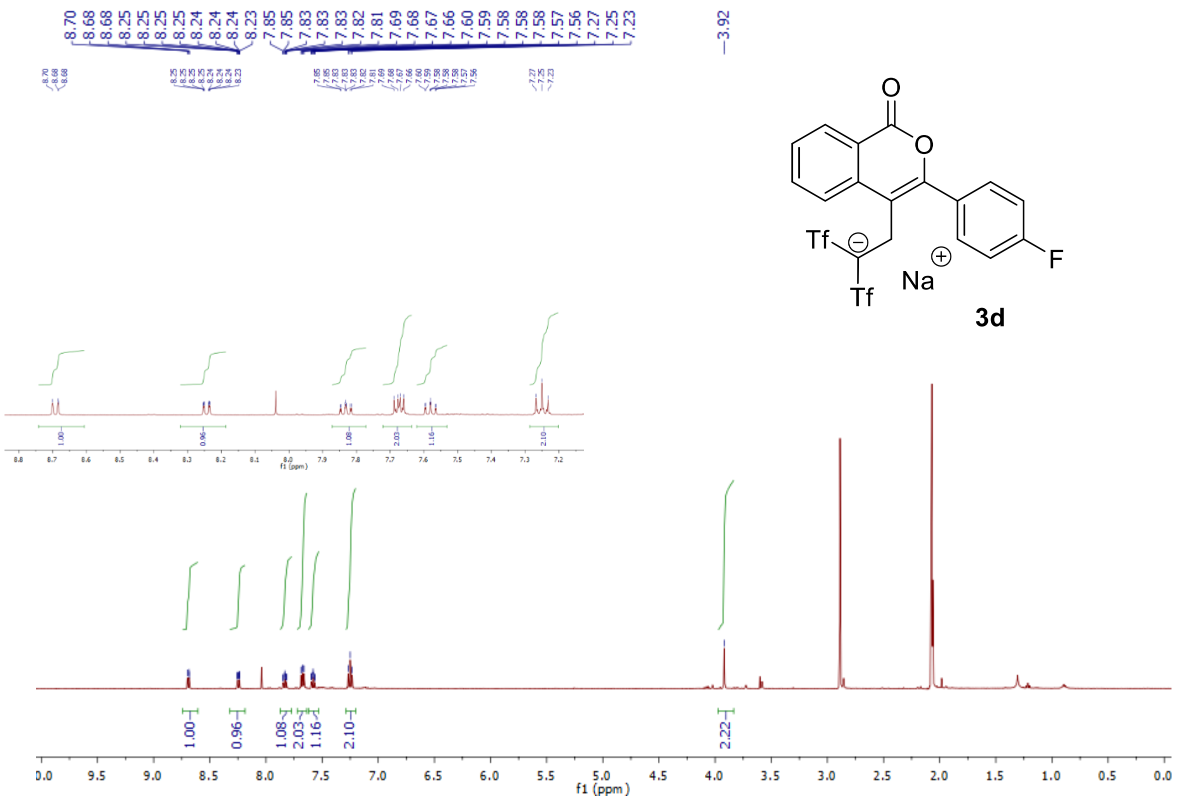

^{13}C NMR compound **3d** (d_6-acetone, 75 MHz, 25 °C)

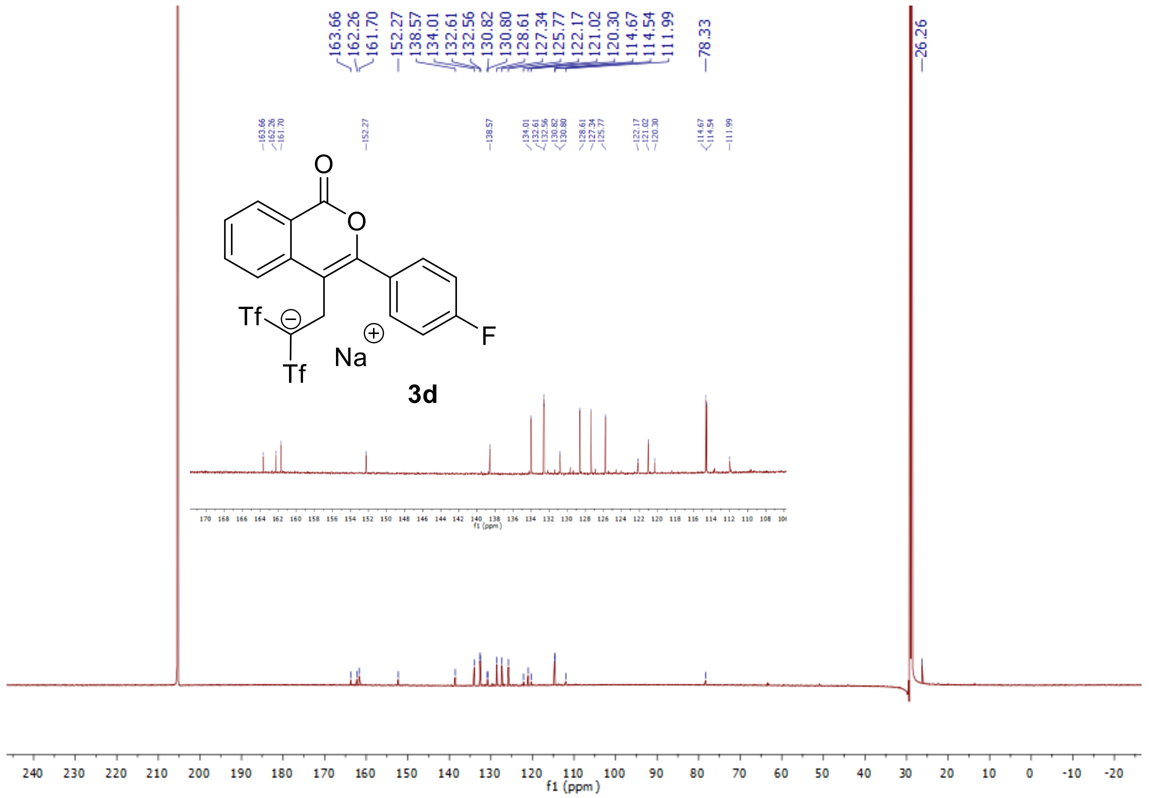

^{1}H NMR compound **3e** (d_6-acetone, 300 MHz, 25 °C)

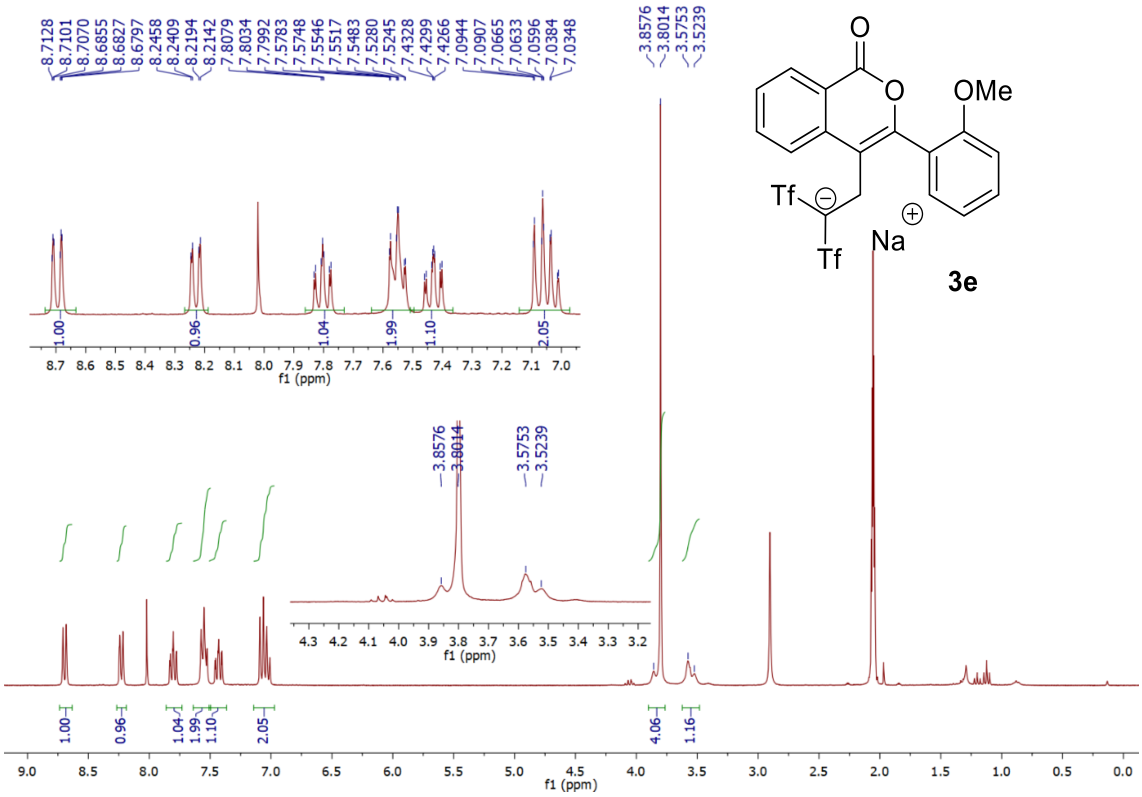

^{13}C NMR compound **3e** (d_6-acetone, 75 MHz, 25 °C)

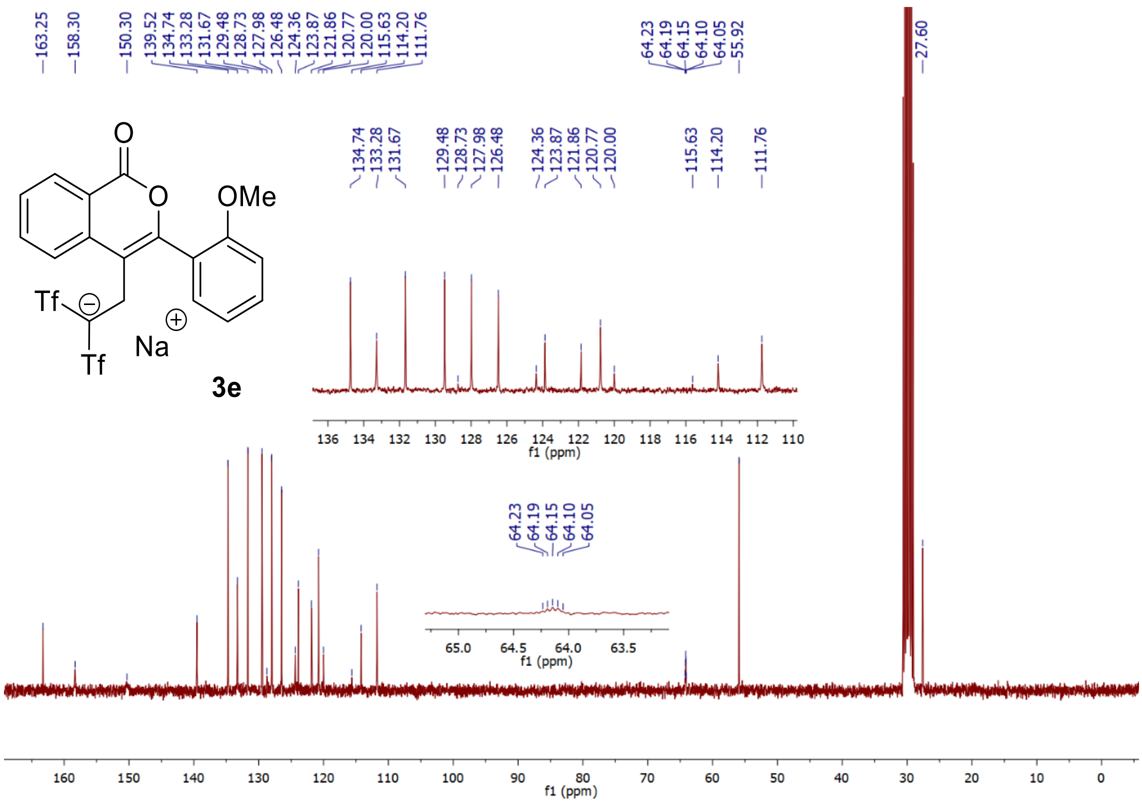

^{19}F NMR compound **3e** (d_6-acetone, 282 MHz, 25 °C)

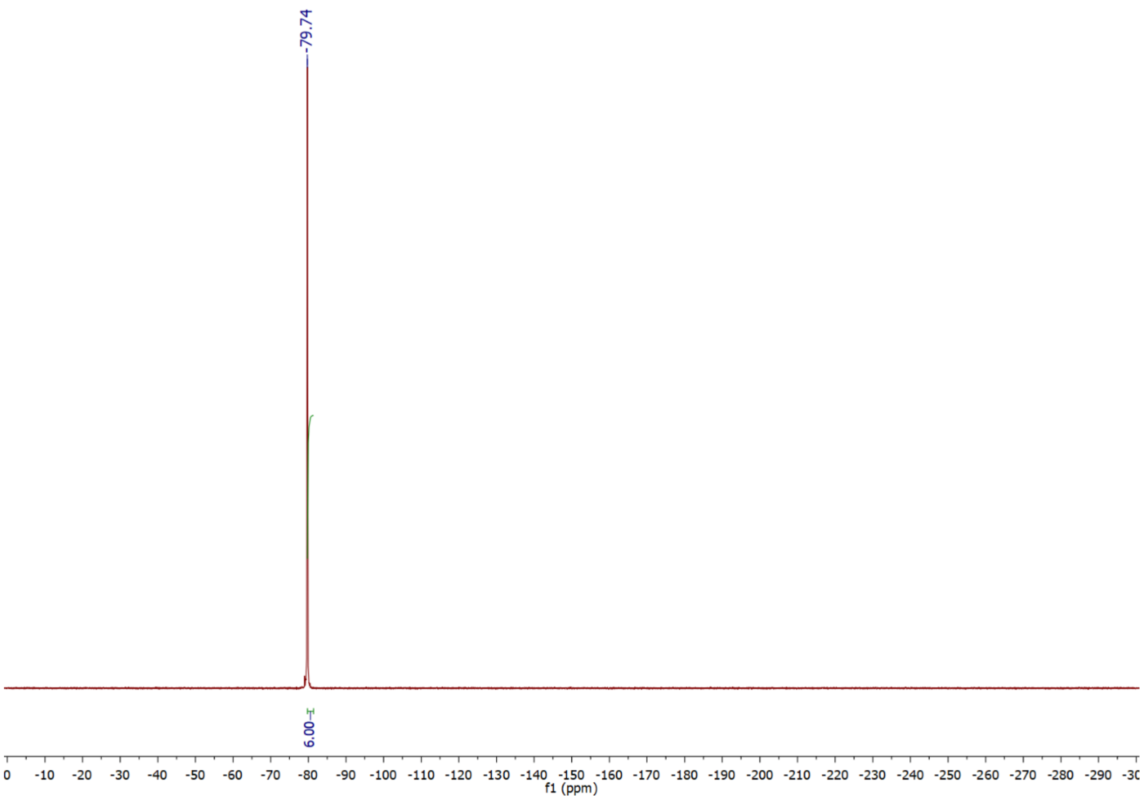

^{23}Na NMR compound **3e** (d_6-acetone, 132 MHz, 25 °C)

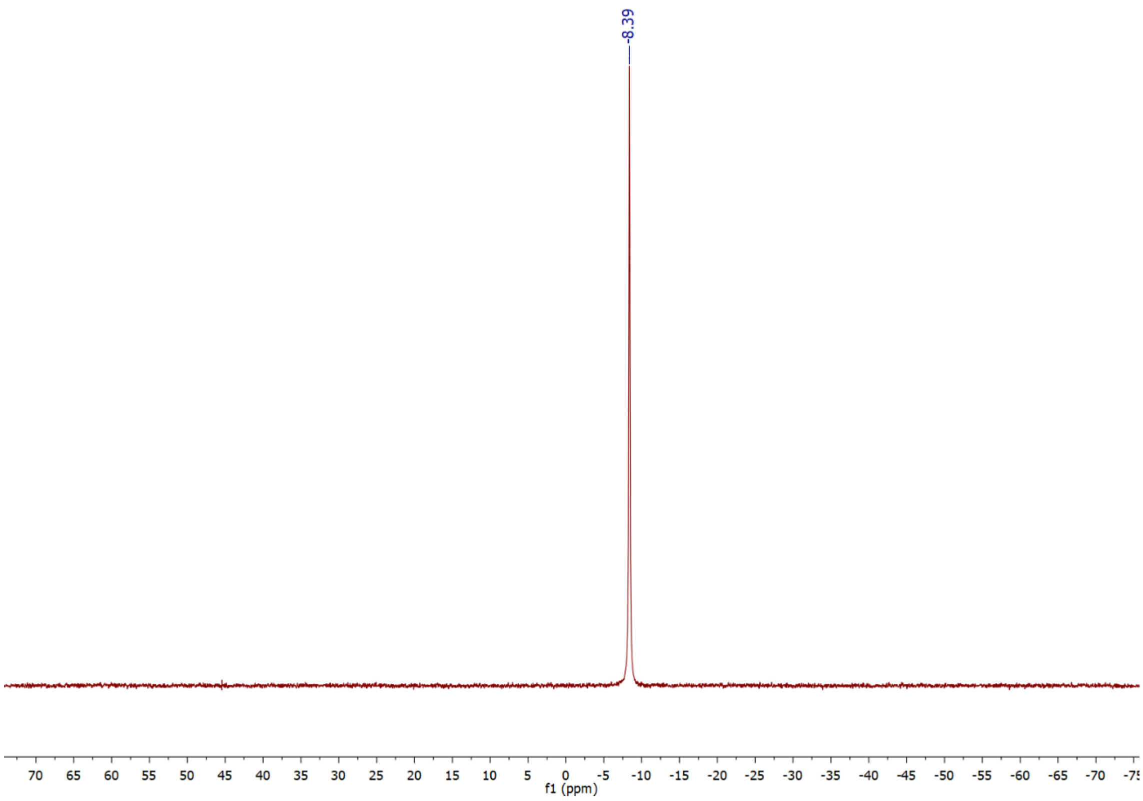

^{1}H NMR compound **3f** (d_6-acetone, 300 MHz, 25 °C)

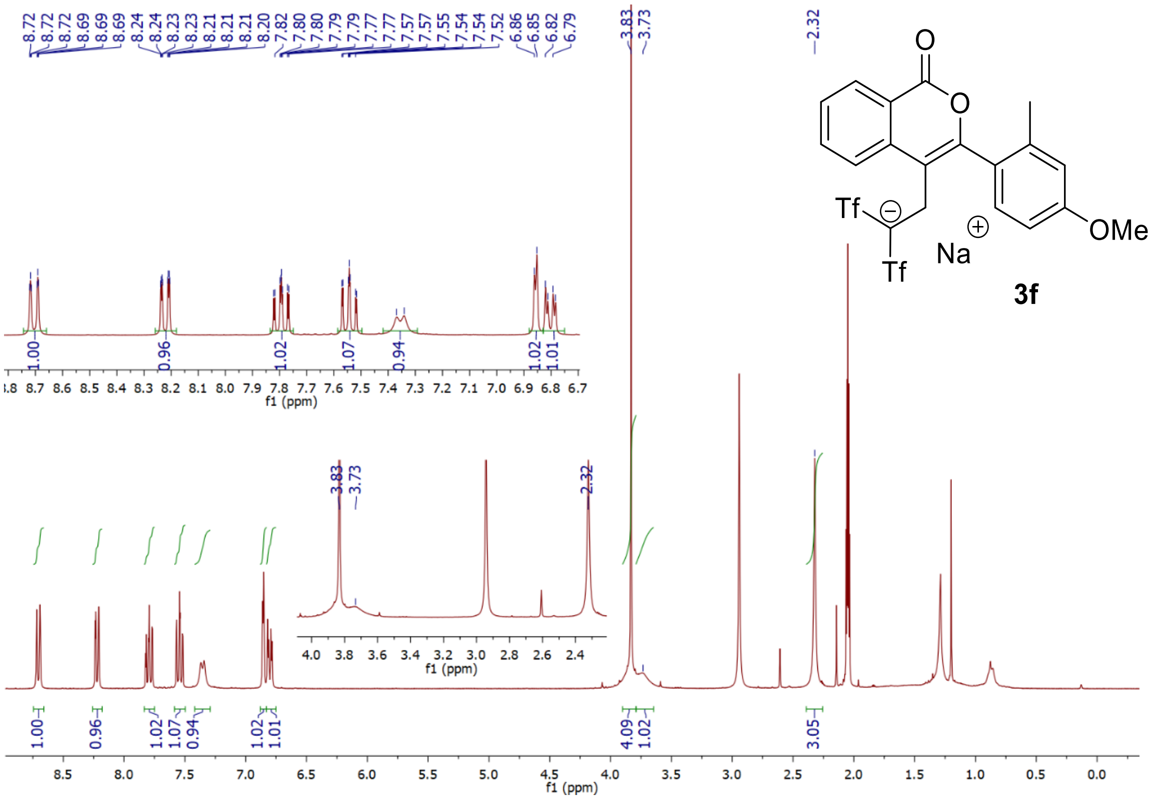

^{13}C NMR compound **3f** (d_6-acetone, 75 MHz, 25 °C)

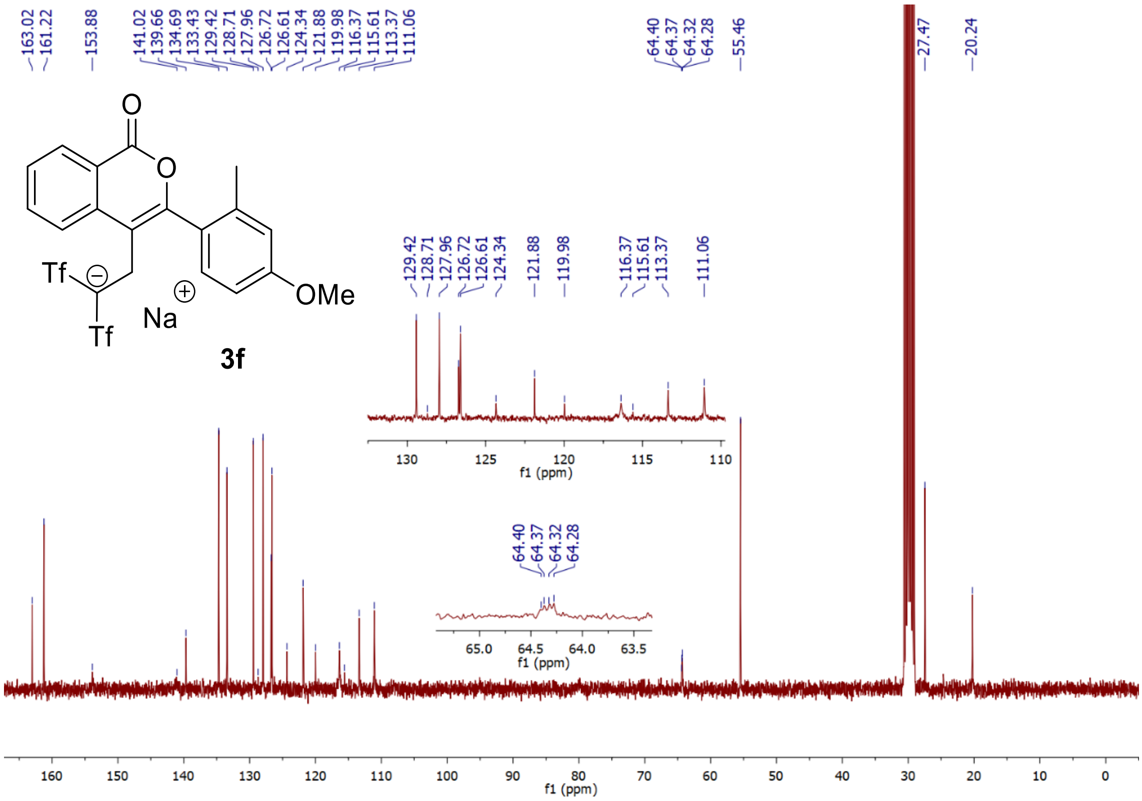

^{1}H – ^{1}H COSY compound **3f**

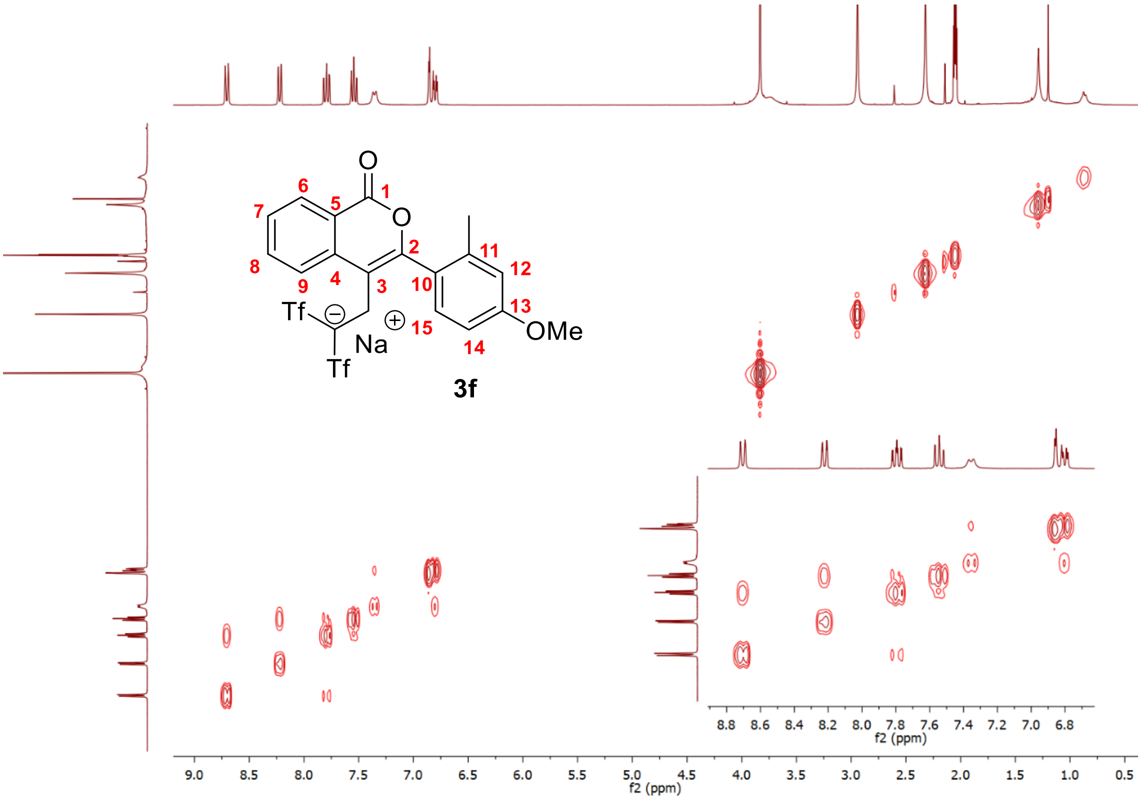

Table S7

#H	δ (ppm)	Multiplicity	Coupled with	J (Hz)
**H-9**	8.7	dt	H-8	8.2
			H-6	0.8
**H-6**	8.22	ddd	H-7	8.0
			H-8	1.5
			H-9	0.8
**H-8**	7.79	ddd	H-9	8.2
			H-7	7.2
			H-6	1.5
**H-7**	7.54	ddd	H-6	8.0
			H-8	7.2
			H-9	1.1
**H-15**	7.35	d	H-14	8.4
**H-12**	6.86	d	H-14	2.7
**H-14**	6.80	dd	H-15	8.4
			H-12	2.7
**OCH_3, CH*H*C$^-$Tf$_2$**	3.83	s	-	-
			C*H*HC$^-$Tf$_2$	-
**C*H*HC$^-$Tf$_2$**	3.73	s	CH*H*C$^-$Tf$_2$	-
**CH_3**	2.32	s	-	-

$^{1}H – ^{13}C$ HMQC compound **3f**

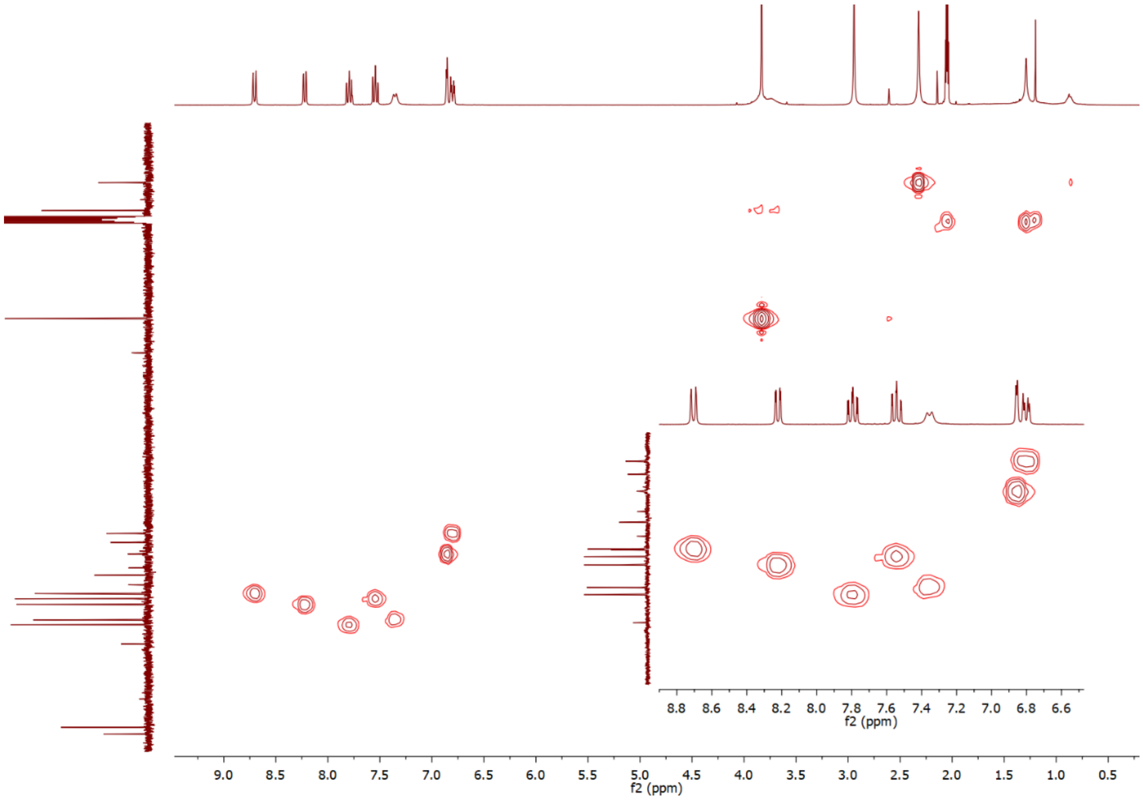

$^{1}H – ^{13}C$ HMBC compound **3f**

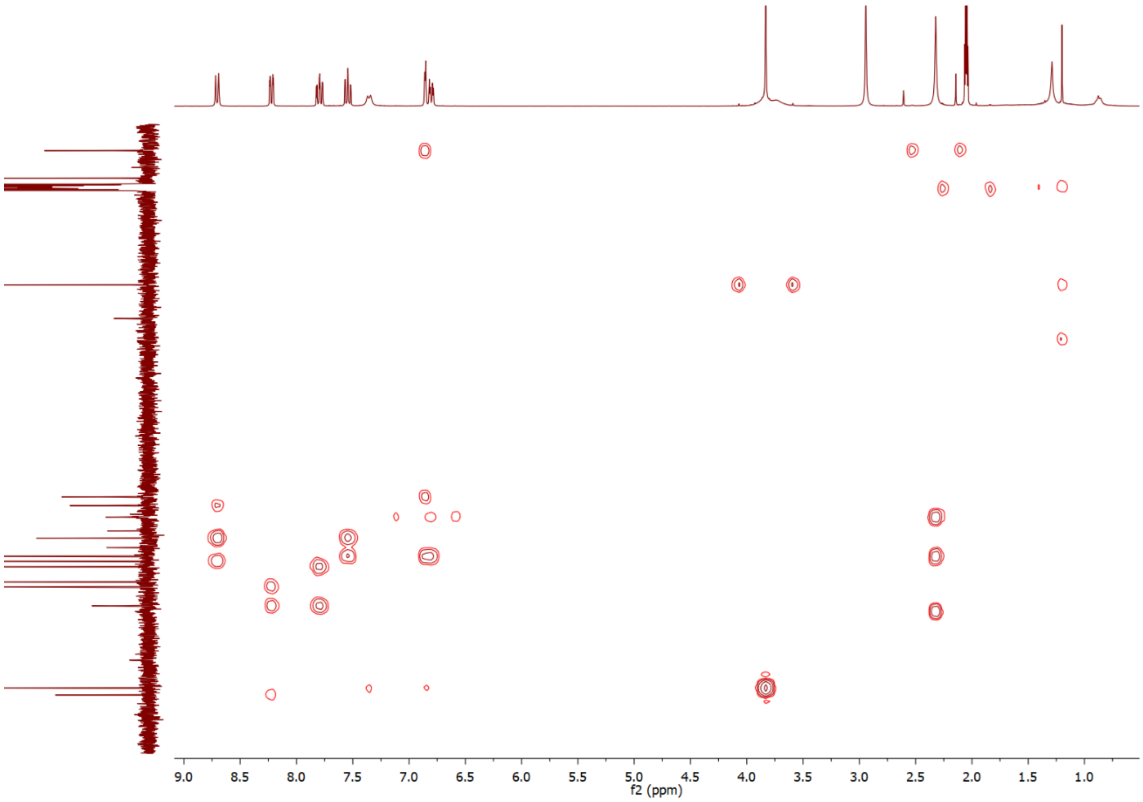

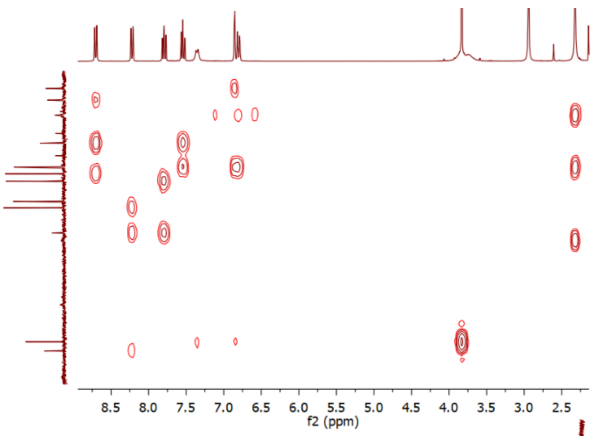

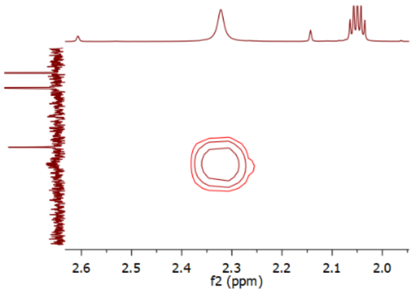

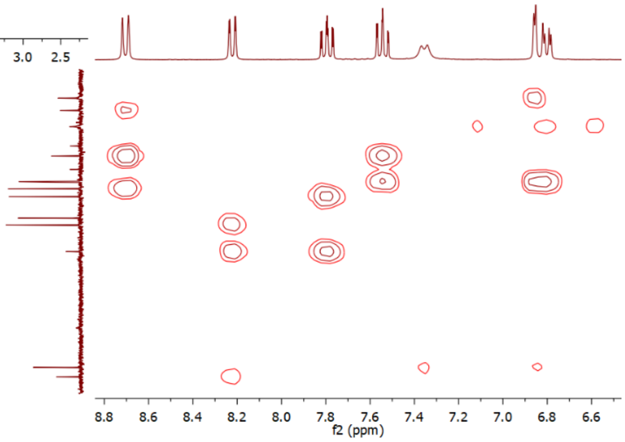

Table S8

#C	δ (ppm)		Coupled with	
**C-1**	163.0	C=o	H-6 (^{3}J)	Fig.1
**C-13**	161.2	C_{Ar}	H-12 (^{2}J), H-14 (^{2}J), H-15 (^{3}J), OCH_3 (^{3}J)	Fig.1
**C-2**	153.9	C_{Ar}	-	-
**C-11**	141.0	C_{Ar}	CH_3 (^{2}J)	Fig.2
**C-4**	139.7	C_{Ar}	H-6 (^{3}J), H-8 (^{3}J)	Fig.2
**C-8**	134.7	CH_{Ar}	H-6 (^{3}J)	Fig.2
**C-15**	133.4	CH_{Ar}	-	-
**C-6**	129.4	CH_{Ar}	H-8 (^{3}J)	Fig.2
**C-7**	128.0	CH_{Ar}	H-9 (^{3}J)	Fig.3
**C-10**	126.7	C_{Ar}	H-12 (^{3}J), H-14 (^{3}J), CH_3 (^{3}J)	Fig.2
**C-9**	126.6	CH_{Ar}	H-7 (^{3}J)	Fig.3
**CF_3**	122.2		-	-
**C-5**	121.9	C_{Ar}	H-7 (^{3}J), H-9 (^{3}J)	Fig.3
**C-12**	116.4	CH_{Ar}	H-14 (^{3}J), CH_3 (^{3}J)	Fig.3
**C-3**	113.4	C_{Ar}	H-9 (^{3}J)	Fig.3
**C-14**	111.1	CH_{Ar}	H-12 (^{3}J)	Fig.3
**$^-CTf_2$**	64.3		-	-
**OCH_3**	55.5	CH	-	-
**CH_2**	24.5	CH	-	-
**CH_3**	20.2	CH	H-12 (^{3}J)	Fig.3

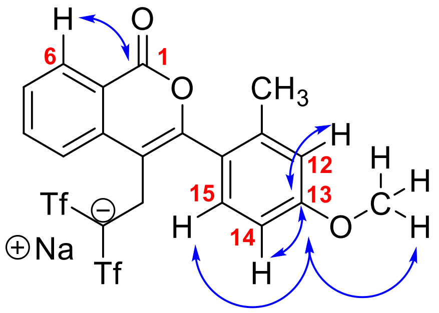

Chart S1

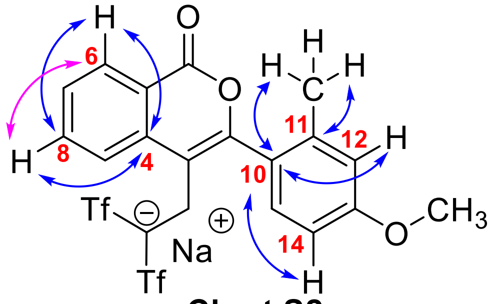

Chart S2

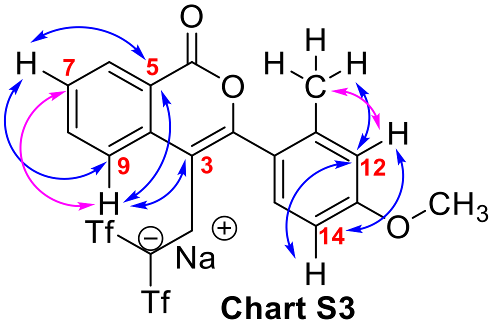

Chart S3

^{19}F NMR compound **3f** (d$_6$-acetone, 282 MHz, 25 ^{o}C)

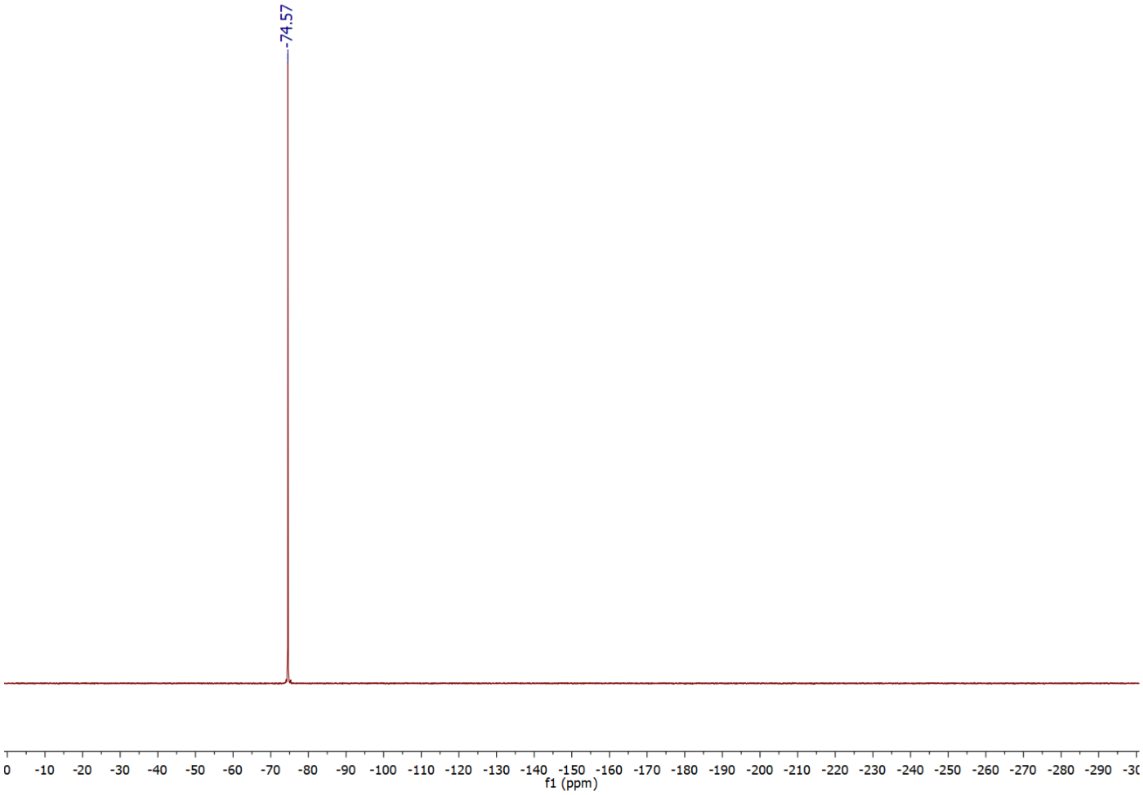

^{1}H NMR compound **3g** (d_6-acetone, 300 MHz, 25 °C)

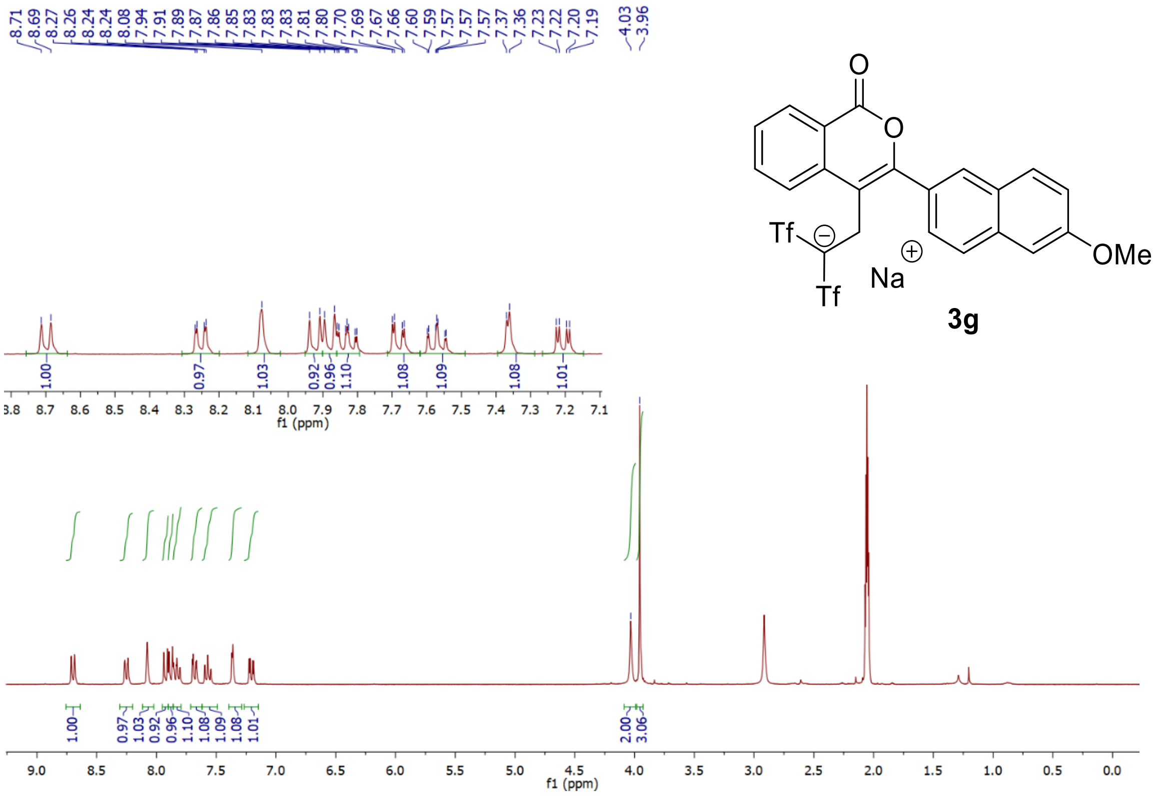

^{13}C NMR compound **3g** (d_6-acetone, 75 MHz, 25 °C)

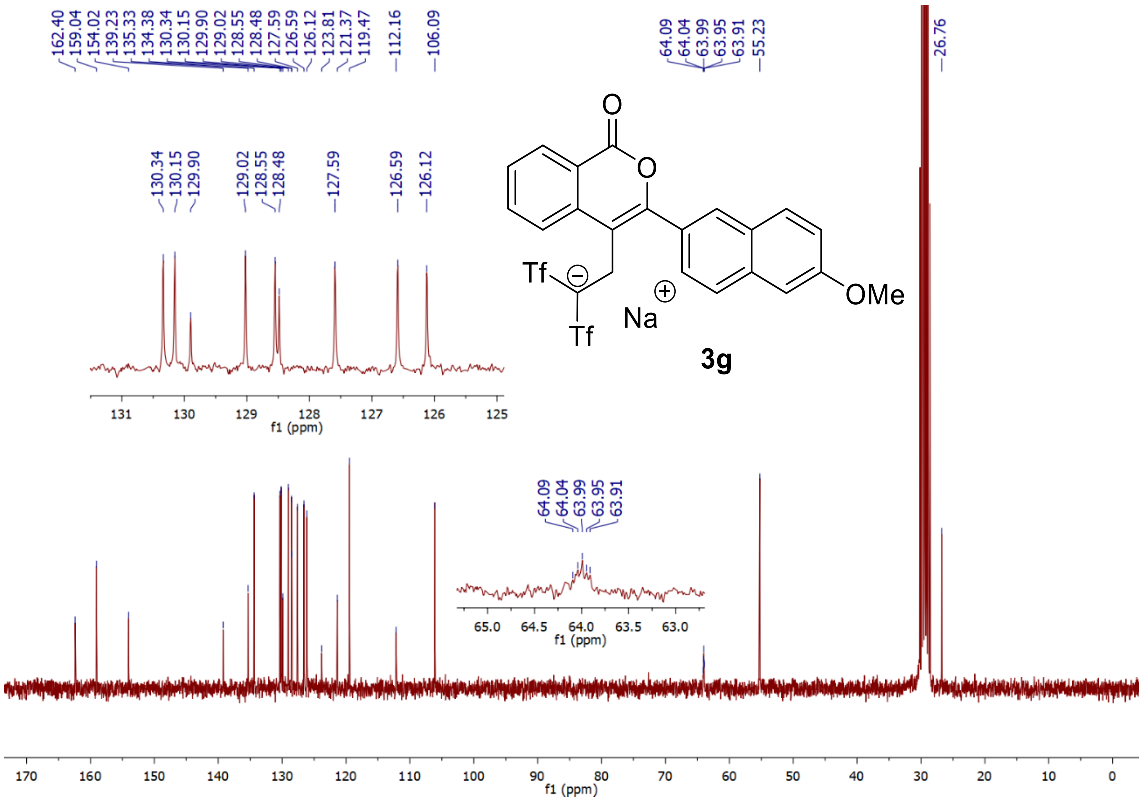

^{19}F NMR compound **3g** (d_6-acetone, 282 MHz, 25 ^{o}C)

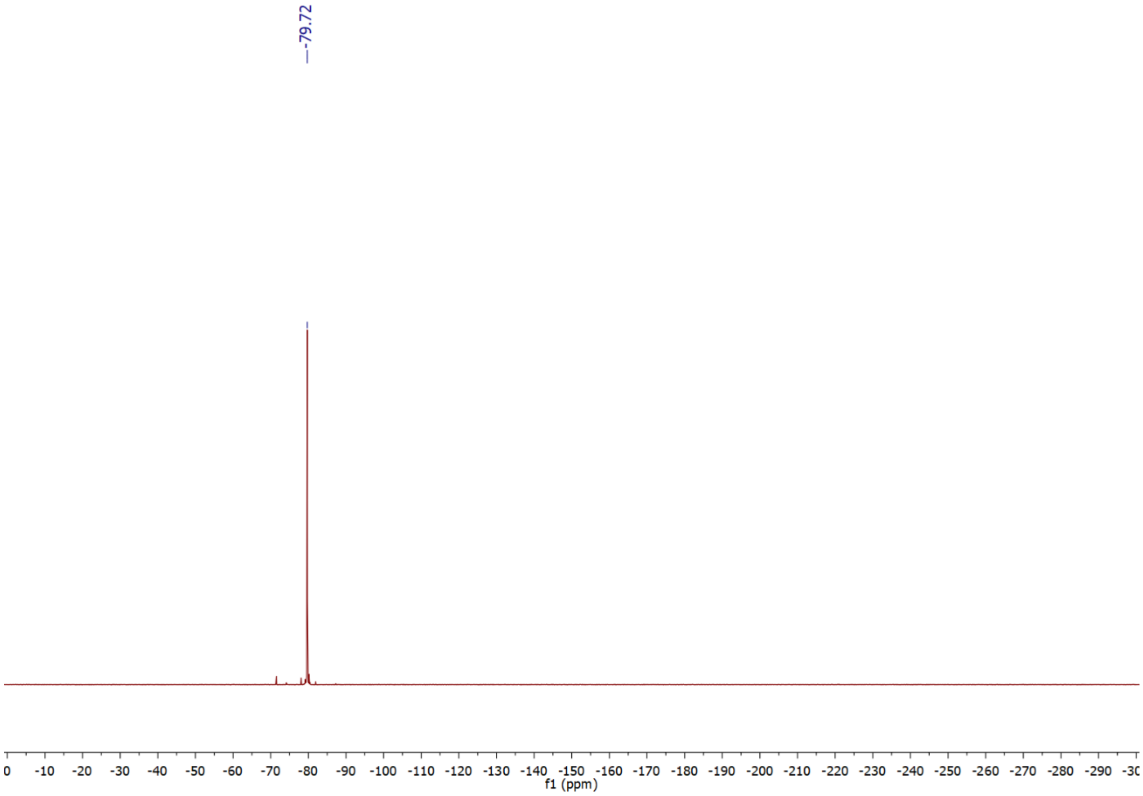

^{1}H NMR Compound **3i** (d_6-acetone, 300 MHz, 25 °C)

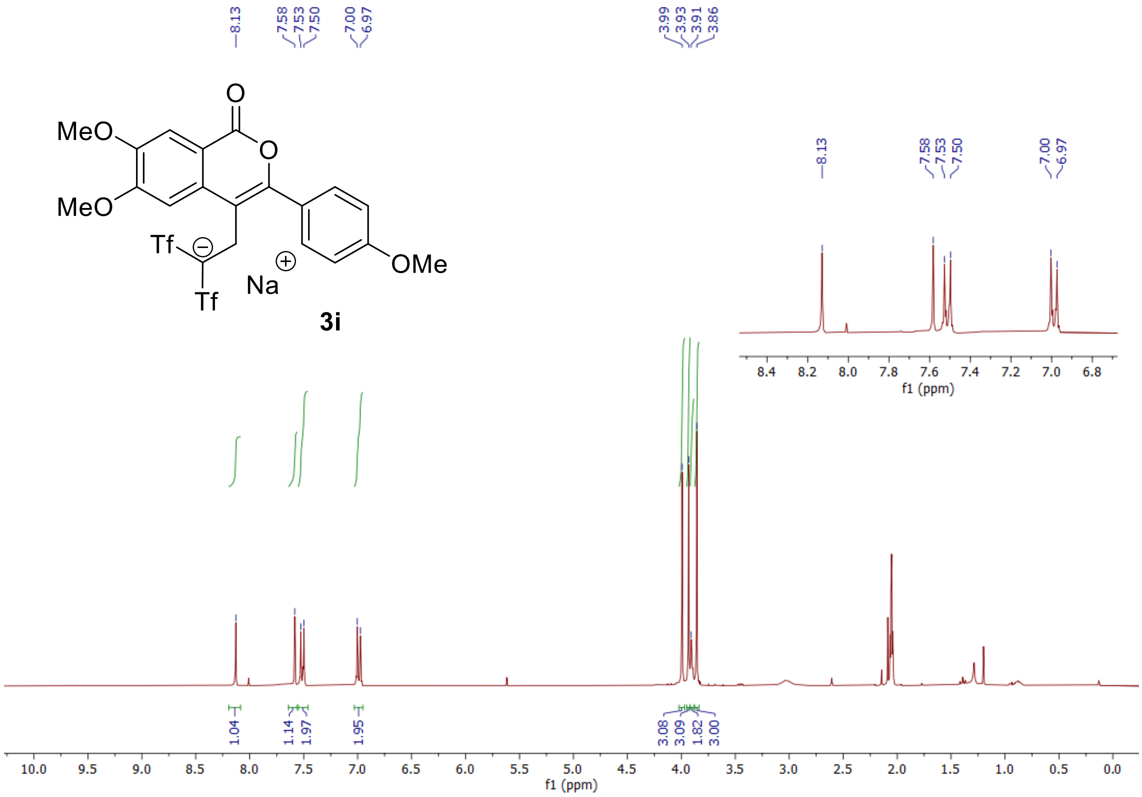

^{13}C NMR Compound **3i** (d_6-acetone, 75 MHz, 25 °C)

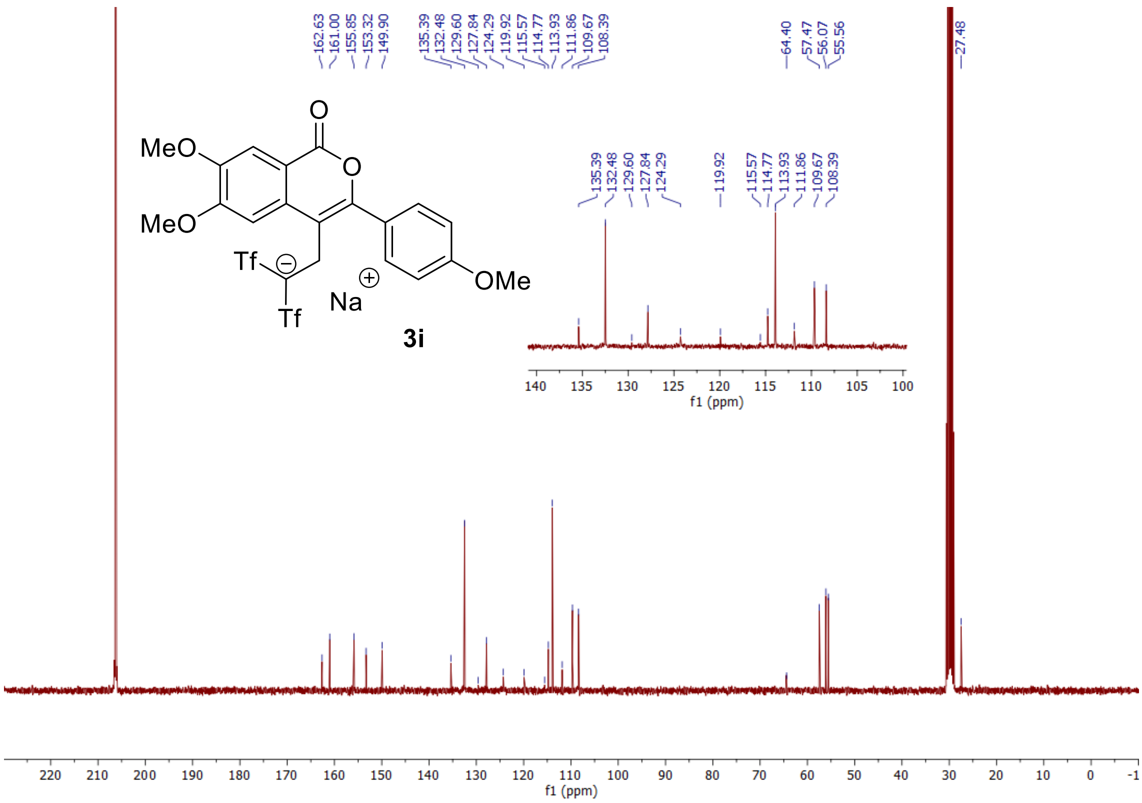

^{19}F NMR Compound **3i** (d_6-acetone, 282 MHz, 25 ^{o}C)

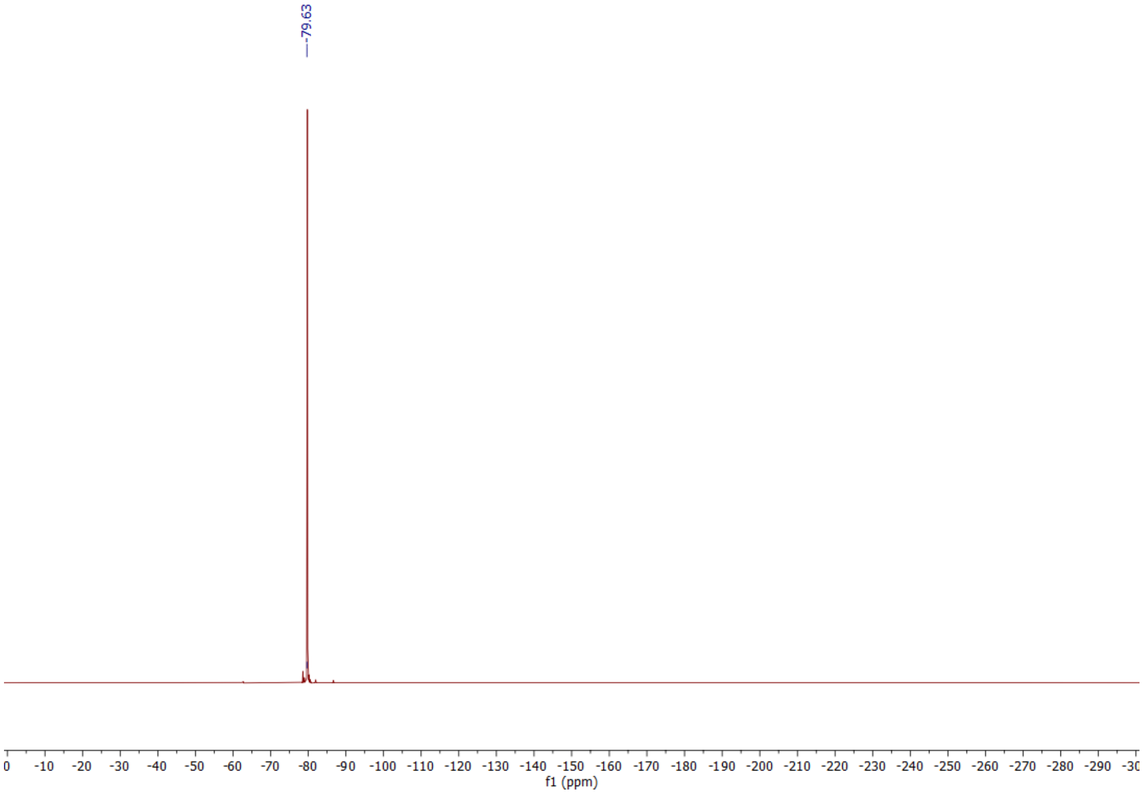

^{1}H NMR Compound **3j** (d_6-acetone, 300 MHz, 25 °C)

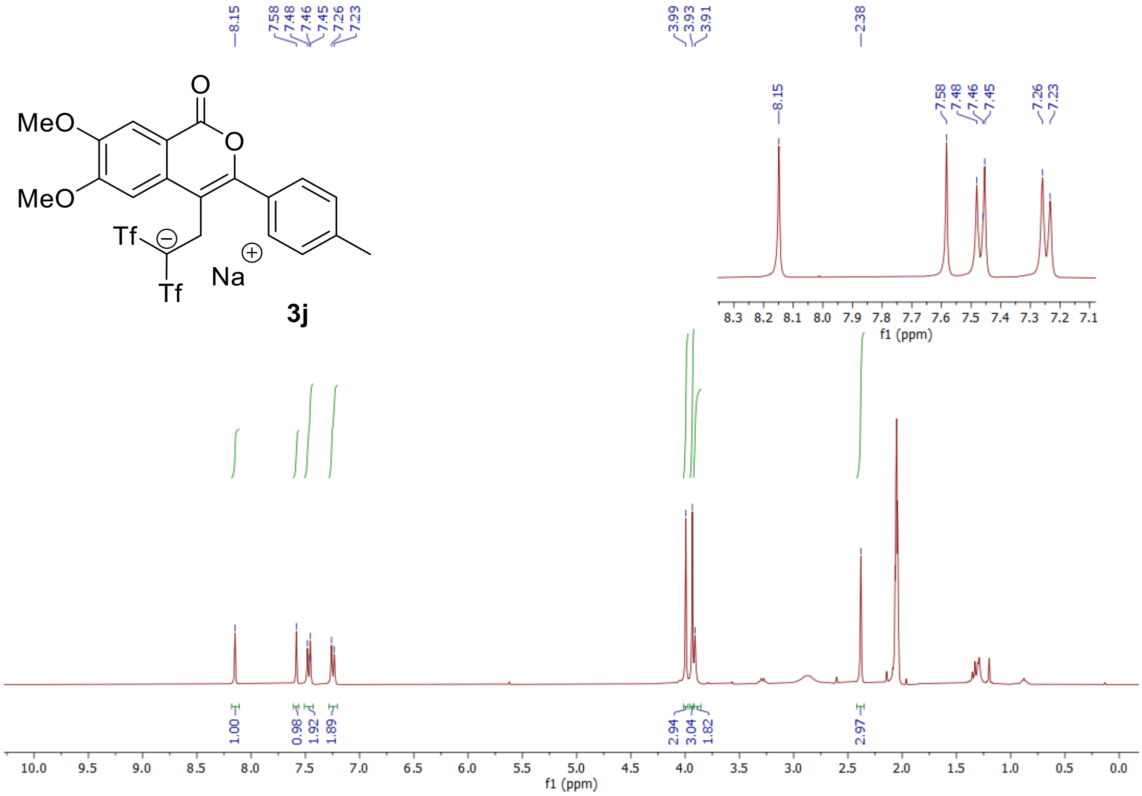

^{13}C NMR Compound **3j** (d_6-acetone, 75 MHz, 25 °C)

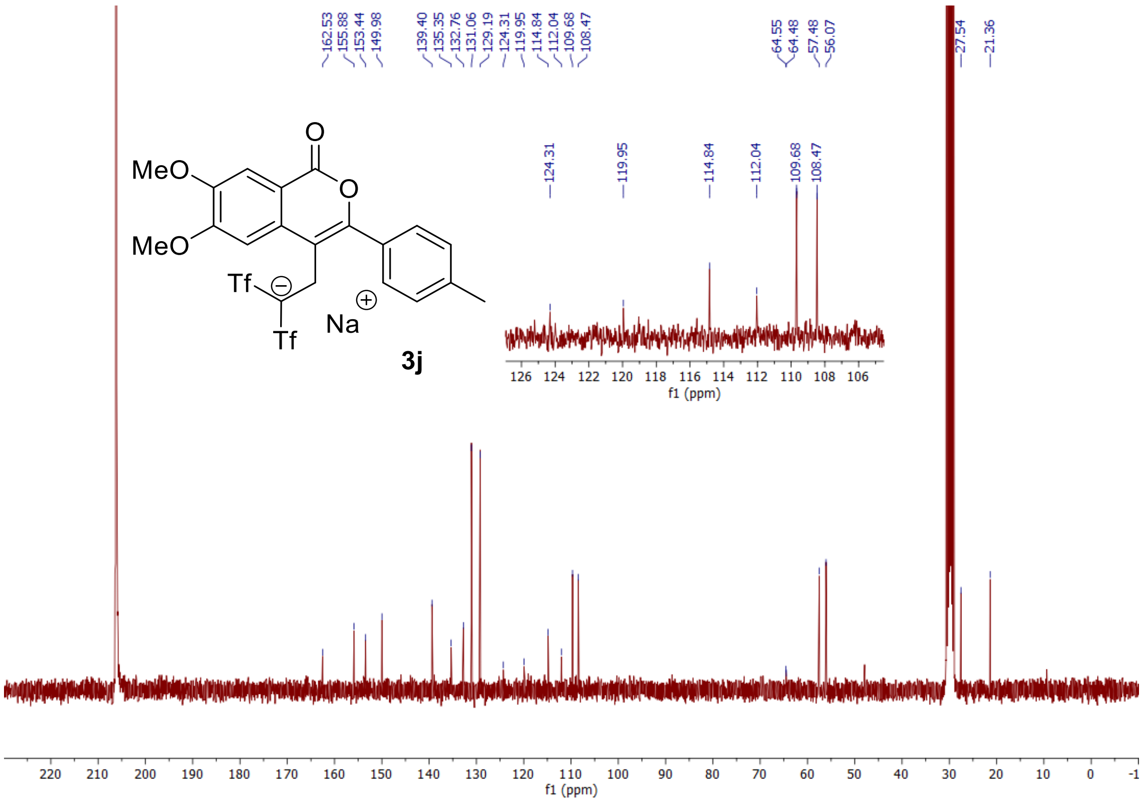

^{19}F NMR Compound **3j** (d_6-acetone, 282 MHz, 25 °C)

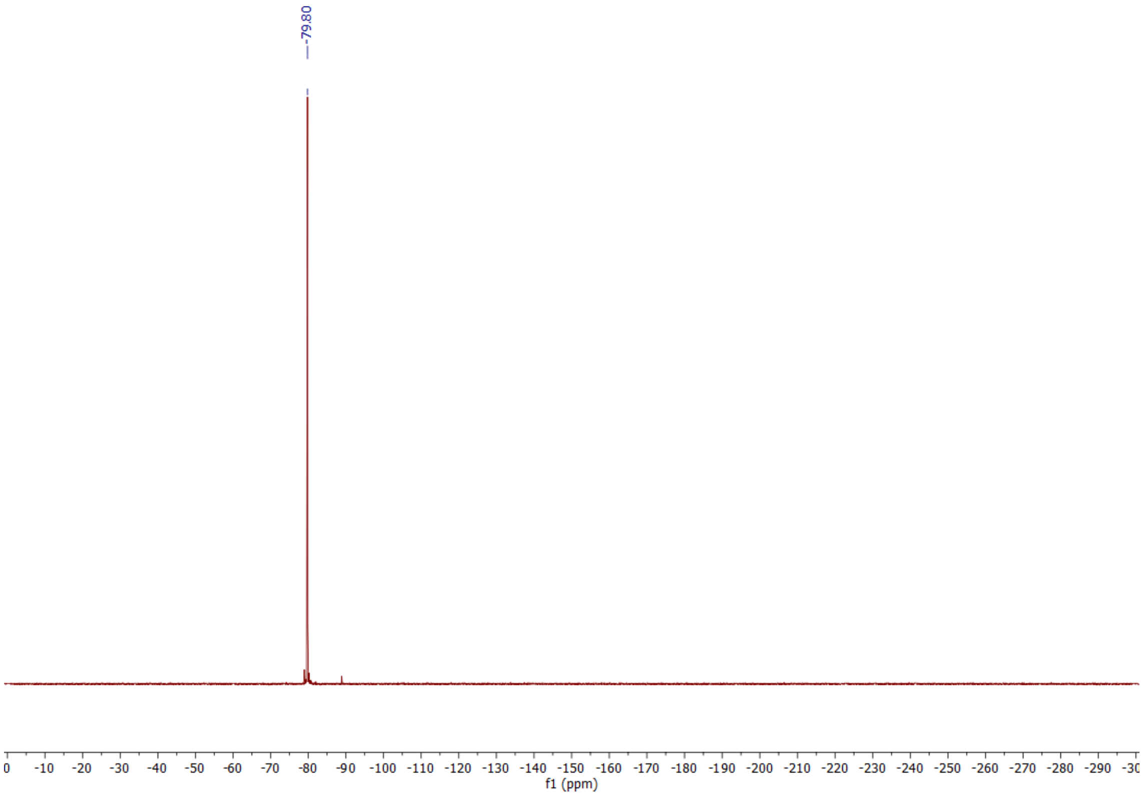

^{1}H NMR Compound **3k** (d_6-acetone, 300 MHz, 25 °C)

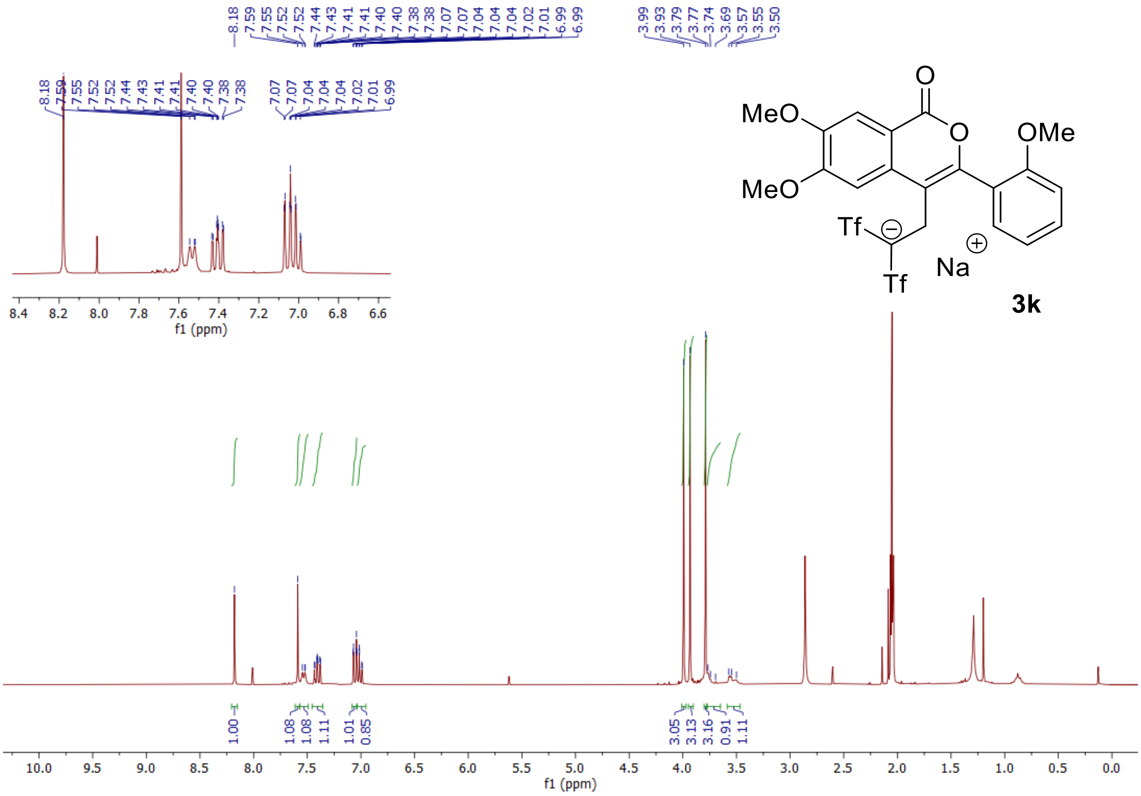

^{13}C NMR Compound **3k** (d_6-acetone, 75 MHz, 25 °C)

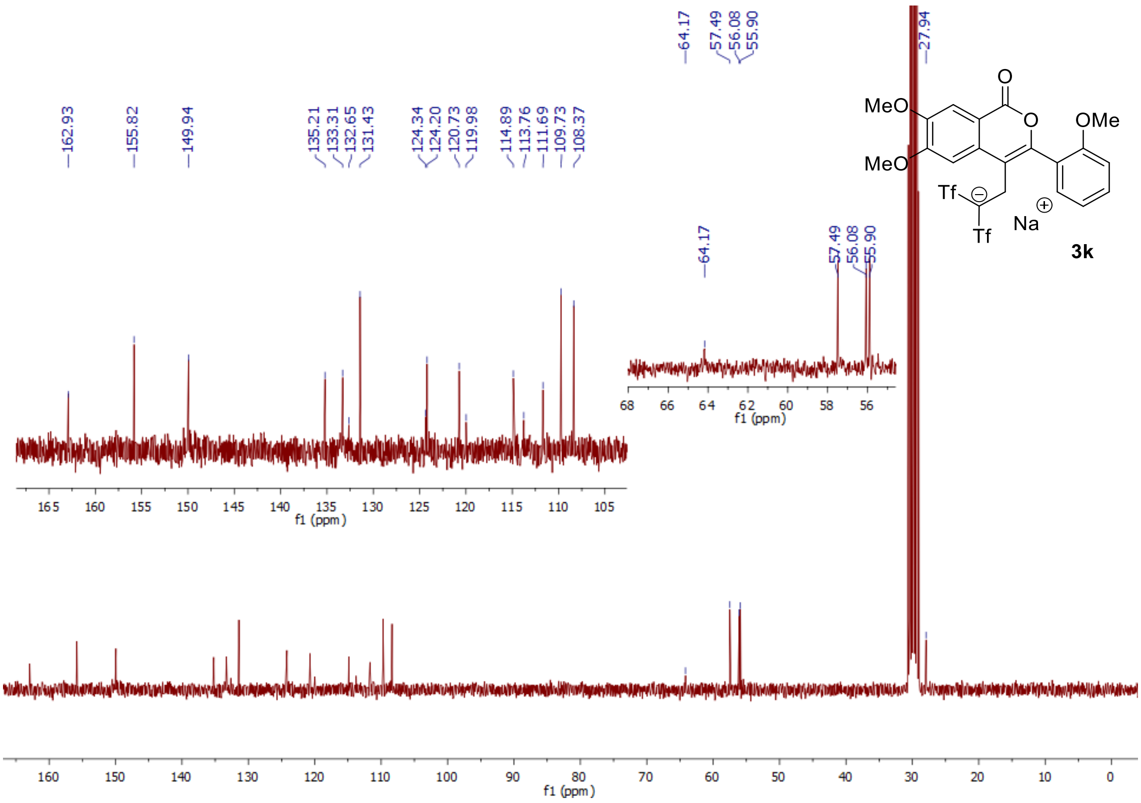

^{19}F NMR Compound **3k** (d_6-acetone, 282 MHz, 25 °C)

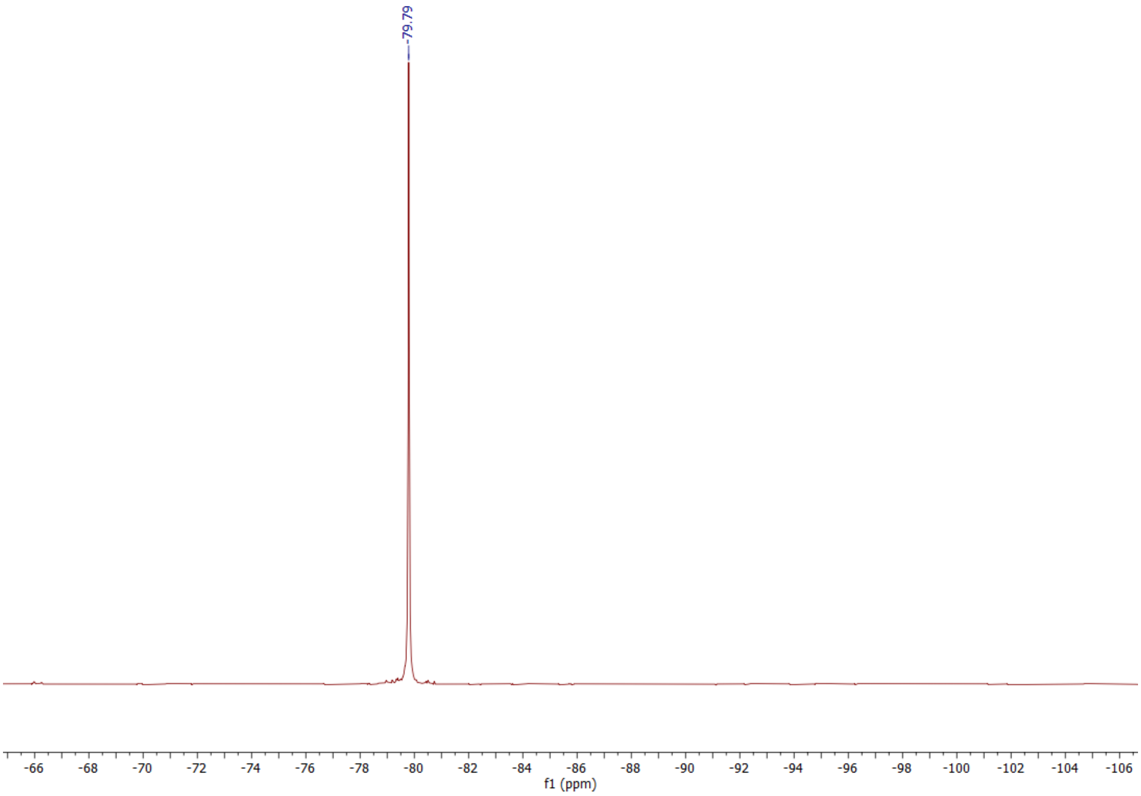

^{1}H – ^{13}C HMQC compound **3k**

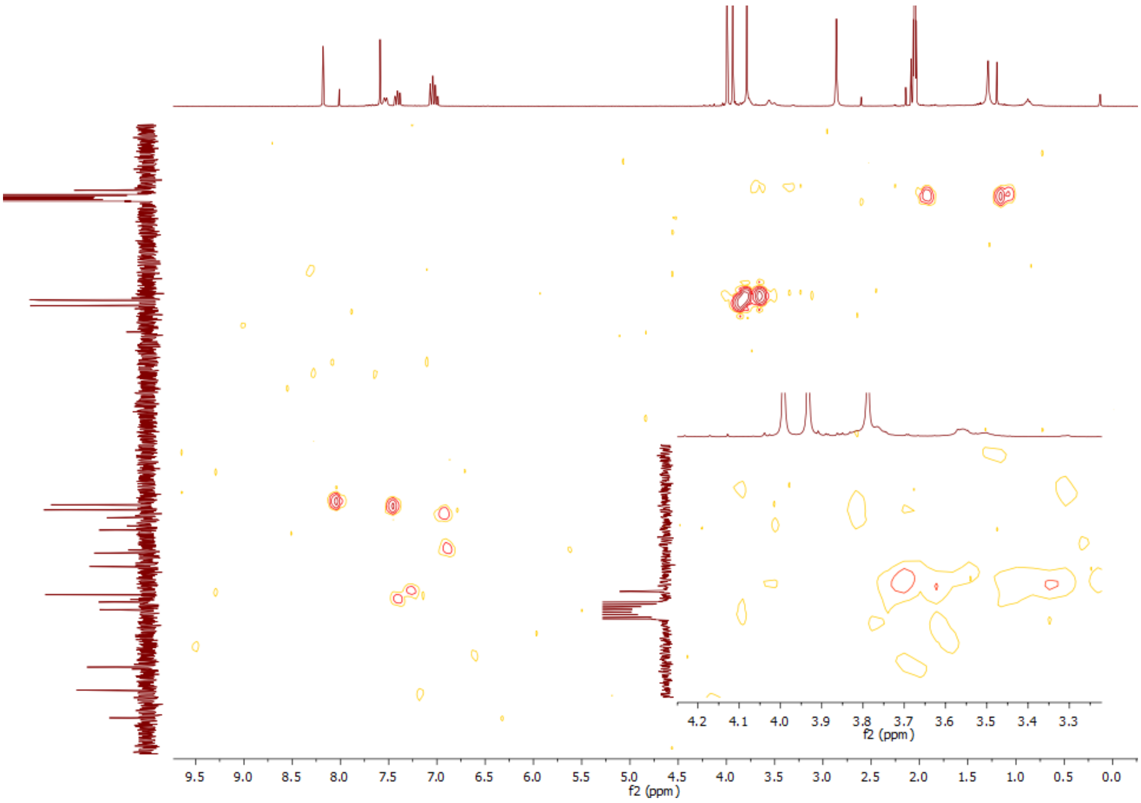

^{1}H NMR Compound **3l** (d_6-acetone, 300 MHz, 25 °C)

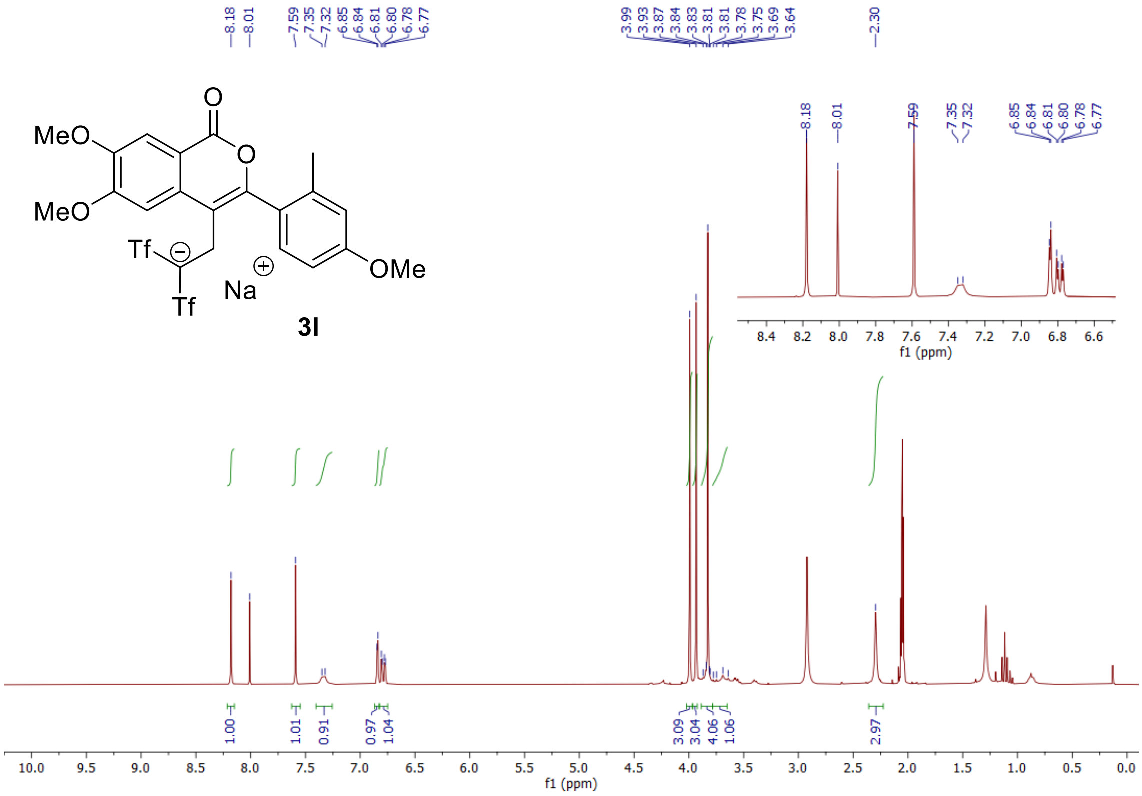

^{13}C NMR Compound **3l** (d_6-acetone, 75 MHz, 25 °C)

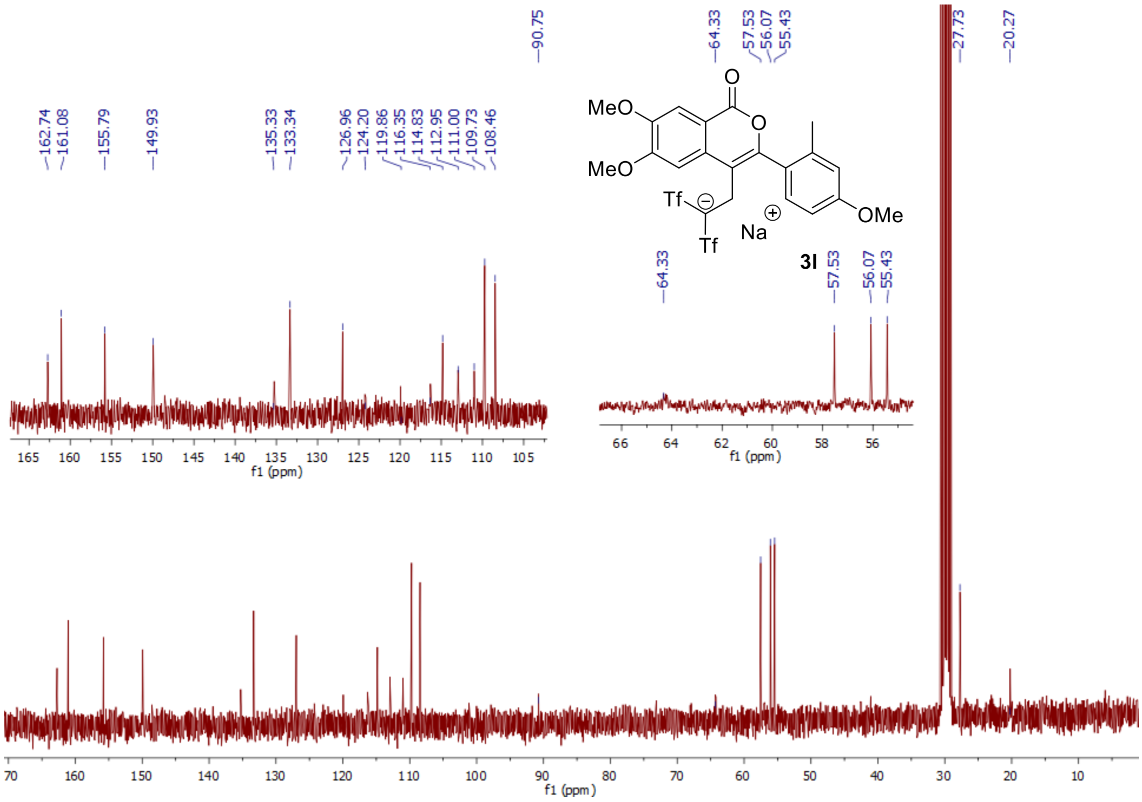

^{19}F NMR Compound **3l** (d_6-acetone, 282 MHz, 25 ^{o}C)

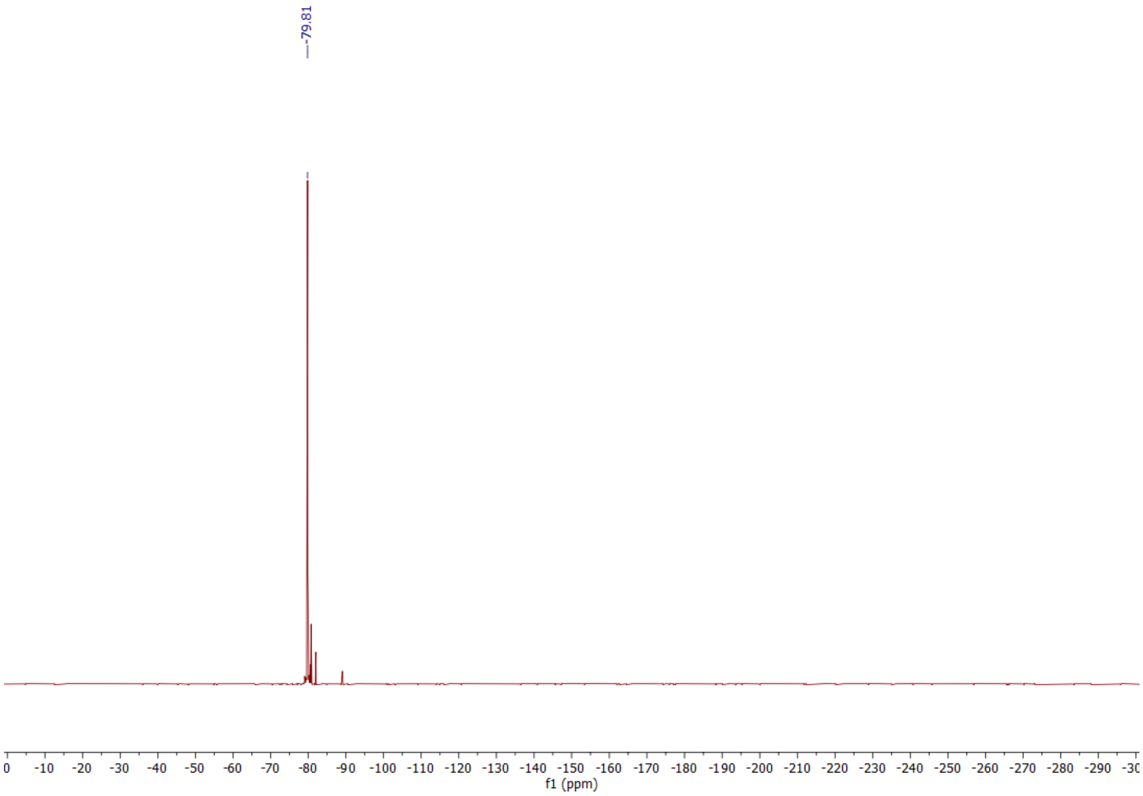

^{1}H NMR Compound **3m** (d_6-acetone, 300 MHz, 25 °C)

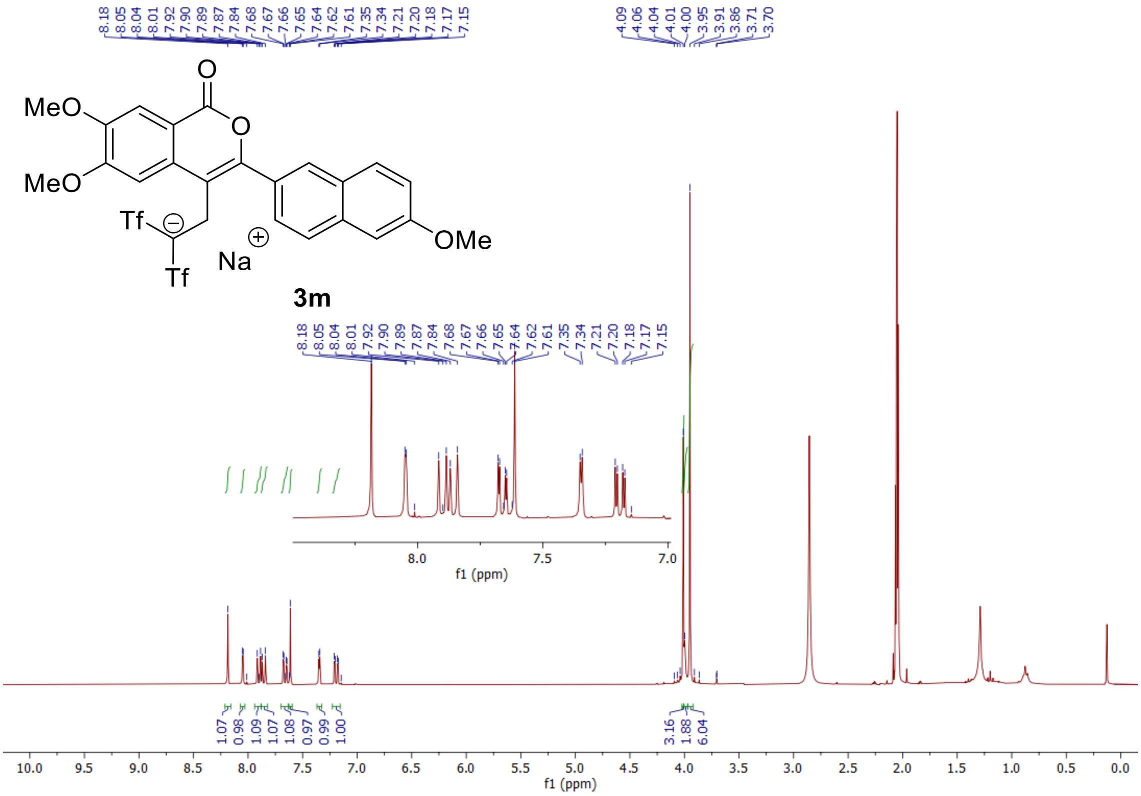

^{13}C NMR Compound **3m** (d_6-acetone, 75 MHz, 25 °C)

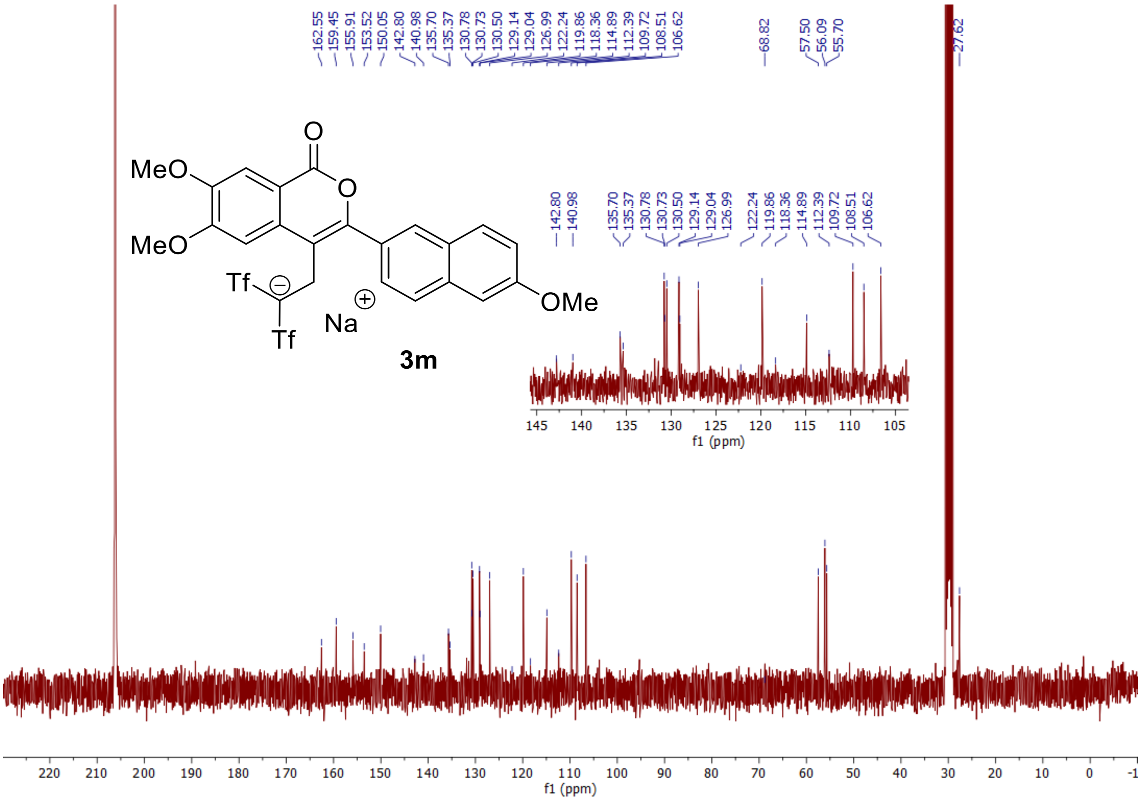

^{19}F NMR Compound **3m** (d_6-acetone, 282 MHz, 25 °C)

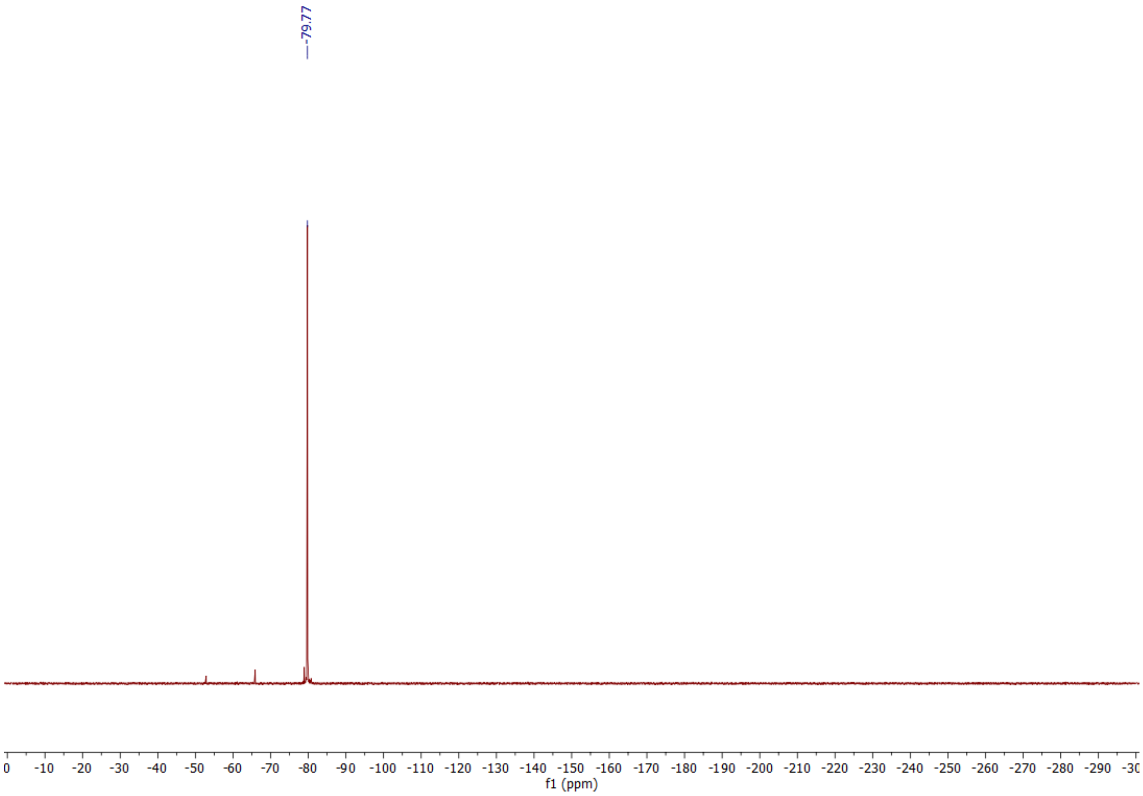

^{1}H NMR Compound **3n** (d_6-acetone, 300 MHz, 25 °C)

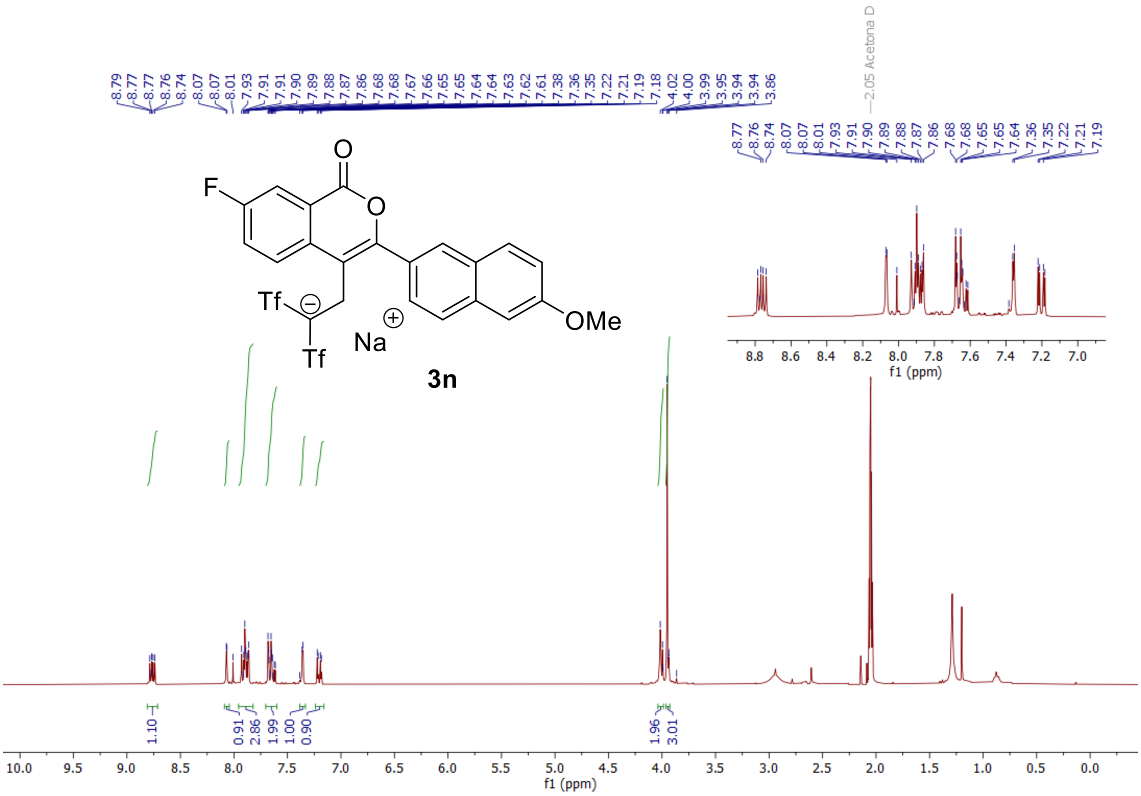

^{13}C NMR Compound **3n** (d_6-acetone, 75 MHz, 25 °C)

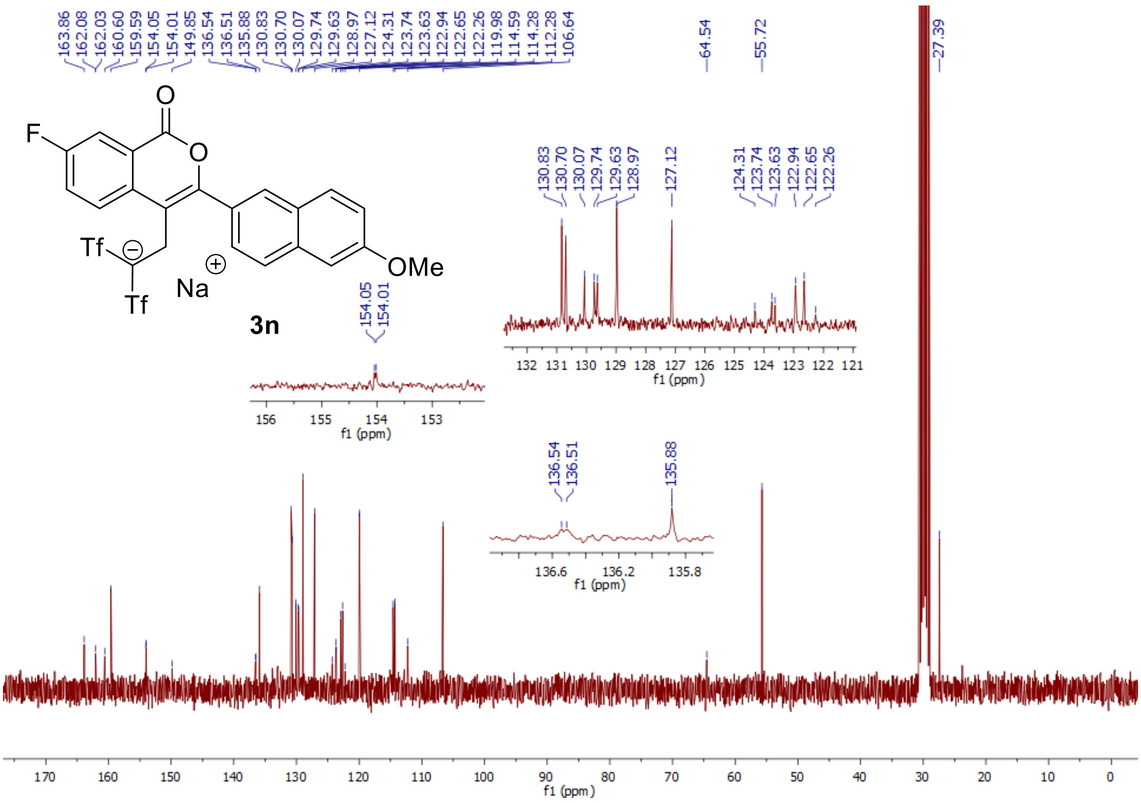

^{19}F NMR Compound **3n** (d_6-acetone, 282 MHz, 25 °C)

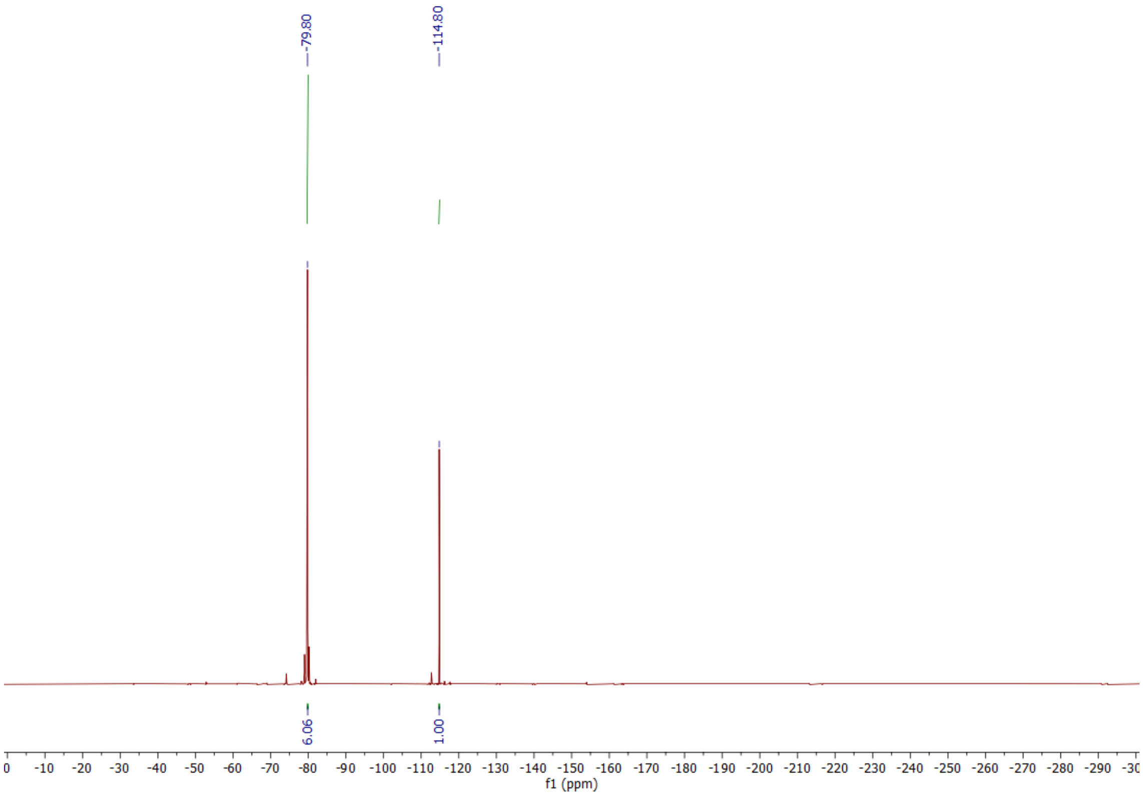

^{1}H NMR Compound **3o** (d_6-acetone, 300 MHz, 25 °C)

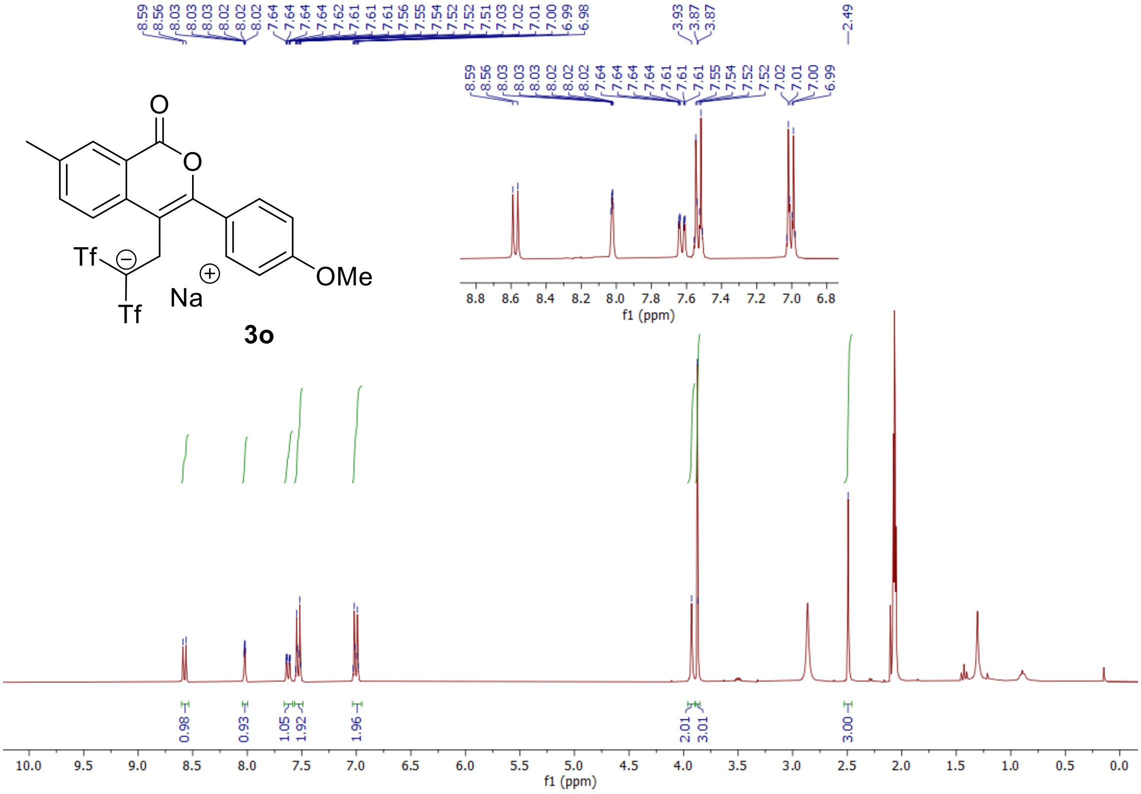

^{13}C NMR Compound **3o** (d_6-acetone, 75 MHz, 25 °C)

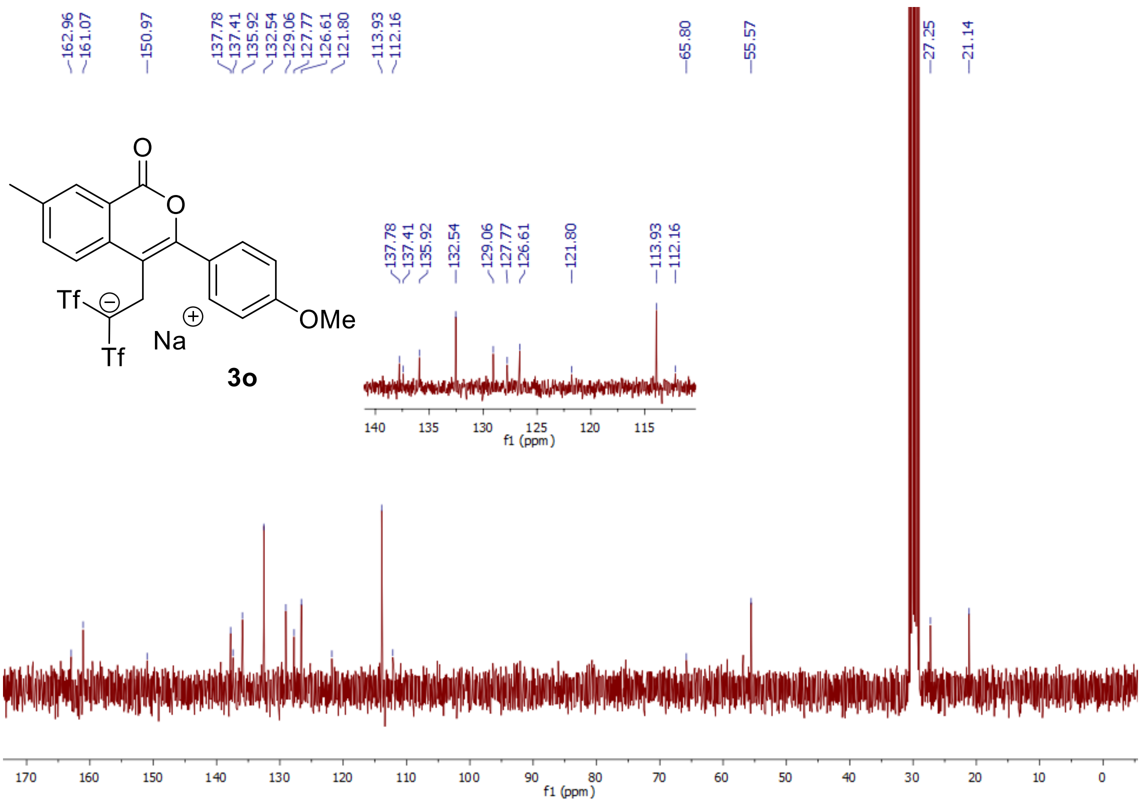

^{19}F NMR Compound **3o** (d_6-acetone, 282 MHz, 25 ^{o}C)

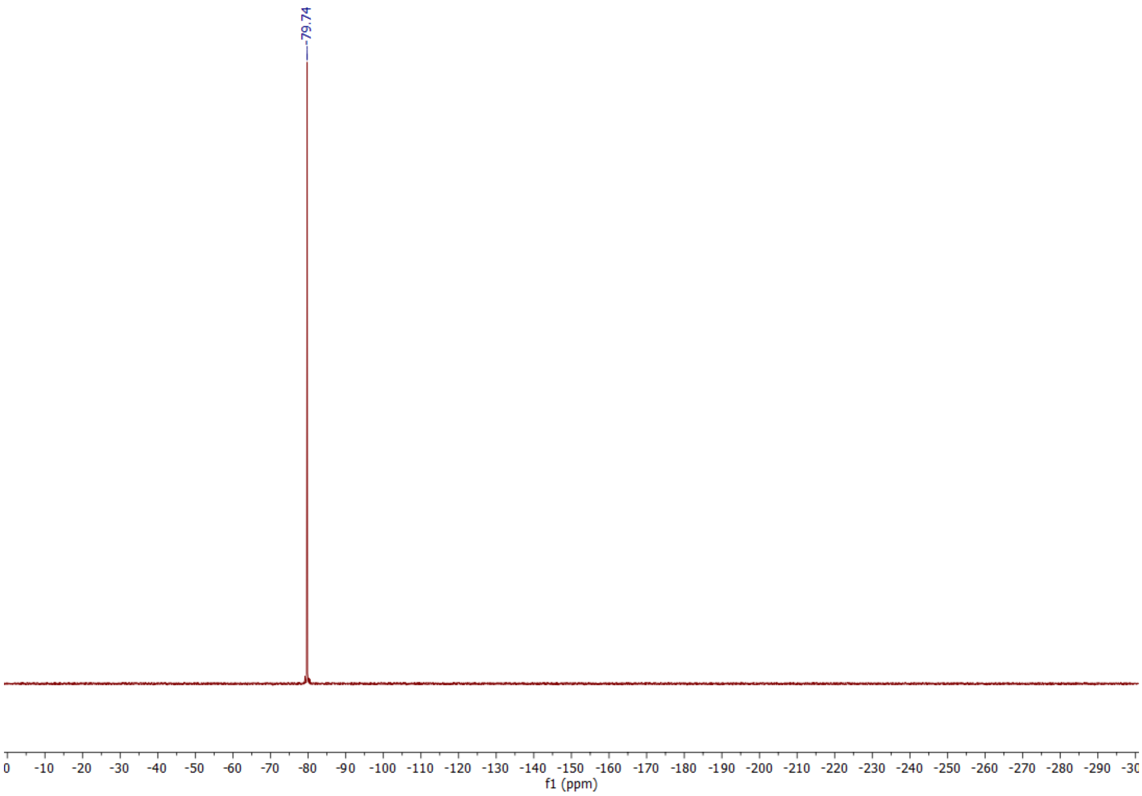

^{1}H NMR Compound **3p** (d_6-acetone, 300 MHz, 25 °C)

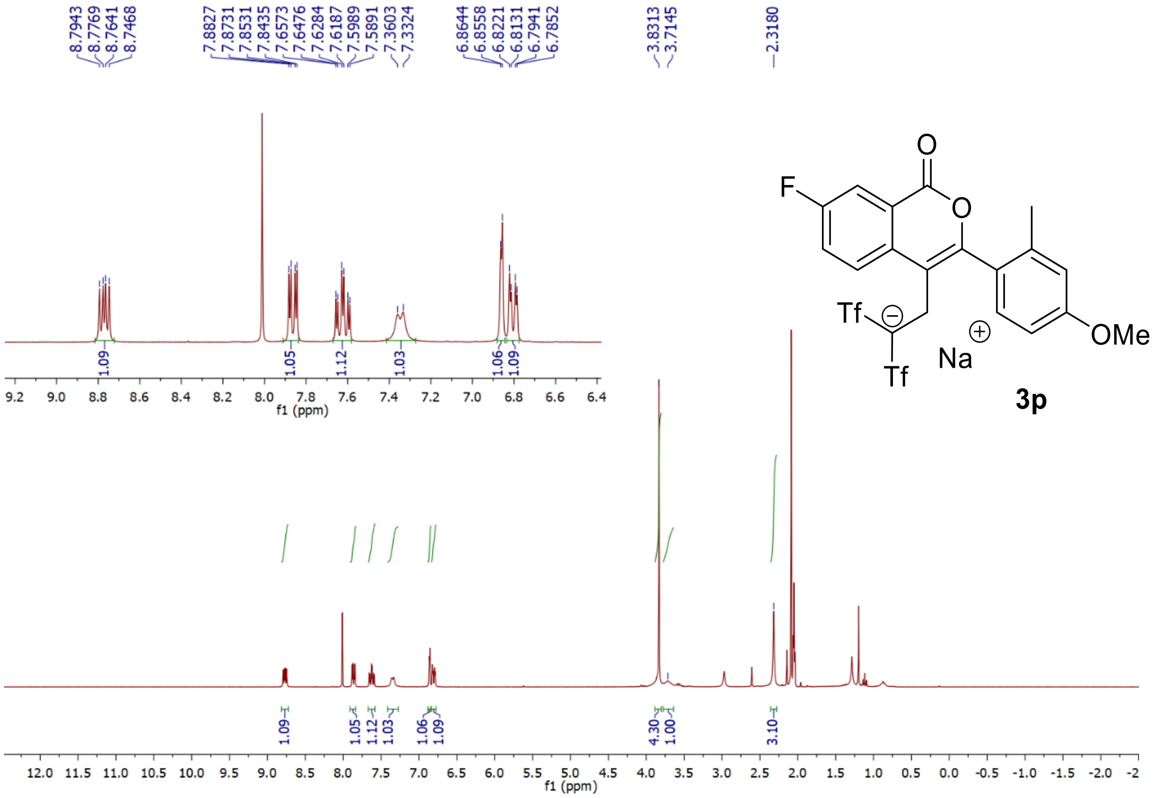

^{13}C NMR Compound **3p** (d_6-acetone, 75 MHz, 25 °C)

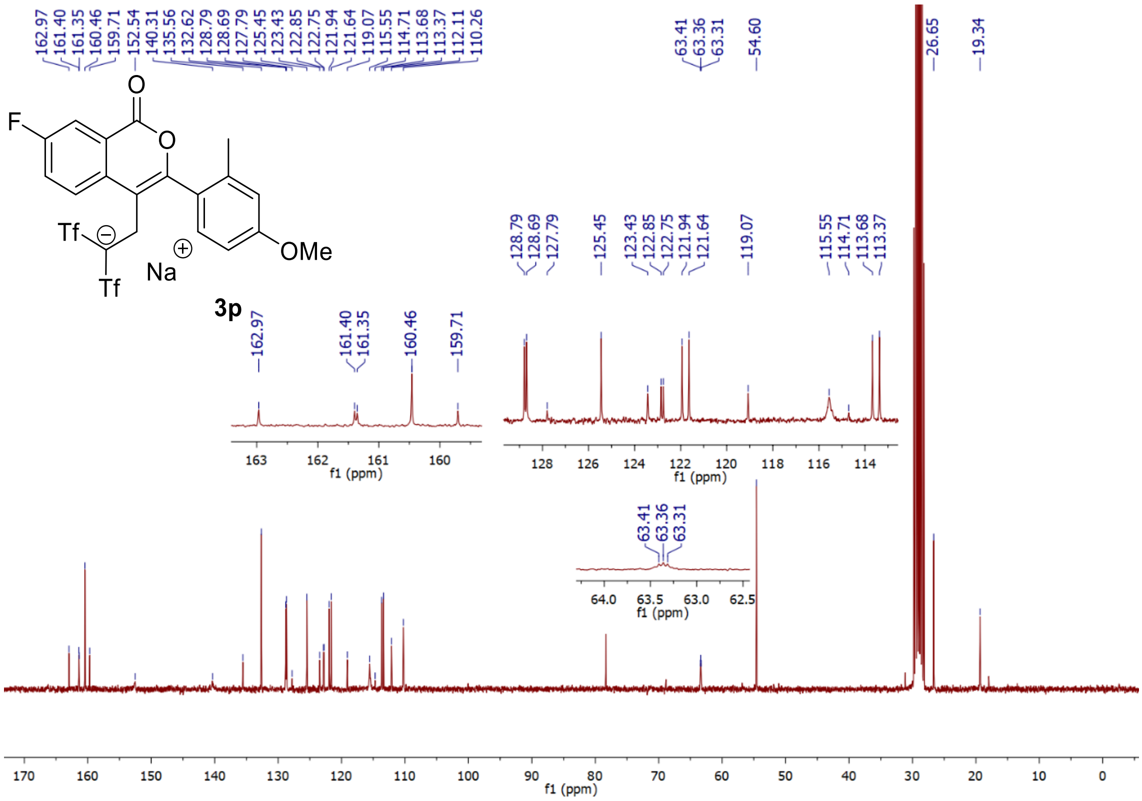

^{19}F NMR Compound **3p** (d_6-acetone, 282 MHz, 25 ^{o}C)

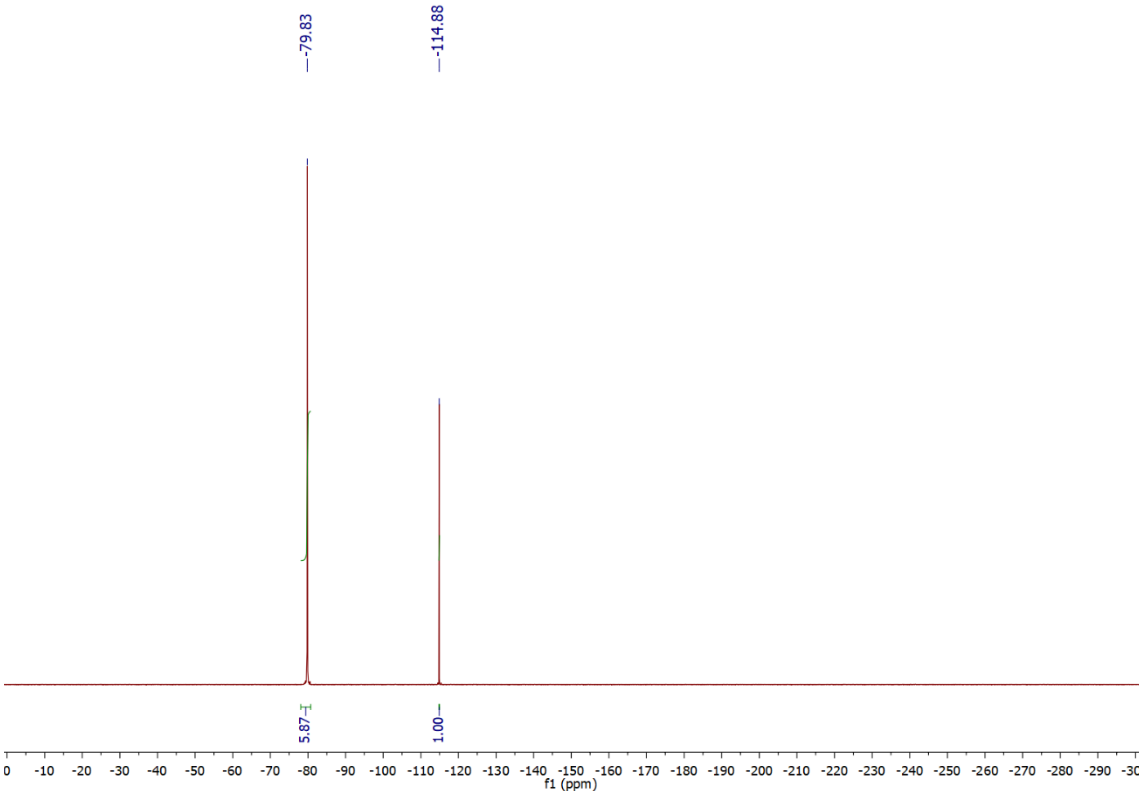

^{1}H NMR Compound **3q** (d_6-acetone, 300 MHz, 25 °C)

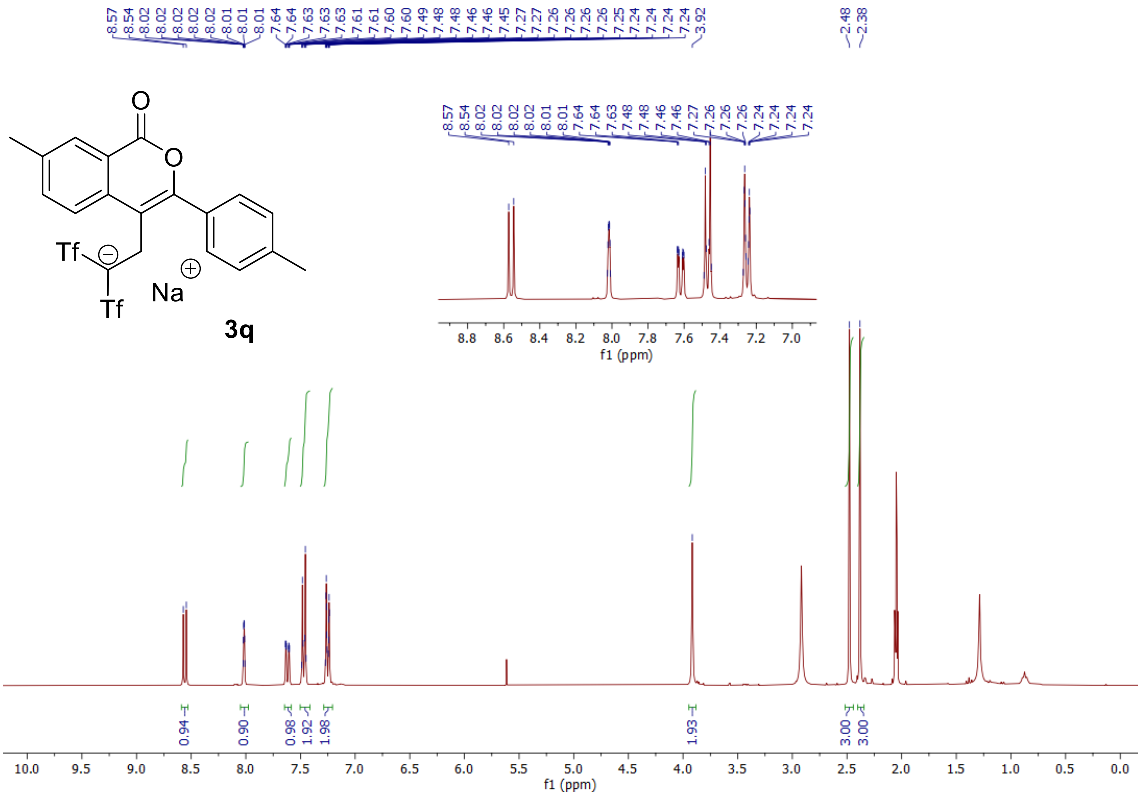

^{13}C NMR Compound **3q** (d_6-acetone, 75 MHz, 25 °C)

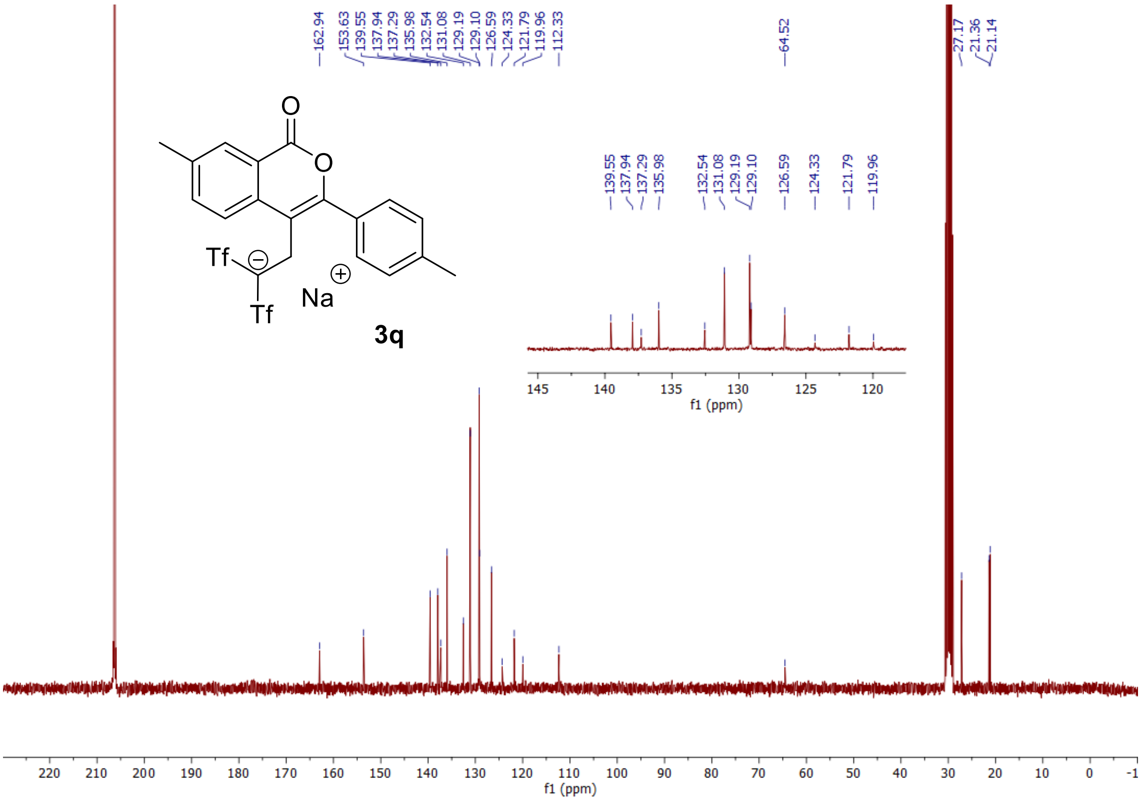

$^{1}H-^{1}H$ COSY Compound **3q**

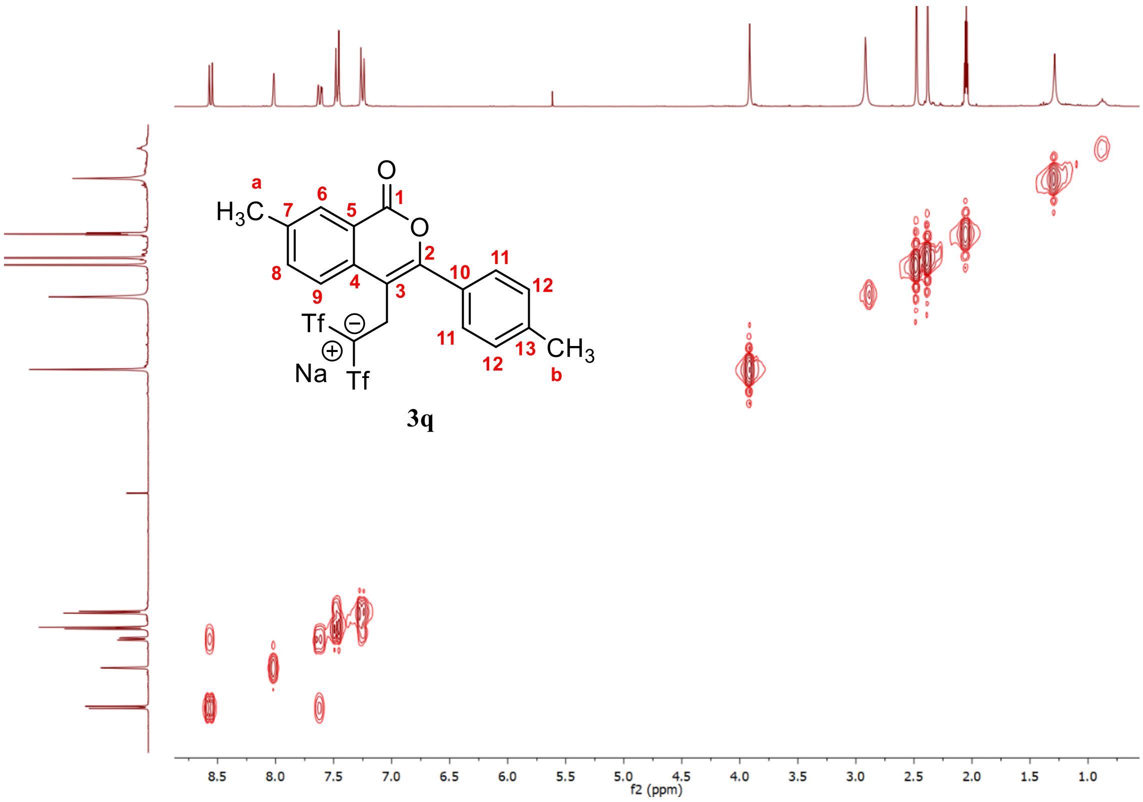

Table S9

#H	δ (ppm)	Multiplicity	Coupling with...	J (Hz)
**H-9**	8.56	d	H-8	8.3
**H-6**	8.02	s_{br}	H-8	2.1
**H-8**	7.62	ddd	H-9	8.3
			H-6	2.1
**H-11**	7.47	d	H-12	7.9
**H-12**	7.25	d	H-11	7.9
**CH_2**	3.92	s	-	-
**CH_3-a**	2.48	s	-	-
**CH_3-b**	2.38	s	-	-

^1H – ^{13}C HMQC Compound **3q**

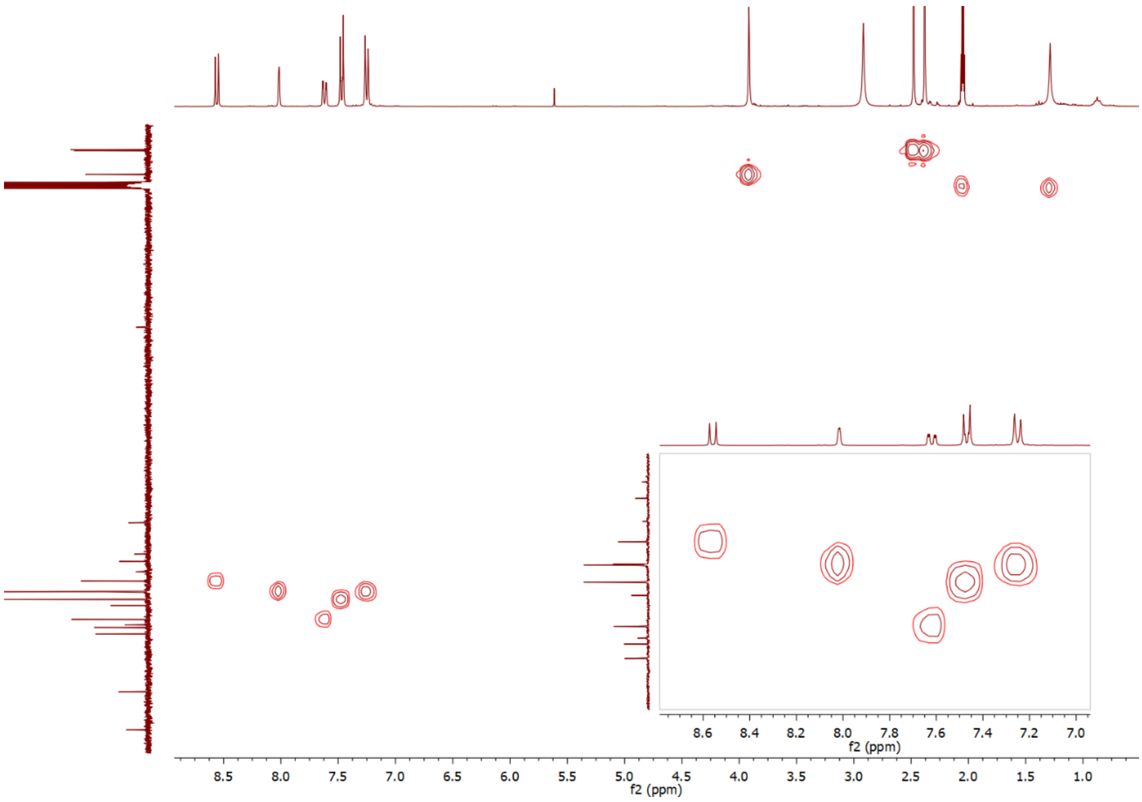

^1H – ^{13}C HMBC Compound **3q**

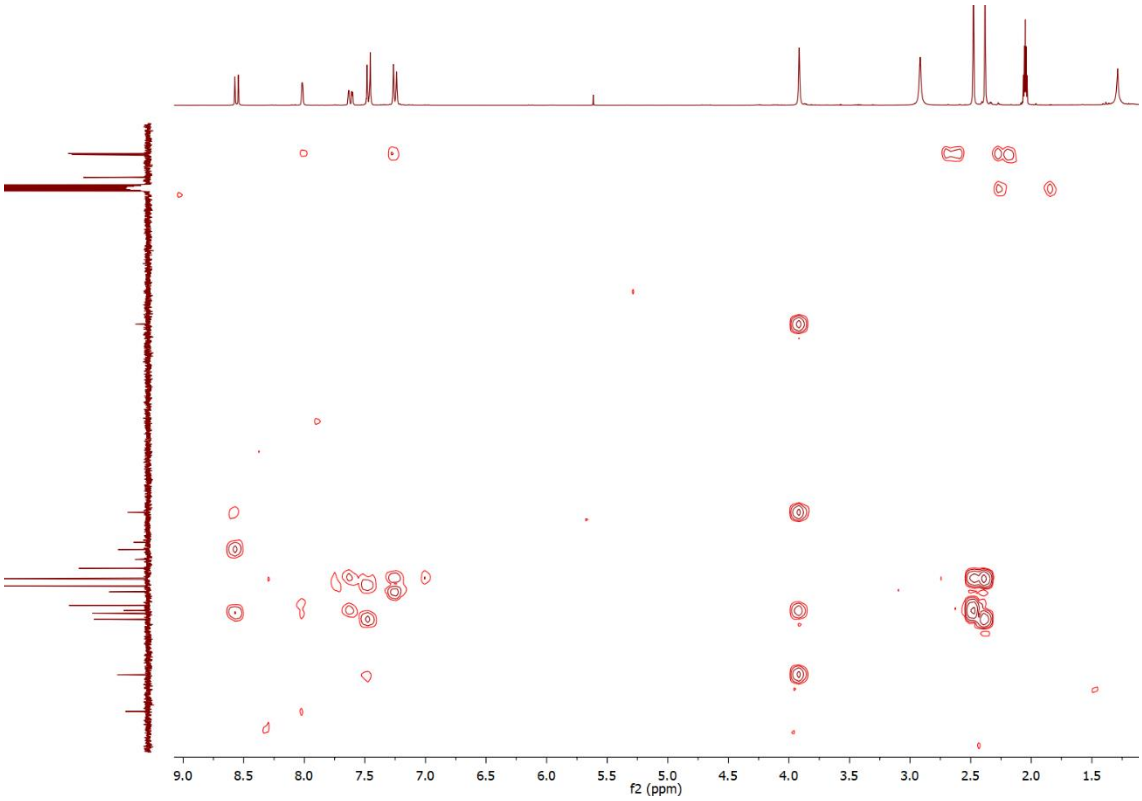

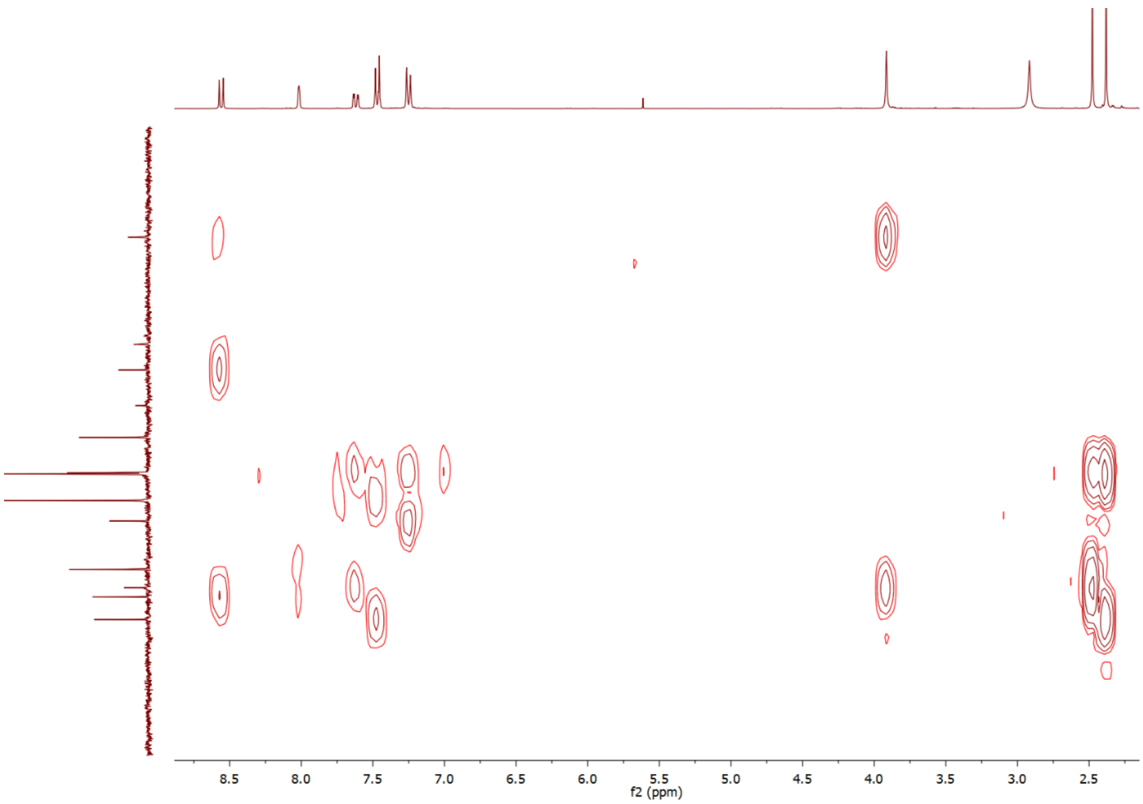

Table S10

#C	δ (ppm)		HMBC coupling	
**C-1**	162.9	C=O	H-6	Fig. 1
**C-2**	153.6	C_{Ar}	H-11 (^{3}J), CH_2 (^{3}J)	Fig. 1
**C-13**	139.6	C_{Ar}	H-11 (^{3}J), CH_3-b (^{2}J)	Fig. 2
**C-7**	137.9	C_{Ar}	H-9 (^{3}J), H-6 (^{2}J)	Fig. 2
**C-4**	137.3	C_{Ar}	H-8 (^{3}J), CH_2 (^{3}J), CH_3-a (^{5}J)	Fig. 3
**C-8**	136.0	CH_{Ar}	H-6 (^{3}J)	Fig. 1
**C-10**	132.5	C_{Ar}	H-12(^{3}J)	Fig. 3
**C-11**	131.1	CH_{Ar}	-	-
**C-12**	129.2	CH_{Ar}	CH_3-b (^{3}J)	Fig. 1
**C-6**	129.1	CH_{Ar}	H-8 (^{3}J), CH_3 -a (^{3}J)	Fig. 1
**C-9**	126.6	CH_{Ar}	-	-
**CF_3**	122.0		-	-
**C-5**	121.8	C_{Ar}	H-9 (^{3}J)	Fig. 4
**C-3**	112.3	C_{Ar}	H-9 (^{3}J), CH_2 (^{2}J)	Fig. 4
**$^{-}CTf_2$**	64.5		CH_2 (^{2}J)	Fig. 4
**CH_2**	27.2	CH	-	-
**CH_3-b**	21.4	CH	H-12 (^{3}J)	Fig. 4
**CH_3-a**	21.1	CH	H-6 (^{3}J), H-8 (^{3}J)	Fig. 4

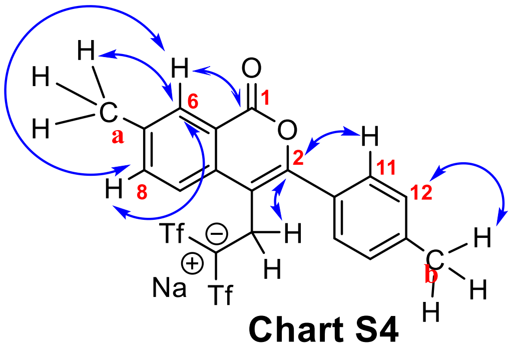

Chart S4

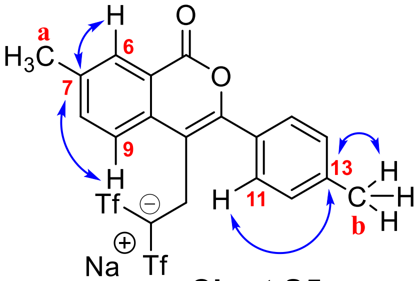

Chart S5

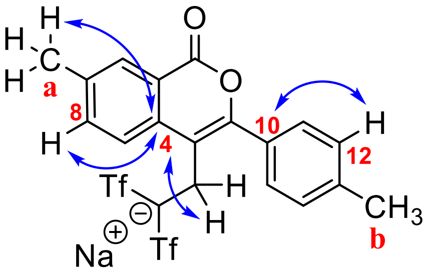

Chart S6

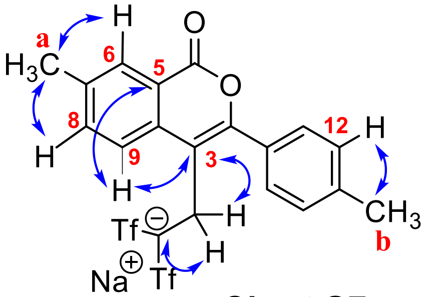

Chart S7

^{19}F NMR Compound **3q** (d_6-acetone, 282 MHz, 25 °C)

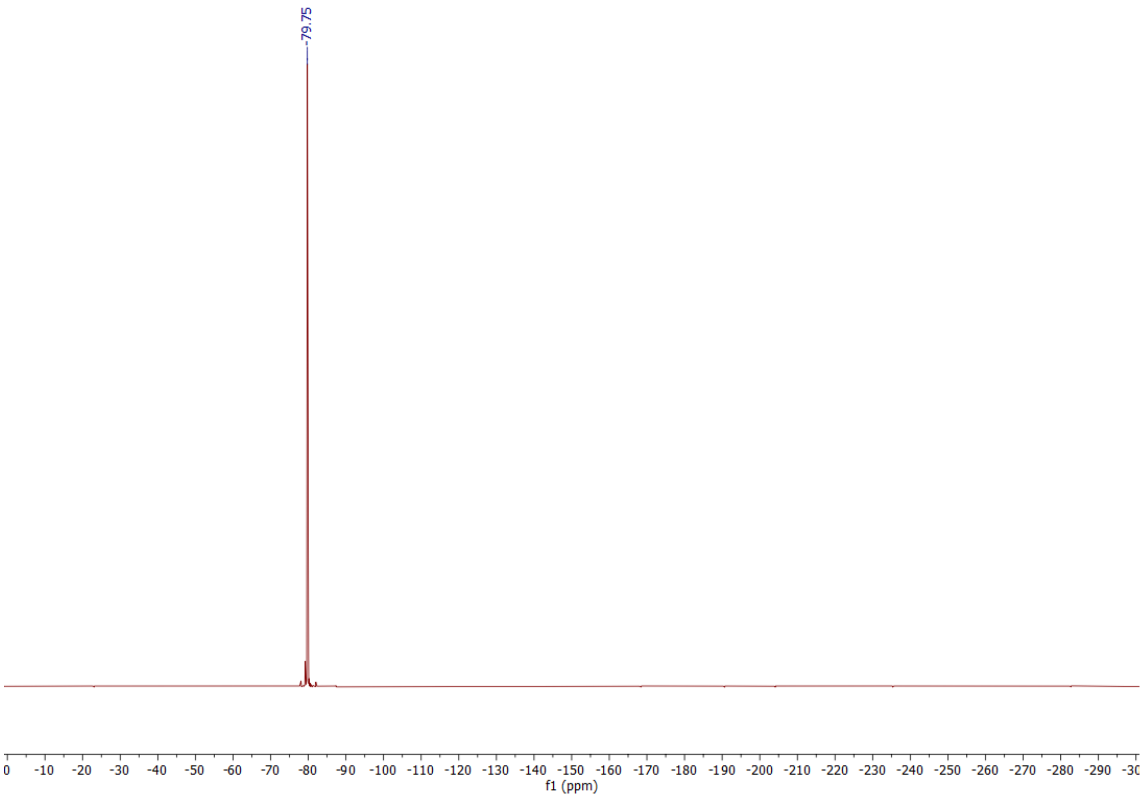

^{1}H NMR Compound **3r** (d_6-acetone, 300 MHz, 25 °C)

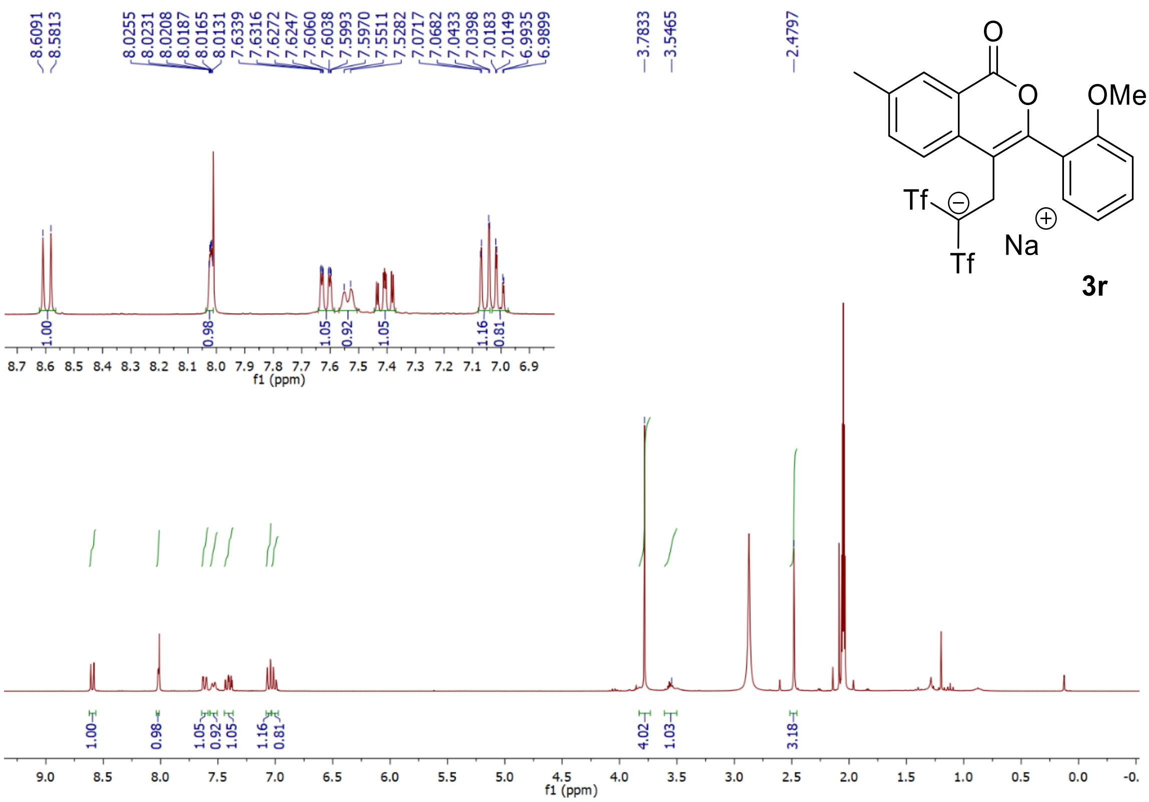

^{13}C NMR Compound **3r** (d_6-acetone, 75 MHz, 25 °C)

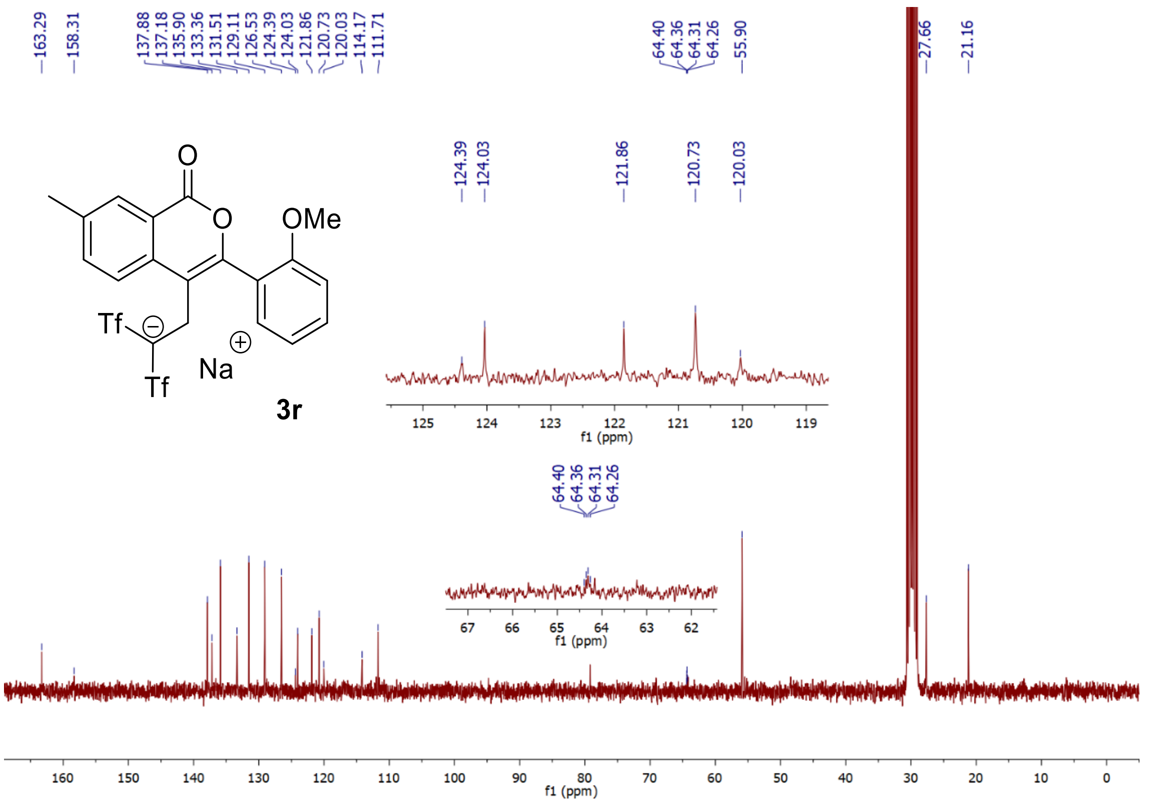

^{19}F NMR Compound **3r** (d_6-acetone, 282 MHz, 25 °C)

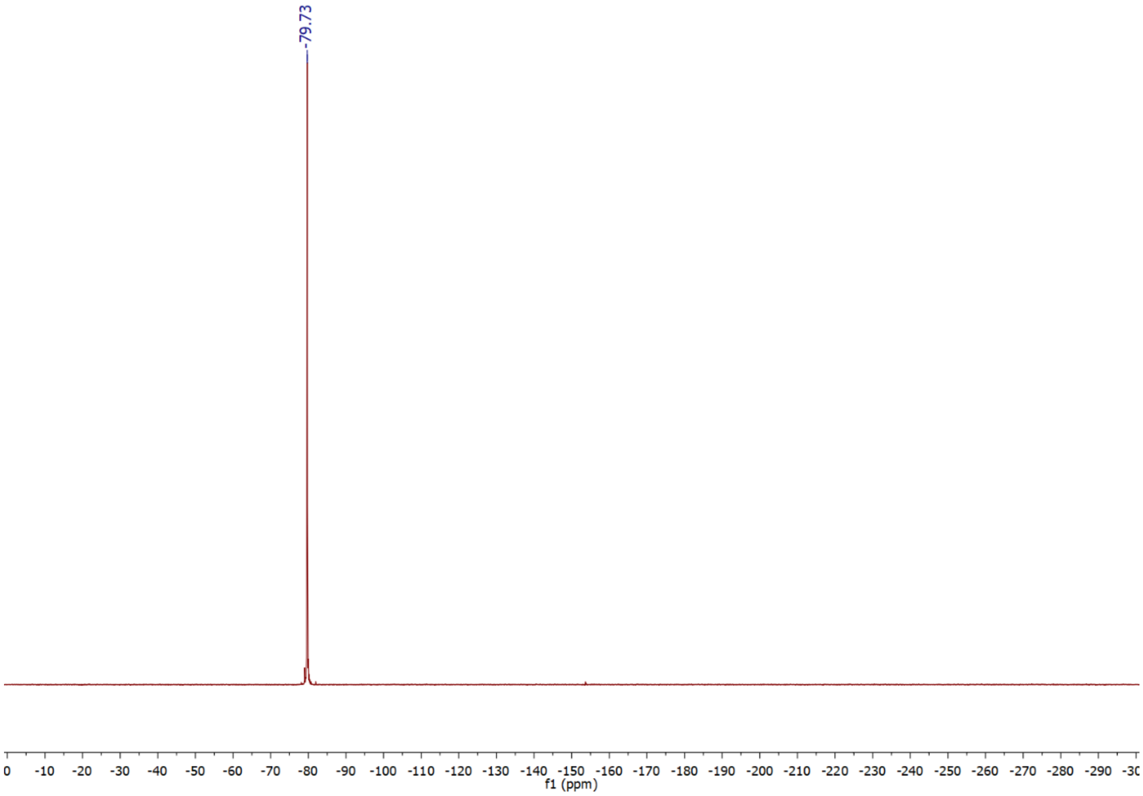

^{1}H NMR Compound **4s-*p*-CF₃** (CDCl₃, 300 MHz, 25 °C)

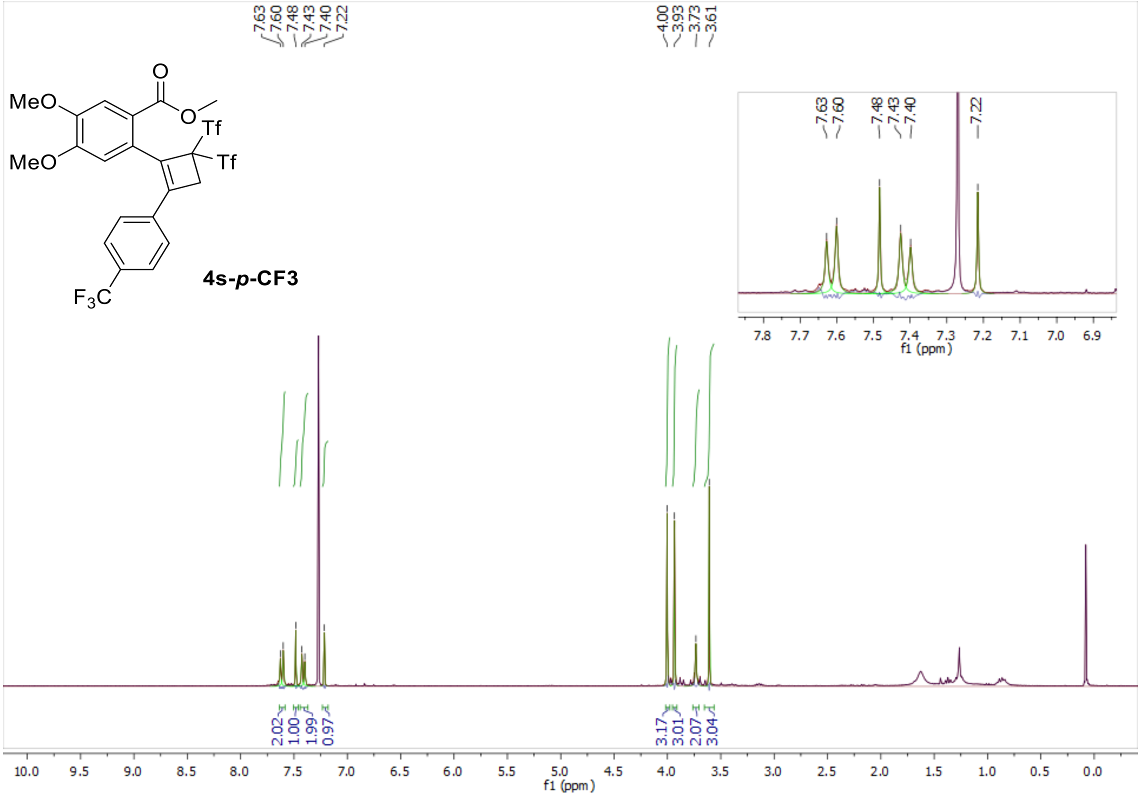

^{13}C NMR Compound **4s-*p*-CF₃** (CDCl₃, 175 MHz, 25 °C)

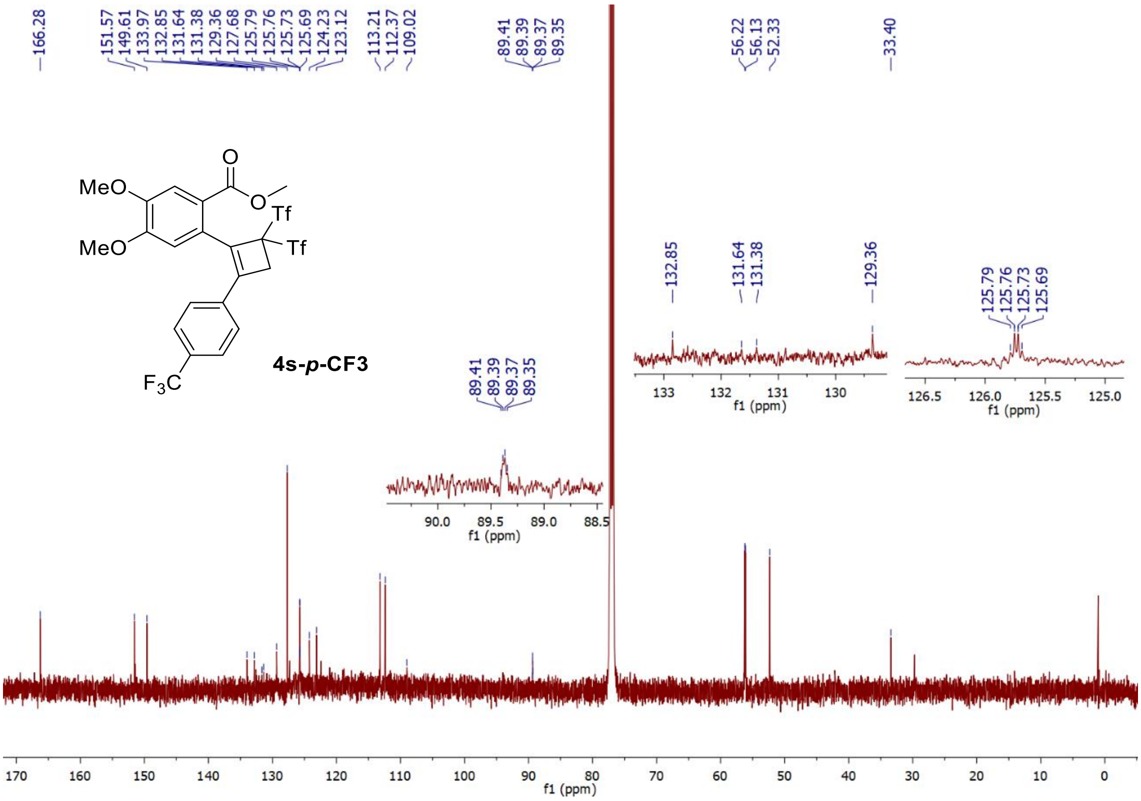

$^{1}H - ^{1}H$ COSY Compound **4s-*p*-CF$_3$**

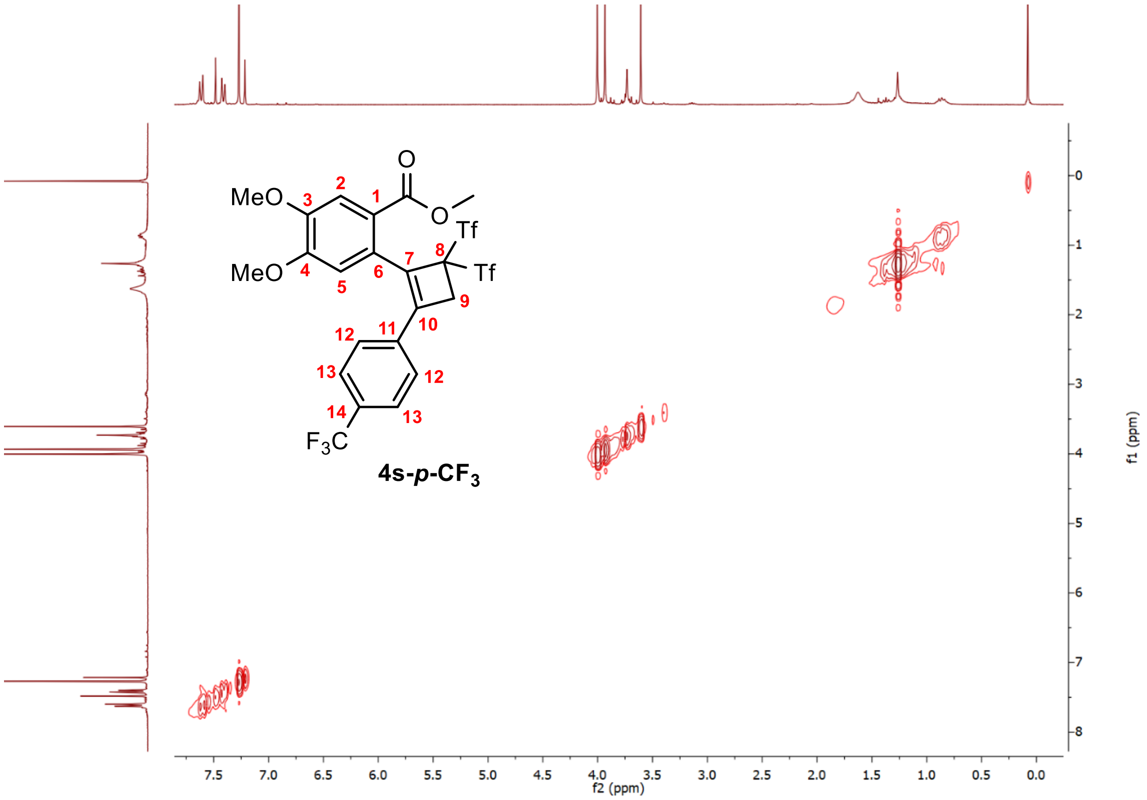

Table S11

#H	δ (ppm)	Multiplicity	Coupled with	J (Hz)
**H-13**	7.61	d	H-12	8.1
**H-2**	7.48	s	-	-
**H-12**	7.41	d	H-13	8.1
**H-5**	7.22	s	-	-
**OCH$_3$ (a)**	4.00	s	-	-
**OCH$_3$ (b)**	3.93	s	-	-
***CH$_2$*CTf$_2$**	3.73	s	-	-
**COO*CH$_3$***	3.61	s	-	-

$^{1}H - ^{13}C$ HMQC Compound **4s-*p*-CF$_3$**

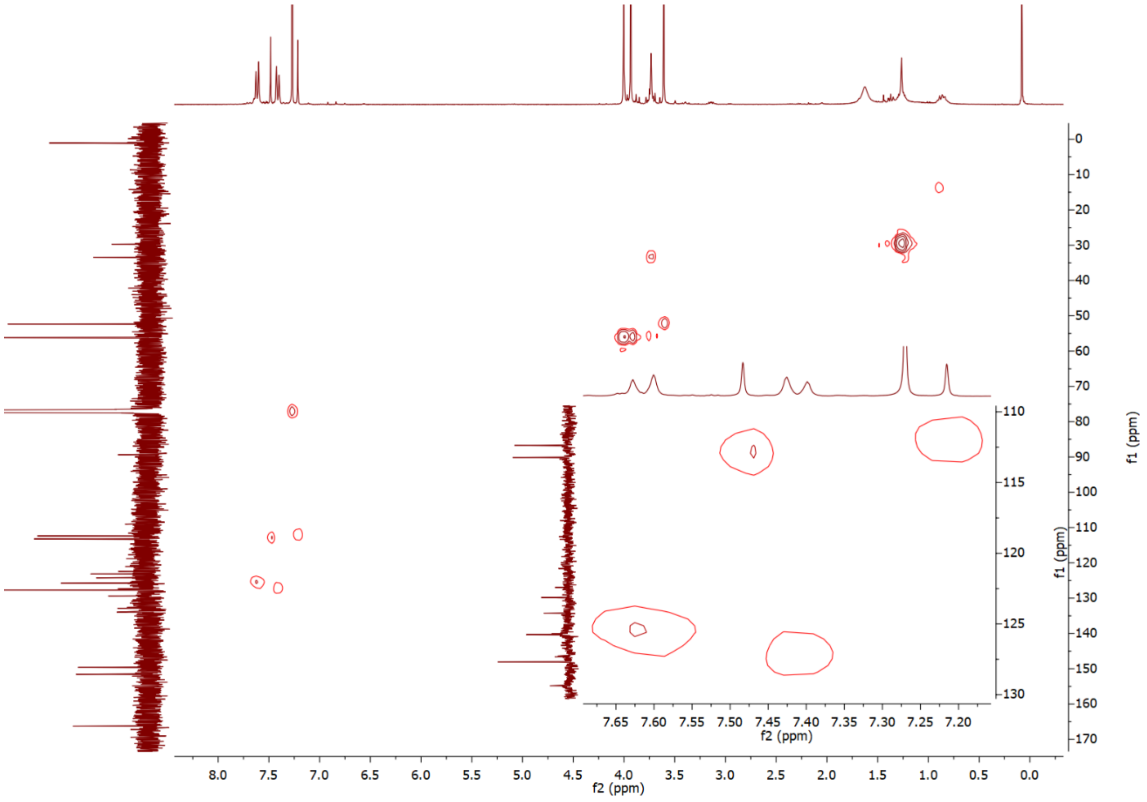

$^{1}H - ^{13}C$ HMBC Compound **4s-*p*-CF$_3$**

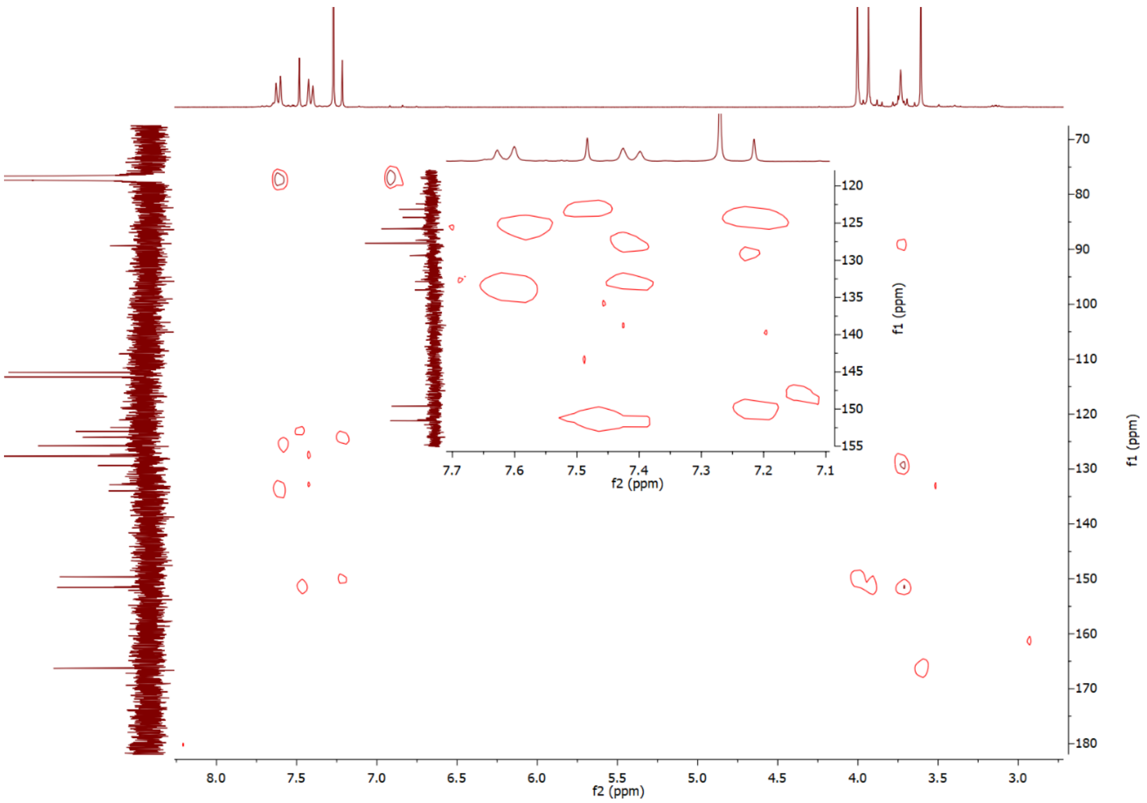

Table S12

#C	δ (ppm)		HMBC coupling	
**C=O**	166.3	C=O	CO*CH_3*	Fig. 1
**C-4**	151.6	C_{Ar}	H-2 (^{3}J), CH_3 - b (^{3}J)	Fig. 1
**C-3**	149.6	C_{Ar}	H-5 (^{3}J), CH_3 - a (^{2}J)	Fig. 2
**C-11**	134.0	C_{Ar}	H-13 (^{3}J)	Fig. 2
**C-10**	132.9	*C*=C	H-12 (^{3}J)	Fig. 1
**C-14**	131.5	C_{Ar}	-	-
**C-7**	129.4	C=*C*	H-5 (^{3}J), H-9 (^{3}J)	Fig. 2
**C-12**	127.7	CH_{Ar}	-	-
**C-13**	125.7	CH_{Ar}	-	-
**C-1**	124.2	C_{Ar}	H-5 (^{3}J)	Fig. 2
**C-6**	123.1	C_{Ar}	H-2 (^{3}J)	Fig. 1
**C-2**	113.2	CH_{Ar}	-	-
**C-5**	112.4	CH_{Ar}	-	-
**C-8**	89.4	*C*Tf_2	H-9 (^{2}J)	Fig. 1
**CH_3 - a**	56.2	CH	-	-
**CH_3 - b**	56.1	CH	-	-
**CO*CH_3***	52.3	CH	-	-
**C-9**	33.4	CH_2	-	-

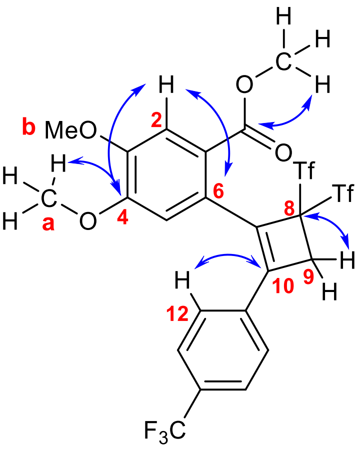

Chart S8

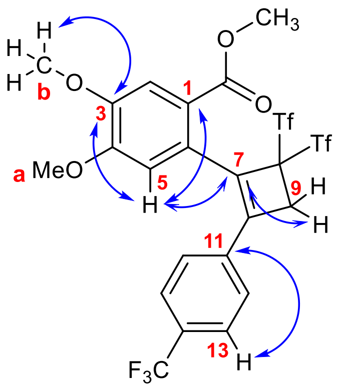

Chart S9

^{19}F NMR Compound **4s-*p*-CF₃** ($CDCl_3$, 282 MHz, 25 °C)

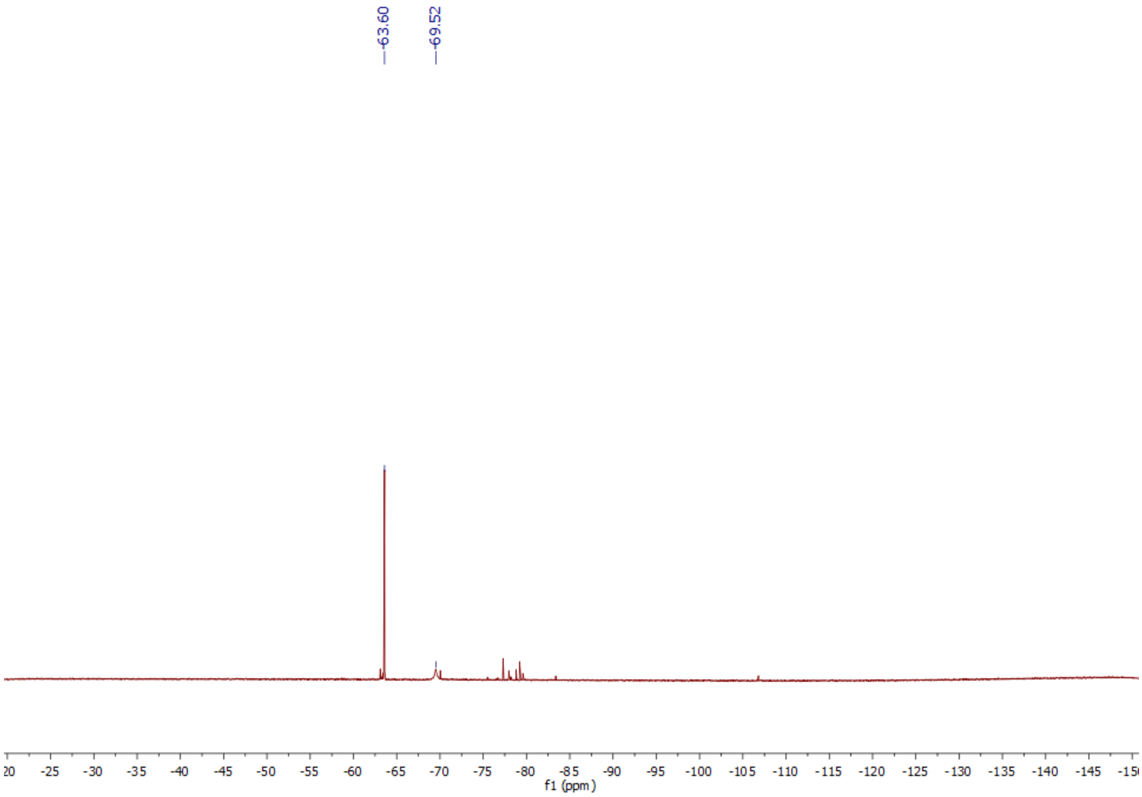

^{1}H NMR Compound **4s-*o*-CF$_3$** ($CDCl_3$, 300 MHz, 25 °C)

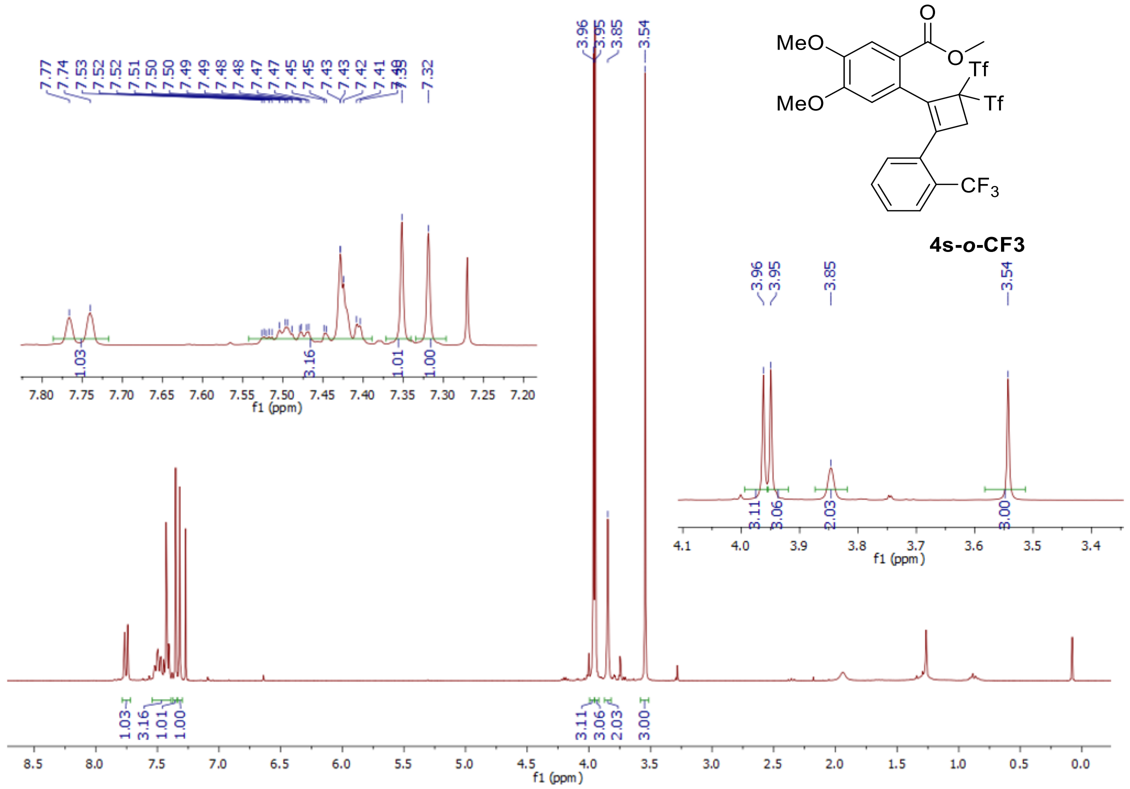

^{13}C NMR Compound **4s-*o*-CF$_3$** ($CDCl_3$, 175 MHz, 25 °C)

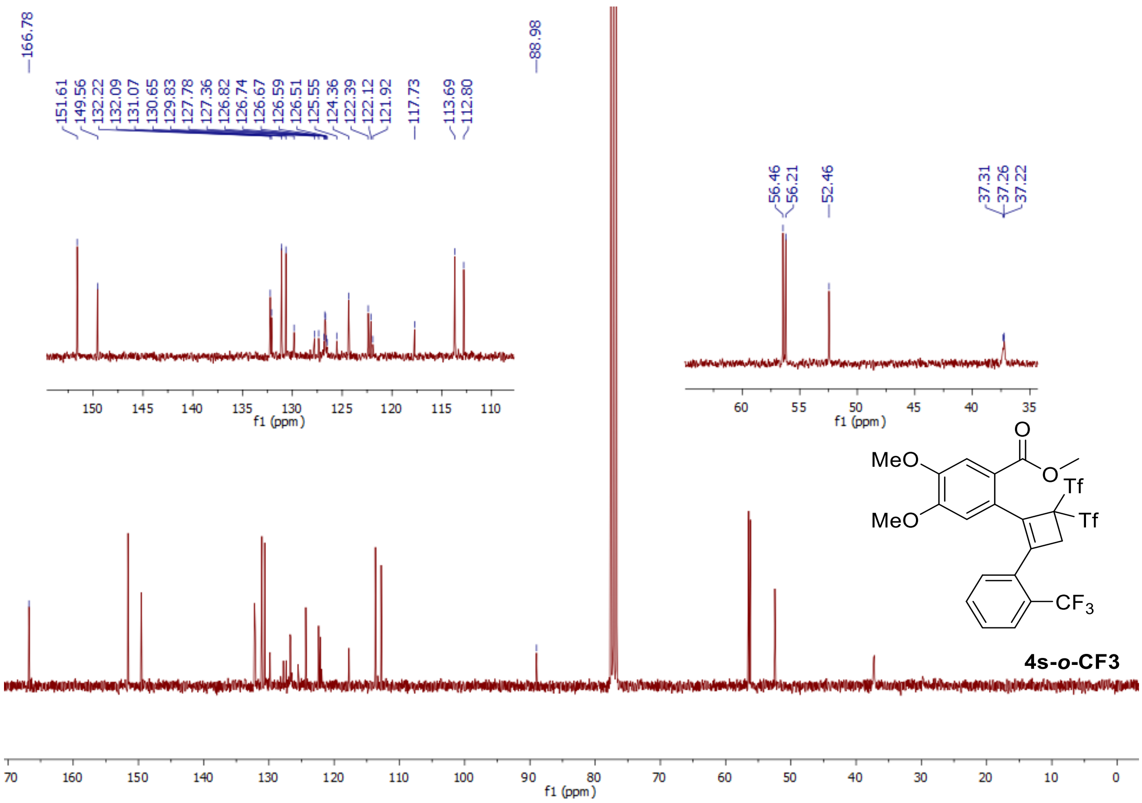

^{19}F NMR Compound **4s-*o*-CF_3** ($CDCl_3$, 282 MHz, 25 °C)

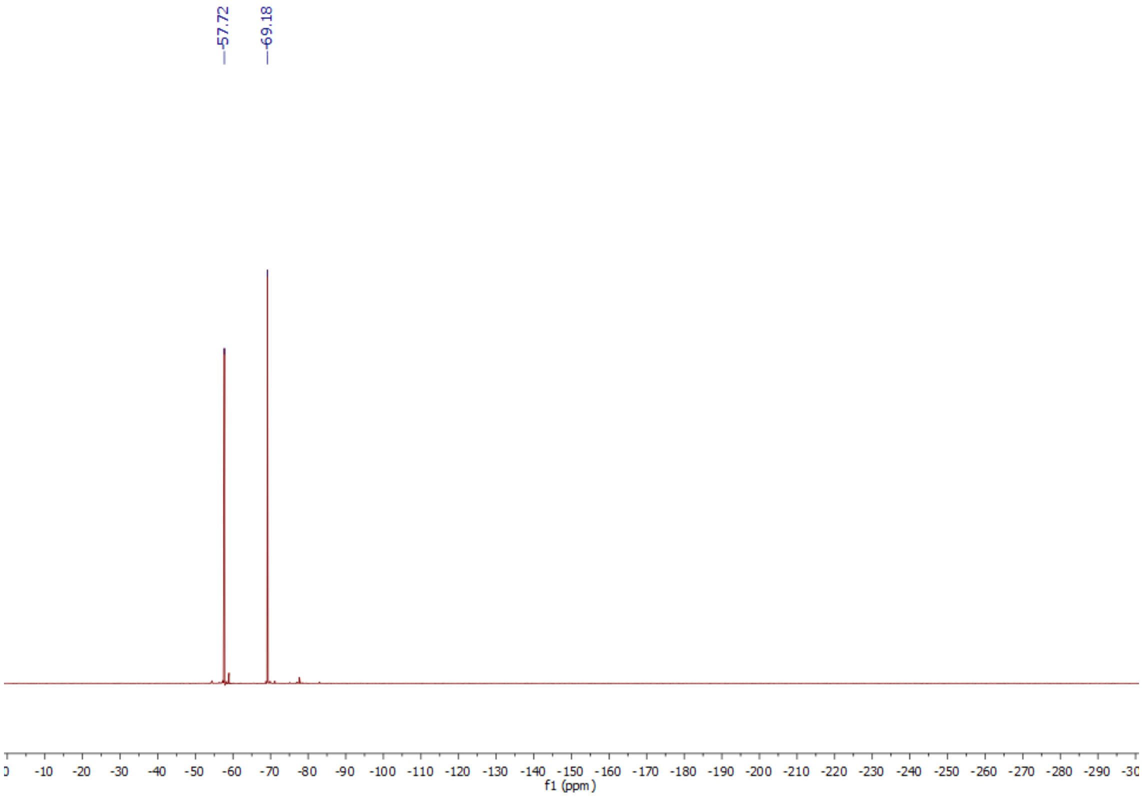

^{1}H NMR Compound [D]-**3i** (d_6-acetone, 300 MHz, 25 °C)

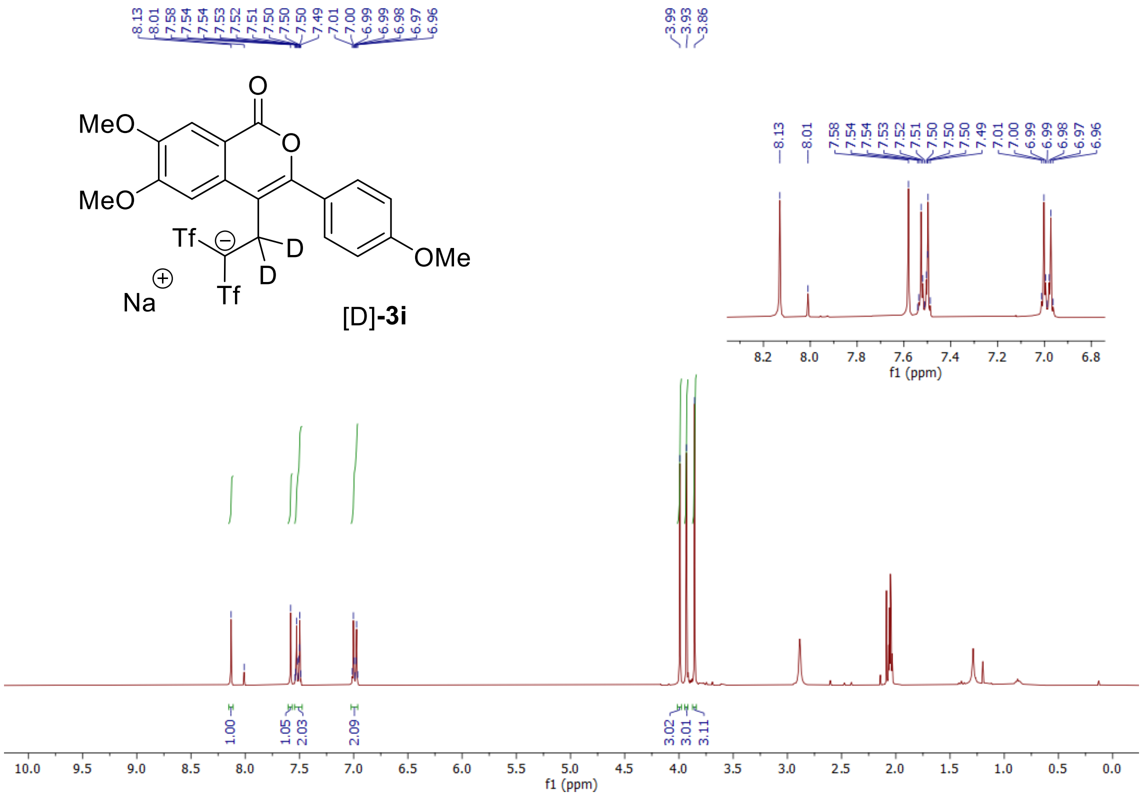

^{13}C NMR Compound [D]-**3i** (d_6-acetone, 75 MHz, 25 °C)

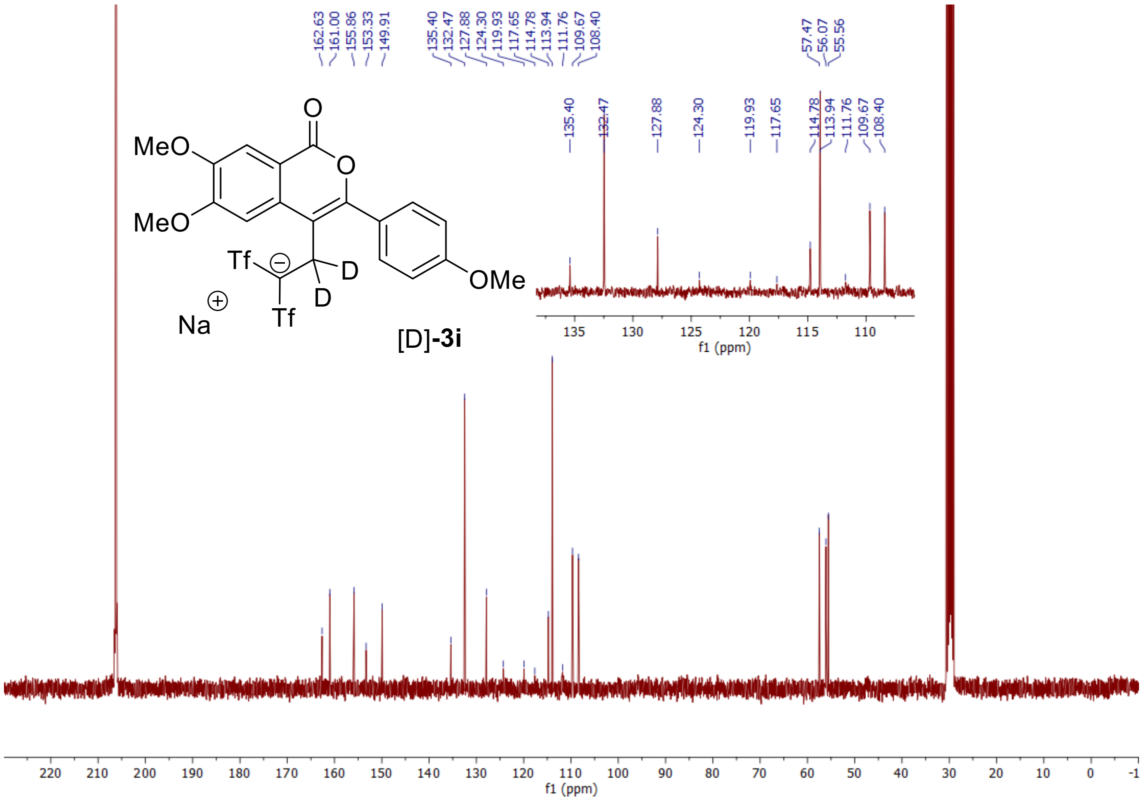

^{19}F NMR Compound [D]-**3i** (d_6-acetone, 282 MHz, 25 ^{o}C)

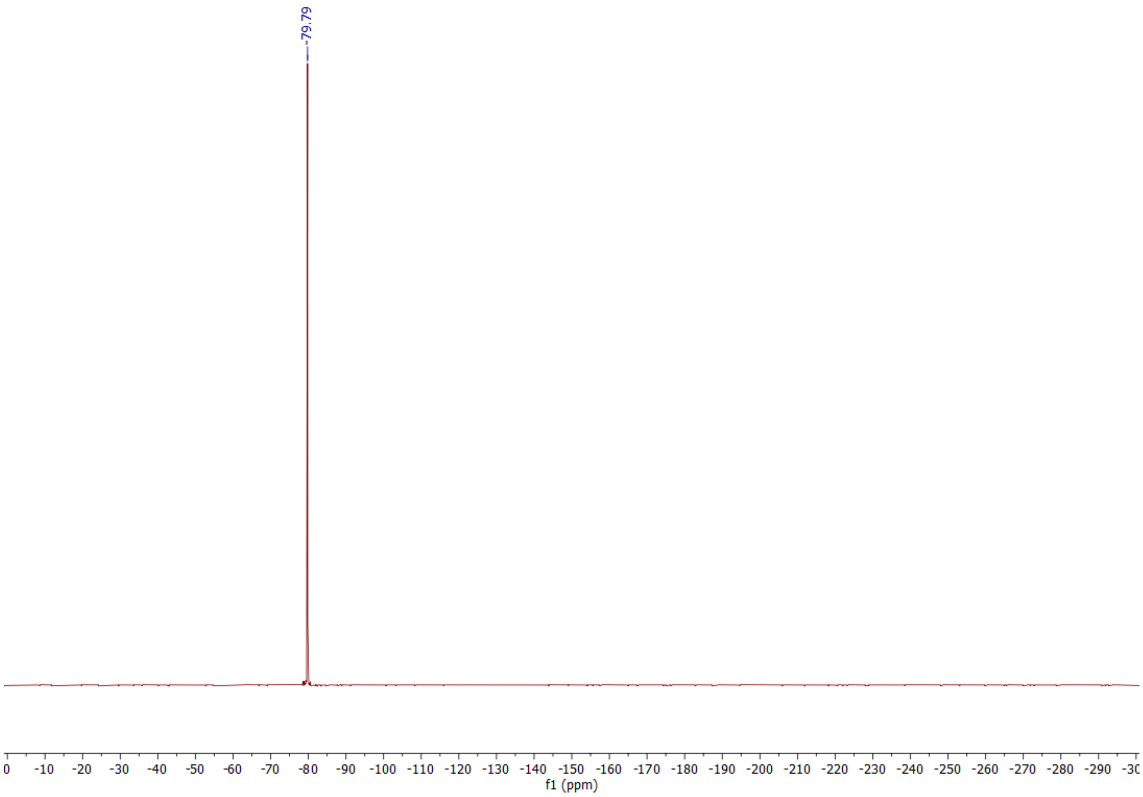

^{1}H NMR Compound **7a** ($CDCl_3$, 300 MHz, 25 °C)

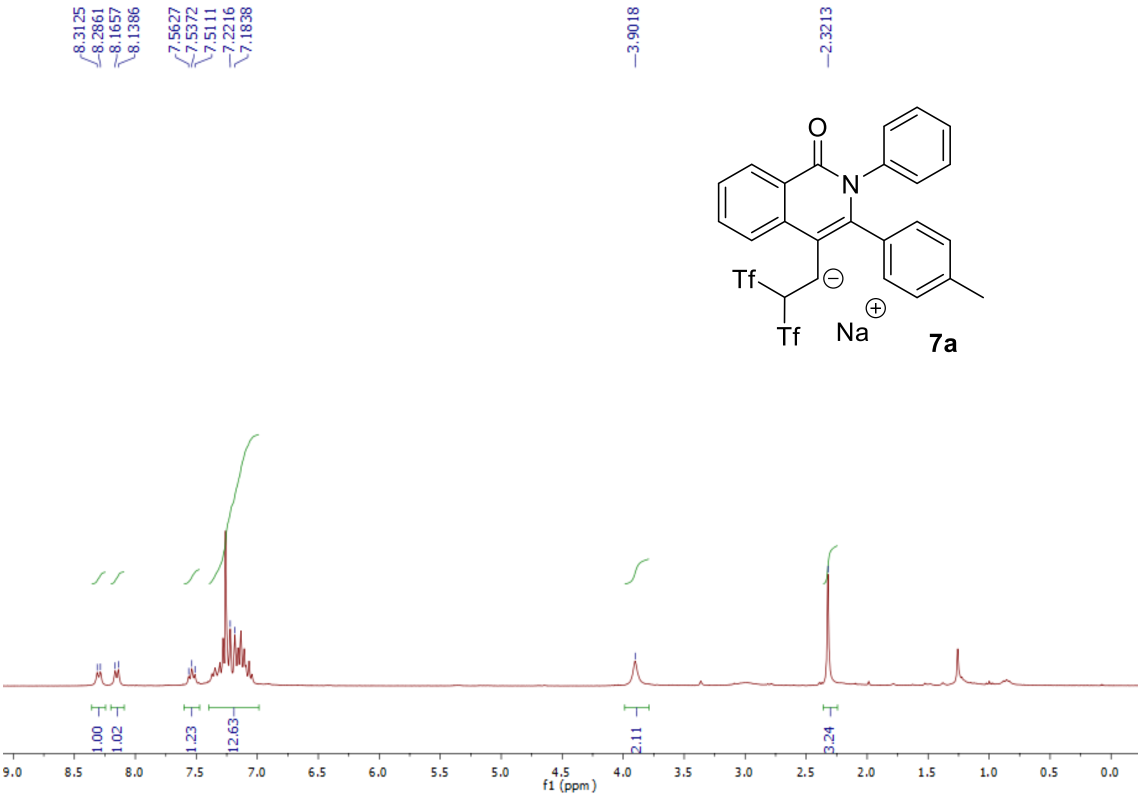

^{13}C NMR Compound **7a** (d_6-acetone, 75 MHz, 25 °C)

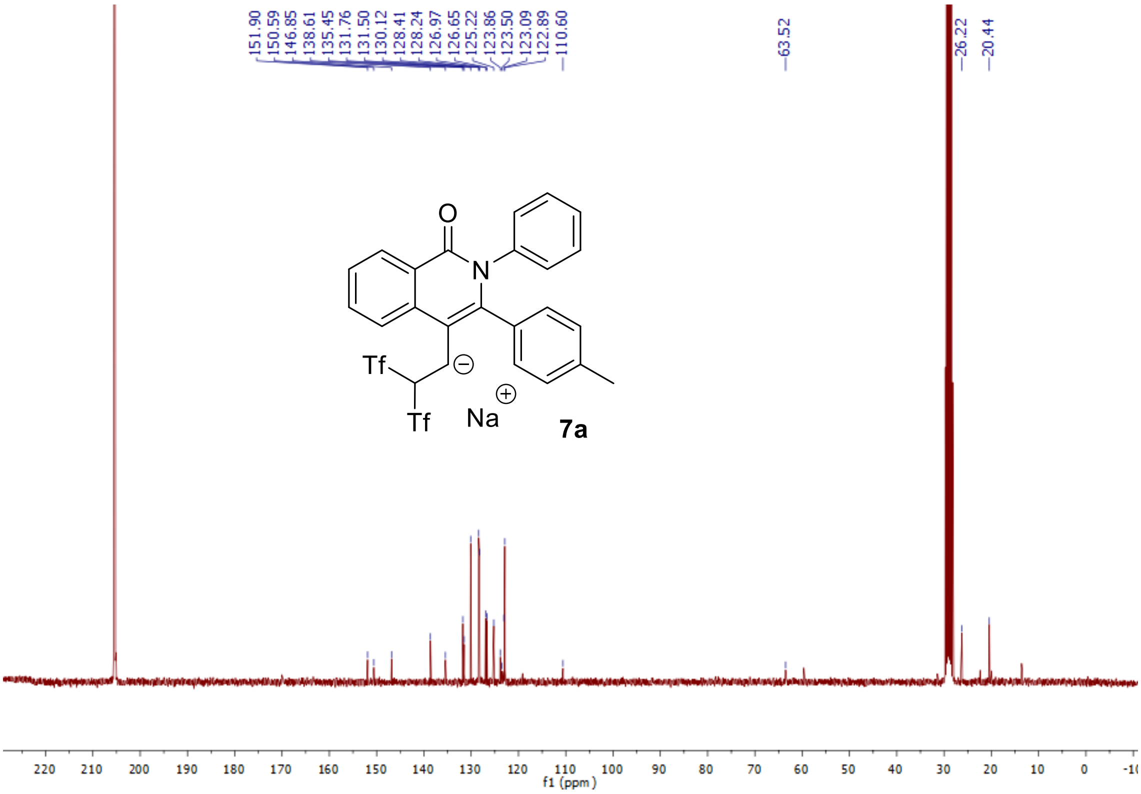

^{19}F NMR Compound **7a** (d$_6$-acetone, 282 MHz, 25 °C)

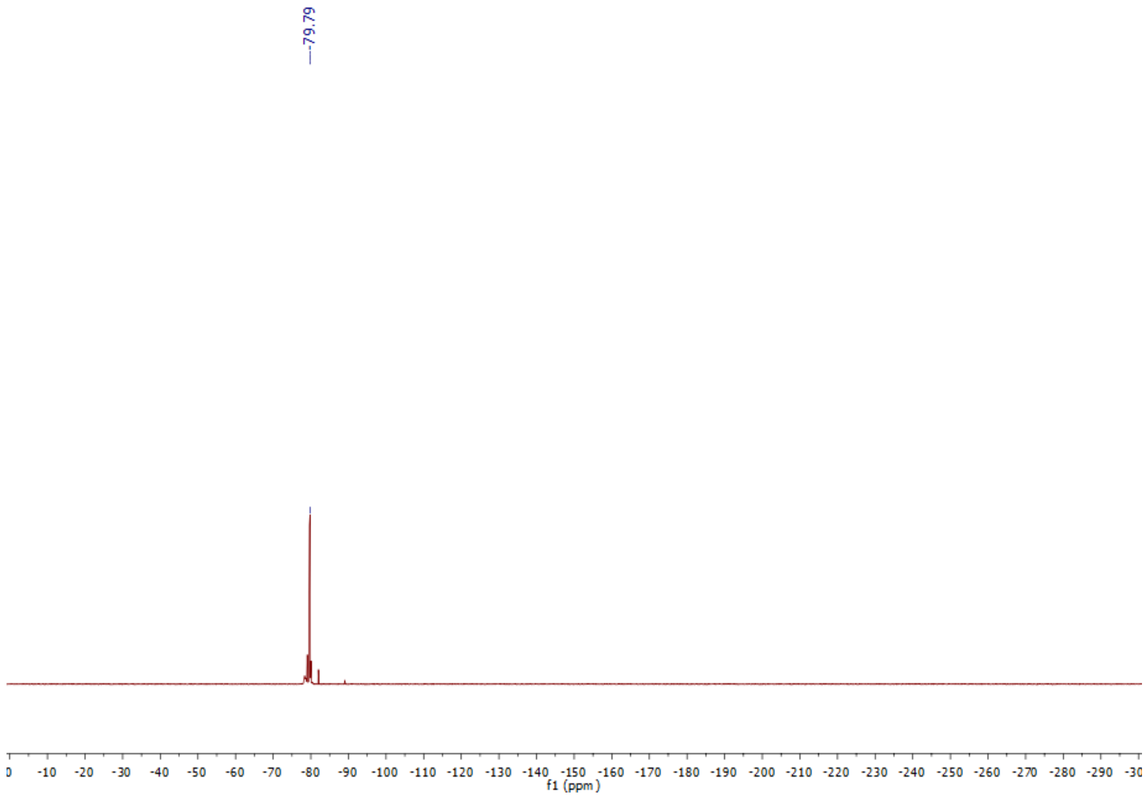

^{1}H NMR Compound **7b** (d_6-acetone, 300 MHz, 25 °C)

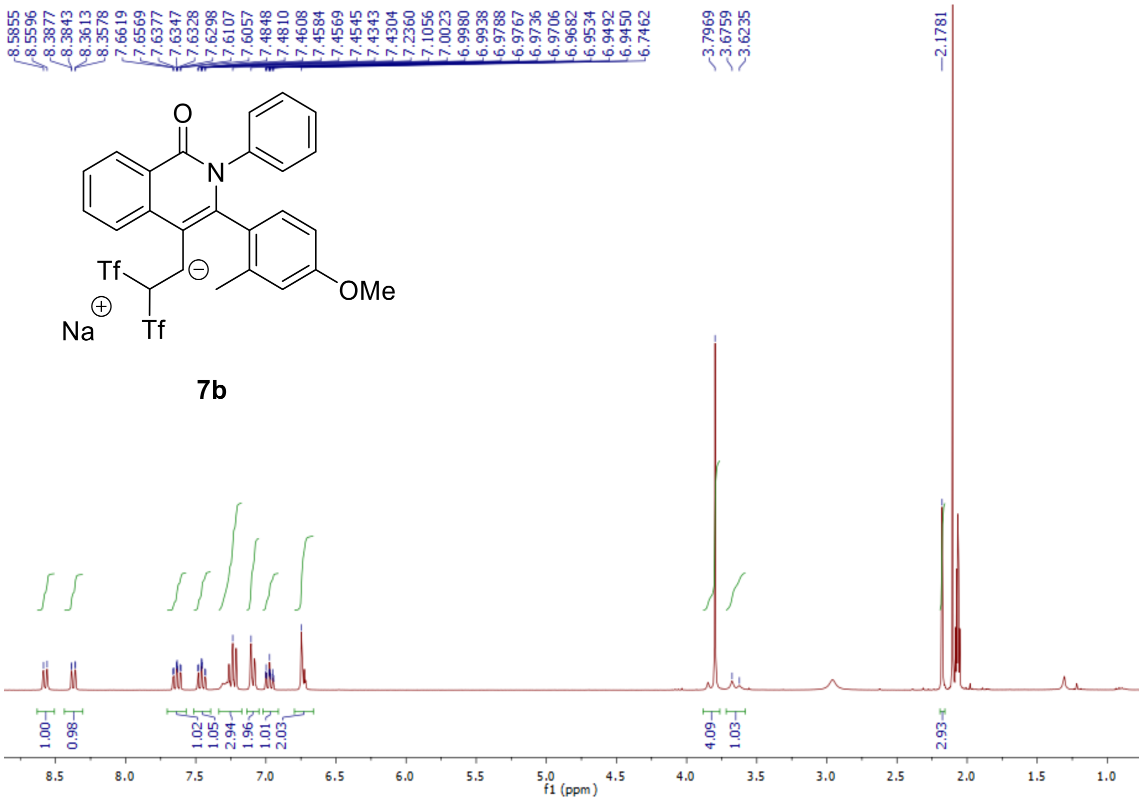

^{13}C NMR Compound **7b** (d_6-acetone, 75 MHz, 25 °C)

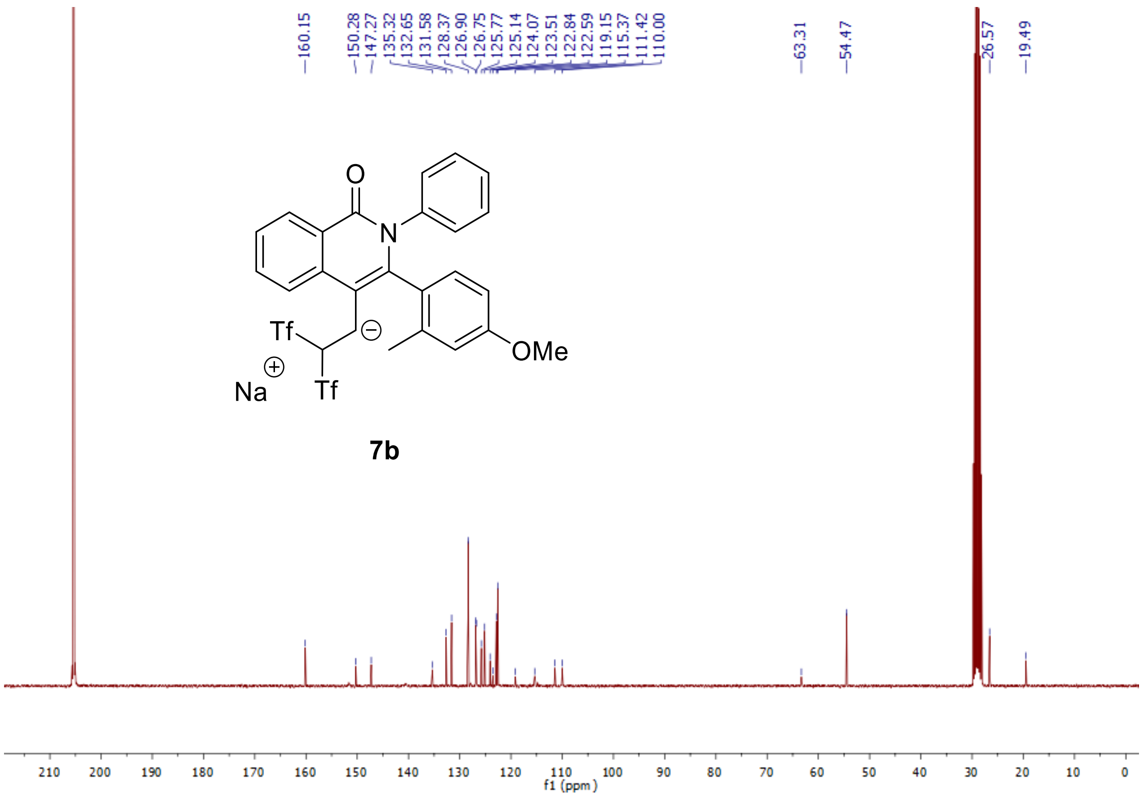

^{19}F NMR Compound **7b** (d_6-acetone, 282 MHz, 25 °C)

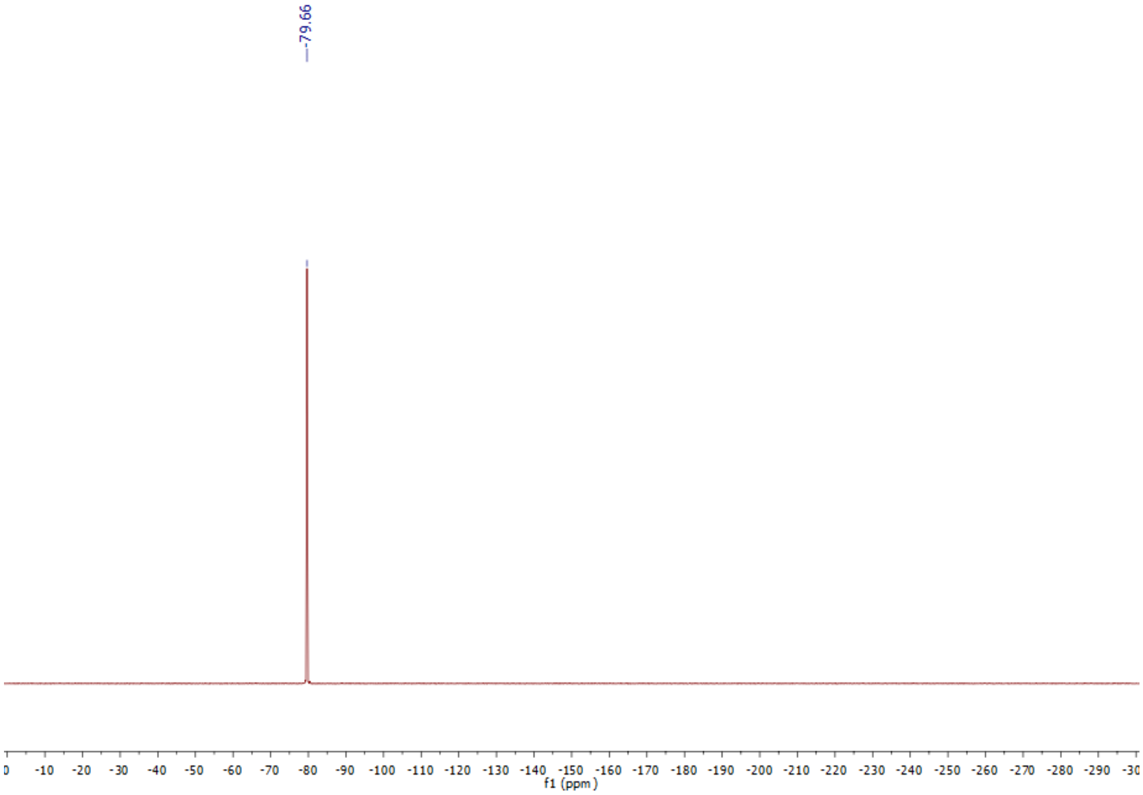

^{1}H NMR Compound **7c** (d_6-acetone, 300 MHz, 25 °C)

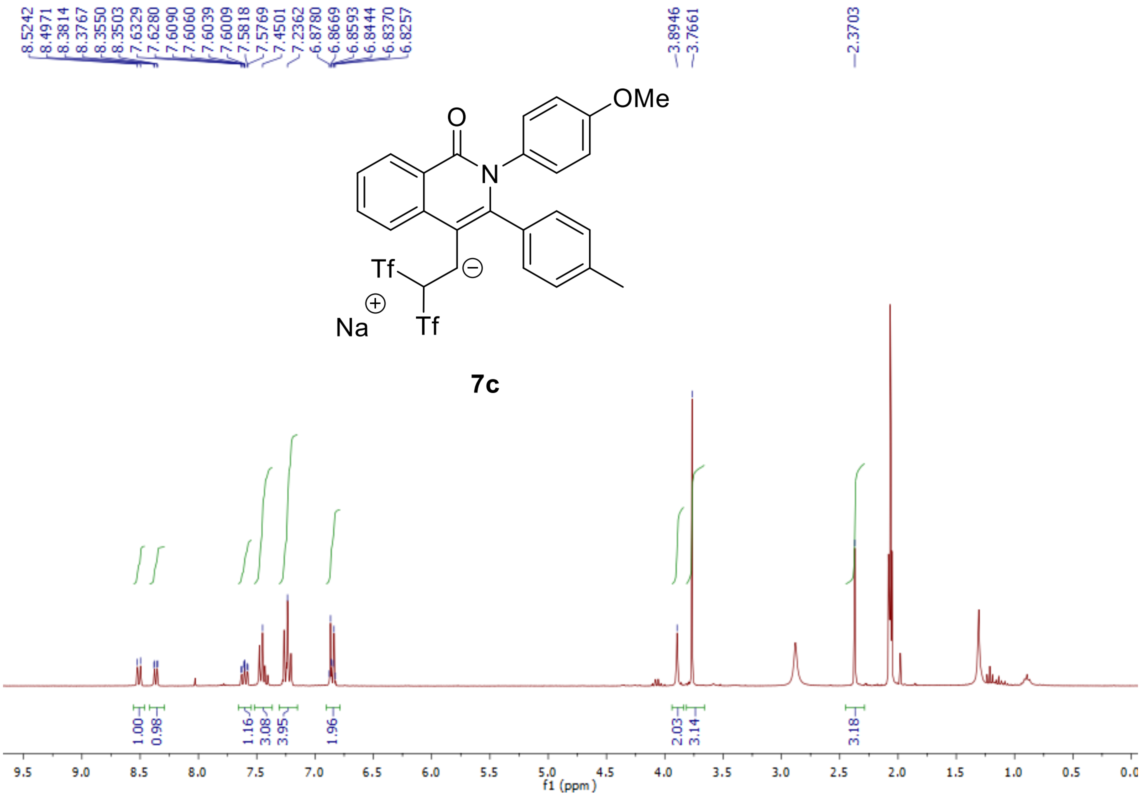

^{13}C NMR Compound **7c** (d_6-acetone, 75 MHz, 25 °C)

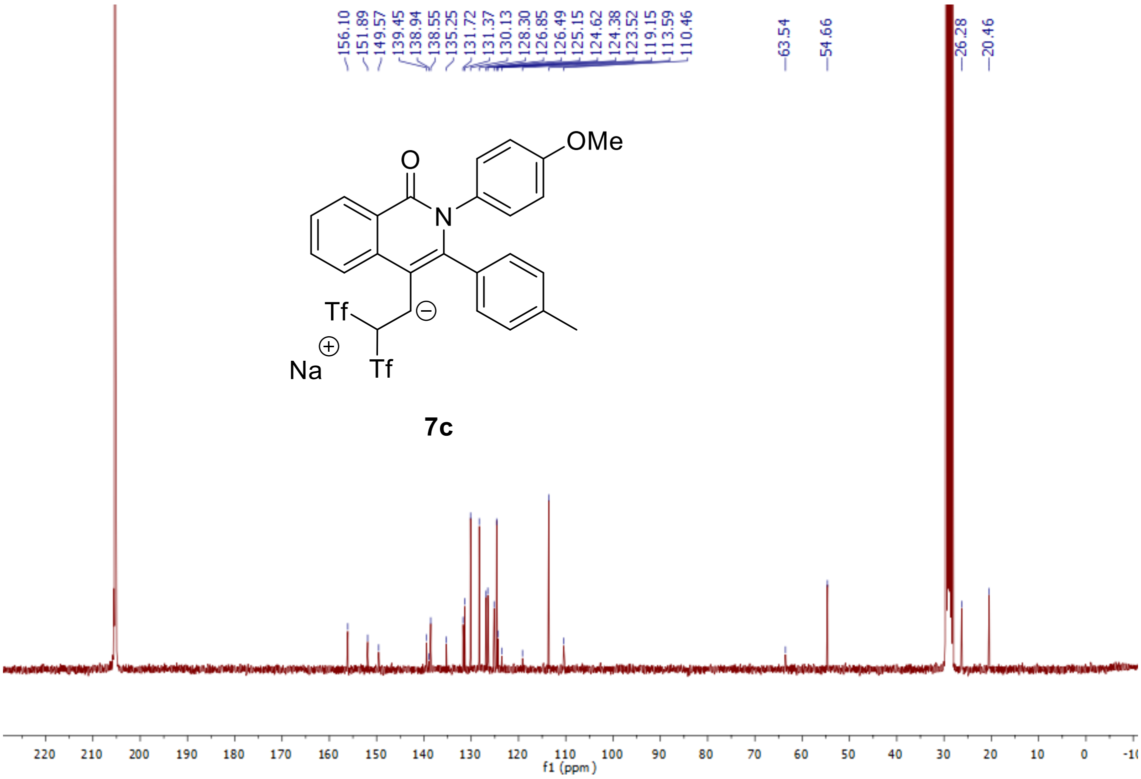

^{19}F NMR Compound **7c** (d_6-acetone, 282 MHz, 25 ^oC)

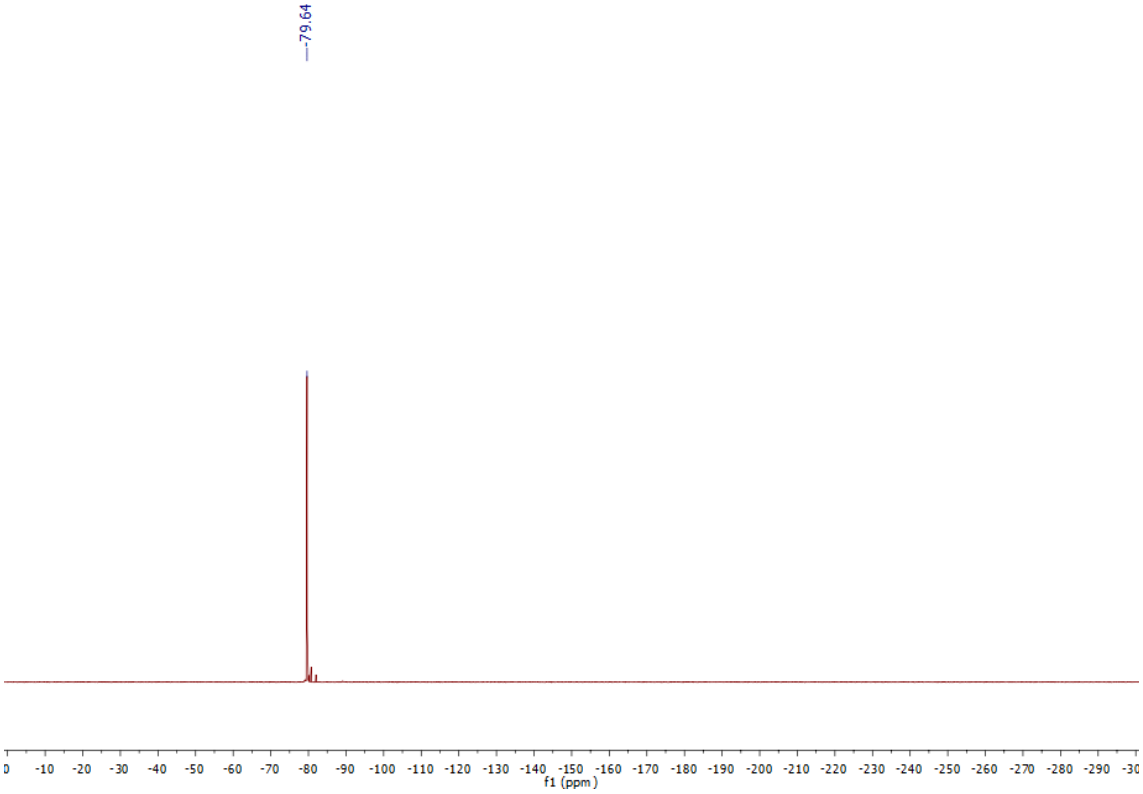

^{1}H NMR Compound **7d** (d_6-acetone, 300 MHz, 25 °C)

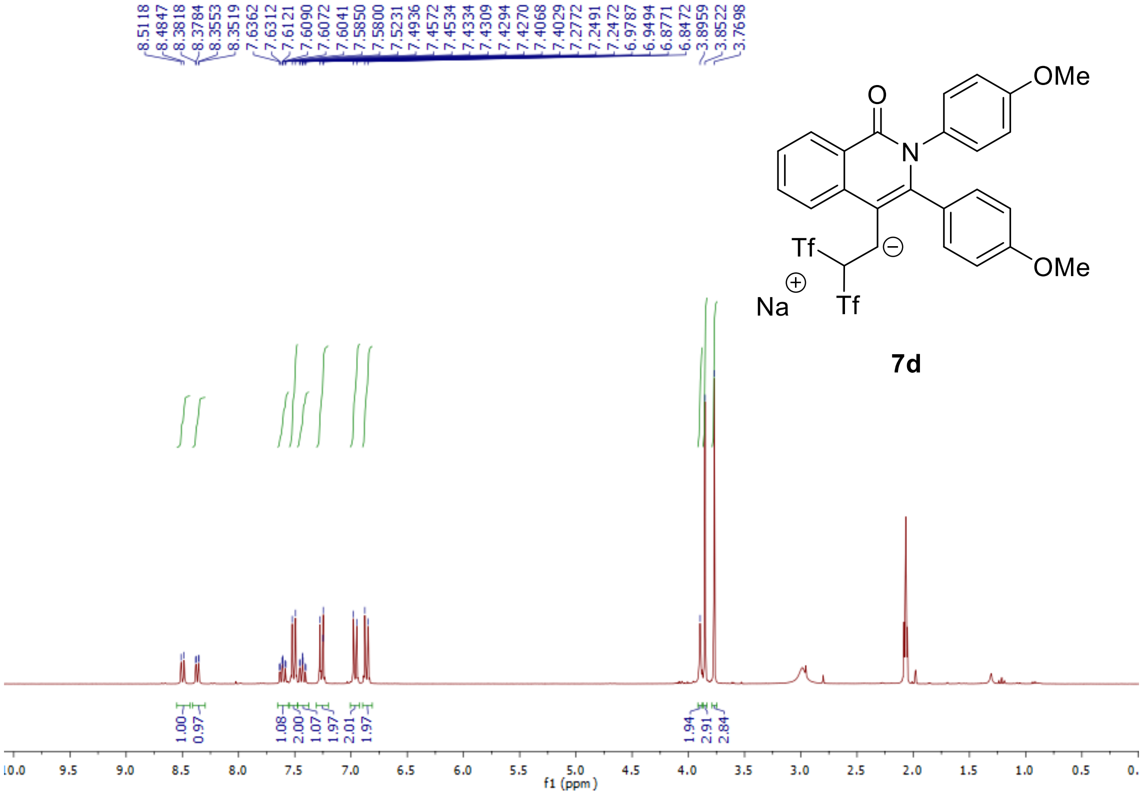

^{13}C NMR Compound **7d** (d_6-acetone, 75 MHz, 25 °C)

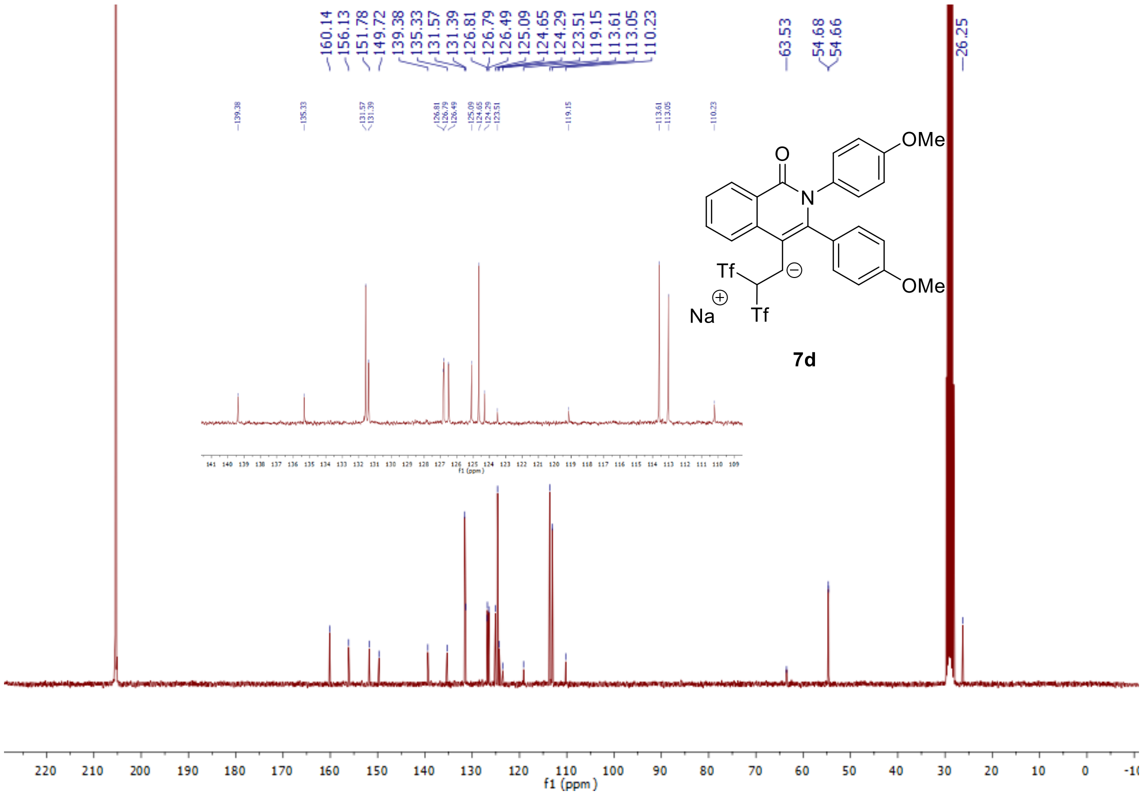

^{19}F NMR Compound **7d** (d_6-acetone, 282 MHz, 25 °C)

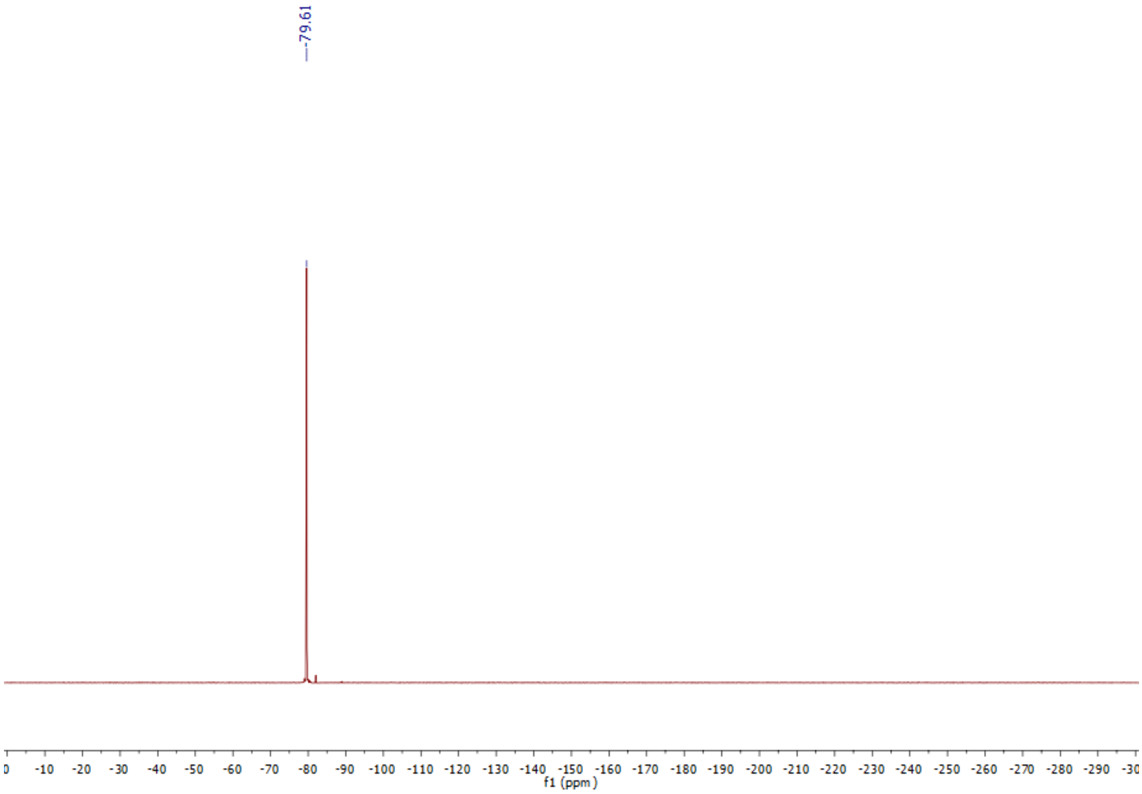

^{1}H NMR Compound **7e** (d$_6$-acetone, 300 MHz, 25 °C)

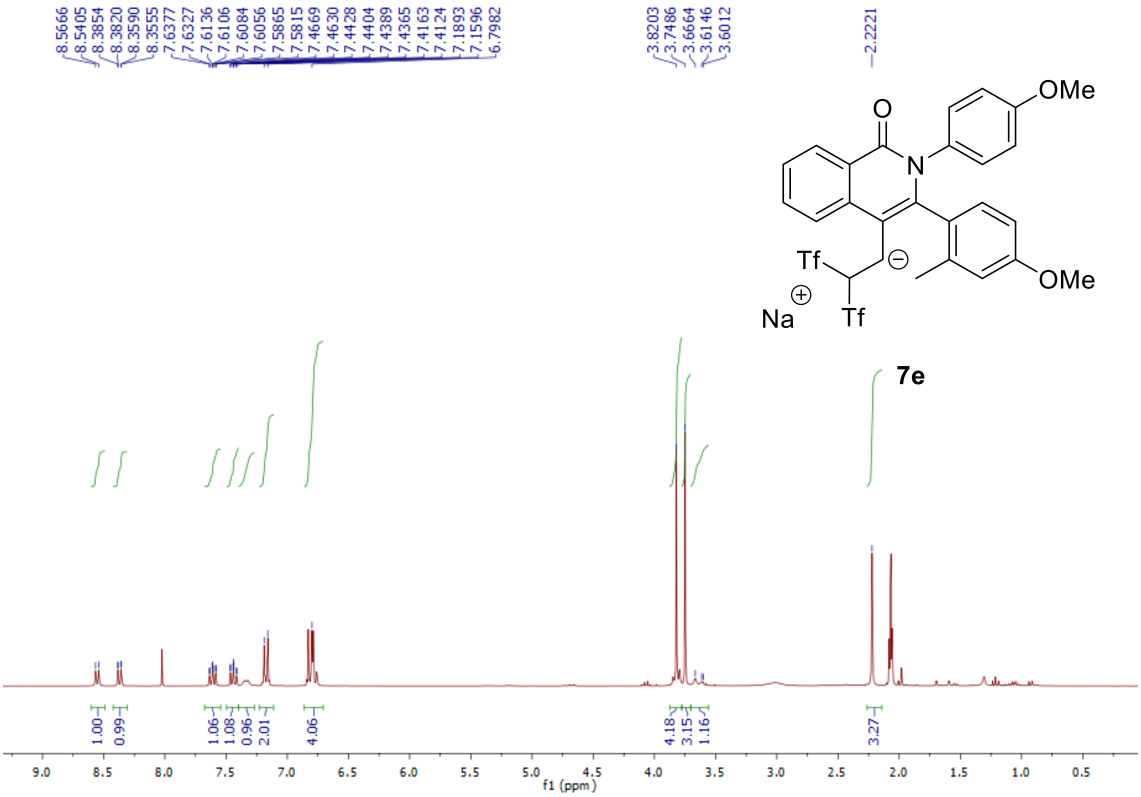

^{13}C NMR Compound **7e** (d$_6$-acetone, 75 MHz, 25 °C)

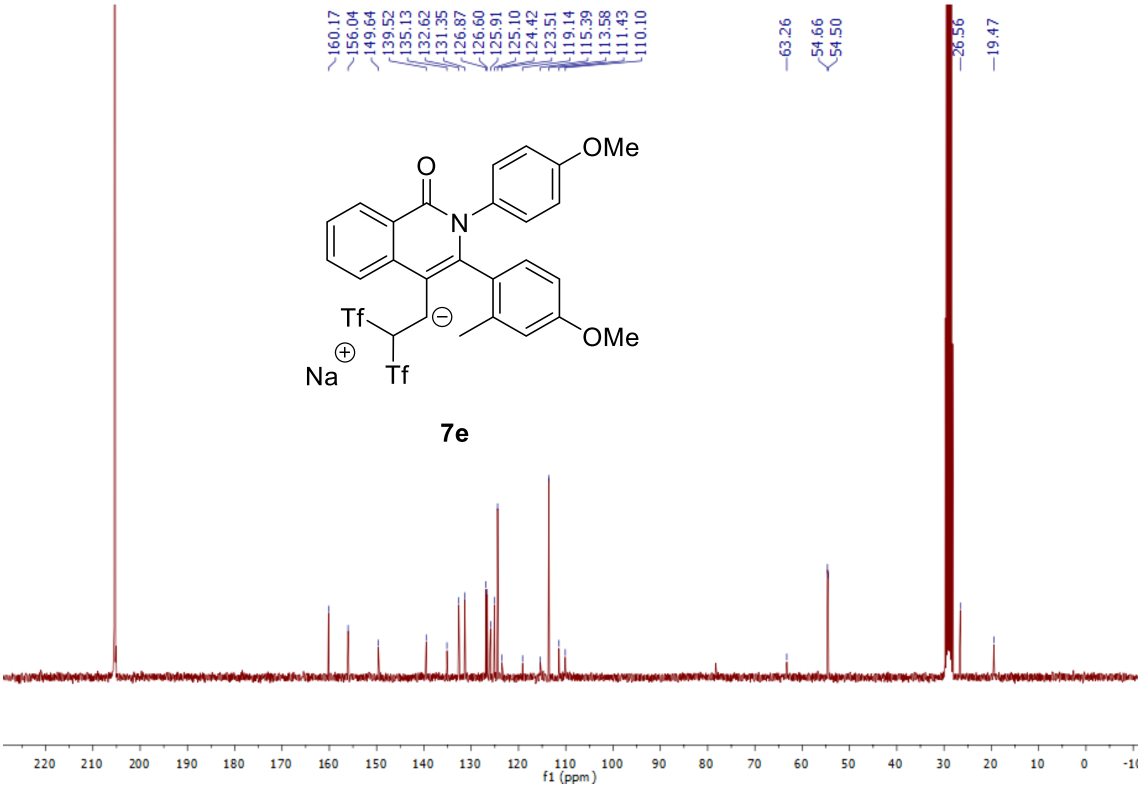

^{19}F NMR Compound **7e** (d_6-acetone, 282 MHz, 25 °C)

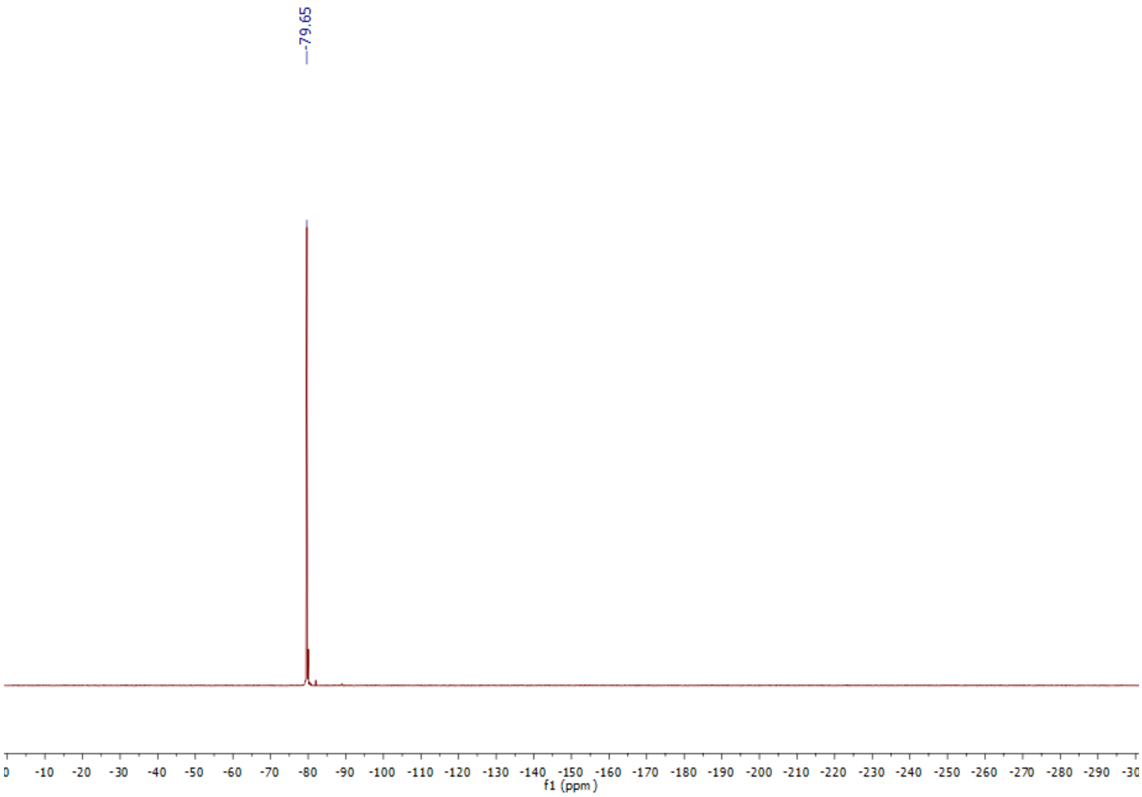

^{23}Na NMR Compound **7e** (d_6-acetone, 132 MHz, 25 °C)

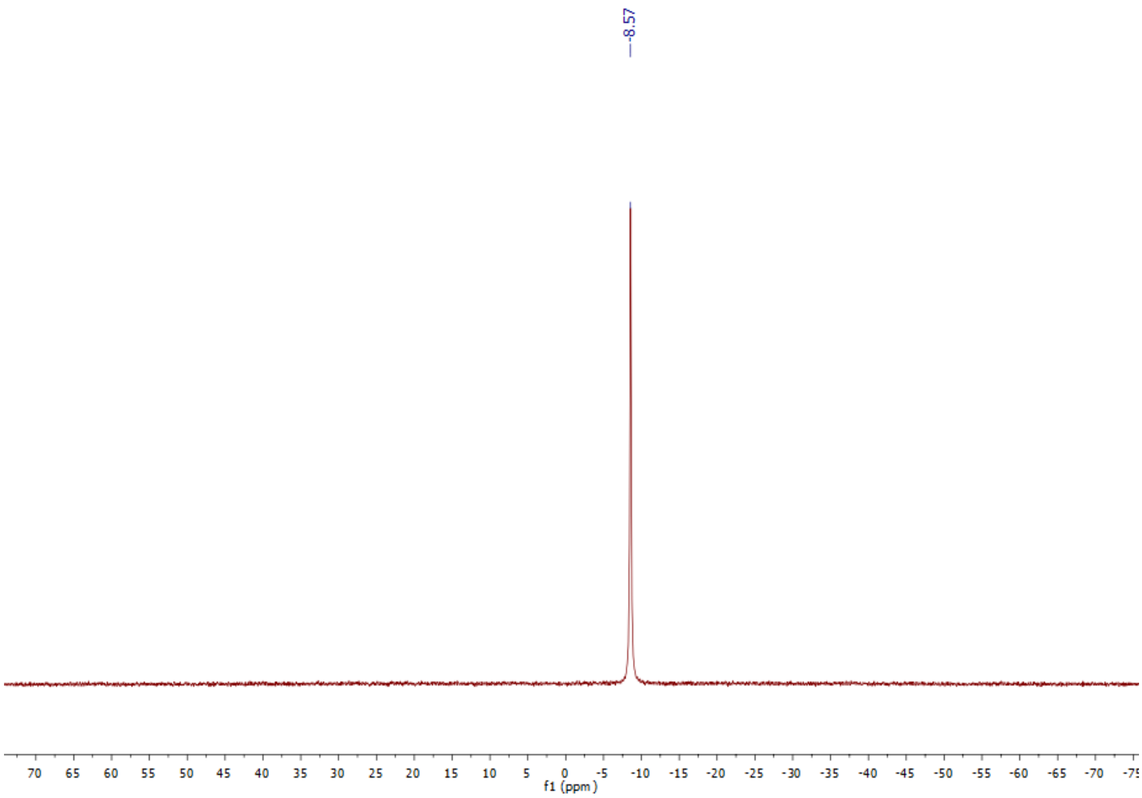

^{1}H NMR Compound **8a** ($CDCl_3$, 300 MHz, 25 °C)

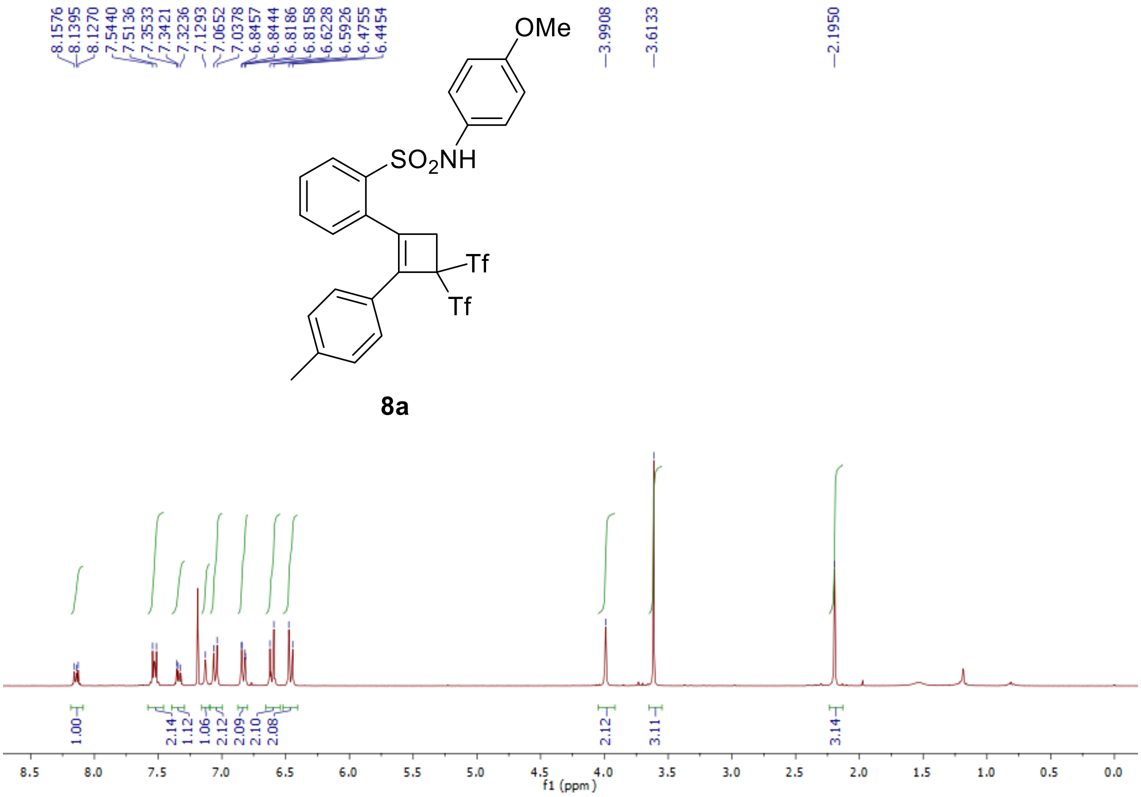

^{13}C NMR Compound **8a** ($CDCl_3$, 75 MHz, 25 °C)

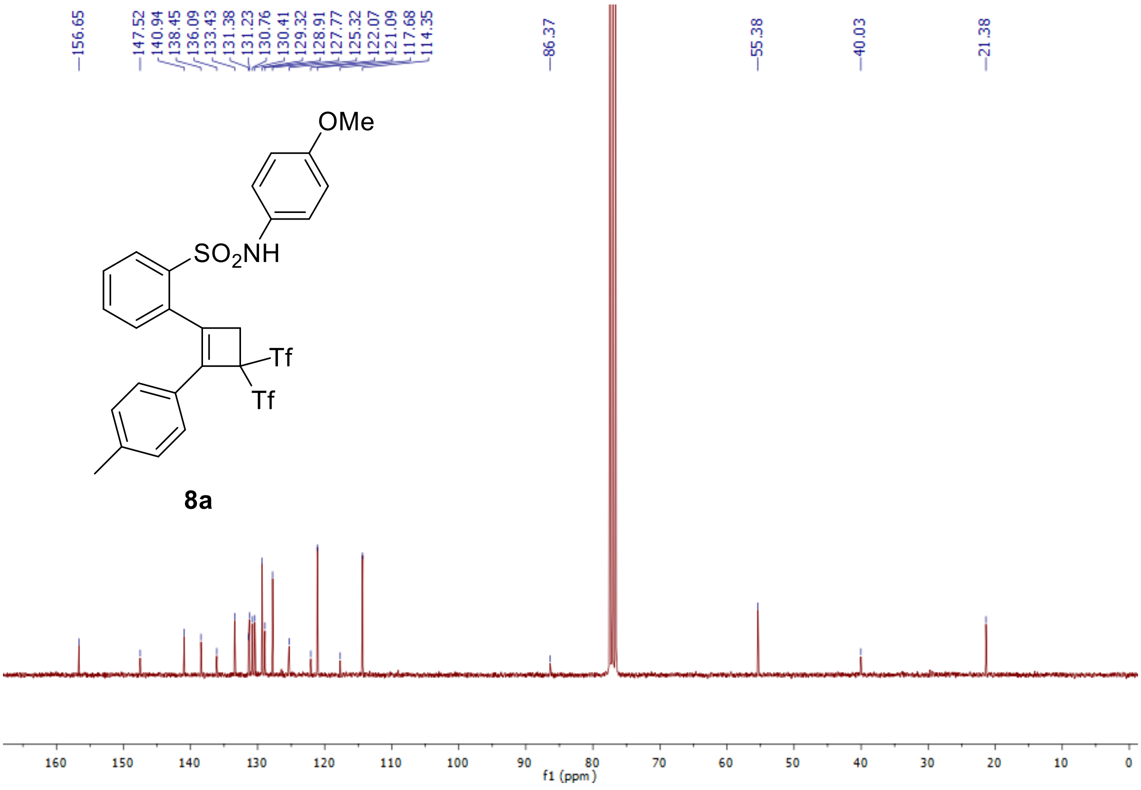

$^{1}H - ^{1}H$ COSY Compound **8a**

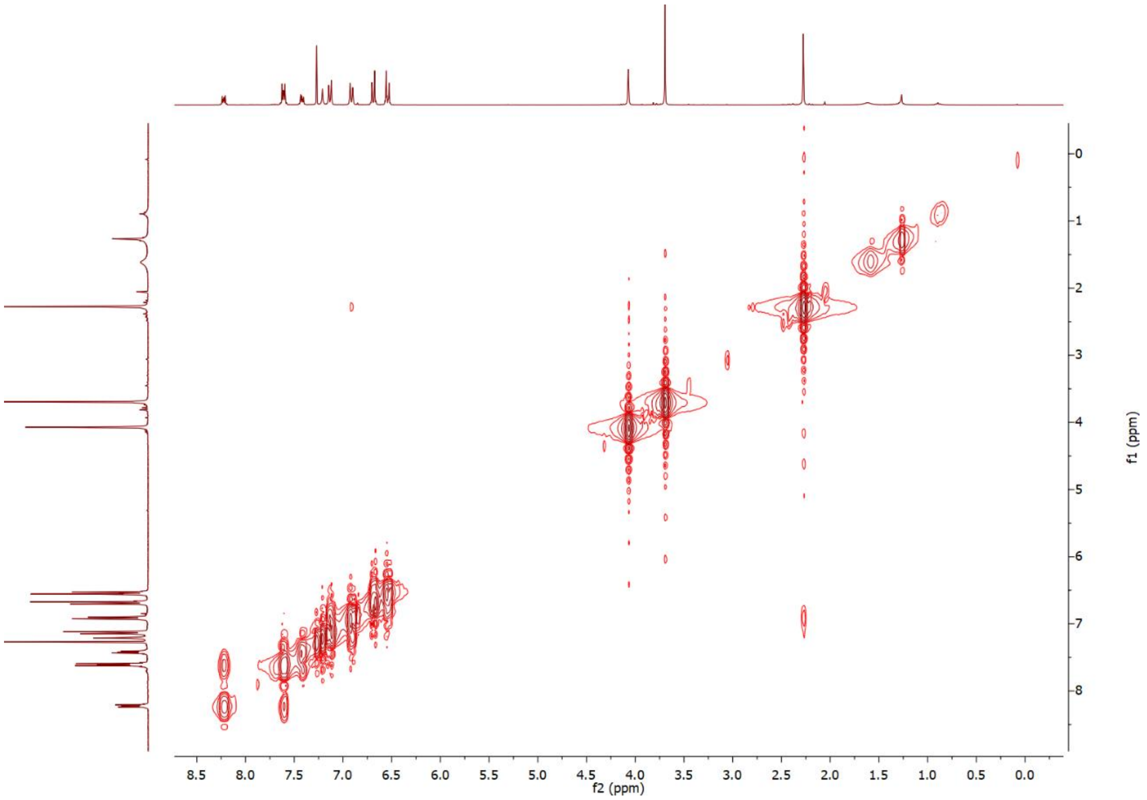

Table S13

#H	δ (ppm)	Multiplicity	Coupled with	J (Hz)
**H-6**	8.22	m	H-7 & H-8	COSY
**H-7 & H-8**	7.61	m	H-6	COSY
**H-9**	7.42	m	-	-
**NH**	7.21	s	-	-
**H-16**	7.13	d	H-17	8.2
**H-17**	6.91	d	H-16	8.2
			CH_3	COSY
**H-3**	6.69	d	H-2	9.1
**H-2**	6.54	d	H-3	9.1
**$C\mathit{H_2}CTf_2$**	4.07	s	-	-
**OCH_3**	3.69	s	-	-
**CH_3**	2.28	s	H-17	COSY

$^{1}H – ^{13}C$ HMQC Compound **8a**

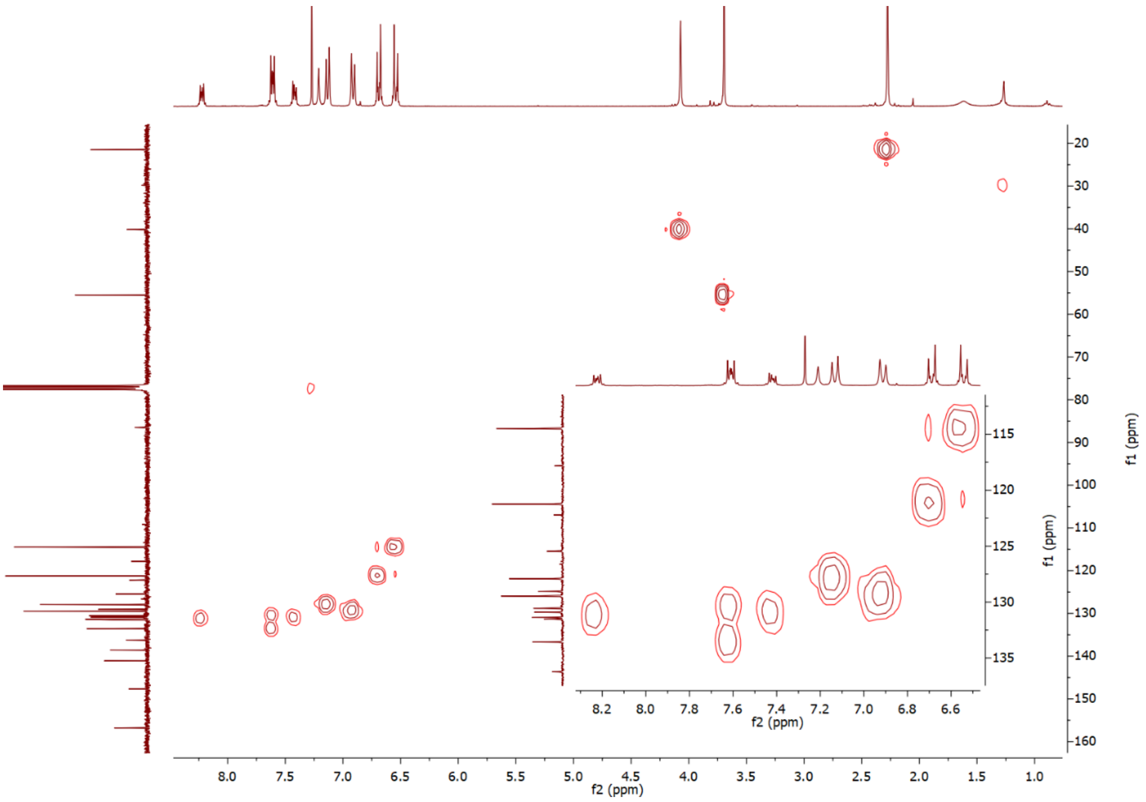

$^{1}H – ^{13}C$ HMBC Compound **8a**

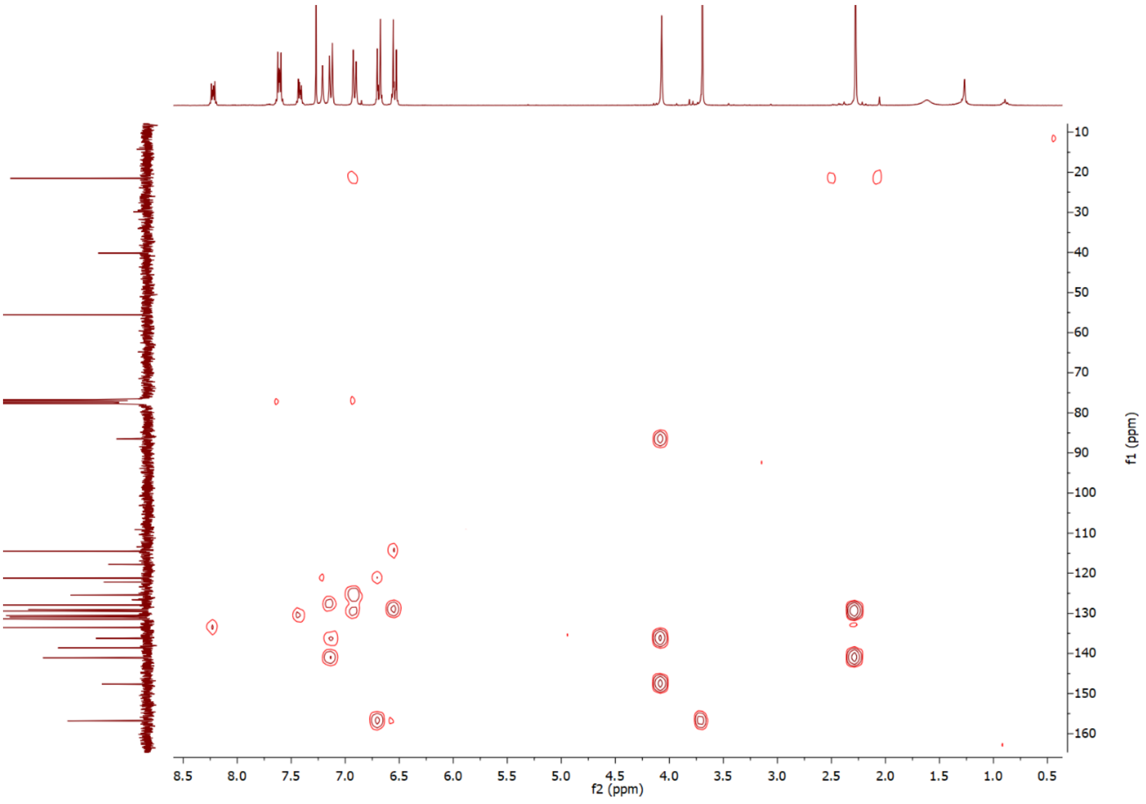

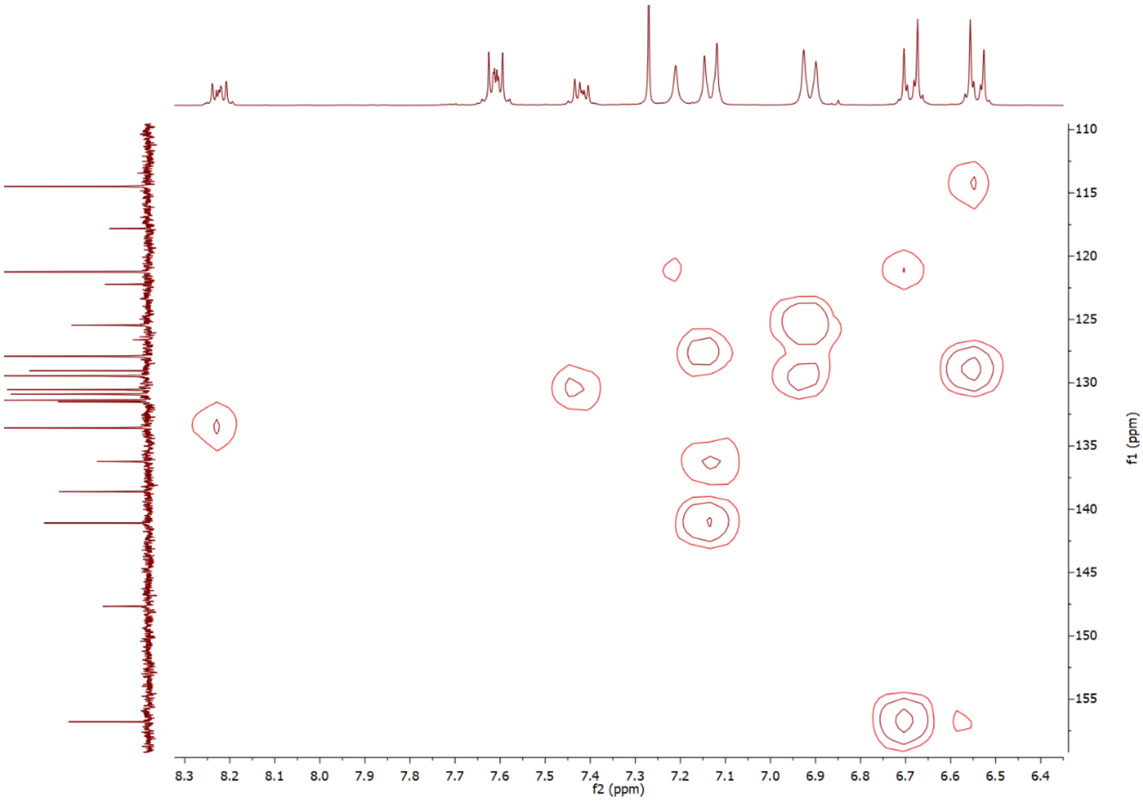

Table S14

#C	δ (ppm)		HMBC coupling	
**C-1**	156.8	$C_{Ar\text{-}OMe}$	H-2 (^{2}J), OCH_3 (^{3}J), H-3 (^{3}J)	Fig. 1
**C-11**	147.7	*C*=C	H-12 (^{2}J)	Fig. 1
**C-18**	141.1	$C_{Ar\text{-}Me}$	CH_3 (^{2}J), H-16 (^{3}J)	Fig. 1
**C-5**	138.6	$C_{Ar\text{-}SO2NH}$	-	-
**C-14**	136.2	C=*C*	H-12 (^{3}J), H-16 (^{3}J)	Fig. 2
**C-8**	133.6	CH_{Ar}	H-6 (^{3}J)	Fig. 1
**C-10**	131.5	C_{Ar}	-	-
**C-6**	131.4	CH_{Ar}	-	-
**C-9**	130.9	CH_{Ar}	-	-
**C-7**	130.6	CH_{Ar}	H-9 (^{3}J)	Fig. 2
**C-17**	129.5	CH_{Ar}	CH_3 (^{3}J)	Fig. 2
**C-4**	129.1	$C_{Ar\text{-}NHSO2}$	H-2 (^{3}J)	Fig. 2
**C-16**	127.9	CH_{Ar}	-	-
**C-15**	125.5	C_{Ar}	H-17 (^{3}J)	Fig. 1
**C-3**	121.2	CH_{Ar}	NH (^{3}J)	Fig. 1
CTf₂	120.0	CF_3	-	-
**C-2**	114.5	CH_{Ar}	-	-
**C-13**	86.5	*C*Tf_2	H-12 (^{2}J)	Fig. 1
**OCH_3**	55.5	CH	-	-
**C-12**	40.2	CH_2	-	-
**CH_3**	21.5	CH	H-17 (^{3}J)	Fig. 2

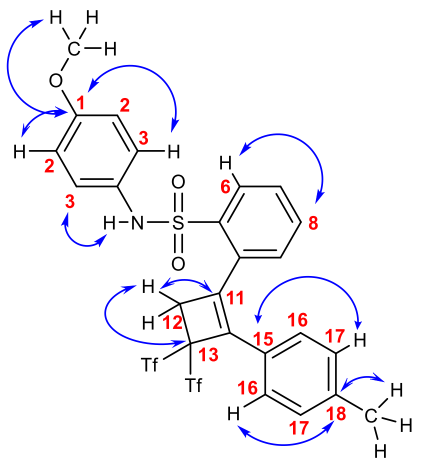

Chart S10

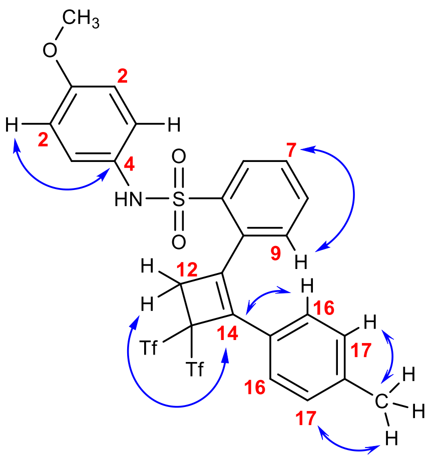

Chart S11

^{19}F NMR Compound **8a** ($CDCl_3$, 282 MHz, 25 °C)

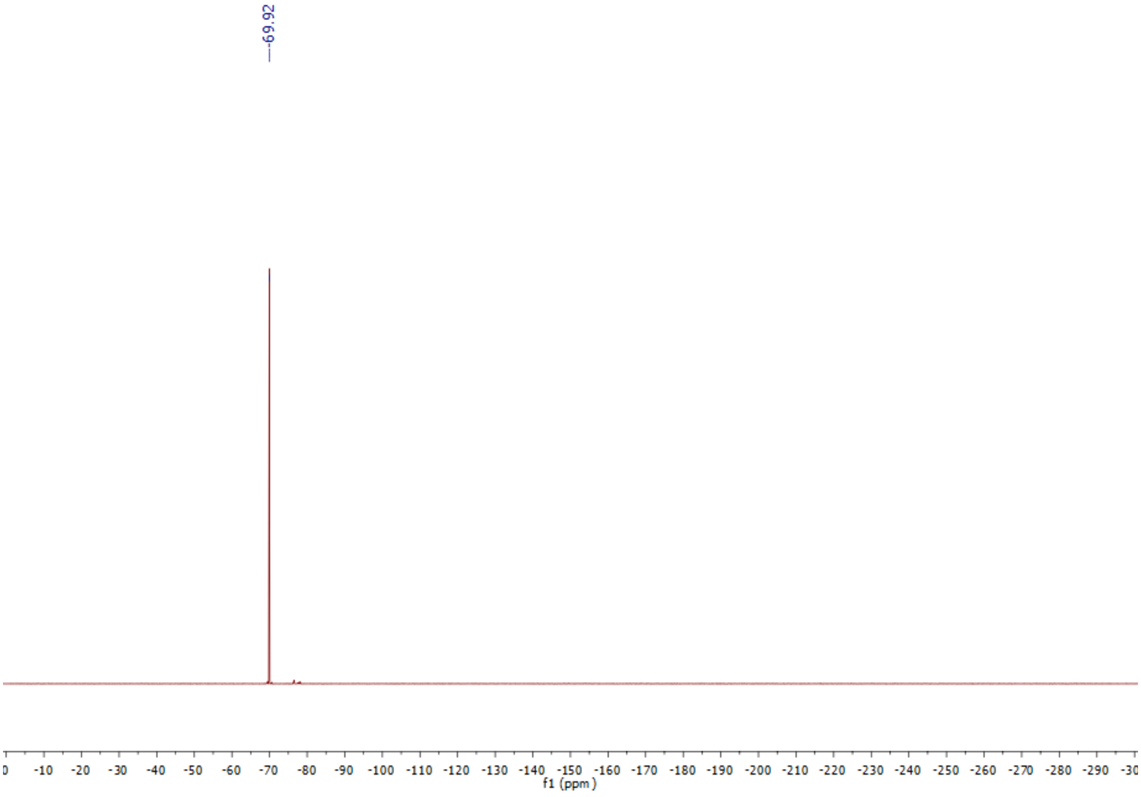

^{1}H NMR Compound **8b** ($CDCl_3$, 300 MHz, 25 °C)

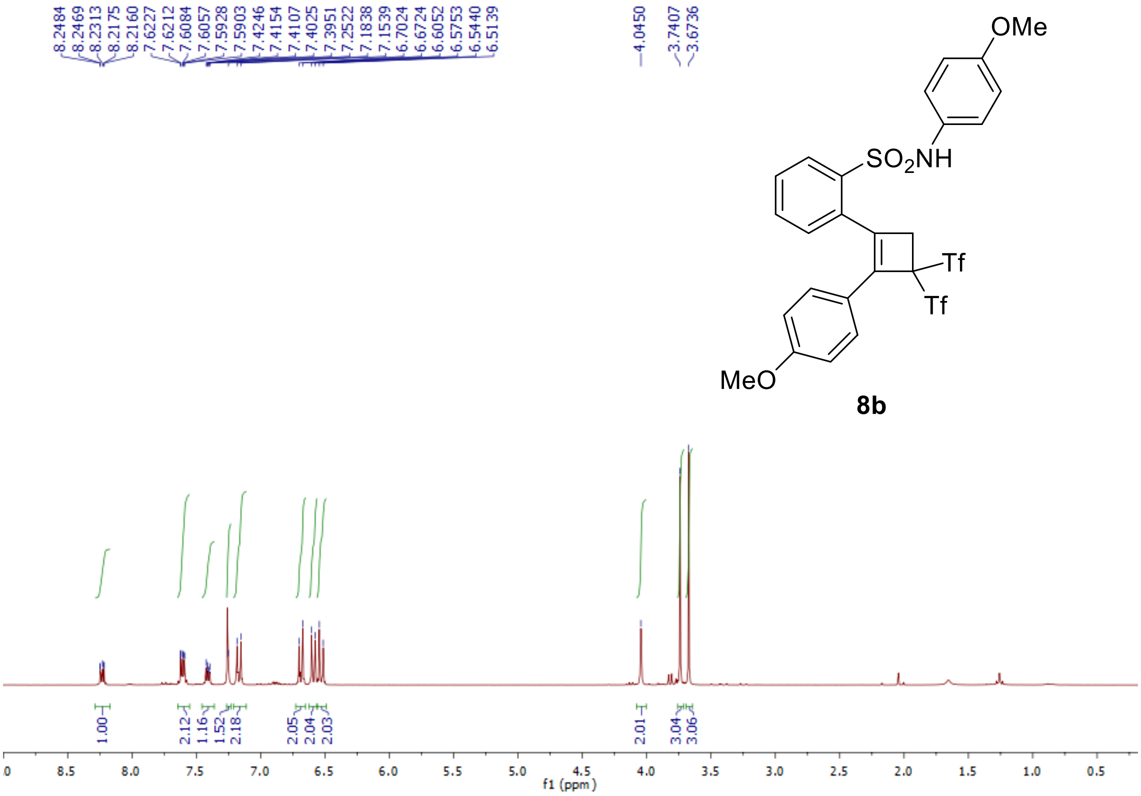

^{13}C NMR Compound **8b** ($CDCl_3$, 75 MHz, 25 °C)

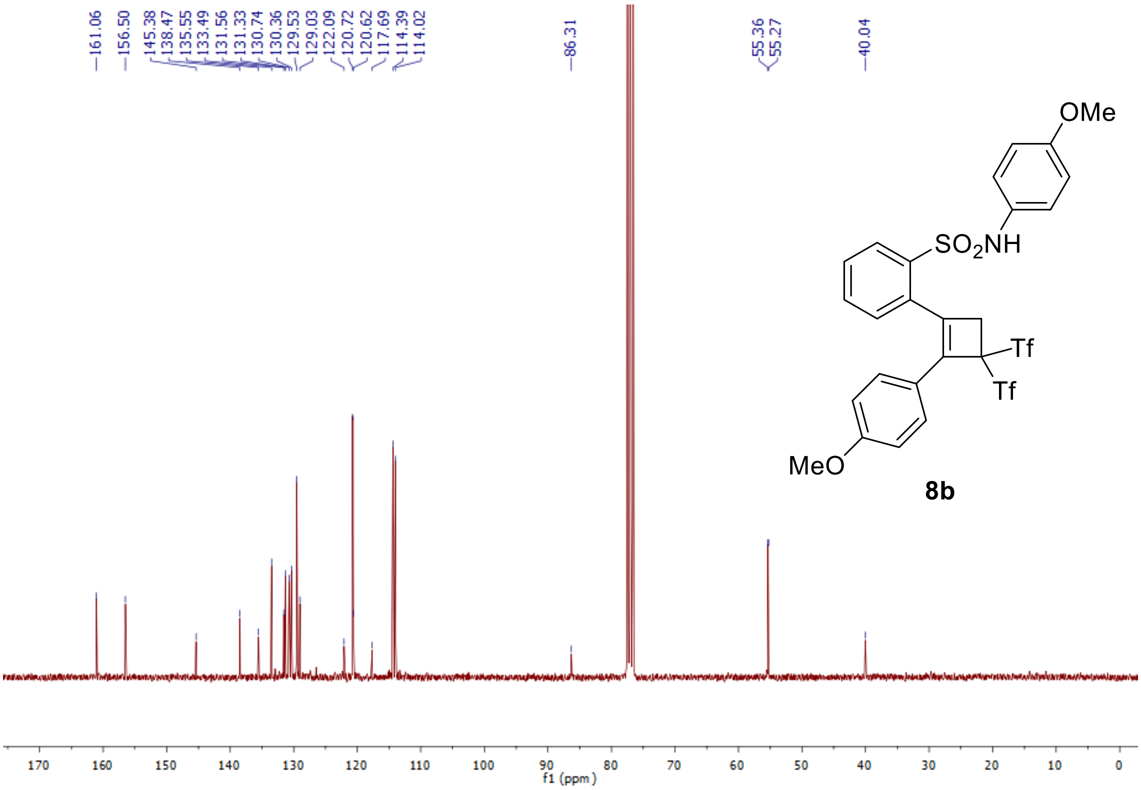

^{19}F NMR Compound **8b** ($CDCl_3$, 282 MHz, 25 °C)

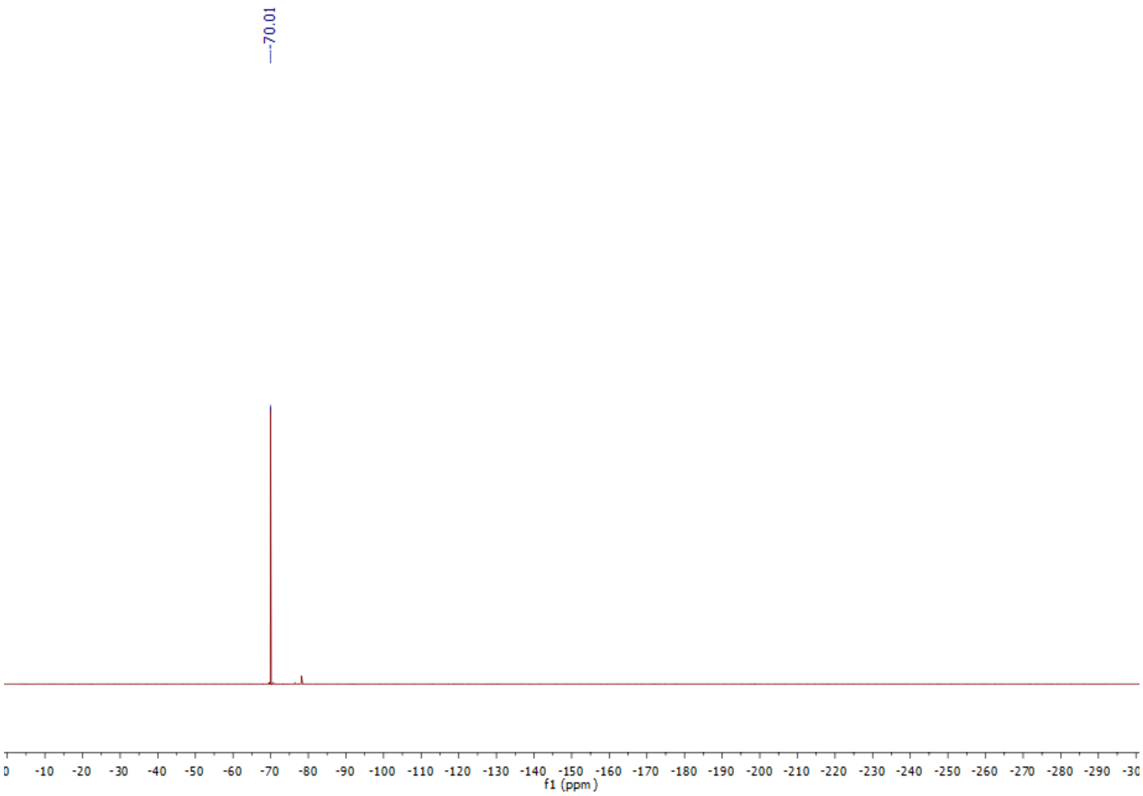

Molecular docking study of compounds 3a and 3l

The spatial conformation of compounds **3a** and **3l** was explored by molecular docking in order to reveal the differences in their binding modes to the hAChE. The structure of hAChE crystallized with fasciculin II (PDB ID: 1B41) was used, the docking was executed using AutoDock Vina [11] software and the results were analyzed with Discovery Studio. As we previously described, the flexibility in the enzyme has been considered by allowing the side chain movement of eight aminoacids during the docking search as performed by software AutoDock Vina [1].

Figure S1 shows the docking orientation of the compound **3a** in the active site of hAChE. Two binding modes (Mode I and Mode II) can be proposed for this compound resulting in its higher affinity with the catalytic active site (CAS) (binding energy: -9.8 kcal/mol) than with the peripheral anionic site (PAS) (binding energy: -8.2 kcal/mol).

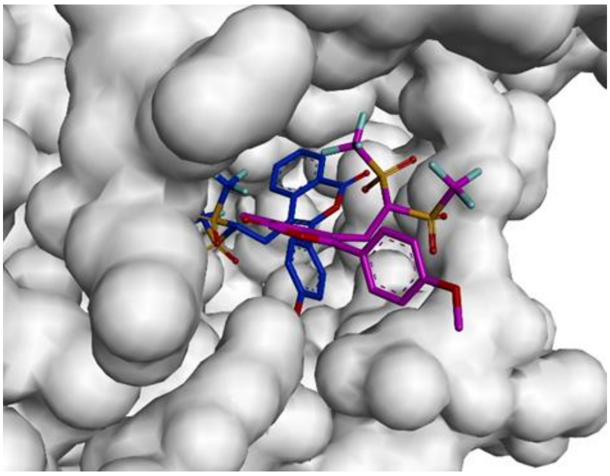

Figure S1. Surface representation of hAchE, **3a, Mode I** (blue) and **3a, Mode II** (pink) complexes.

At the CAS (Mode I), compound **3a** forms stable network of interactions through triflyl groups (Figure S2). The F atoms of the CF_3 groups interacted with key aminoacids to form strong halogen interactions. F atoms held Gly448, His447 (aminoacid of the catalytic triad), Gly121 and Gly122 (oxyanion hole) resulting in the presence of O…F, N…F, C-H…F and N-H…F interactions. The sulfonyl group was found to form a hydrogen bond with Tyr337 and π-sulfur interactions with His447 and Trp86. Near the bottom of the gorge, the benzene ring of the chromenone moiety established π-sigma interactions with Trp86, a residue known for attracting the quaternary amine of the acetylcholine. The methoxy-phenyl and lactone moieties lay in the middle of the gorge

between the CAS and PAS interacting with the amino acids Tyr341 and Tyr124 through π-π T-shaped interactions. Additionally, the phenyl ring forms π–Anion interactions with Asp74 (PAS) (Figure S2).

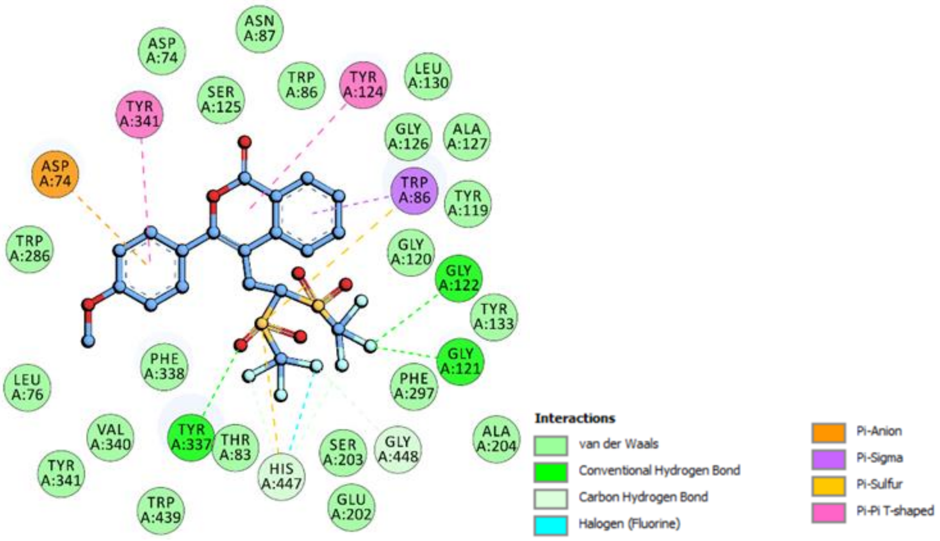

Figure S2. Amino acids in the binding site of hAChE interacting with ligand **3a**, Mode I**.** 2D representation of the amino acids in the binding site interacting with the ligand.

In Mode II, compound **3a** is located in the pocket forming PAS where the Trp286 is interacting with the methoxy-phenyl ring via π-π stacking interaction, and with lactone moiety forming two hydrogen bonds (Figure S3). In this situation, the halogen atoms are interacting with Val73, Asp74 and Leu76, which also highly contributed to the stabilization of the complex (Figure S3). On the other hand, sulfonyl groups form a hydrogen bond with Thr75 and π-sulfur interaction with Tyr72.

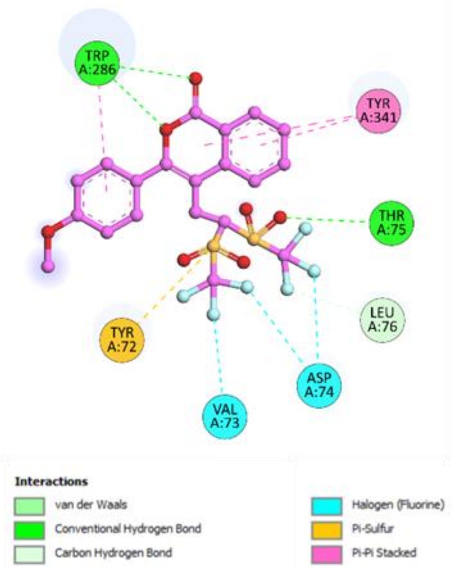

Figure S3. Predicted binding mode II of compound **3a** in hAChE. 2D representation of the amino acids in the binding site interacting with the ligand.

To understand the role of the R groups on the ligands, docking is done with the substituted ligand **3l** and the binding energy and interacting amino acid are compared with that of the unsubstituted ligand **3a.**

We observed that the compound **3l** is arranged at the active-site gorge of hAchE in a position similar to that found for compound **3a** in Mode II. As shown in Figure S4 the most energetically favoured binding mode places the ligand (**3l**) in the PAS with chromenone moiety stacking with Tyr341 residue and no binding of compound was observed at catalytic triad.

In the complex, the sulfonyl group interacts with Tyr341 and Tyr72 via π-sulfur interactions and with Thr75 via hydrogen bond interaction. In this situation, the fluorine atoms are also located in the PAS interacting with Asp74, Leu76, Thr75 and Tyr124. The methoxy groups of the chromenone are pointed toward the catalytic triad residues but it cannot reach the bottom of the gorge interacting with the amino acids in the middle (Figure S4). Finally, the disubstituted-benzene ring is positioned out of the gorge entrance.

Based upon docking experiments, it can be proposed that the less substituted compound (**3a**) provides a better chance for the triflyl group to access the active site on the bottom of the gorge.

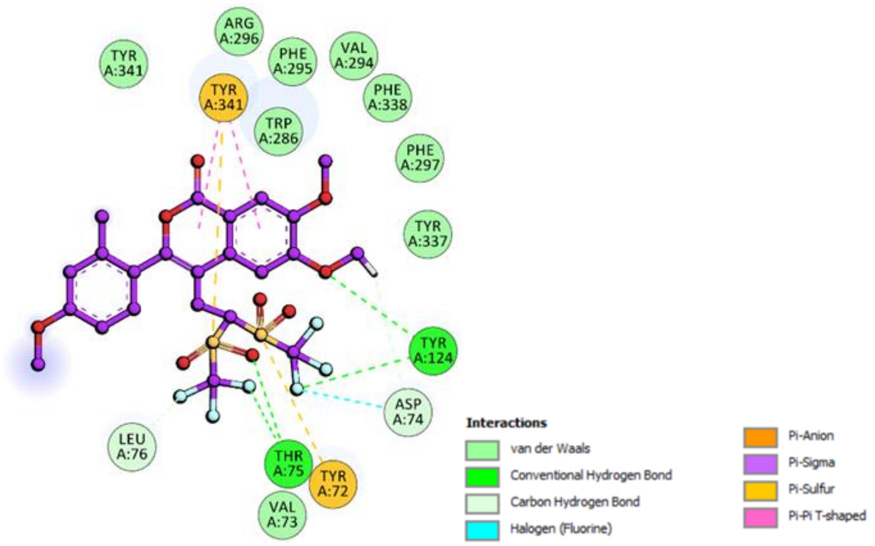

Figure S4. 2D-representation of interactions established by **3l** with hAChE.

Computational Details

To carry out docking simulations, the starting ligand geometries for compounds **3a** and **3l** were built with Discovery Studio (DS) 2022, software package. The resulted molecules were minimized using the adopted-based Newton-Rapson algorithm and the structures were considered fully optimized when the energy changes between interactions were less than 0.01 kcal/mol [2].

The coordinates of hAChE (PDB: 1B41), were obtained from the Protein Data Bank (PDB). The structure was initially processed by "prepare protein" module in DS, to give the structure suitable for docking. AutoDockTools (ADT; version 1.5.7) was used to add hydrogens and partial charges for proteins and ligands using Gasteiger charges. Flexible torsions in the ligands were assigned with the AutoTors module, and the acyclic dihedral angles were allowed to rotate freely. Docking runs were carried out allowing the rotation of Tyr337, Tyr341 Trp286, Tyr124, Trp86, Thr75, Asp74 and Tyr72 receptor residues, using the AutoTors module.

The box center was defined and the docking box was displayed using ADT. The grid box was built with a resolution of 1 Å and 60 x 60 x 72 points and it was positioned at the middle of the protein (x = 116.546; y = 110.33; z = -134.181).

Ligand-protein docking was conducted using the program Autodock Vina [1]. The top ranked conformations for each docked compound were retained and visually inspected for binding pattern analysis, which was visualized and depicted in DS software.

References

1. Trott O, Olson A.J. AutoDock Vina: improving the speed and accuracy of docking with a new scoring function, efficient optimization, and multithreading, J Comput Chem, 31 (2010) 455-461.

2. A. Morreale, F. Maseras, I. Iriepa, E. Galvez, Ligand-receptor interaction at the neural nicotinic acetylcholine binding site: a theoretical model, Journal of Molecular Graphics and Modelling, (2002), 21, 111-118.

Label : Spectrum 1

Collected : 16-feb-2023 12:42 PM

Livetime (s) : 50.00

Real time (s) : 56.72

Detector : X-Max

Window : SATW

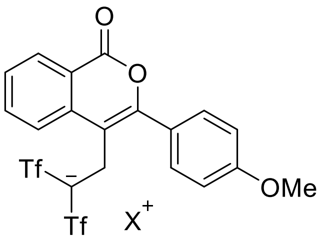

3a

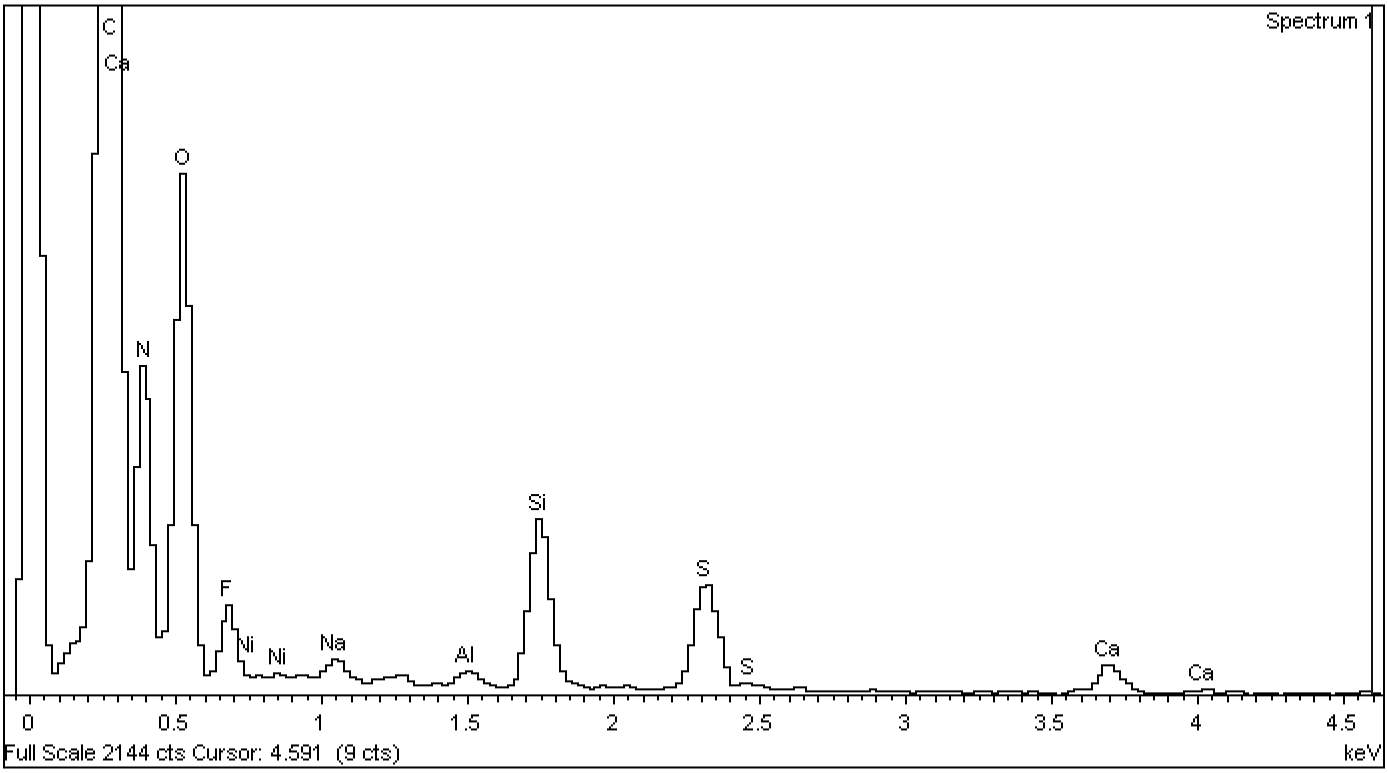

Spectrum processing :

Peaks possibly omitted : 0.521, 2.311, 6.395 keV

Quantitation method : Cliff Lorimer thin ratio section.

Processing option : All elements analyzed (Normalised)

Number of iterations = 2

Standardless

Element	Peak Area	Area Sigma	k factor	Abs Corrn.	Weight%	Weight% Sigma	Atomic%
Na K	241	48	1.191	1.015	8.74	1.63	10.91
Mg K	111	41	1.064	1.011	3.58	1.28	4.23
Si K	2401	91	1.000	1.000	72.14	2.06	73.72
K K	17	27	0.970	0.999	0.49	0.78	0.36

Ca K	528	44	0.953	0.997	15.06	1.20	10.79
Totals					100.00		

Label : Spectrum 4

Collected : 16-feb-2023 01:42 PM

Livetime (s) : 50.00

Real time (s) : 54.02

Detector : X-Max

Window : SATW

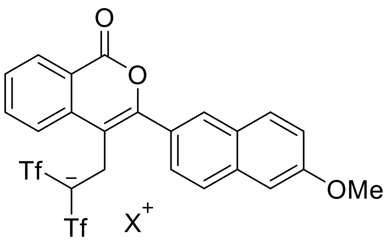

3g

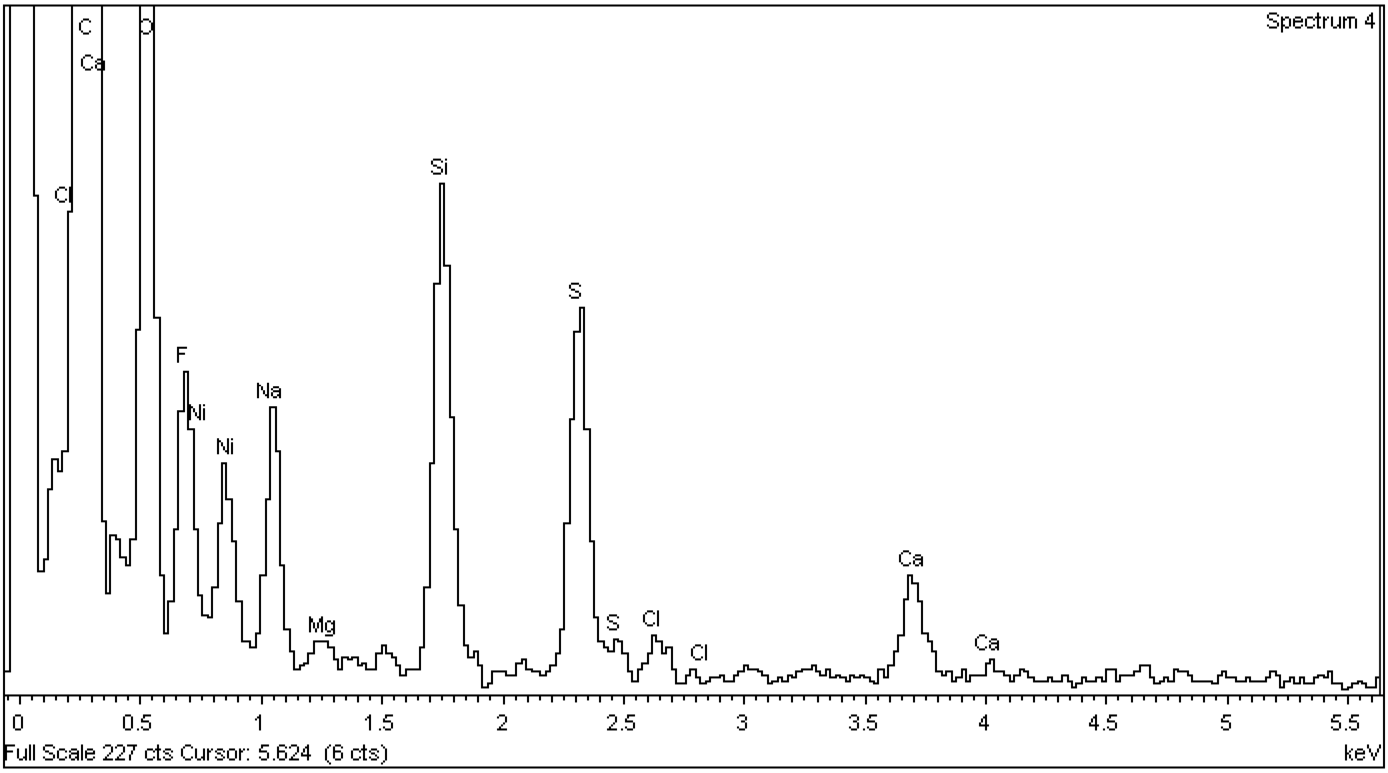

Spectrum processing :

No peaks omitted

Quantitation method : Cliff Lorimer thin ratio section.

Processing option : All elements analyzed (Normalised)

Number of iterations = 3

Standardless

Element	Peak	Area	k	Abs	Weight%	Weight%	Atomic%
	Area	Sigma	factor	Corrn.		Sigma	
F K	441	42	1.752	1.051	32.60	2.39	44.00
Mg K	40	21	1.064	1.015	1.73	0.91	1.83
Si K	702	50	1.000	1.000	28.19	1.86	25.73
S K	679	52	0.959	1.001	26.14	1.85	20.90

Cl K	86	25	0.983	1.004	3.42	0.95	2.47
K K	6	19	0.970	0.997	0.23	0.75	0.15
Ca K	202	31	0.953	0.995	7.69	1.14	4.92
Totals					100.00		

Label : Spectrum 2

Collected : 16-feb-2023 01:57 PM

Livetime (s) : 50.00

Real time (s) : 54.25

Detector : X-Max

Window : SATW

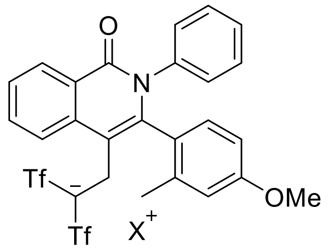

7b

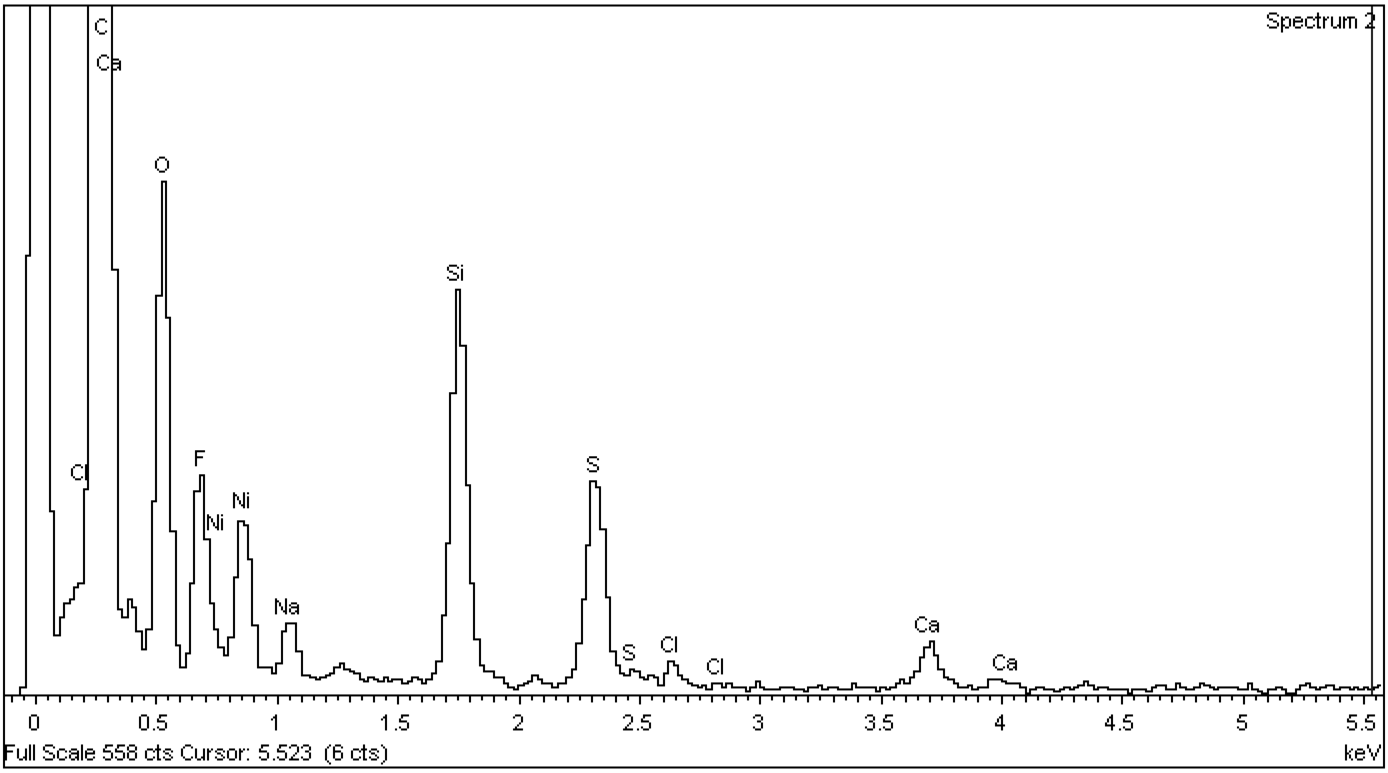

Spectrum processing :

No peaks omitted

Quantitation method : Cliff Lorimer thin ratio section.

Processing option : All elements analyzed (Normalised)

Number of iterations = 3

Standardless

Element	Peak Area	Area Sigma	k factor	Abs Corrn.	Weight%	Weight% Sigma	Atomic%
F K	753	53	1.752	1.046	34.54	1.81	45.61
Mg K	48	25	1.064	1.015	1.31	0.67	1.35
Si K	1367	67	1.000	1.000	34.19	1.53	30.54
S K	946	62	0.959	1.002	22.75	1.37	17.80

Cl K	94	28	0.983	1.004	2.31	0.69	1.63
Ca K	207	32	0.953	0.995	4.91	0.74	3.07
Totals					100.00		
